
JOURNAL INFORMATION
==============================
NLM Title Abbreviation: JBMR Plus
Journal ID: JBMR Plus
NLM Journal ID: 101707013
Journal ID: jbmrplus

JBMR Plus

EISSN: 2473-4039
Publisher: Oxford University Press

ARTICLE INFORMATION
==============================
PMCID: PMC12375535
DOI: 10.1093/jbmrpl/ziaf138
Article ID: ziaf138
Article version: 1
Subjects: Abstracts, AcademicSubjects/MED00010, AcademicSubjects/MED00160, AcademicSubjects/MED00250

Proceedings of the 2025 ORS Musculoskeletal Biology Workshop

Publication date: 2025 Sep
Electronic publication date: 2025 Aug 24
Volume: 9
Issue: Suppl 4
First page: iv1
Last page: iv91
Copyright: © The Author(s) 2025. Published by Oxford University Press on behalf of the American Society for Bone and Mineral Research.
Copyright year: 2025
License: This is an Open Access article distributed under the terms of the Creative Commons Attribution License (https://creativecommons.org/licenses/by/4.0/), which permits unrestricted reuse, distribution, and reproduction in any medium, provided the original work is properly cited.
License URL: https://creativecommons.org/licenses/by/4.0/

Page count: 1

==============================

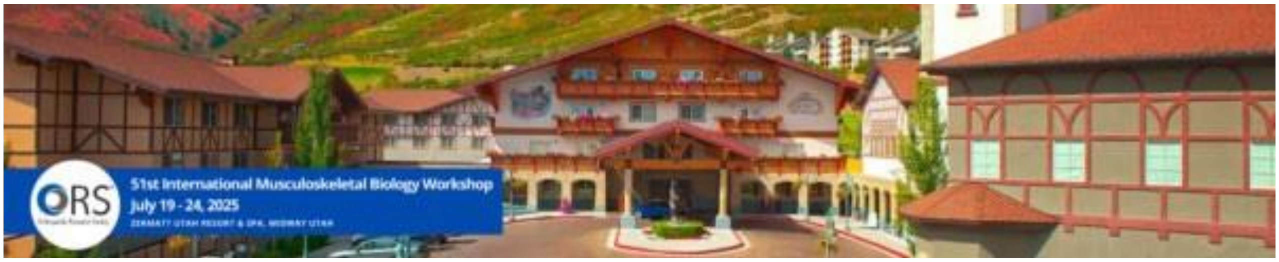

### SCIENTIFIC PROGRAM AND ABSTRACTS

July 2025

This packet contains abstracts presented at the 51st Musculoskeletal Biology Workshop, held July 19 – 24, 2025 in Midway, Utah. The abstracts were prepared by musculoskeletal biologists, engineers, and clinicians, and centered around basic, translational, and clinical research.

The Musculoskeletal Biology Workshop provided an ideal forum to identify problems in musculoskeletal disease and explore experimental approaches that provide solutions. Historically, concepts debated at the Musculoskeletal Biology Workshops have had a strong and lasting impact on the study of skeletal physiology and disorders. This year's meeting drew 74 attendees; seasoned and newer researchers had the opportunity to engage in presentations and informal discussions. The workshop produced simulating and spirited conversations over research findings and breakthroughs. It is widely recognized for its emphasis on training early career investigators and continues to influence scientific thinking in the field of musculoskeletal biology research.

The chairs of the program are below:

Teresita Bellido, PhD
Professor and Chair Dept of Physiology and Cell Biology
University of Arkansas for Medical Sciences (UAMS)
tmbellido@uams.edu
Central Arkansas Veterans Healthcare System Litle Rock, Arkansas
https://medicine.uams.edu/research/hubs/musculoskeletal/

Alexander Robling
Professor and Chair Dept of Anatomy, Cell Biology, and Physiology
Indiana University (IU)
arobling@iupui.edu
RLR VA Medical Center Department of Veterans Affairs
Indianapolis, Indiana

**2025 ORS Musculoskeletal Biology Workshop**
**51st International Musculoskeletal Biology Workshop**
**Zermatt, Utah | July 19 – 24, 2025**

Back to Basics: Bone Remodeling, Skeletal Cell Stimulation, and Mechanisms of MSK Disease Prevention

**Saturday, July 19, 2025**
3:00 PM – 5:30 PM Registration
7:00 PM – 10:00 PM Networking Reception – 51st Anniversary Celebration

**Sunday, July 20, 2025**
7:00 AM – 8:00 AM Breakfast
8:00 AM – 8:10 AM Welcome from the Workshop Co-Directors
Teresita Bellido, PhD (University of Arkansas for Medical Sciences UAMS) and Alex Robling PhD, (Indiana University IU)
8:10 AM – 9:10 AM Session: RIB Award and Lecture, Chronic Diseases and Musculoskeletal Dysfunction, Henry Donahue, PhD (Virginia Commonwealth University)
9:15 AM – 9:50 AM Blue Ribbon Posters, Chair: Teresita Bellido, PhD (UAMS)
9:50 AM – 10:00 AM Break
10:00 AM – 12:00 PM Session: Incretins and Bone. Chair: Elena Ambrogini, PhD (University of Arkansas for Medical Sciences-UAMS and Little Rock VA Medical Center)

- Introduction and goals of the session, Elena Ambrogini, PhD
- Role of Incretins in the Treatment of Obesity and Diabetes, Lisa Morselli, MD, PhD (Medical College of Wisconsin and Milwaukee VA Medical Center)
- Incretins and Bone in Pre-Clinical Models, Guilleume Mabilleau, PhD (University of Angers, France)
- Incretins and Bone in Humans. What Do We Know? Elena Ambrogini, PhD
- Conclusions and Wrap-up, Elena Ambrogini, PhD

12:00 PM – 7:00 PM Free Time *(Lunch and Dinner On Own)*
7:00 PM – 10:00 PM Poster Session and Poster Reception

**Monday, July 21, 2025**
7:00 AM – 8:00 AM Breakfast
8:00 AM – 9:00 AM ASBMR/Harold M. Frost Young Investigator Award Presentations. Chair: Teresita Bellido, PhD (UAMS)

- 8:00 AM - 8:20 AM Insights into Intestinal Pathophysiology and Microbial Ecology in Osteogensis imperfecta, Josephine Tauer (Johannes Kepler University)

- 8:20 AM - 8:40 AM Glutamine Metabolism Through the Hexosamine Biosynthesis Pathway Regulates Mesenchymal Progenitor Cell Fate and Heterotopic Ossification, Heeseog Kang (UT-Southwestern)
- 8:40 AM - 9:00 AM Bone-Adipose Crosstalk may Contribute to Bone Loss during Semaglutide-Induced Weight Loss, Carolyn Chlebek (Maine Health Institute for Research)

9:00 AM – 9:15 AM Break
9:15 AM – 12:00 PM Session: Bone Modeling and Remodeling Revisited. Chair: Thomas LG Andersen, MSc, PhD (University of Southern Denmark, Aarhus University, Odense University Hospital, Denmark)

- Introduction to the Lecture and Speakers, Thomas LG Andersen, MSc, PhD
- New Perspective on Cellular Organization and Regulation of Bone Remodeling, Thomas LG Andersen, MSc, PhD
- Novel Insights into Bone Remodeling Dynamics through Four-Dimensional Imaging of the Rabbit Model. David ML Cooper, BA (University of Saskatchewan, Saskatoon, Saskatchewan, Canada)
- Unified Theory of Bone Modeling and Remodeling, Reinhold G Erben (Ludwig Boltzmann Institute of Osteology, Vienna, Austria)
- Conclusions/Wrap-up, Thomas LG Andersen, MSc, PhD

12:00 PM – 1:00 PM Lunch On Own
1:00 PM – 2:30 PM Career Development Session: How to Find a Post-Doctoral Position: From Academic, National Laboratory, and Industry Perspectives? Joel Boerckel, PhD (UPenn) and Gabriela Loots, PhD (UCDavis), Co-Chairs
2:30 PM – 7:15 PM Free Time and Dinner On Own
7:15 PM – 9:30 PM Session: Osteocytes, Chair: Marc Wein, MD, PhD (Massachusetts General Hospital and Harvard Med School)

- Introduction, Marc Wein, MD, PhD
- Targeting Sclerostin and DKK1 for Developing Treatments for Bone Diseases, David Ke, MD (Angitia Biopharmaceuticals)
- Osteocyte Dendrite Development and Function, Marc Wein, MD, PhD
- Sex-Specific Effects of Muscle-Derived Factors on Osteocytes, Lynda Bonewald, PhD (Indiana University)
- Conclusions, Wrap-Up, and Future Studies, Marc Wein, MD, PhD

**Tuesday, July 22, 2025**
7:00 AM – 8:00 AM Breakfast
8:00 AM – 9:00 AM ASBMR/Harold M. Frost Young Investigator Award Presentations, Chair: Alexander Robling, PhD (IU)

- 8:00 AM - 8:20 AM Understanding the Role of Piezo1 During the Catabolic Stage of Digit Tip Regeneration, Connor Dolan (Arizona State University)
- 8:20 AM - 8:40 AM Piezo1-Mediated Mechanotransduction Controls Osteocyte Maturation and Dendrite Development via a Yap-Ccn-Src Signaling Axis, Yizhong Hu (Harvard School of Dental Medicine)
- 8:40 AM - 9:00 AM Aging and the Mechano-Sensitivity of Bone Healing: Developing an Experimental Approach to Investigate Age-Dependent Mechanobiological Responses in Bone, Neashan Mathavan (ETH Zurich)

9:00 AM – 9:10 AM Break
9:10 AM – 12:00 PM Session: Mechanobiology, Chair: Yingzi Yang, PhD (Harvard School of Dental Medicine)

- Introduction, Yingzi Yang, PhD
- Bone Anabolic Effects of Combined Mechanical Loading and Wnt Targeting, Matt Silva, PhD (Washington University)
- Osteocytic Connexin Hemichannels in Mechanotransduction and Osteogenesis, Jean Jiang, PhD (UT-HSC San Antonio)
- Piezo1-Mediated Mechanotransduction Controls Osteocyte Maturation and Dendrite Development, Yingzi Yang, PhD
- Conclusions and Wrap-Up, Yingzi Yang, PhD

12:00 PM – 1:00 PM Lunch On Own
1:00 PM – 2:30 PM Career Development Session "By and To Early/Mid-Career Investigators": Navigating Transitions in Academia: From PhD to Postdoc to Faculty. Natalia Harasymowicz (University of Utah) and Georgios Kotsaris (University of Pennsylvania), Co-Chairs
2:30 PM – 6:00 PM Free Time
6:00 PM – 8:00 PM Awards and Recognition Dinner Banquet

**Wednesday, July 23, 2025**
7:00 AM – 8:00 AM Breakfast
8:00 AM – 9:00 AM ASBMR/Harold M. Frost Young Investigator Award Presentations, Chair: Teresita Bellido, PhD (UAMS)

- 8:00 AM - 8:15 AM Identification of an Age-Dependent Osteocyte-Osteoblast-Osteoclast Circuitry Using Systems Genetics, Rafiou Agoro (The Jackson Laboratory)
- 8:15 AM - 8:30 AM Inhibition of CDK8 Rescues Impaired Ischemic Fracture Healing Christina Capobianco (University of Michigan)
- 8:30 AM - 8:45 AM LRIG3 Identified by Single-Cell Profiling as a Key Regulator of Skeletal Stem Cell-Mediated Calvarial Morphogenesis and Craniosynostosis, Zhirui Jiang (ADA Forsyth Institute)

- 8:45 AM - 9:00 AM Control of Lipid Metabolism in Chondrocytes by Ppp1r15b is Critical for Skeletal Growth, Aijaz Ahmad John Bhat (University of Massachusetts)

9:00 AM – 9:15 AM Break

9:15 AM – 12:00 PM Session: Osteomyelitis, Chair: Deb Veis, MD, PhD (Washington University, St Louis, US)

- Introduction, Deb Veis, MD, PhD
- Osteoclasts and Osteomyelitis, Deb Veis, MD, PhD
- Using Humanized Mice to Study S. Aureus Mediated Osteomyelitis, Gowrishankar Muthukrishnan, PhD (University of Rochester, NY)
- Biofilms in Osteomyelitis, Ebru Oral, PhD (MGH)
- Conclusions/Wrap up, Deb Veis, MD, PhD

12:00 PM – 7:15 PM Free Time *(Lunch and Dinner On Own)*

7:15 PM – 9:30 PM Session: Pain in Osteoarthritis. Chair: Gabriela Loots, PhD (University of California-Davis)

- Introduction, Gabriela Loots, PhD
- Peripheral Neuroplasticity in Osteoarthritis Pain, Rachel Miller, PhD (Rush University Medical Center, Chicago)
- Ion Channels as Gatekeepers of Pain and Pathology in Osteoarthritis, Chuan-Ju Liu, PhD (Yale University School of Medicine)
- MSK-Neuro Crosstalk in Osteoarthritis, Gabriela Loots, PhD
- Conclusions and Wrap-up, Gabriela Loots, PhD

Henry Donahue, PhD
Virginia Commonwealth University
2025 Remodeling in Bone (RIB) Recipient
51st International Musculoskeletal Biology Workshop
July 19 - 24, 2025
ZERMATT UTAH RESORT & SPA, MIDWAY UTAH
ORS
Orthopaedic Research Society

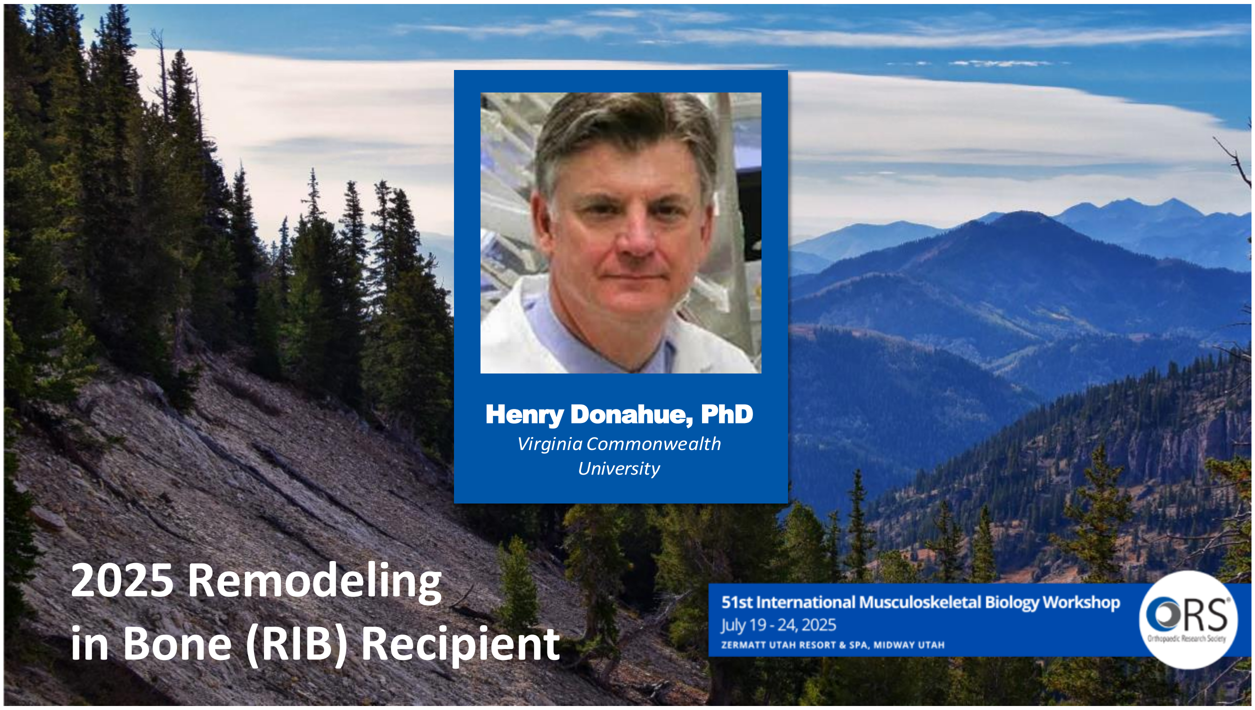

similar bone loss in B6 mice
V/TV

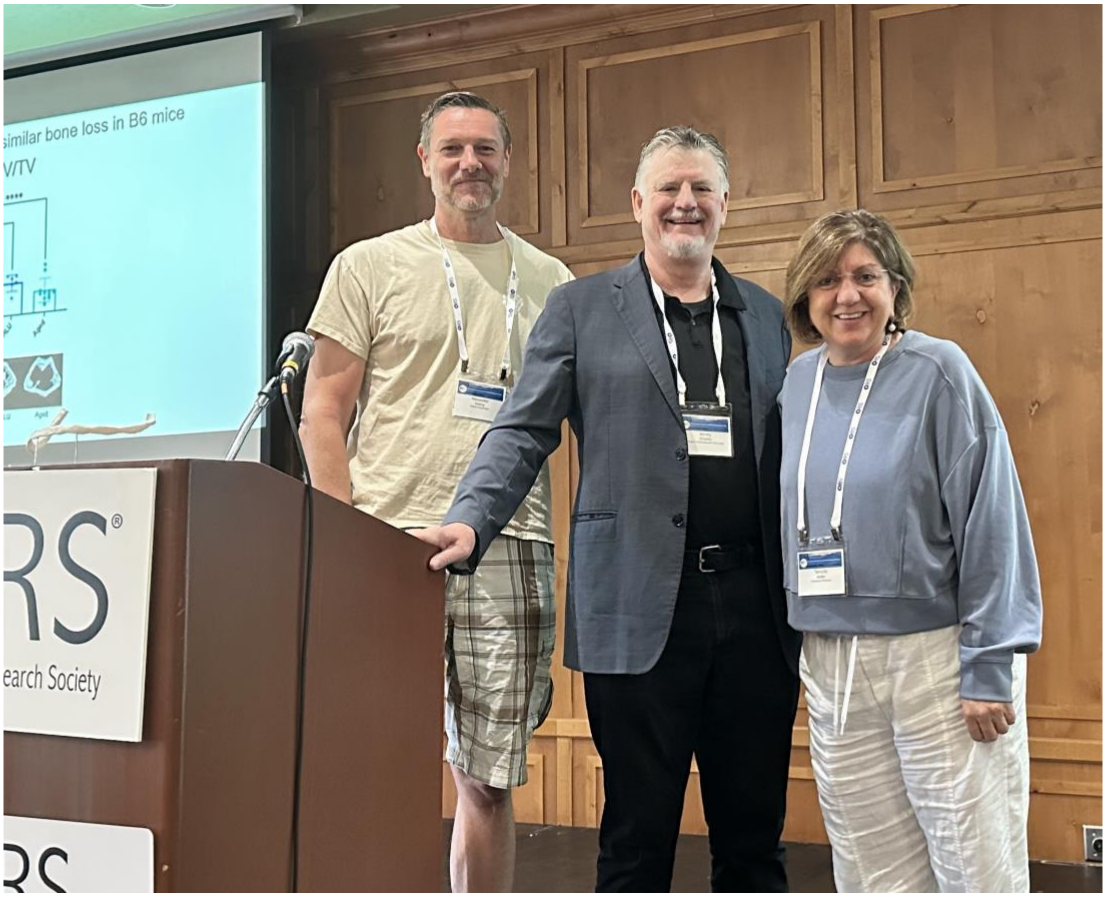

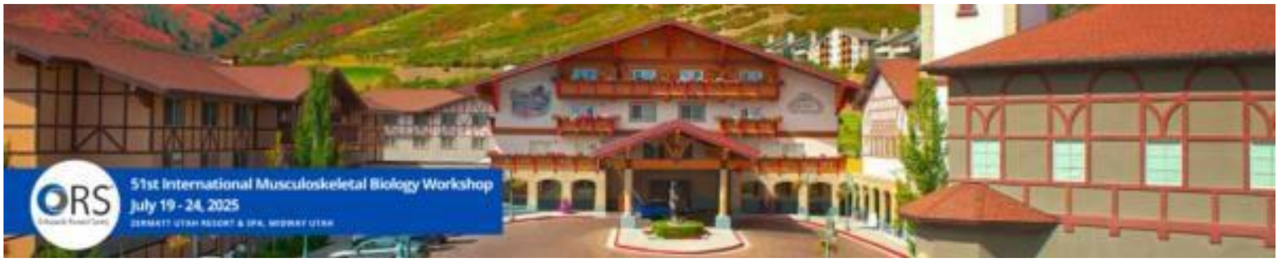

### 2025 ASBMR Harold M. Frost

### Young Investigator Award Recipients

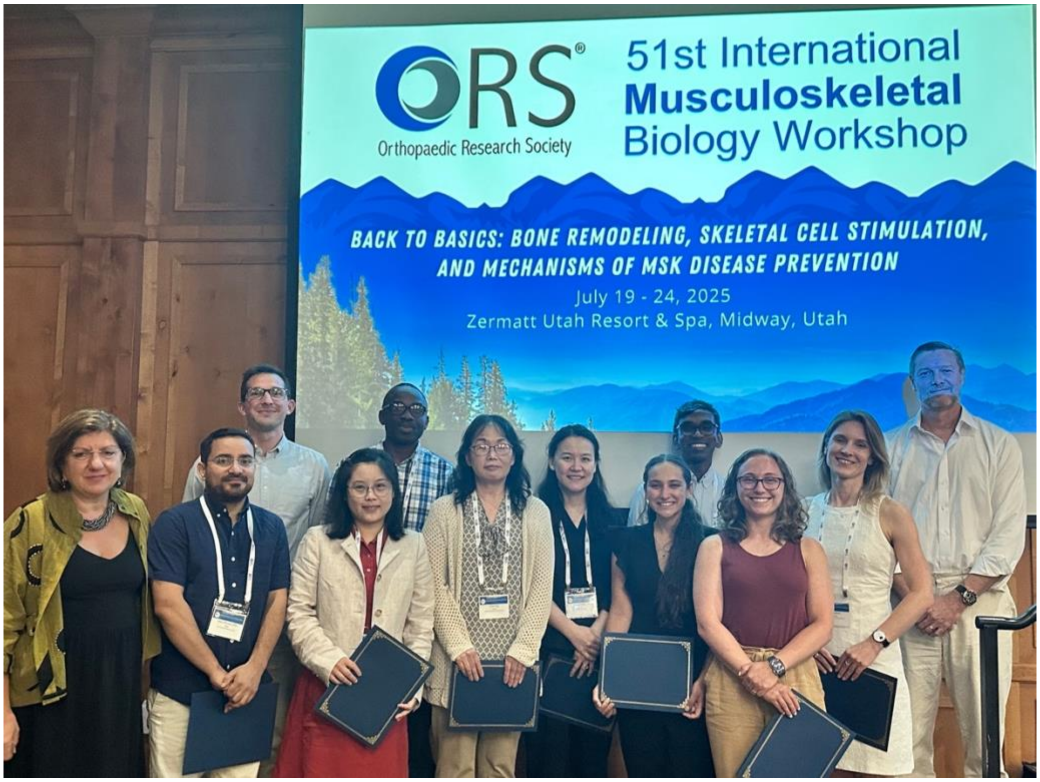

### Abstract: Identification of an Age-Dependent Osteocyte-Osteoblast-Osteoclast Circuitry Using Systems Genetics

*Katie Janeczko*[1], *Daniel M. Gatti*[1], *Gary A. Churchill*[1] *and* ***Rafiou Agoro***[1*]
[1]*The Jackson Laboratory, Bar Harbor, ME 04609, USA; * Corresponding Author and Presenter.*

With aging, the skeleton becomes fragile and unfit for physiological and mechanical activities. Complex combinatorial genetics contribute to skeletal aging. We undertake an approach of tapping on genetic variability and systems genetics approach to determine cell-cell communication patterns in bone microenvironment. Using omics and systems genetics, we pinpointed a coordinated osteocyte-osteoprogenitor-osteoclast circuitry that controls bone remodeling, and this circuitry is disrupted in old bones. We demarcate mouse osteoblasts and osteocytes genes and reveal that PDGFA is expressed in osteocytes whereas PDGFRA were detected in pre-osteoblasts (**Fig. 1a-c**) highlighting a novel PDGFR signaling that may contribute to osteoblast PDGFRA signaling to promote osteoblast differentiation. Further, *Pdgfa* correlates with *Pdgfra* and the osteoblast commitment transcription factor *Sp7* in addition to a strong correlation with a novel bone formation candidate *S1pr3*, a sphingosine 1 phosphate receptor gene located on Chromosome 13. In diversity outbred (DO) mice, *Pdgfra* is positively associated with bone formation. Human GWAS analysis revealed a PDGFRA variant associated with positive bone mineral density (BMD).

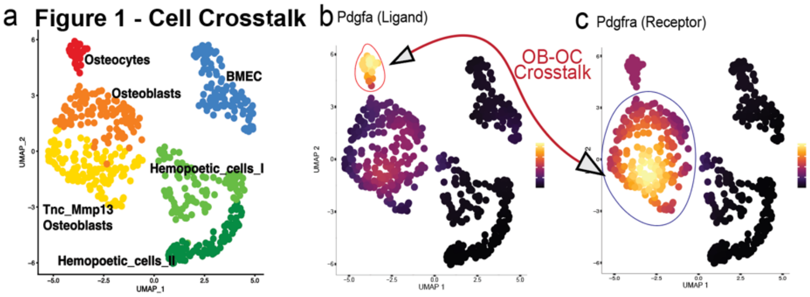

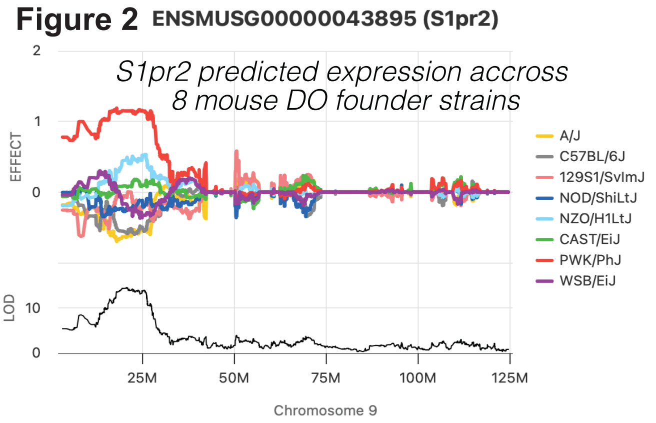

*S1pr3* is one of the five receptors of the sphingosine-1-phosphate (S1P) which is a core component of the sphingolipid machinery. S1pr3 expression is restricted to osteolineage cells with a high expression detected in osteoprogenitors. Expression quantitative trait loci-(QTL) based systems genetics analysis in DO mice revealed that alleles from PWK/PhJ mouse strain drive negative effects on *Pdgfa, Pdgfra,* and *S1pr3* and a positive effect on *S1pr2*. In DO mice, S1pr2 eQTL on chromosome 9 (**Fig. 2**) is positively driven by alleles derived from PWK/PhJ mice. Further, *S1pr2* was the gene with the highest positive correlation with bone collagenase and osteoclast marker *Mmp9*. Among S1prs, S1pr2 was the marker that increase significantly with aging (6, 12, and 18 months) in DO mice.

Because our systems genetics predicted PWK/PhJ mice have high S1pr2 expression, we hypothesize that PWK/PhJ mice have a genetic predisposition that negatively affects bone strength. We intercrossed 8 mouse strains (129S1/SvlmJ, C57BL/6J, BALB/cJ, C58/J, C3H/HeJ, NZB/BlNJ, PWK/PhJ, and SJL/J) and measured BMD at first generation (F1) in 7-8 weeks old male and female mice. Whole BMD (**Fig. 3**) analysis of 63 mouse strains at F1 revealed that PWK/PhJ mouse strain has a dominant negative effect over the other strains. PWK/PhJ mice have a dominant negative effect over the other strains. Interestingly, these low BMD phenotype in PWK/PhJ mice translates onto lower biomechanical proprieties across a panel of different mouse strains. We found that PWK/PhJ male and female mice had lower mechanical behaviors with lower maximum load on load displacement curve and lower area under load-displacement curve to break point.

Because PWK/PhJ mice have low bone, we hypothesize that PWK/PhJ mice are susceptible to greater osteoclastogenesis associated with bone loss and that inhibition of S1PR2 may suppress osteoclast activity in PWK/PhJ osteoclasts. We generated hematopoietic-derived osteoclasts and assessed osteoclastogenesis in C57BL/6J and PWK/PhJ mice. Compared to C57BL/6J, PWK/PhJ osteoclasts have higher proliferation rate in presence of Macrophage colony-stimulating factor (M-CSF). This is associated with high expression of Csf1r in PWK/PhJ osteoclasts. Interestingly, an S1PR2 inhibitor, JTE-013, a potent and selective sphingosine-1-phosphate (S1P) receptor 2 (S1P2) antagonist, decreases osteoclastogenesis in both C57BL/6J osteoclasts and PWK/PhJ osteoclasts. In parallel experiment, by differentiating the pre-osteoblasts MC3T3-E1 towards osteoblasts, we found a downregulation of S1pr2 with osteoblast differentiation. Importantly, pharmacological inhibition of S1PR2 using JTE-013 sustains osteoblast differentiation with elevated *Runx2* expression, *Bglap3*, and *Pth1r*.

In sum, we discover an osteocyte-osteoprogenitor-osteoclast circuitry that controls bone remodeling. We propose PWK/PhJ mouse strain as a useful model to understand the underlying mechanisms of skeletal aging and highlight the need for person-specific combinatorial approach based on genetic background to prevent age-related bone loss.

### Control of lipid metabolism in chondrocytes by *Ppp1r15b* is critical for skeletal growth

*Aijaz Ahmad John[1,2,3], Yeon-Suk Yang[1,2,3], Jun Xie[2,3,4,5], Sachin Chaugul[1,2,3,], Guangping Gao[2,3,4,5] and Jae-Hyuck Shim[1,2,3,4,5*]*

*[1] Department of Genetic and Cellular Medicine, University of Massachusetts Chan Medical School, Worcester, Massachusetts, USA; [2] Department of Medicine, University of Massachusetts Chan Medical School, Worcester, Massachusetts, USA; [3] Horae Gene Therapy Center, University of Massachusetts Chan Medical School, Worcester, Massachusetts, USA; [4]Viral Vector Core, University of Massachusetts Chan Medical School, Worcester, Massachusetts, USA;*

*[5] Li Weibo Institute for Rare Diseases Research, University of Massachusetts Chan Medical School, Worcester, Massachusetts, USA*

### Abstract

Chondrodysplasias is a genetic disorder, characterized by skeletal deformities and growth deficiencies. Dysregulation of endoplasmic reticulum (ER) stress response impairs chondrogenesis, leading to chondrodysplasia. Here, we demonstrate an essential role of the ER stress regulator *Ppp1r15b* in chondrocyte development. PPP1R15B is a protein phosphatase that acts as a constitutive repressor of *eIF2α* phosphorylation. Under ER stress, *eIF2α* phosphorylation increases through two main pathways: elevated phosphorylation by PERK and decreased dephosphorylation resulting from IRE1α-mediated downregulation of PPP1R15B. This dual mechanism contributes to the overall attenuation of translation in response to stress. Remarkably, deletion of *Ppp1r15b* in *Prx1*$^{+}$ skeletal progenitors ($Ppp1r15b^{Prx1}$) dampens chondrocyte development, resulting in disorganized growth plate and reduced trabecular bone, followed by significantly shortened long bones in mice (Fig. 1). Similarly, mice with inducible deletion of *Ppp1r15b* in *Col2+* chondroprogenitors ($Ppp1r15b^{Col2}$) display abnormal skeletal growth (Fig. 2). However, these phenotypes are not seen in mice lacking *Ppp1r15b* in committed osteoblast-lineage cells such as *Osx+* osteoprogenitors ($Ppp1r15b^{Osx}$) and *Dmp1+* mature osteoblasts and osteocytes ($Ppp1r15b^{Dmp1}$), suggesting that PPP1R15B is required for chondrogenesis, but not osteoblast development. Mechanistically, increased phosphorylation of EIF2α by *Ppp1r15b* deletion upregulates the lipogenesis pathway by suppressing Leptin expression (Fig. 3). This is reversed in $Ppp1r15b^{Prx1}$ mice by expression of wildtype Ppp1r15b, not the Ppp1r15b mutant that fails to dephosphorylate EIF2α using recombinant adeno-associated virus (rAAV) (Fig. 4). Similarly, rAAV-mediated expression of Leptin improves skeletal abnormalities in $Ppp1r15b^{Prx1}$ mice (Fig. 5). Collectively, these findings highlights a new molecular action of PPP1R15B regulating cartilage development via the control of Leptin expression.

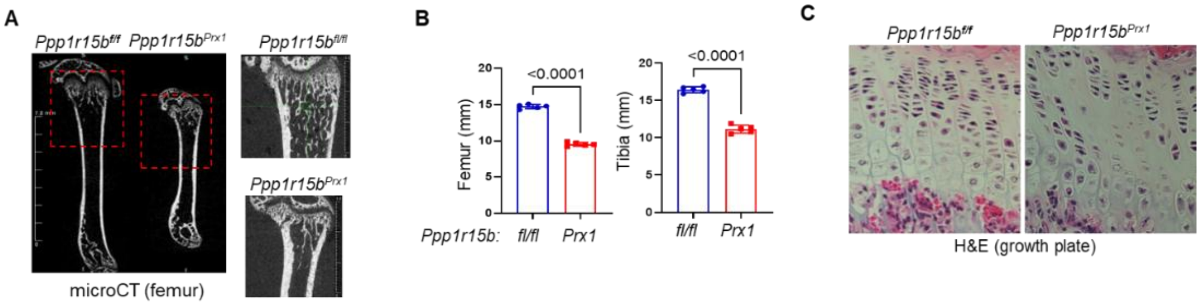

**Figure 1: Loss of *Ppp1r15b* in *Prx1*-expressing cells impairs limb development.** (A left) MicroCT analysis showing 3D-reconstruction images of hindlimbs and femoral trabecular bones of 2-month-old $Ppp1r15b^{Prx1}$ and $Ppp1r15b^{(fl/fl)}$ control mice ($Ppp1r15b^{(fl/fl)}$ n=5; $Ppp1r15b^{Prx11}$, n=5). (A right) MicroCT analysis showing sagittal views of femur of 2-month-old $Ppp1r15b^{(fl/fl)}$ and $Ppp1r15b^{Prx1}$ mice. (B) Measurements of femur and tibia bone lengths in $Ppp1r15b^{prx1}$ mice. (C) Hematoxylin and eosin staining of proximal tibial growth plates reveals severe growth plate disruptions following the deletion of Ppp1r15b in Prx1-expressing cells. A two-tailed unpaired Student's t-test for comparing two groups (B; error bars, data represent mean±SD) was applied.

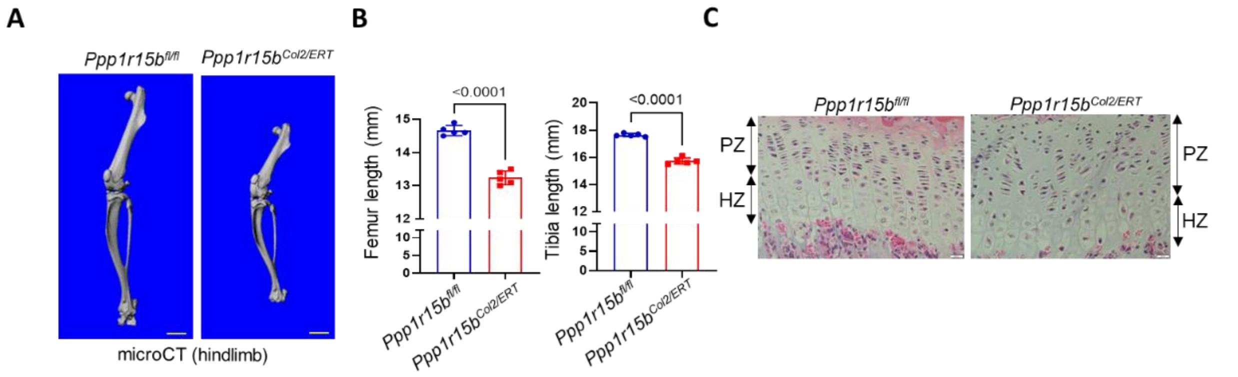

**Figure 2: *Ppp1r15b* deficiency in committed chondroblast-lineage cells (*Col*$^{2+}$ progenitors) results in impaired limb development and growth plate abnormalities.** Deletion of *Ppp1r15b* in $Col^{2+}$ progenitors resulted in significant reduction of tibia and femur lengths. 3D microCT representative images of hindlimbs (A) and their average length (B) (n=5). Hematoxylin and eosin staining of proximal tibial growth plates demonstrates severe disruptions following the deletion of *Ppp1r15b* in *Col2*$^{+}$ progenitor cells. (C) Deletion of *Ppp1r15b* in *Col2*$^{+}$ progenitor cells resulted in a significant expansion of the proliferative zone (PZ) within the proximal tibial growth plate, while the hypertrophic zone (HZ) maintained a normal width. The overall growth plate size increased, accompanied by a significantly reduced number of cells in both the PZ and HZ (n=5). A two-tailed unpaired Student's *t*-test for comparing two groups.

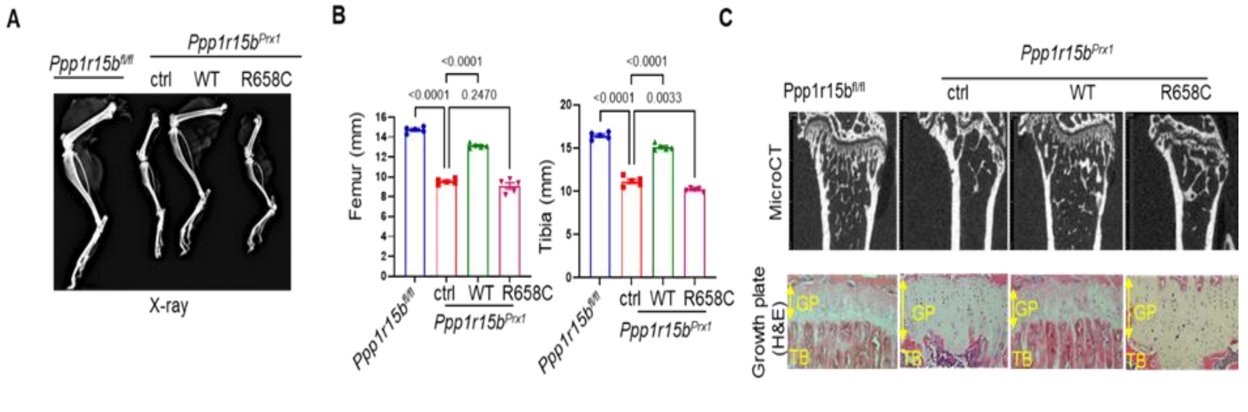

**Figure 3: rAAV-mediated expression of *Ppp1r15b* reverses $Ppp1r15b^{Prx1}$ skeletal phenotypes.** X-ray images of hind limbs (A) and femur and tibia measurements (B) from various rAAV9-treated groups. MicroCT analysis of the femur of 2-month-old AAV-treated $Ppp1r15b^{fl/fl}$ and $Ppp1r15b^{Prx1}$ mice (n=5). Representative microCT images of the femoral bone (C top) are displayed. Longitudinal sections of 2-month AAV-treated $Ppp1r15b^{fl/fl}$ and $Ppp1r15b^{Prx1}$ femurs were stained for H&E (C bottom). one-way ANOVA with Dunnett's multiple comparisons test (B; error bars, SD of biological replicates) for more than two groups was used. GP (growth plate); TB (trabecular bone)

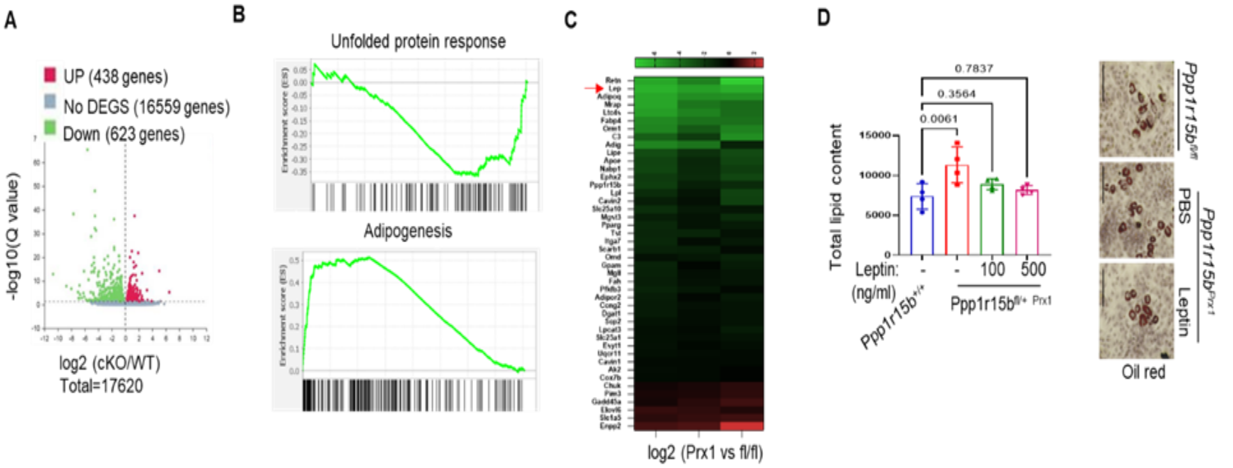

**Figure 4: *Ppp1r15b* deficiency promotes lipogenesis in chondrocytes via *leptin*.** RNA sequencing was performed on FACS-sorted Ppp1r15b$^{+/+}$Prx1-Cre;Rosa$^{mT/mG}$ and Ppp1r15b$^{fl/flPrx1}$-Cre;Rosa$^{mT/mG}$ primary chondrocyte. (A) A volcano plot shows the gene expression for up/downregulated genes in cKO cells relative to WT cells. Gene ontology analysis showing biological processes with gene enrichment in cKO vs WT primary chondrocytes. Gene set enrichment analysis (GSEA) showing the enrichment of genes involved in unfolded response (UPR) (B top) and adipogenesis (B bottom). Heatmaps for adipogenesis associated gene expression. The differentially regulated genes in Ppp1r15b$^{Prx1}$ relative to Ppp1r15b$^{fl/fl}$ are displayed as each row and column represents the sample, respectively. The log2 expression (read count) was centered across samples and red and green denote upregulated and downregulated, respectively (C). Primary chondrocytes from Ppp1r15b$^{fl/fl}$ and Ppp1r15b$^{Prx1}$ pups were cultured and treated with different doses of leptin on day 1 of chondrogenic culture and 7-10 days later analyzed for total lipid content using BODIPY 493/503 staining with flow cytometry (D left), and lipid accumulation using Oil Red O staining (D, right). one-way ANOVA with Dunnett's multiple comparisons test (D left; error bars, SD of biological replicates) was used for more than two groups.

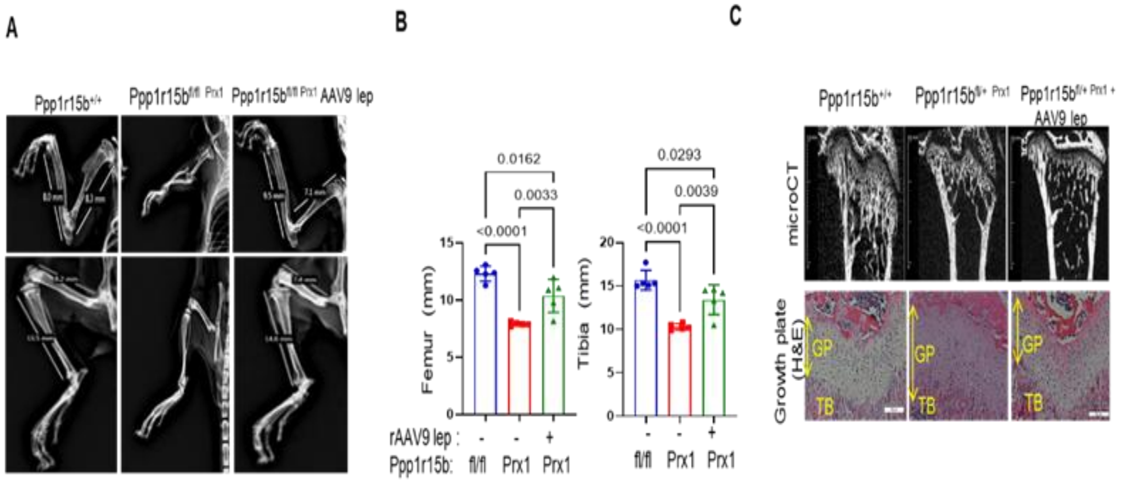

**Figure 5: rAAV-mediated leptin overexpression restored chondrogenesis in Ppp1r15b$^{Prx1}$ skeletal progenitors, nearly rescuing skeletal deformities in forelimbs and hindlimbs.** (A) Whole-body X-ray images of rAAV9-treated groups, and (B) Forelimb and hindlimb bone measurements from different treatment groups. MicroCT analysis of the femur of 2-month-old AAV-treated Ppp1r15b$^{fl/fl}$ and Ppp1r15b$^{Prx1}$ mice (n=5). (C, top) Representative microCT images of the femoral bone. (C, bottom) Longitudinal sections of 2-month AAV-treated Ppp1r15b$^{fl/fl}$ and Ppp1r15b$^{Prx1}$ femurs were stained for H&E. One-way ANOVA with Dunnett's multiple comparisons test (B; error bars, SD of biological replicates) for more than two groups was used. GP (growth plate); TB (trabecular bone)

### Inhibition of CDK8 rescues impaired ischemic fracture healing

*Christina A. Capobianco, MS[1], Michelle J. Song, BS[2], Easton C. Farrell, MS[1], Alexander J. Knights, PhD[1], Karen Kessell, BS[1], Alexis Donneys, MD[1], Jeanna T. Schmanski[2], Luke R. Schroeder[1], Mengqian Chen, PhD[2], Igor B. Roninson, PhD[2], Yadav Wagley, PhD[1], Tristan Maerz, PhD[1], Kurt D. Hankenson, DVM, PhD[1]*
*[1]University of Michigan; Ann Arbor, MI, United States*
*[2]University of South Carolina; Columbia, SC, United States*

INTRODUCTION: While fracture repair typically results in complete restoration of functional bone, approximately 10% of fractures result in a nonunion or malunion, requiring further surgical intervention and treatment [1-3]. Trauma, aging, associated inflammatory dysregulation, and/or smoking place patients at increased risk of ischemia (reduced vascular perfusion) upon fracture[4]. The likelihood of nonunion increases five-fold under ischemic conditions. Despite the clinical recognition of these factors, how altered vascularization negatively impact bone regeneration remains largely unexplored. While it is well established that ischemia results in decreased callus size, inferior mineralization, and exacerbated fibrosis[5], the mechanisms driving pathological fibrosis as opposed to chondrogenesis and osteogenesis, which are necessary for bone repair, have yet to be described. The objective of this study was to identify, describe, and therapeutically target the cellular and molecular perturbations in the early fracture callus during ischemic fracture healing.

We utilized a well-characterized mouse model that mimics vascular injury to assess the effect of ischemia on fracture healing. The model surgically disrupts the femoral blood supply to the lower limb at the time of tibia fracture. We used single-cell RNA sequencing (scRNAseq) to analyze calluses at 4- and 7- days post-fracture (dpf), representing the early phases of stromal progenitor recruitment and differentiation. Consistent with previous histological observations of fibrotic, smaller calluses under ischemic conditions[5], we observed increased fibroblast presence and delayed chondrogenic cell differentiation. Associated with these manifestations, we found *Cdk8* to be highly upregulated by stromal cells under ischemic fracture conditions. While CDK8 has been shown to negatively regulate cellular differentiation[6,7], its role in chondrogenesis has not been described. We demonstrated that pharmacological CDK8 inhibition promoted chondrogenesis *in vitro*, consistent with CDK8's role as a negative regulator of cell differentiation. Finally, in two *in vivo* experiments with different treatment timing, we showed that an orally-available CDK8 inhibitor improved fracture healing by enhancing chondrogenesis and osteogenesis, establishing a translational therapeutic approach to treating ischemic fractures.

METHODS: *Ischemic fracture model:* Under the guidance of a university-wide AAALAC-accredited laboratory animal medicine program, C57 Bl/6 mice underwent tibia fractures at ~18 weeks of age. Tibia were stabilized with a 30G hypodermic needle followed by a transverse fracture generated by dropping a 272-gram weight (3-point bend) to induce a traumatic fracture. To create an ischemic environment, mice underwent a 2mm resection of the femoral artery immediately prior to fracture. At 7 dpf, tibiae were formalin-fixed and paraffin embedded (FFPE), sectioned (5µm), and stained with Safranin-O/Fast Green to assess cartilage formation.

*Single-cell RNA seq of ischemic and control early fracture callus:* At 4 and 7 dpf, calluses were micro-dissected and underwent enzymatic digestion using Collagenase P and Hyaluronidase to generate single cell suspensions. Cells from 8 fracture calluses were pooled together and processed for single cell RNA-sequencing via Parse Biosciences. Trajectory analysis was performed using Monocle3 from an identified aSMA+ stromal progenitor cell population towards hypertrophic chondrocytes under both control fracture and ischemic fracture conditions. A heatmap was generated using genes found to have significant changes ($q\text{-value}<0.05$, Moran's $I>0.25$) in pseudotime in control fracture. The same genes were analyzed in ischemic fracture along pseudotime. Normalized expression was assessed for individual genes related to chondrogenesis and plotted with their 95% CI as a function of pseudotime. Differential gene expression analysis was assessed at 7 dpf and all genes were plotted in a volcano plot. Fibrotic genes significantly upregulated ($p<0.05$) under ischemic conditions were plotted with their log2FC compared to control.

*CDK8 inhibition in vitro:* Human mesenchymal stem cells (hMSC) were placed in a hypoxic incubator for 72 hours. Furthermore, hMSC were pelleted for 3D chondrocyte differentiation in the presence and absence of Senexin B for 14 days. ATDC5 cells additionally underwent chondrogenic differentiation in the presence and absence of CDK8 inhibitors, Senexin B or SNX631-6 for 14 days (not shown). At harvest, all cells were lysed with TRIzol, RNA was extracted, and chondrogenic gene expression was assessed via quantitative real-time PCR (qPCR). hMSC chondrogenic pellets were additionally FFPE and stained with Alcian Blue to assess cartilage formation.

*CDK8 inhibition in vivo:* C57 Bl/6 male mice underwent ischemic left tibia fractures. Mice received oral gavage of the CDK8 inhibitor SNX631-6 at 25 mg/kg (or vehicle) twice a day for 5-10 days post fracture (dpf) or 5-15 dpf. All limbs were µCT scanned and analyzed using Dragonfly software at 15 dpf. Tissues were formalin-fixed, paraffin embedded and stained with Safranin-O/Fast Green. Cartilage was analyzed using ImageJ.

### Bone-Adipose Crosstalk may Contribute to Bone Loss during Semaglutide-Induced Weight Loss

*Carolyn Chlebek[1], Sophia Silvia[2], Benjamin Aaronson[2], Samantha N. Costa[1,3], Ryan Neilson[1], Victoria E. DeMambro[1,3], Clifford J. Rosen[1,3,4]*

*[1]MaineHealth Institute for Research, Scarborough, ME, [2]University of New England College of Osteopathic Medicine, Biddeford, ME, [3]University of Maine, Orono, ME, [4]Tufts University School of Medicine, Boston, MA*

Obesity negatively affects the skeleton, increasing fracture riskr[1]. Dietary interventions are not successful weight loss strategies in most patients with obesity, but novel therapeutic interventions have shown promise. Semaglutide, a glucagon-like peptide-1 receptor agonist (GLP-1Ra), reduces body weight by decreasing food intake[2]. Although calorie restriction causes bone loss[3], the effects of the GLP-1Ras on the skeleton are controversial, with some studies suggesting negative effects on bone mass[4] and others reporting no change[5]. Therefore, we hypothesized that compared to calorie restriction, weight loss with GLP-1Ra treatment would be more detrimental to the skeleton.

Obesity was induced in 8-week-old male and female C57Bl6 with a 60% high fat diet. At 20 weeks-of-age, mice were randomly assigned to 8 weeks of: continued obesity (HFD), 30% caloric restriction (CR), or semaglutide (SEMA). A final group was fed 10% low fat diet (LFD). Body weights were recorded and adipose depots weighed. At the tibia, cortical and trabecular bone morphology were assessed. 4-point bending tests determined cortical strength. Circulating adipokines were quantified in males using lipidomics.

In both sexes, SEMA and CR reduced bodyweight (Fig 1A) and adipose mass. Compared to SEMA, HFD-CR mice lost more weight. SEMA negatively affected the skeleton in males, but not females. In males, SEMA reduced cortical area and ultimate strength (Fig 1B). CR reduced cortical bone mass, but not strength. In females, cortical bone mass and strength did not differ between groups. In males, both HFD and SEMA reduced bone volume fraction compared to LFD. In females, CR increased bone volume fraction and trabecular number but SEMA did not affect trabecular bone morphology.

In HFD-CR males, white adipose tissue mass negatively correlated with bone morphology (Fig 2A). Conversely, in SEMA males, brown adipose tissue mass, normalized to body weight, positively correlated with bone mass. In females, brown adipose mass positively correlated with bone volume fraction in most groups (Fig 2B). In male mice, circulating adipokines varied greatly between all four groups (Fig 2C); circulating cardiolipins, a lipid class necessary for mitochondrial function, were upregulated by SEMA.

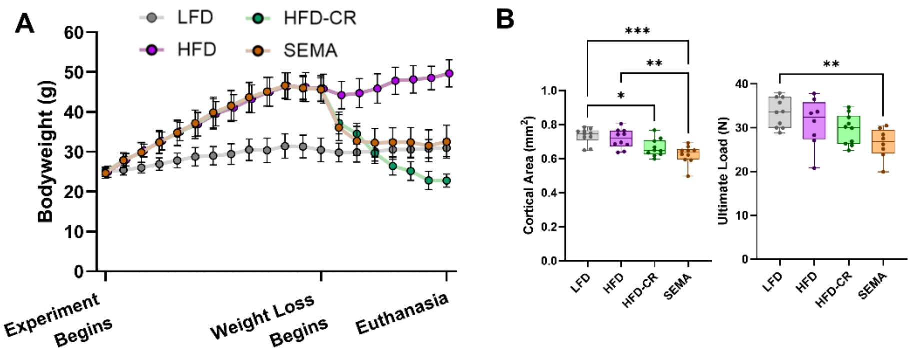

*Figure 1. (A) Bodyweights increased during obesity induction but decreased during weight loss with caloric restriction or semaglutide. (B) In males, semaglutide reduced cortical area and ultimate load at the tibia. Compared to LFD controls, calorie restriction reduced cortical area. Data analyzed by one-way ANOVA and Tukey post-hoc analysis. Males displayed. n=8-10/gp.*

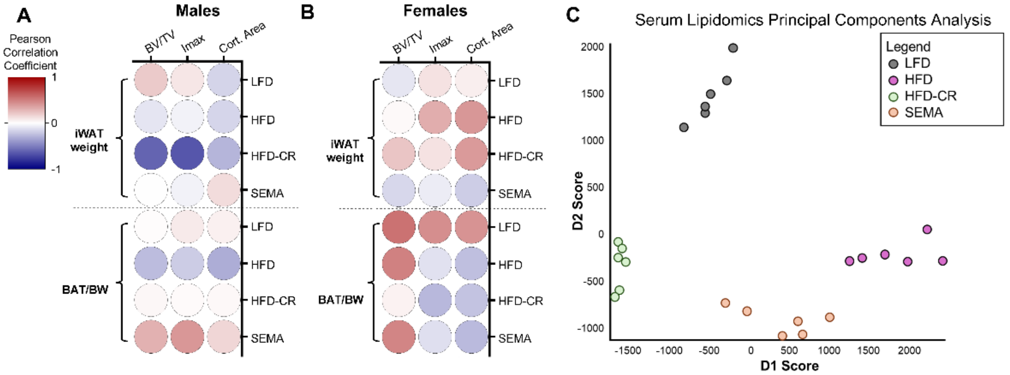

*Figure 2. (A) Inguinal adipose mass (iWAT) negatively correlated with bone morphology in male HFD-CR mice. In SEMA males, normalized brown adipose tissue mass (BAT/BW) positively correlated with bone morphology. n=8-10/gp (B) In females, BAT/BW positively correlated with bone volume fraction in all groups except HFD-CR. n=10/gp. (C) In male mice, circulating lipids varied across all four groups, suggesting distinct adipokine production based on diet and weight loss strategy. Samples were run in duplicate; n=3/gp.*

In summary, semaglutide caused bone loss in male but not female mice. Changes to adipose depots and circulating adipokines may contribute to bone loss during weight loss.

Citations: 1. Rikkonen+ *Osteoporosis Int.* 2021. 2. Gabery+ *JCI Insight* 2020. 3. Liu+ *JBMR* 2024. 4. Hansen+ *eClinical Medicine.*2024. 5. Lv+ *Pharmaceuticals* 2024.

### Understanding the Role of Piezo1 During the Catabolic Stage of Digit Tip Regeneration

Cole B. Dahlstrom, BS[1], *Dana Gaddy, PhD[1]*, *Larry J. Suva, PhD[1]*, ***Connor P. Dolan, PhD**[1,2]*

[1]Texas A&M University, TX, College Station, USA, [2]School of Life Sciences, Arizona State University, AZ, Tempe, USA

Mammals display poor regenerative capabilities and typically respond to traumatic injury with fibrotic wound healing rather than regeneration[1,2]. Although limited, mice and humans successfully regenerate the distal tip of the digit/finger following amputation[3,4]. A substantial amount of work (including our own) has demonstrated that mammals are capable of regeneration after amputation of the digit tip (i.e., the terminal phalanx, **P3**). P3 regeneration proceeds through an overlapping series of interdependent stages[5,6]. However, P3 regeneration can more simply be divided into 2 stages: **catabolic** and **anabolic**. The catabolic stage includes inflammation and bone resorption, whereas the anabolic stage includes blastema formation and *de novo* bone regeneration (**Fig. 1**).

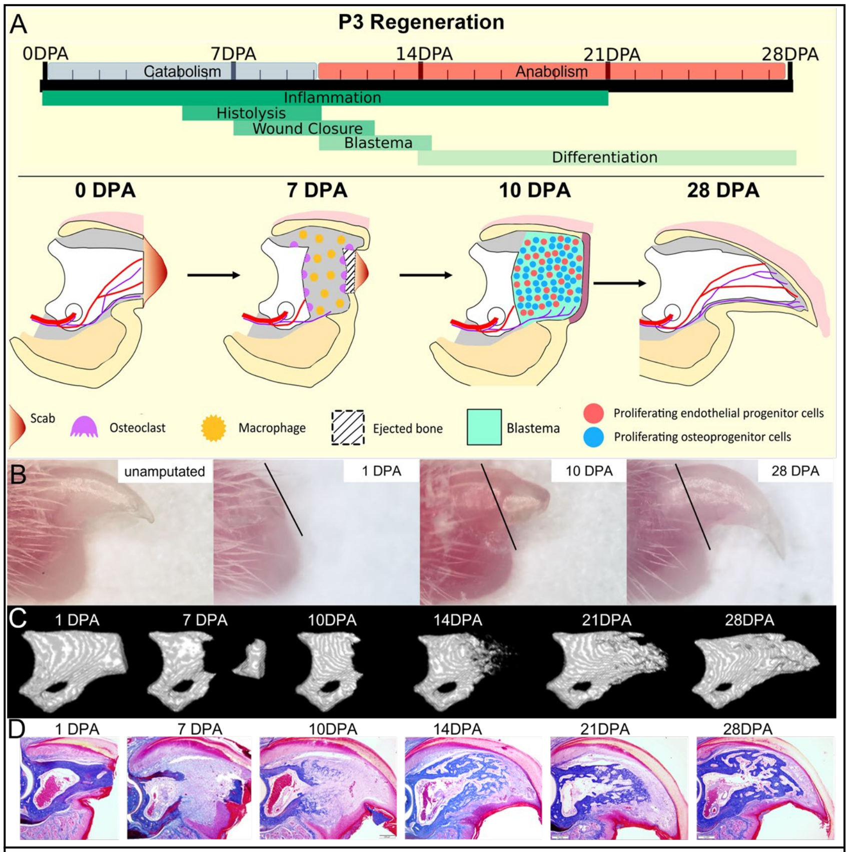

**Figure 1: Overview of P3 Regeneration.** A) Schematic of P3 regeneration. B) Pictures of P3 regenerating. C) Longitudinal μCT images of a P3 regenerating. D) Histology of P3 regeneration

Decreased mechanical load has been shown to diminish regeneration in non-mammalian animals, such as salamanders[7], zebrafish[8], and planaria[9]. Mouse ambulation generates mechanical forces experienced by the phalangeal elements[10]. Based on this, and the extensive literature demonstrating the role of mechanical load in skeletal physiology, we investigated whether mechanical load was required for P3 regeneration. To test this, we used a well-established hindlimb unloading (<u>**HU**</u>) model in which the hindlimbs are suspended such that they are non-weight bearing during regeneration[11–13]. To determine if P3 regeneration is mechanical load-dependent, the distal digit tips of mice were amputated and immediately exposed to 4 weeks of HU. *In vivo* micro-computed tomography (μCT) monitoring of HU regenerates ($HU^{Amp}$) demonstrated a complete inhibition of P3 bone regeneration compared to load-bearing amputation (Amp) controls[14] (**Fig. 2**). **These data demonstrate the significant inhibition of P3 regeneration in the absence of mechanical load.**

Because $HU^{Amp}$ digits display defects in all regeneration stages, we conducted experiments to determine whether decreased load impaired the <u>*catabolic and/or the anabolic stage*</u> of the P3 regeneration response. To assess the role of mechanical load on the catabolic stage (i.e., inflammation, bone resorption, and wound healing) mice were placed in HU during the early stages of regeneration (0-12 DPA), then removed from HU and allowed to ambulate for an additional 4 weeks ($HU^{0\text{-}12\ DPA}$). As in $HU^{Amp}$ mice, $HU^{0\text{-}12\ DPA}$ digits exhibited impaired bone resorption response compared with Amp control digits at 12 DPA. When removed from HU and allowed to re-ambulate, digits progressed through a regeneration sequence that was temporally shifted by the period of HU treatment, but otherwise paralleled the regeneration sequence of control P3 regenerates[14] (**Fig. 5**). This response to re-ambulation induced a level of bone resorption and new bone formation with a final bone volume and bone length comparable to controls[14]. <u>These results demonstrate</u>

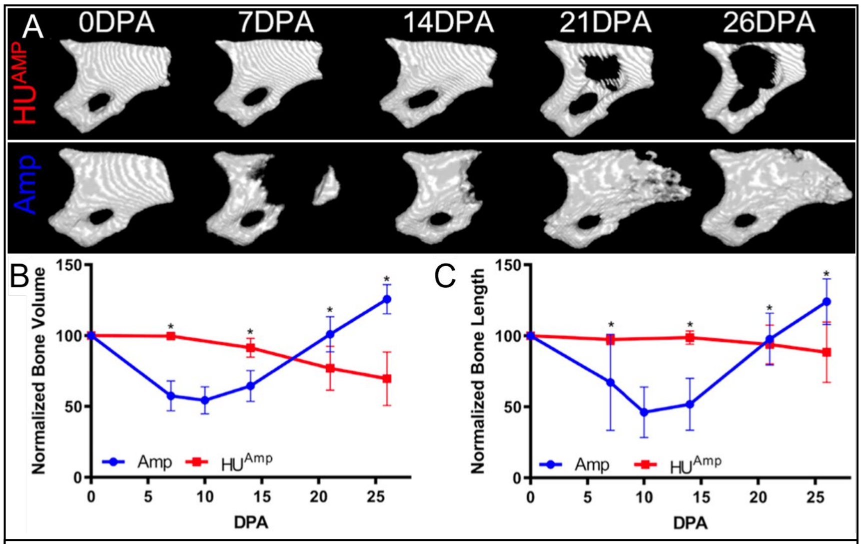

**Figure 2:** HU Inhibits P3 Regeneration that HU inhibits early regenerative events but that the inhibition is reversible, and regeneration is rescued by the restoration of mechanical load.

Presently, *a major gap in our knowledge* is the lack of understanding how mechanical load is recognized and interpreted at the cellular level during P3 regeneration. However, our recent work has focused on the role of Piezo1, a mechanosensitive transmembrane protein that non-selectively allows cations to move intracellularly in response to direct mechanical forces[15]. Moreover, Piezo1 is critical in the musculoskeletal system, and recent work has demonstrated that it is important during skeletal development, homeostasis, and fracture repair[16–19].

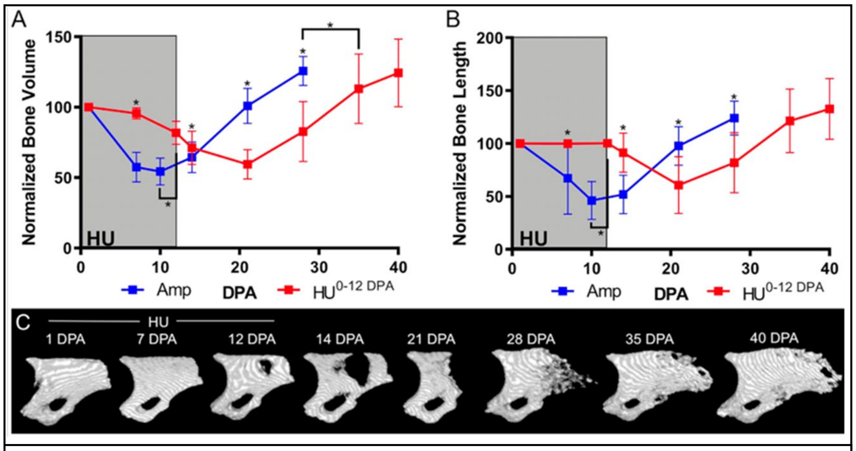

**Figure 3:** Mechanical Load is Critical during the Catabolic and Anabolic Stages of P3 Regeneration

Because **1)** Piezo1 is critical for mechanotransduction and **2)** P3 regeneration is mechanical load-dependent, we investigated whether *Piezo1* was expressed during P3 regeneration. By investigating publicly available single-cell RNA sequencing data sets collected at various stages of P3 regeneration[20,21]. Following amputation, the percentage of osteoblasts and fibroblasts expressing *Piezo1* increases, as does the percentage of cells expressing components of the Piezo1 signaling pathway (*data not shown*). We next sought to confirm that Piezo1 was expressed during P3 regeneration. In the unamputated digit, Piezo1 is expressed at low levels in the P3 bone, surrounding connective tissue, and articular chondrocytes (data not shown). At 7 DPA, Piezo1 is expressed throughout the bone marrow and P3 bone (**Fig. 4A**). At 12 DPA, when the blastema is fully formed, Piezo1 is expressed by the majority of blastemal cells which predominantly consists of osteoprogenitors cells[6] (**Fig. 4B**). We next investigated whether Piezo1 expression was altered by HU. Piezo1 expression was increased in $HU^{Amp}$ mice compared to ambulatory controls (**Fig. 4C-E**). Increased Piezo1 expression in $HU^{Amp}$ digits suggests that cells are responding to decreased mechanical load.

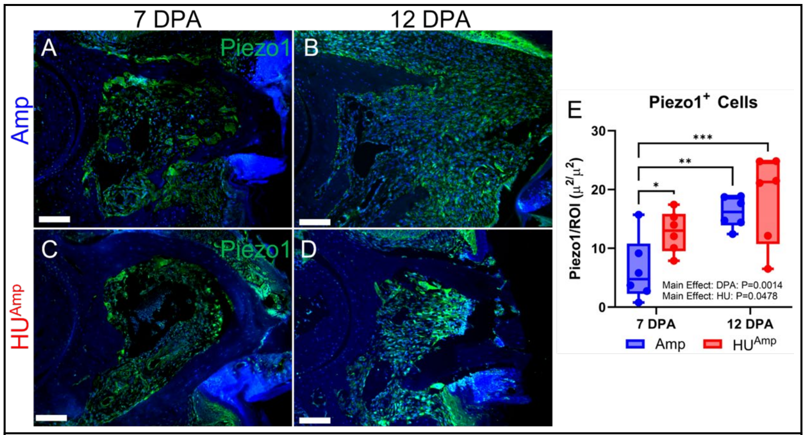

**Figure 4:** Piezo1 Expression During P3 Regeneration

We next sought to determine if P3 regeneration could be rescued by stimulating Piezo1 without mechanical stimulation (i.e., ambulation). Yoda1 is a synthetic agonist which opens Piezo1 allowing for cation influx through the cell membrane[22]. Yoda1 has been shown to accelerate fracture repair and prevent bone loss during HU[23]. To test this hypothesis, we conducted an exploratory study in which mice were hindlimb unloaded and received daily IP injections of Yoda1 (5µM; $HU^{Yoda1}$) or vehicle ($HU^{Veh}$; 5% Ethanol), and P3 regeneration was monitored for 12 days (i.e., the catabolic stage). $HU^{Yoda1}$ mice had increased osteoclast-mediated bone resorption compared to $HU^{Veh}$ mice at 12 DPA, and which was evident as early as 5 DPA (**Fig. 5**). ***Critically, $HU^{Yoda1}$ mice experience bone resorption comparable to ambulatory control animals treated with vehicle*** (**$Amp^{Veh}$; Fig. 5**). These data indicate that Yoda1 is sufficient to **1)** recruit monocytes to the amputation wound and **2)** enable monocytes to differentiate into osteoclasts capable of resorbing bone. ***Taken together, our data demonstrates that Piezo1 is directly responsible for mechanotransduction, and that stimulating Piezo1 with Yoda1 rescues the catabolic stage of P3 regeneration that is compromised by HU.***

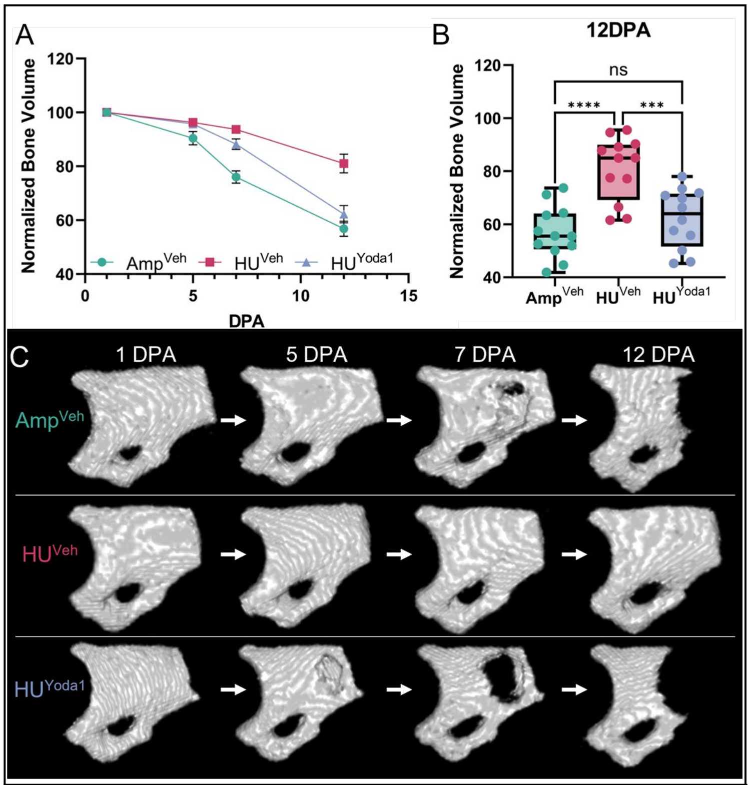

**Figure 5:** Yoda1 Treatment Rescues the Catabolic Stage of P3 Regeneration in Hindlimb Unloaded Mice

<u>References</u>

1. Dawson, L. A. *et al.* Analogous cellular contribution and healing mechanisms following digit amputation and phalangeal fracture in mice. *Regeneration* **3**, 39 (2016).
2. Turner, N. J., Johnson, S. A. & Badylak, S. F. A histomorphologic study of the normal healing response following digit amputation in C57bl/6 and MRL/MpJ mice. *Arch. Histol. Cytol.* **73**, 103–111 (2010).
3. Han, M., Yang, X., Lee, J., Allan, C. H. & Muneoka, K. Development and regeneration of the neonatal digit tip in mice. *Dev. Biol.* **315**, 125–135 (2008).
4. Illingworth, C. M. Trapped fingers and amputated finger tips in children. *J. Pediatr. Surg.* **9**, 853–858 (1974).
5. Dolan, C. P., Dawson, L. A. & Muneoka, K. Digit Tip Regeneration: Merging Regeneration Biology with Regenerative Medicine. *Stem Cells Transl. Med.* **7**, 262 (2018).
6. Fernando, W. A. *et al.* Wound healing and blastema formation in regenerating digit tips of adult mice. *Dev. Biol.* **350**, 301 (2010).
7. Mitashov, V. I., Brushlinskaya, N. V., Grigoryan, E. N., Tuchkova, S. Y. & Anton, H. J. Regeneration of organs and tissues in lower vertebrates during and after space flight. *Adv. Space Res.* **17**, 241–255 (1996).
8. Recidoro, A. M. *et al.* Botulinum Toxin Induces Muscle Paralysis and Inhibits Bone Regeneration in Zebrafish. *J. Bone Miner. Res.* **29**, 2346–2356 (2014).
9. Morokuma, J. *et al.* Planarian regeneration in space: Persistent anatomical, behavioral, and bacteriological changes induced by space travel. *Regeneration* **4**, 85–102 (2017).
10. Mendes, C. S. *et al.* Quantification of gait parameters in freely walking rodents. *BMC Biol.* **13**, 50 (2015).
11. Ferreira, J. A., Crissey, J. M. & Brown, M. An Alternant Method to the Traditional NASA Hindlimb Unloading Model in Mice. *J. Vis. Exp.* 2467 (2011) doi:10.3791/2467.
12. Globus, R. K. & Morey-Holton, E. Hindlimb unloading: rodent analog for microgravity. *J. Appl. Physiol.* **120**, 1196–1206 (2016).
13. Morey-Holton, E. R. & Globus, R. K. Hindlimb unloading rodent model: technical aspects. *J. Appl. Physiol.* **92**, 1367–1377 (2002).
14. Dolan, C. P. *et al.* Mouse Digit Tip Regeneration Is Mechanical Load Dependent. *J. Bone Miner. Res. Off. J. Am. Soc. Bone Miner. Res.* **37**, 312–322 (2022).
15. Coste, B. *et al.* Piezo1 and Piezo2 Are Essential Components of Distinct Mechanically Activated Cation Channels. *Science* **330**, 55–60 (2010).
16. Li, X. *et al.* Stimulation of Piezo1 by mechanical signals promotes bone anabolism. *eLife* **8**, e49631 (2019).
17. Qin, L. *et al.* Roles of mechanosensitive channel Piezo1/2 proteins in skeleton and other tissues. *Bone Res.* **9**, 44 (2021).
18. Wang, L. *et al.* Mechanical sensing protein PIEZO1 regulates bone homeostasis via osteoblast-osteoclast crosstalk. *Nat. Commun.* **11**, 282 (2020).
19. Zhou, T. *et al.* Piezo1/2 mediate mechanotransduction essential for bone formation through concerted activation of NFAT-YAP1-ß-catenin. *eLife* **9**, e52779 (2020).
20. Storer, M. A. *et al.* Acquisition of a Unique Mesenchymal Precursor-like Blastema State Underlies Successful Adult Mammalian Digit Tip Regeneration. *Dev. Cell* **52**, 509-524.e9 (2020).
21. Johnson, G. L., Masias, E. J. & Lehoczky, J. A. Cellular Heterogeneity and Lineage Restriction during Mouse Digit Tip Regeneration at Single-Cell Resolution. *Dev. Cell* **52**, 525-540.e5 (2020).
22. Botello-Smith, W. M. *et al.* A mechanism for the activation of the mechanosensitive Piezo1 channel by the small molecule Yoda1. *Nat. Commun.* **10**, 4503 (2019).
23. Liu, Y. *et al.* Mechanosensitive Piezo1 is crucial for periosteal stem cell-mediated fracture healing. *Int. J. Biol. Sci.* **18**, 3961–3980 (2022).

### Piezo1-Mediated Mechanotransduction Controls Osteocyte Maturation and Dendrite Development via a Yap-Ccn-Src Signaling Axis

*Yizhong Jenny Hu, PhD[1], Xinchen Wu, PhD[1], Fuhua Wang[1], Yu Jin, PhD[1], Yangchen Jin[1], Yuchen Liu, PhD[1], Qian Cong, PhD[1], Yingzi Yang, PhD[1]*
*[1]Harvard School of Dental Medicine, MA*

Mechanosensing and mechanotransduction are essential for maintaining skeletal health[1]. While anabolic mechanical loading promotes bone formation, both insufficient and excessive loading can lead to pathological conditions[2]. Osteocytes—mechanosensitive cells derived from terminally differentiated osteoblasts—are the most abundant cell type in adult bone and play a central role in coordinating bone-forming osteoblasts and bone-resorbing osteoclasts[3]. Embedded deep within the mineralized matrix, osteocytes extend long, interconnected dendritic processes that enable them to detect and respond to mechanical cues, including matrix strain and fluid shear stress[4]. These dendritic structures deteriorate with age and in osteoporotic bone, highlighting their critical role in maintaining bone homeostasis[5]. Several studies have identified signaling pathways required for the integrity of osteocyte dendrites and the perilacunar network (PLN)[6]. However, the processes by which osteocytes mature from osteoblasts and acquire their characteristic dendritic morphology remain poorly understood.

By analyzing developing mouse long bones, we observed progressive maturation of the osteocyte dendritic network in terms of density, length, and orientation. This structural maturation was spatially correlated with the transcriptional transition of osteoblasts into osteocytes and with increasing bone matrix stiffness, as measured by microscopy-guided bone microindentation testing. In an *in vitro* model of osteocyte mechanotransduction using OCY454 cells, both extracellular matrix (ECM) stiffening and fluid shear stress (FSS) increased dendrite number and length. Genetic deletion of Piezo1, a mechanically activated ion channel, in late osteoblasts and osteocytes (*Piezo1* cKO) impaired osteocyte differentiation, disrupted formation of the dendritic and perilacunar network, and reduced bone mass. Moreover, *Piezo1* deletion abolished the enhancement of dendrite formation in response to ECM stiffening and FSS, suggesting that Piezo1-mediated mechanosensing is a key driver of the transcriptional and morphological transition from osteoblasts to osteocytes.

Bulk RNA-sequencing revealed activation of Hippo/Yap signaling and suppression of Wnt/β-catenin signaling during osteocyte differentiation—effects that were reversed upon *Piezo1* deletion. Pharmacological activation of Yap using TDI-011536 and inhibition of Wnt signaling via LGK974 or recombinant human sclerostin (rhSOST) each promoted osteocyte dendrite formation. Combined Yap activation and Wnt inhibition produced more pronounced effects, enhancing dendrite formation than either treatment alone. Interestingly, Yap activation suppressed the expression of canonical Wnt target genes, suggesting that downstream of Piezo1, Yap may act upstream to inhibit Wnt signaling. *In vivo*, genetic activation of Yap modulated the osteocyte dendritic network and bone mass in a dose- and temporally dependent manner. Intermittent Yap activation increased dendrite number while preserving dendrite length and orientation, and enhanced bone mass by promoting lamellar, well-organized bone formation, all while maintaining normal bone morphology and osteocyte differentiation. In contrast, sustained Yap activation disrupted the dendritic network, led to disorganized woven bone, aberrant osteoblast activation, and impaired osteocyte differentiation. Importantly, intermittent Yap activation fully rescued cortical and trabecular bone mass in *Piezo1* cKO mice and restored the osteocyte dendritic and perilacunar network to normal levels. In comparison, Wnt inhibition only partially restored the dendritic network and failed to rescue bone mass, highlighting Yap activation as the primary downstream effector of Piezo1-mediated mechanotransduction in regulating osteocyte dendrite formation and skeletal integrity.

Although Yap activation fully rescued the dendritic and bone defects associated with Piezo1 loss, its oncogenic potential limits the feasibility of direct Yap activation as a therapeutic strategy for treating unloading-induced skeletal disorders[7]. To identify safer downstream targets of Piezo1 and Yap signaling, we explored alternative strategies to enhance the osteocyte dendritic network and bone mass. Among canonical Yap target genes, *Cyr61* and *Ctgf* encode the secreted, extracellular matrix–associated proteins Ccn1 and Ccn2, respectively. We found that non–cell-autonomous overexpression of Ccn1 and Ccn2 promoted dendrite outgrowth and rescued dendritic defects caused by *Piezo1* loss. Mechanistically, we found Ccn1/2 activated the integrin-associated protein Src kinase, which regulated actin polymerization and cytoskeletal remodeling. Activated Src further enhanced Yap signaling, establishing a feedforward loop that amplified dendrite formation. Remarkably, systemic overexpression of Ccn1/2 from the liver rescued osteocyte dendritic defects and cortical bone mass in *Piezo1* cKO mice, indicating their central role in mediating dendrite formation and skeletal integrity downstream of Piezo1-Yap signaling. These findings identify Yap and its downstream effectors Ccn1 and Ccn2 as promising therapeutic targets for skeletal disorders induced by mechanical unloading and characterized by osteocyte dendritic dysfunction.

**References**: [1]Mamato et al., 2010. [2]Stavnichuk et al., 2020. [3]Schaffler et al., 2014.[4]McNamara et al., 2009. [5]Milovanovik et al., 2013. [6]Wang et al., 2021. [7]Piccolo et al., 2023.

### LRIG3 Identified by Single-Cell Profiling as a Key Regulator of Skeletal Stem Cell-Mediated Calvarial Morphogenesis and Craniosynostosis

*Zhirui Jiang[1], Kai Li[1], H-M Ivy Yu[1, 5], Takamitsu Maruyama[1, 5], Ang Cui[2, 3], Clinton S Morrison[5], and Wei Hsu[1,2,3,4,5]*

*[1]The ADA Forsyth Institute, [2]Faculty of Medicine of Harvard University, [3]Harvard School of Dental Medicine, [4]Harvard Stem Cell Institute, 245 First Street, Cambridge, MA 02142*

*[5]University of Rochester Medical Center, 601 Elmwood Avenue, Rochester, NY 14642*

**Introduction:** Intramembranous ossification, essential for craniofacial bone formation, operates through a process distinct from that of endochondral bone in the body skeleton (1). Cranial suture mesenchyme harbors a population of skeletal stem cells naturally programmed for osteoblast differentiation to form intramembranous bones (2). Previous studies have identified Axin2-expressing cells within the suture mesenchyme as essential mediators of calvarial bone development, homeostasis, and regeneration (2). These cells exhibit hallmark stem cell properties, including self-renewal, multipotency, and clonal expansion. However, transplantation-based, stem cell-mediated regeneration studies suggest heterogeneity in this Axin2+ cell population. Thus, it is necessary to examine the relevant cellular heterogeneity to investigate further the regulation of SuSC stemness and osteogenic potential for stem cell expansion, and to identify cell surface markers for stem cell purification. Our objectives in the current study were to investigate transcriptional landscape and cellular heterogeneity inherent to the Axin2-expressing suture cell population using an unbiased single-cell transcriptomic approach.

**Method:** *Animals*- With IACUC approval, *Axin2GFP* (genotype: *Axin2-rtTA*; *TRE-H2BGFP*), and *Axin2Cre-Dox* (genotype *Axin2-rtTA*; *TRE-Cre*) and B6;129P2-*Lrig3Gt(LST016)Byg*/Mmucd (*Lrig3* mutant) mouse strain were used (2,4). To induce Cre activity, Dox (2 mg ml-1 plus 50 mg ml-1 sucrose) was provided in the drinking water for 3 days (2). Primary mesenchymal cells containing SuSCs were isolated from calvarial tissue containing the sagittal suture of $Axin2^{GFP}$ or Lrig3 mice. The calvarial injury model was established by the creation of a circular lesion about 2 mm in diameter using a micro-drill (2). *MicroCT, histology and staining*- mouse skulls were imaged at 6 µm resolution by Skyscan 1271 scanner, followed by FFPE or cryosectioning, H&E, LRIG3, GLI1, TWIST1, OSX, BMPR1A or KI67 antibodies staining. *scRNA-seq analysis*- Sorted GFP+ primary suture mesenchymal cells were subjected to library construction and sequencing. After QC filtering, unsupervised clustering was conducted by Seurat, GO/KEGG/Reactome term enrichment, RNA velocity, trajectory analysis and cell-cell interaction were analyzed by Clusterprofiler/DAVID, Velocyto, Monocle3, and CellChat, respectively. *Statistics*- Data are expressed as mean±SD and analyzed by t-tests.

**Results:** UMAP plot revealed six clusters identified from scRNA-seq on 10,594 Axin2+ cells exhibiting distinct gene expression profiles, including neutrophils (NEUT1 and NEUT2), myeloid progenitors (MP), monocytes (MONO), mast cells (MC), and skeletogenic cells (SC) (Fig. 1A). The SC cluster was highly enriched for genes implicated in craniosynostosis, and key osteogenic signaling pathways (Wnt, BMP, TGF-β, and SMAD signaling). Subclustering, RNA velocity, and trajectory analysis refined a high-definition SuSC subset (126 cells), comprising ~1% of Axin2-expressing cells (Fig. 1B-D). Among candidate regulators, LRIG3 emerged as a cell surface molecule selectively expressed in this high-definition SuSC subpopulation. LRIG3 expression was confined to the suture midline in both human and mouse, and co-localized with Axin2+ cells *in vivo* and in suture sphere *ex vivo* (Fig. 2A&B). Functional analysis using Lrig3 knockout mice demonstrated abnormal skull shapes and multiple suture synostoses, with a significant reduction of BMPR1A+ and GLI1+ SuSCs (Fig. 2C&D). Serial culture and kidney capsule transplantation also revealed that SuSC self-renewal and stem cell stemness were affected by the loss of Lrig3 (Fig. 3A-C). Furthermore, SuSC-mediated bone regeneration is severely impaired in LRIG3-deficient mice (Fig. 3D).

**Discussion:** Based on the transcriptomic diversity of Axin2-expressing cells, we have successfully refined the high-definition SuSC cluster using single-cell profiling analysis. With expression in the Axin2+ cells in the suture midline and tight association with BMP signaling (3), LRIG3 stands out among other top candidates for regulating suture morphogenesis. Here we identify LRIG3 as essential for SuSC self-renewal and suture patency; thereby, the findings advance our knowledge base of SuSCs and provide novel insights into the molecular mechanisms governing intramembranous bone formation, offering a foundation for targeted regenerative approaches in craniofacial skeletal disorders.

**Reference:**

1. Ornitz DM, Marie PJ. 2002. Genes & development 16, 1446-1465.
2. Maruyama T, Jeong J, Sheu TJ, Hsu W. 2016. Nature communications 7, 10526.
3. Herdenberg C, et al. 2021. Commun Biol 4, 90.
4. Maruyama T, et al. 2021. Sci Transl Med 13.

Fig. 1

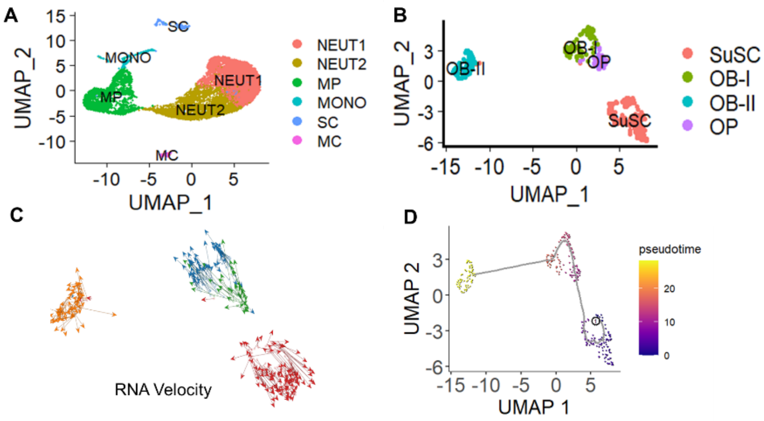

Fig. 2

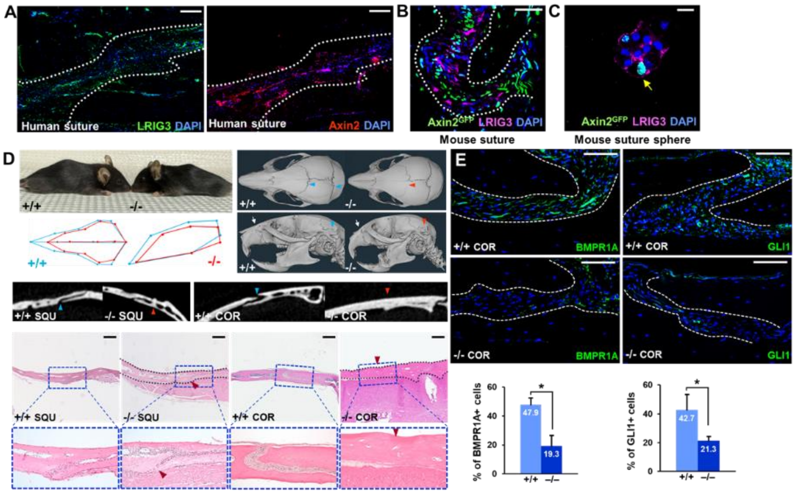

Fig. 3

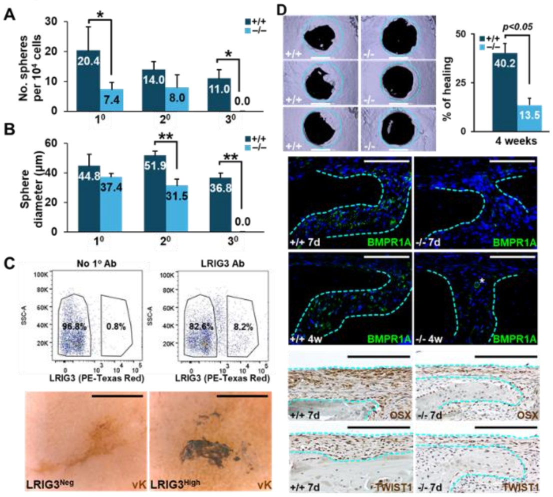

**Fig.1 scRNA-seq analysis predicts a high-definition SuSC population.** (A) The UMAP plot of Axin2-GFP+ cells isolated from P28 Axin2$^{GFP}$ mouse suture mesenchyme. (B) The UMAP plot of SC subsets. (C) RNA Velocity plot from SC subsets. (D) Monocle3 trajectory plot from SC subsets. **Fig. 2 Association of LRIG3 in suture patency and craniosynostosis.** (A) Immunostaining of LRIG3 and AXIN2 examining the 10-month-old human lambdoid suture sections. (B) Immunostaining of LRIG3 reveals co-localized expression of Lrig3 and Axin2 on the sagittal suture section of AxinGFP at P28 and ex vivo Axin2GFP mouse suture sphere (D) microCT (3D and 2D) and H&E staining images of Lrig3-deficient mice at 3wk and 3 months of age. (E) Immunostaining of SuSC markers revealed a significant reduction of positive cells in the coronal (COR) suture deficient for LRIG3 (n =3; * $p$ < 0.01). Double labeling analysis indicating a GFP+ SuSC positive for Lrig3 (yellow arrow). Broken lines define the calvarial bones and highlight the suture mesenchyme. Blue and red arrowheads indicate suture patency and synostosis, respectively. The bottom image shows an enlarged view of the insert. Scale bars, 200 µm (A), 50 µm (B, D, E), and 100 µm (C). **Fig. 3. The loss of LRIG3 affects SuSC self-renewal and stem cell stemness and impairs bone regeneration in mice.** Ex vivo serial culture analysis of suture spheres examining the disruption of LRIG3 on stem cell self-renewal. Graphs represent the average number (A) and size (B) of spheres formed by the culture of WT (+/+) and LRIG3-deficient (-/-) mouse suture cells starting with $10^4$ cells for 1°, 2°, and 3° cultures. (C) Plots showing representative FACS-based isolation of LRIG3$^{Neg}$ and LRIG3$^{High}$ cell populations from P10 mouse suture mesenchyme. Images showing whole mount von Kossa (vK) staining of the kidney after renal capsule transplantation of 4 x $10^3$ indicated cells at 4 weeks. (D) MicroCT of the calvarial region and immunostaining of BMPR1A, OSX, and TWIST examine the suture of the indicated calvarial after injury. Broken circular lines indicate the original damage of 2.0 mm in diameter. Graphs show the average healing percentage in the indicated mice. Broken lines highlight periosteum and suture regions. Brackets and asterisks indicate periosteum thickness and nonspecific staining, respectively. Scale bars, 500 µm (C), 1 mm (D-µCT), 100 µm (D-immunostaining).

### Glutamine metabolism through the hexosamine biosynthesis pathway regulates mesenchymal progenitor cell fate and heterotopic ossification

Ji Hae Choi [1], Yuxiao Sun [1], Monisha Mittal [1], Conan Juan [1], Chase A. Pagani [1], Neda Vishlaghi [1], Alec Bancroft [1], Robert J. Tower [1], Courtney M. Karner [2], Benjamin Levi [*1], Heeseog Kang [*1]
[1] Department of Surgery, University of Texas Southwestern, Dallas, TX; [2] Center for Mineral Metabolism and Clinical Research, Department of Internal Medicine, University of Texas Southwestern, Dallas, TX.

### Introduction & Objectives

Heterotopic ossification (HO) – the pathological formation of bone in soft tissues – occurs when mesenchymal progenitor cells (MPCs) undergo aberrant differentiation following trauma or surgery. Current anti-inflammatory treatments of HO show limited efficacy and significant side effects, underscoring the need for targeted interventions. Given the hypermetabolic state induced by traumatic injury, understanding how cells utilize and process nutrients at the injury site is critical for developing effective therapies. Despite its clinical relevance, the metabolic adaptations driving this process remain incompletely characterized. Here, we identify glutamine metabolism as a critical regulator of MPC fate during heterotopic bone formation.

### Methods

We employed a multi-omics strategy integrating spatial transcriptomics, single-cell RNA-Seq, stable isotope metabolite tracing, metabolite profiling, and genetic and pharmacological interventions to dissect glutamine metabolism pathways in MPCs.

### Results

### Hexosamine biosynthesis pathway (HBP) activation induces metabolic reprogramming in MPCs at the HO site

To assess glutamine metabolism in MPC fate determination, we used a well-established, clinically relevant burn/tenotomy model, where ectopic bone formation occurs at and around the injury site [1]. Spatial transcriptomic analysis 7 days post-B/T revealed strong enrichment of glutamine transport and glutamine metabolism pathways at the HO site compared to surrounding tissues, indicating increased glutamine utilization in the developing HO microenvironment (**Figure 1A**). Glutamine is essential for the hexosamine biosynthesis pathway (HBP), which produces uridine diphosphate N-acetylglucosamine (UDP-GlcNAc) for protein glycosylation. Glutamine-fructose-6-phosphate transaminase (GFPT) enzymes catalyze the rate-limiting step of the HBP, converting glutamine and fructose-6-phosphate to glucosamine-6-phosphate [2]. scRNA-Seq analysis (GSE126060) [3] spanning 0-, 3-, 7-, and 21-days post-B/T injury showed a significant elevation of *Gfpt1* and *Gfpt2* expression in MPCs, suggesting increased HBP activity (**Figure 1B**). Immunofluorescence confirmed higher GFPT1 levels in PDGFRα(+) MPCs at the injury site compared to the uninjured limb (**Figure 1C**).

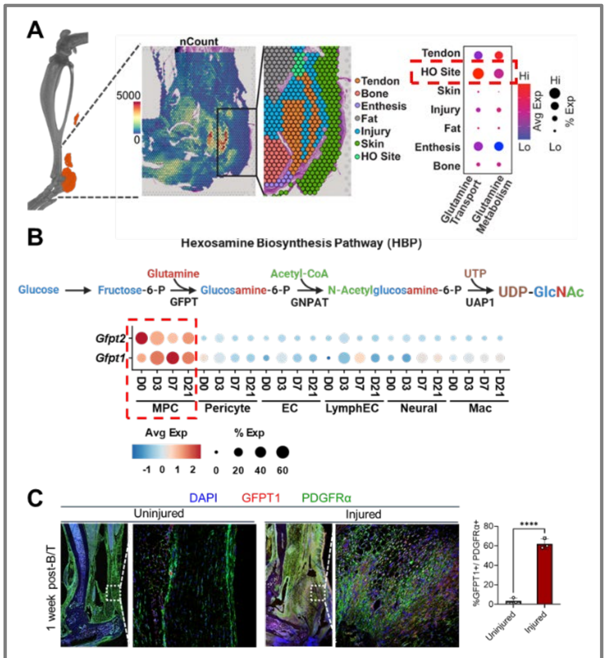

Figure 1. Hexosamine biosynthesis pathway (HBP) activation induces metabolic reprogramming in MPCs at the HO site. (A) 3D-renderd micro-CT scan of a mouse limb highlighting ectopic bone in pseudo-color (orange). Spatial feature plots of H&E-stained histological sections, overlaid with transcript counts (nCount) and a dot plot showing glutamine transport and metabolism pathway enrichment based on module scores across spatial spots within the tenotomy injury site. (B) The HBP. scRNA-seq (B/T-D0_D3_D7_D21) and a dot plot depicting Gfpt1 and Gfpt2 expression across cell clusters at the HO site. (C) Representative images and quantification plot of immunofluorescent staining for GFPT1 protein in PDGFRα(+) MPCs.

### HBP/O-GlcNAcylation enhances MPC osteogenic differentiation

Since HBP generates a crucial substrate for protein O-GlcNAcylation, we assessed the role of OGT—the enzyme mediating GlcNAc transfer onto the free hydroxyl group of select serine or threonine residues in cytosolic, nuclear, and mitochondrial proteins[4] (**Figure 2A**). scRNA-Seq analysis revealed a progressive increase in *Ogt* expression during HO progression (**Figure 2B**). Immunostaining confirmed elevated protein O-GlcNAcylation in PDGFRα(+) MPCs 7 days post-B/T injury (**Figure2C**). In primary MPC cultures, OGT inhibition (OGTi; OSMI-1) significantly suppressed ECM mineralization (**Figure 2D**), whereas OGA inhibition (OGAi; thiamet-G) accelerated mineralization (**Figure 2E**). Pull-down assays using succinylated wheat germ agglutinin (sWGA) beads, which specifically bind O-GlcNAcylated proteins, demonstrated O-GlcNAcylation of RUNX2 in HO

progenitor cells (**Figure 2F**), further supporting the link between HBP activity and MPC osteogenic differentiation. These findings demonstrate that increased HBP/O-GlcNAcylation pathway stimulates osteogenic differentiation of MPCs and that RUNX2 undergoes O-GlcNAcylation in HO progenitor cells.

### Elevated glutamine synthesis fuels metabolic adaptation in MPCs at the HO site

Our spatial transcriptomics revealed enrichment of glutamine biosynthesis pathway at the HO site. (**Figure 3A**). Glutamine synthetase (GS, encoded by *Glul*) is the enzyme responsible for *de novo* glutamine biosynthesis, which condenses ammonia and glutamate into glutamine [5]. Using *Glul*-CreER;tdTom reporter mice [6], in which the *Glul* promoter/enhancer sequences direct the expression of CRE recombinase, thereby recapitulating the endogenous *Glul* expression pattern marked by tdTomato, we confirmed increased GS expression in PDGFRα(+) MPCs in injured versus uninjured limbs (**Figure 3B**). These findings indicate that *de novo* glutamine synthesis increases in MPCs during HO progression, supporting metabolic adaptations that drive pathological bone formation.

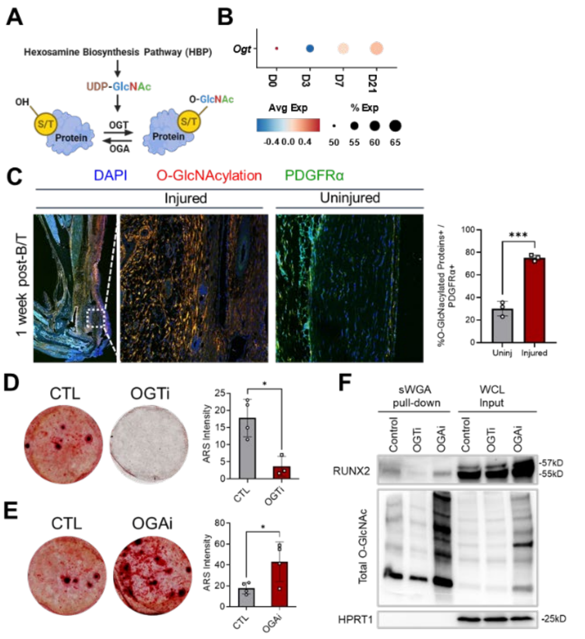

**Figure 2. HBP/O-GlcNAcylation enhances MPC osteogenic differentiation.** **(A)** Schematic diagram illustrating the Hexosamine Biosynthesis Pathway (HBP) and O-GlcNAcylation process. **(B)** Dot plot illustrating *Ogt* expression levels in MPCs at different time points. **(C)** Representative images and quantification plot of immunofluorescence staining for protein O-GlcNAcylation in PDGFRα(+) MPCs in tissue sections. **(D-E)** Representative images and quantification plot of ECM mineralization in MPCs cultures subjected to osteogenic differentiation with (H) OGT inhibitor (OGTi; OSMI-1) or vehicle (DMSO), and (I) OGA inhibitor (OGAi; thiamet-G) or vehicle (DMSO), followed by Alizarin Red staining. **(F)** RUNX2 O-GlcNAcylation assay using sWGA pull-down.

### Glutamine synthesis connects HBP flux and MPC osteogenic differentiation

To assess GS-mediated glutamine flux into HBP, we knocked down *Glul* in MPCs isolated from the tenotomy site and performed $^{15}N$-ammonia ($^{15}N$-$NH_4^+$) stable isotope tracing (**Figure 4A**). *Glul*-deficient cells showed significantly reduced UDP-GlcNAc labeling, indicating decreased HBP activity (**Figure 4B**). Pharmacological GS inhibition using methionine sulfoximine (MSO) suppressed alkaline phosphatase activity in cultured MPCs (**Figure 4C**). Consistent with this observation, i*n vivo* inhibition of the HBP with azaserine, a glutamine antagonist that inhibits GFPT, significantly reduced HO formation in B/T-injured mice (**Figure 4D**), highlighting the therapeutic potential of metabolic interventions.

### Targeting GLS-mediated glutaminolysis reduced HO formation

Given the role of glutamine in fueling the tricarboxylic acid (TCA) cycle and the HBP/OGT/O-GlcNAcylation axis in HO, we investigated glutaminolysis. Glutaminolysis is a metabolic pathway where glutamine is converted to α-ketoglutarate (αKG) via sequential actions of glutaminases (GLS) and glutamate dehydrogenases (GDH), providing essential TCA cycle intermediates for cells with high metabolic demand[7] (**Figure 5A**), such as MPCs at sites of injury. MPC-specific GLS knockout mice (*Hoxa11*-CreER$^{T2}$(+);*Gls*$^{fl/fl}$) developed significantly less HO following B/T injury compared to controls (*Hoxa11*-CreER$^{T2}$(-);*Gls*$^{fl/fl}$). This establishes glutaminolysis as a key metabolic driver of pathological ossification.

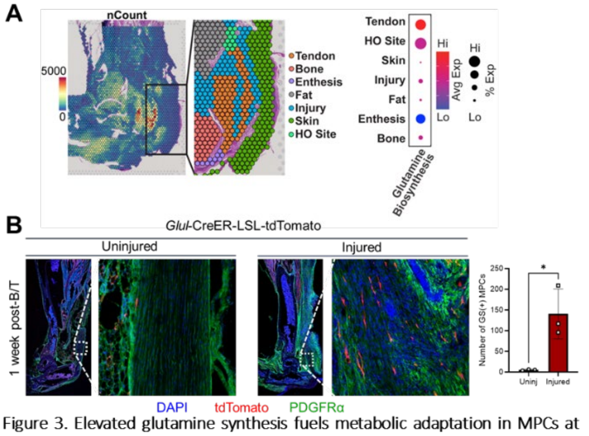

**Figure 3. Elevated glutamine synthesis fuels metabolic adaptation in MPCs at the HO site.** **(A)** Spatial feature plots of transcript counts (nCount) alongside a dot plot illustrating glutamine biosynthesis pathway enrichment based on module scores across spatial spots within the tenotomy injury site. **(B)** Representative images and quantification plot of immunofluorescence staining for PDGFRα(+) MPCs in tissue sections of Glul-CreER;tdTomato reporter mice 7 days post-B/T.

### The HBP/OGT axis modulates GLS expression via YAP activation

Given that GLS deficiency reduced HO formation, we hypothesized that the HBP/OGT axis is mechanistically connected with GLS-mediated glutaminolysis. To investigate the molecular mechanism, we took primary MPCs from tenotomy injury site and transfected them with *Ogt* siRNA to knockdown *Ogt* expression. Interestingly, *Ogt* knockdown dramatically reduced GLS protein levels (**Figure 6A**), suggesting a mechanistic link between O-GlcNAcylation and glutaminolysis. Given the pivotal role of YAP in HO by driving osteogenic differentiation through mechanotransduction[8] and metabolic regulation by influencing glutamine metabolism[9], we further examined if the HBP/OGT axis regulates YAP activity to modulate GLS-mediated glutaminolysis. YAP knockdown significantly decreased GLS expression (**Figure 6B**), reinforcing the role of YAP in integrating metabolic regulation and mechanotransduction during HO progression. These results suggest that YAP signaling integrates with GLS-mediated glutaminolysis and O-GlcNAcylation to regulate MPC fate.

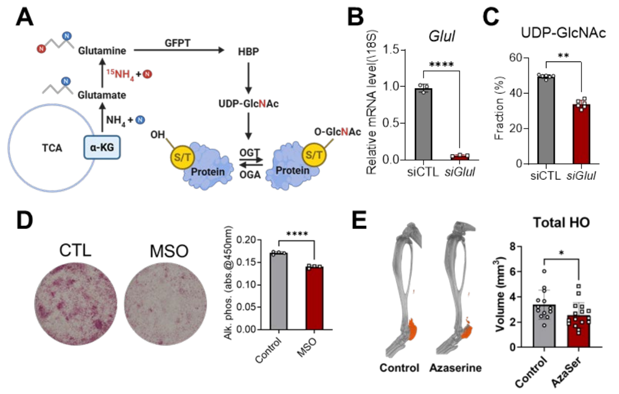

Figure 4. Glutamine synthesis connects HBP flux and MPC osteogenic differentiation. **(A)** Schematic illustration of $^{15}NH_3$-originated $^{15}$N-glutamine incorporation into HBP and UDP-GlcNAc. **(B)** Real-time qPCR analysis of *Glul* transcript levels in MPCs following *Glul* knockdown via siRNA transfection. **(C)** Fractional isotopomer labelling of UDP-GlcNAc from $^{15}NH_3$ ($^{15}$N-labelled UDP-GlcNAc), measured by LC-MS. **(D)** Representative images and quantification plot of alkaline phosphatase (ALP) activity in MPC cultures subjected to osteogenic differentiation with GS inhibitor (MSO) or vehicle (DMSO) followed by ALP staining. **(E)** Representative images and quantification of total HO via µCT of BT injured limbs. Eight-week-old male C57bL/6J wild-type mice were treated with azaserine for 3 weeks post-B/T.

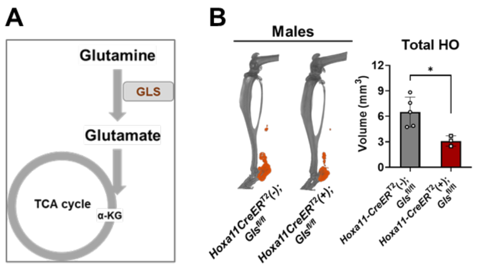

Figure 5. Targeting GLS-mediated glutaminolysis reduced HO formation. **(A)** Illustration of GLS-mediated glutaminolysis pathway. **(B)** Representative images and quantification of total HO via µCT of B/T injured limbs from *Hoxa11*-CreER$^{T2}$(–);*Gls*$^{fl/fl}$ and *Hoxa11*-CreER$^{T2}$(+);*Gls*$^{fl/fl}$ mice 9-weeks post-B/T.

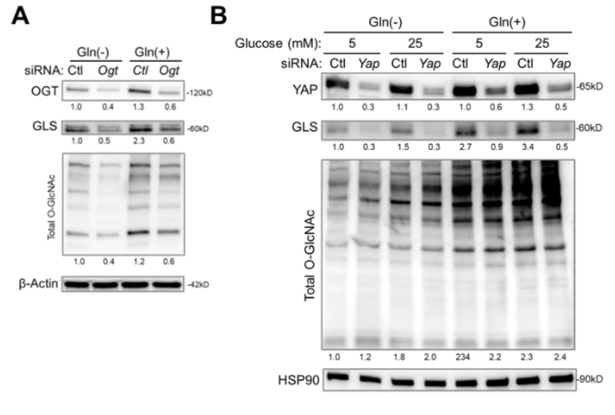

Figure 6. The HBP/OGT axis regulates GLS expression via YAP activation.
**(A)** Western blot analysis for OGT, GLS, and total protein O-GlcNAcylation in MPCs cultured with or without L-glutamine following Ogt siRNA knockdown. β-Actin served as a loading control. Band intensity was quantified using ImageJ and annotated beneath each blot. **(B)** Western blot analysis for YAP, GLS, and total protein O-GlcNAcylation in MPCs cultured with or without L-glutamine following Ogt siRNA knockdown. HSP90 served as a loading control.

Graphical Abstract

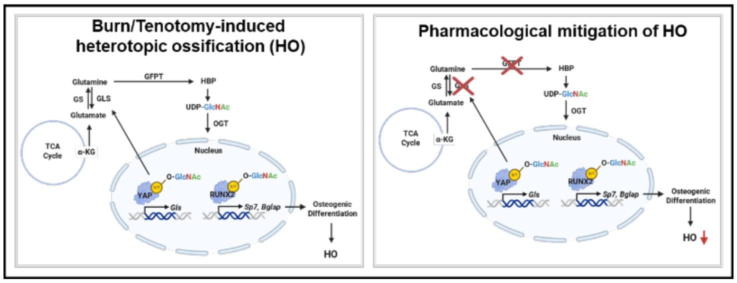

### Conclusion

Traumatic HO is associated with increased glutamine metabolism, which elevates UDP-GlcNAc levels via HBP. This facilitates RUNX2 O-GlcNAcylation and subsequent osteogenic differentiation, thereby controlling MPC fate in HO. Our findings establish glutamine metabolism as a key regulator of HO progression and highlight the HBP and GLS-mediated glutaminolysis as promising therapeutic targets for mitigating pathological bone formation.

### References

1 Peterson, J. R. *et al.* Direct Mouse Trauma/Burn Model of Heterotopic Ossification. *J Vis Exp*, e52880 (2015). https://doi.org/10.3791/52880

2 Yamazaki, K. in *Handbook of Glycosyltransferases and Related Genes* (eds Naoyuki Taniguchi *et al.*) 1465-1479 (Springer Japan, 2014).

3 Sorkin, M. *et al.* Regulation of heterotopic ossification by monocytes in a mouse model of aberrant wound healing. *Nat Commun* **11**, 722 (2020). https://doi.org/10.1038/s41467-019-14172-4

4 Stephen, H. M., Adams, T. M. & Wells, L. Regulating the Regulators: Mechanisms of Substrate Selection of the O-GlcNAc Cycling Enzymes OGT and OGA. *Glycobiology* **31**, 724-733 (2021). https://doi.org/10.1093/glycob/cwab005

5 Listrom, C. D. *et al.* Expression, purification, and characterization of recombinant human glutamine synthetase. *Biochem J* **328 ( Pt 1)**, 159-163 (1997). https://doi.org/10.1042/bj3280159

6 Wei, Y. *et al.* Liver homeostasis is maintained by midlobular zone 2 hepatocytes. *Science* **371** (2021). https://doi.org/10.1126/science.abb1625

7 Yang, L., Venneti, S. & Nagrath, D. Glutaminolysis: A Hallmark of Cancer Metabolism. *Annu Rev Biomed Eng* **19**, 163-194 (2017). https://doi.org/10.1146/annurev-bioeng-071516-044546

8 Cong, Q. *et al.* A self-amplifying loop of YAP and SHH drives formation and expansion of heterotopic ossification. *Sci Transl Med* **13** (2021). https://doi.org/10.1126/scitranslmed.abb2233

9 Cox, A. G. *et al.* Yap reprograms glutamine metabolism to increase nucleotide biosynthesis and enable liver growth. *Nat Cell Biol* **18**, 886-896 (2016). https://doi.org/10.1038/ncb3389

### Aging and the mechano-sensitivity of bone healing: Developing an experimental approach to investigate age-dependent mechanobiological responses in bone.

*Neashan Mathavan[1], Graeme Paul[1], Sara Lindenmann[1], Stefanie Wissmann[1], Francisco Correia Marques[1], Dilara Yilmaz[1], Gisela A Kuhn[1], Esther Wehrle[1,2]*, Ralph Müller[1]**

*[1]Institute for Biomechanics, ETH Zürich, Zürich, Switzerland, [2]AO Research Institute Davos, Davos Platz, Switzerland.*
** equal contribution*

**Introduction:** Aging is associated with the dysregulation of mechanobiological mechanisms that impairs the ability of cells to sense and adapt to extracellular mechanical stimuli (*1*). The mechanical environment is of critical importance to the regenerative response in bone. However, no consensus exists on the effect of aging on the mechano-sensitivity of bone regeneration. Certain characteristics of bone aging have been identified with the potential to influence bone mechano-sensitivity and its subsequent regenerative capacity. These include: decreased osteocyte density, reducing the primary mechano-sensing cell population; deterioration of the lacuno-canalicular network (LCN), disrupting mechanotransduction pathways; and declining osteogenic cell populations coupled with compromised angiogenesis, limiting the tissue's ability to respond appropriately to mechanical cues (*2*). Given the higher incidence of recalcitrant fractures in elderly populations, insights into the mechanisms by which aging alters bone mechano-sensitivity may provide much-needed novel therapeutic targets to enhance bone regeneration in these patients.

Investigating the influence of aging on the mechano-sensitivity of bone healing presents multifaceted challenges. Although rodent models are well-established within the field, the use of naturally aged animals requires significantly longer investigation timelines, and maintaining such colonies is resource intensive. Furthermore, in vivo mechanical loading applied at the organ scale is heterogeneously distributed throughout fracture sites, resulting in complex mechanical environments with distinct regions of high and low strain. Consequently, each cell within a fracture site resides within different local mechanical microenvironments. This heterogeneity in local mechanical environments renders measuring cellular responses to mechanical stimuli within bone tissue technically challenging.

To address these challenges, our aim is to develop an experimental approach to investigate age-dependent mechanobiological responses in bone healing. Our approach consists of combining: (i) an established femur defect mouse model (*3*), (ii) the use of $PolgA^{D257A/D257A}$ (PolgA) mice - a mouse model of premature aging (*4*), (iii) an osteogenic cyclic loading protocol (*5*), and (iv) our recently established spatial transcriptomics–based "mechanomics" platform (*6*).

**Methods:** 12-week-old or "Young" female PolgA mice (n=15) and 35-week-old or "Aged" female PolgA mice (n=14) received a femoral fracture (Young: 0.57±0.1 mm, Aged: 0.43±0.1 mm) stabilized with an external fixator (license: ZH229/19, ZH033/2022 Kantonales Veterinäramt Zürich, Switzerland). Time-lapsed in vivo micro-CT imaging was performed weekly at the fracture site (weeks 0-7; 10.5µm resolution). Following bridging of the fracture site at 3 weeks after surgery, mice were subdivided into Loaded or Control groups and received either cyclic mechanical loading (2-16N, 10Hz, 3000cycles; 3 times per week) or sham-loading. Mechanical loading applied at each time point is based on the computation of the strain distribution within the fracture site of each mouse and the scaling of the strain distribution to achieve a predefined median target strain. Statistical significance ($p < 0.05$) between groups at each weekly time-point was assessed using two-way ANOVA with Geisser-Greenhouse and Bonferroni corrections.

**Results:** The regenerative response at fracture sites varied both spatially and temporally between Young and Aged female PolgA mice. In our assessment of time-lapsed in vivo CT imaging data, BV/TV in the defect center (DC) was significantly higher at weeks 2-3 in Young mice (Fig 1. [A]; $p < 0.01$). Similarly in peripheral regions, BV/TV was significantly higher at week 3 in the defect periphery (DP) (Fig 1. [B]; $p < 0.05$) and week 2 in the cortex periphery (FP) (Fig 1. [C]; $p < 0.05$). In Aged mice, BV/TV values comparable to Young mice were achieved in both DC and DP regions from week 4 onwards suggestive of a delayed response with aging (Fig 1. [A][B]). In contrast, BV/TV values remained significantly higher in the FP region of Young mice from week 4 onwards reflecting a diminished osteogenic response in peripheral regions (Fig 1. [C]; $p < 0.05$). Moreover, Young mice exhibited significantly higher bone formation rates (BFR) across all three volumes of interest at early timepoints (Fig 1. [D][E][F]; $p < 0.001$, $p < 0.05$).

In response to mechanical loading, both Young and Aged mice exhibited a clear trend of increasing BV/TV in the defect center (DC) across all timepoints at which loading was applied (Fig 2. [G][J]). Notably, these differences were only statistically significant in the Aged mice (Fig 2. [G][J]; $p < 0.001$, $p < 0.01$). In contrast, only Young mice exhibited significantly greater BV/TV in peripheral regions in response to loading (Fig 2. [H][I]; $p < 0.01$, $p < 0.05$).

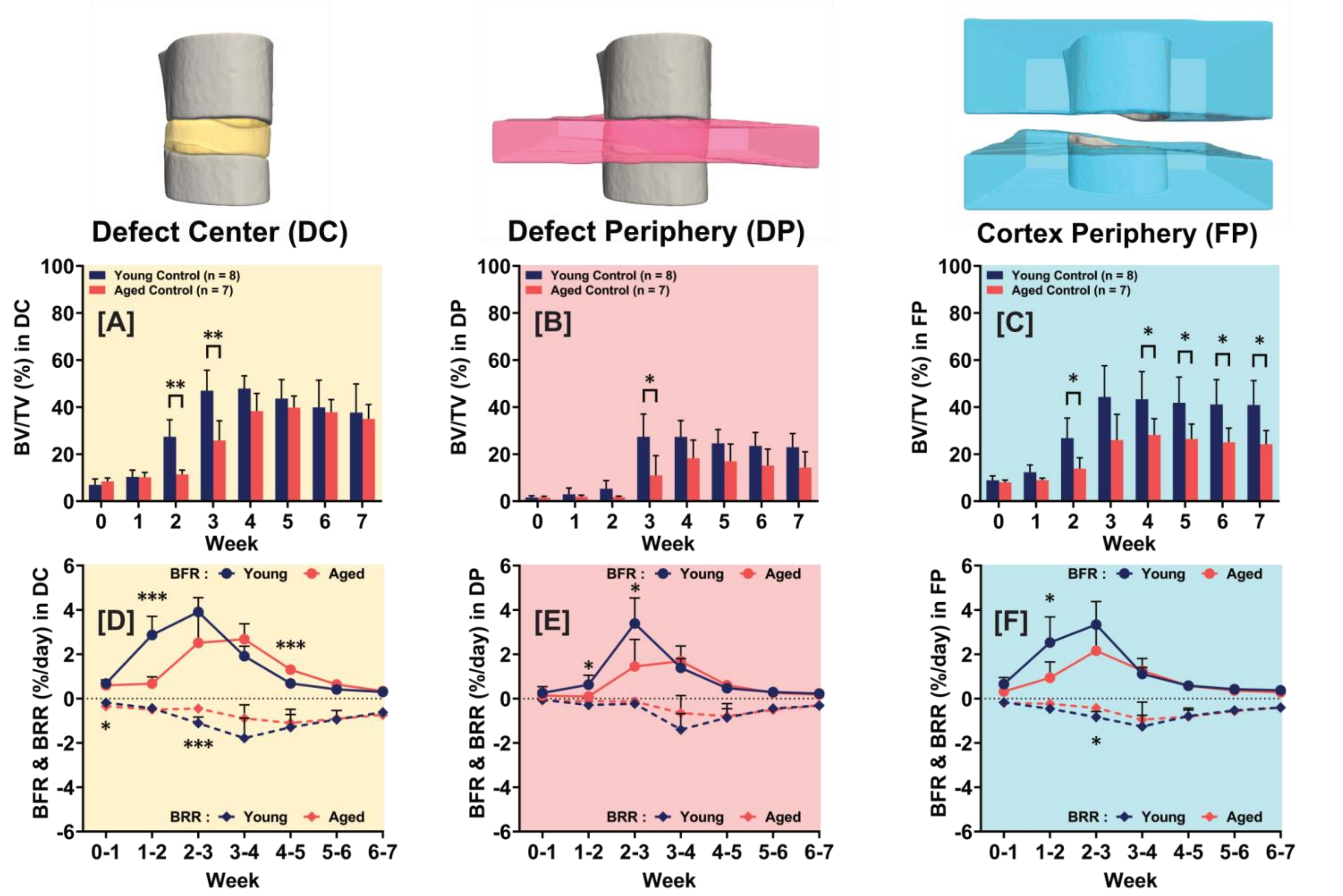

Figure 1. Regenerative response in Young vs. Aged mice. Quantitative morphometric analyses were performed in three volumes of interest: the defect center (DC), the defect periphery (DP), and the cortex periphery (FP). Two parameters are presented: [A][B][C] BV/TV and [D][E][F] bone remodelling rates: bone formation rate (BFR) and bone resorption rate (BRR).

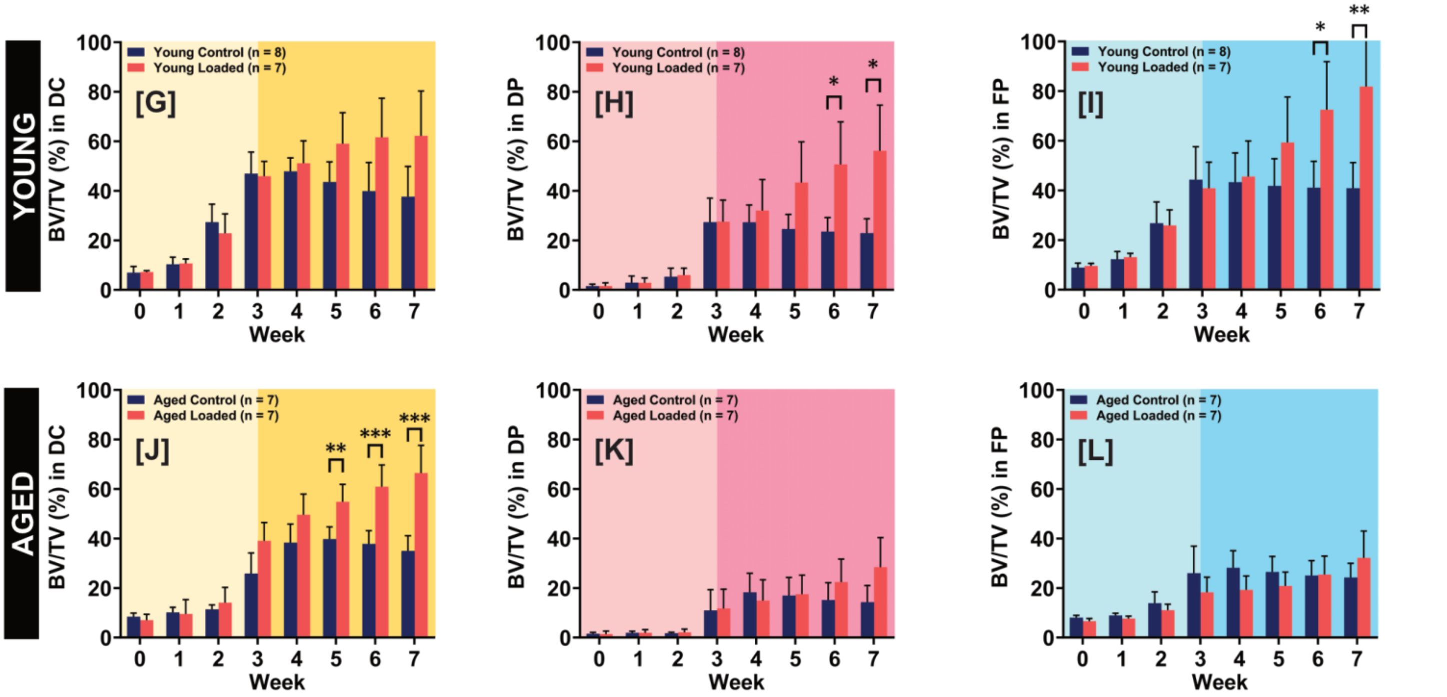

Figure 2. Regenerative response in Control vs. Loaded mice. BV/TV is presented in [G][H][I] Young and [J][K][L] Aged mice in the DC, DP and FP volumes of interest. Shaded regions correspond to time points at which loading was applied 3× per week.

**Discussion:** Using an established femur-defect mouse model, we demonstrate that cyclic loading significantly enhances regeneration in young mice, and this mechano-responsiveness is remarkably retained with aging. In our previous characterizations of the musculoskeletal phenotype of this mouse strain, we found PolgA mice to exhibit frailty, osteosarcopenia and deterioration of the osteocyte lacuno-canalicular network with age (*7*). Moreover, aging in these mice was also associated with impaired osteogenesis, increased osteoclast activity and declines in bone mass (*8*). Collectively, these findings underscore PolgA mice as a relevant preclinical model in investigations of age-dependent mechanobiological responses in bone. Our findings thus suggest that mechanical stimulation therapies have the potential to be promising interventions in the treatment of challenging fractures in elderly patients.

As described previously, the heterogeneity in local mechanical environments at fracture sites renders measuring cellular responses to mechanical stimuli within bone tissue technically challenging. Furthermore, in our morphometric analyses, we demonstrate that PolgA mice exhibit spatially and temporally varying regenerative responses with age. In future studies, we therefore plan to use our recently established "spatial mechanomics" approach (Fig 3.) which permits analysis of spatially-resolved gene expression as a function of the local mechanical environment. In doing so, we hope to gain insights into age-dependent mechanobiological responses in bone with the potential to discover mechanoresponsive targets and identify strategies to enhance repair in compromised healing environments.

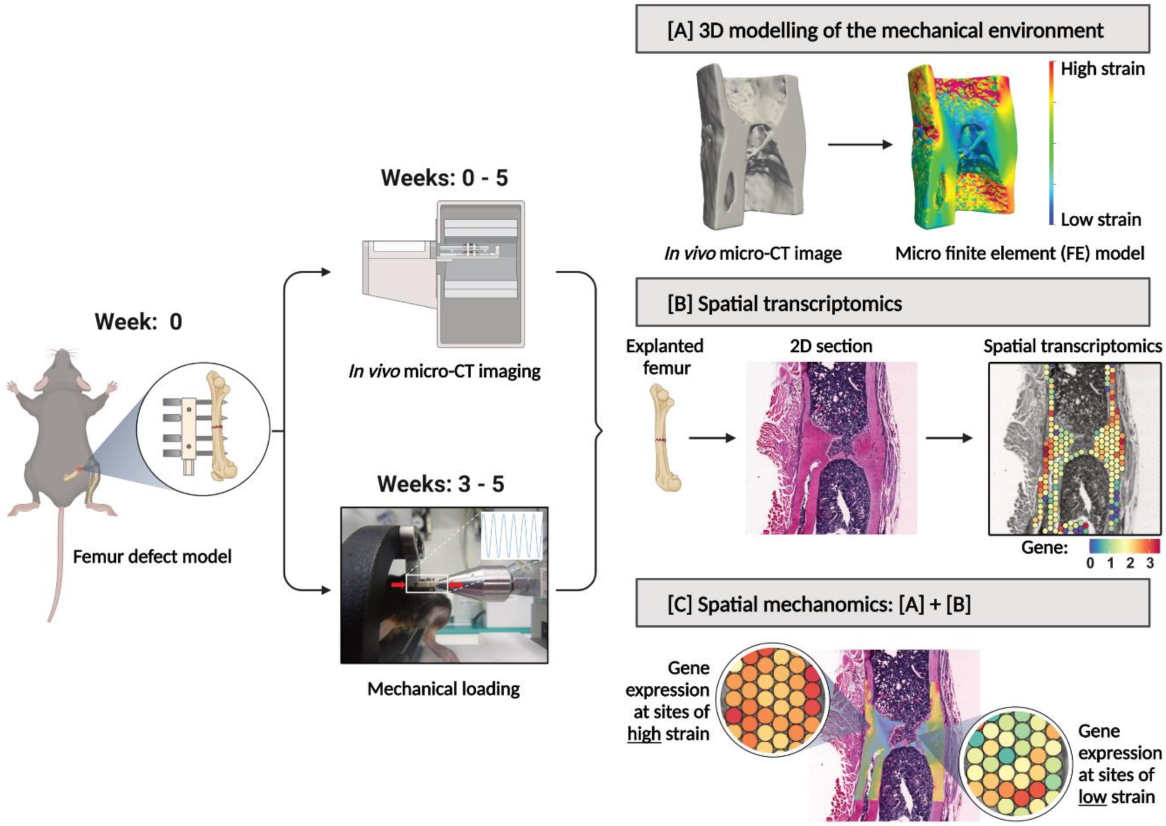

**Figure 3. Overview of our "spatial mechanomics" approach.** Our approach permits the generation and spatial integration of multimodal datasets (CT bone morphology data, 3D mechanical environments, and spatially resolved gene expression data) from a single fracture site (*6*).

### References:

[1] Bajpai A. et al. *Ann. N. Y. Acad. Sci.* (2021)
[2] Javaheri A. et al. *Curr. Osteoporos. Rep.* (2019)
[3] Wehrle E. et al. *Sci. Rep.* (2021)
[4] Yilmaz D. et al. *Ageing Res. Rev.* (2024)
[5] Paul G. R. et al. *Sci. Rep.* (2021)
[6] Mathavan N. et al. *Sci. Adv.* (2025)
[7] Yilmaz D. et al. *Bone Res.* (2025)
[8] Dobson P. F. et al. *Sci. Rep.* (2020)

**Insights into Intestinal Pathophysiology and Microbial Ecology in Osteogensis imperfecta**

*Yuan Zhang* [1,2], *Michèle Iskandar* [1], *Stan Kubow* [1], *Svetlana V. Komarova* [2,3,4], ***Josephine T. Tauer*** [2,3,5]

*1) School of Human Nutrition, McGill University, Montreal, Canada; 2) Shriners Hospitals for Children, Montreal, Canada; 3) Faculty of Dental Medicine and Oral Health Sciences, McGill University, Montreal, Canada; 4) Biomedical Engineering, University of Alberta, Edmonton, Canada; 5) Centre for Growth and Osteology, Department of Pediatrics and Adolescent Medicine, Johannes Kepler University Linz, Linz, Austria*

**Project funding:** Shriners of North America
**Publication:** As of May 13, 2025, the results of this project are currently under review at JBMR.
**Author Contributions:** *Zhang*: Methodology, Investigation, Formal Analysis, Data Curation, Validation; *Iskandar*: Methodology; *Kubow*: Supervision; *Komarova*: Conceptualization, Funding acquisition, Supervision; *Tauer*: Conceptualization, Funding acquisition, Project administration, Methodology, Formal Analysis, Writing.

**Introduction:** Osteogenesis imperfecta (OI) is a genetic disorder of the connective tissue, most commonly caused by mutations in collagen type I — a fundamental structural protein in the extracellular matrix (ECM). Although OI is best known for its impact on the skeleton, collagen-I is also vital for the integrity of the intestinal ECM, which supports digestive function, nutrient absorption, and overall energy balance (Graham et al. 1988). Recent studies suggest that OI involve more than just bone fragility: both young patients and juvenile OI mouse models exhibit signs of hypermetabolism, including elevated energy expenditure, increased body temperature, and reduced fat mass (Cropp & Myers 1972; Boraschi-Diaz et al. 2017; Gremminger et al. 2021). These features hint at higher nutritional demands during early development. Interestingly, this hypermetabolic state appears to diminish with age and is often replaced by an increased body fat-to-muscle ratio in adulthood (Chagas et al. 2012).

Adding to this complexity, a recent large-scale cohort study revealed that individuals with OI face a heightened risk of gastrointestinal (GI) disorders, such as peptic ulcers, inflammatory bowel disease, and chronic constipation. These complications not only affect quality of life but also contribute significantly to increased mortality in aging OI patients (Folkestad et al. 2016; Anderesen et al. 2025). Intriguingly, dietary studies in OI mouse models have shown that while high-fat diet-induced obesity can be mitigated, glucose intolerance persists, underscoring a complex interaction between OI and metabolic regulation.

Despite the central role of the intestine in systemic metabolism — and the fact that collagen-I is the predominant collagen in intestinal tissue (Gremminger et al. 2021) — the gut has remained an underexplored territory in OI research. Given the frequency and severity of GI issues in OI (Lee et al. 1995; Anderesen et al. 2025), a deeper understanding of intestinal alterations in this disease is urgently needed.

**Objective:** This study investigated the structure and function of the gastrointestinal tract (GIT), as well as microbial composition in male and female OI mouse models. Specifically, we examined Col1a1Jrt/+ mice carrying a COL1A1 mutation and oim-/- mice with a COL1A2 mutation, both maintained on a standard chow diet, to uncover potential intestinal contributions to the broader OI phenotype.

**Results:** Both OI mouse models exhibited age-independent GIT fragility and increased relative intestinal length, especially in females (**Fig. 1A**, **Fig. 4A**).

In Col1a1Jrt/+ mice, structural adaptations in the small intestine were observed, including an increased villus-to-crypt ratio and greater surface area throughout the small intestine (**Fig. 1B**). Total collagen content was significantly reduced, particularly in the duodenum of males (**Fig. 2A**), with corresponding downregulation of Col1a1 and Col3a1 gene expression (**Fig. 2B**). Despite reduced intestinal permeability in males, outliers with high plasma dextran levels (likely due to tissue rupture) support the observed GIT fragility (*data not shown*). Microbial profiling at 4 weeks of age showed similar overall diversity across genotypes (**Fig. 3A**, left), but Col1a1Jrt/+ mice exhibited significantly reduced abundance of saccharide-fermenting genera (Lactobacillus, Desulfovibrio, Lachnospiraceae_FCS020; **Fig. 3A**, right). These microbial shifts were accompanied by reduced cecal short-chain fatty acid (SCFA) production, especially in females (**Fig. 3B**), with specific reductions in most SCFAs and branched-chain fatty acids (**Fig. 3C**), suggesting impaired microbial fermentation.

In oim-/- mice, similar increases in intestinal length and fragility were observed (**Fig. 4A**). ECM gene expression revealed reductions in Col1a1, Col2a1, Col3a1, and elastin (**Fig. 4B**). Intestinal permeability was increased, particularly in males, and again associated with evidence of tissue breakage (*data not shown*). Microbial diversity in oim-/- mice remained broadly comparable to WT (**Fig. 5**, left), but discriminant analysis revealed increased Lactobacillus (in males), increased Leuconostoc, and reduced Anaerosporobacter (a butyrate producer) (**Fig. 5**, right), all of which influence gut pH, inflammation, and nutrient absorption.

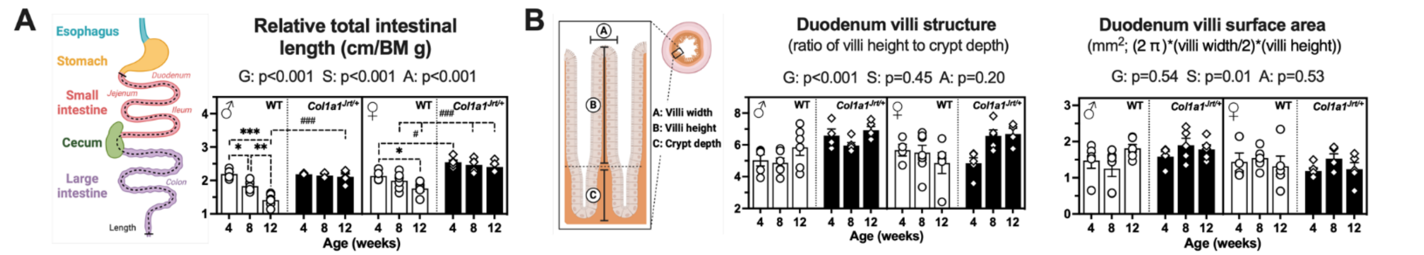

**Figure 1: Gastrointestinal phenotype in WT (o, white bar) and Col1a1Jrt/+ (◇, black bar) mice.** Data represent mean±SEM. Statistics: 3way-ANOVA with Bonferroni post-hoc-test, G, overall genotype-effect; overall S, sex-effect; A, overall age-effect; * age-effect; # genotype-effect; *,#, p<0.05; **,##, p<0.01, ***,###, p<0.001.

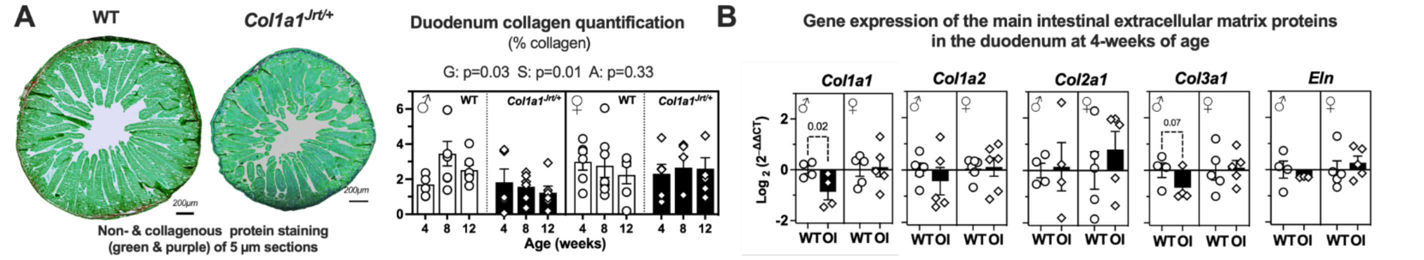

**Figure 2: Duodenal collagen content (A) and extracellular matrix protein gene expression (B) in WT (○, white bar) and Col1a1Jrt/+ (◇, black bar) mice.** Data represent mean±SEM. Statistics: (A) 3way-ANOVA with Bonferroni post-hoc-test, G, genotype-effect; S, sex-effect; A, age-effect. (B) Unpaired one-tailed T-Test to assess genotype effect per sex.

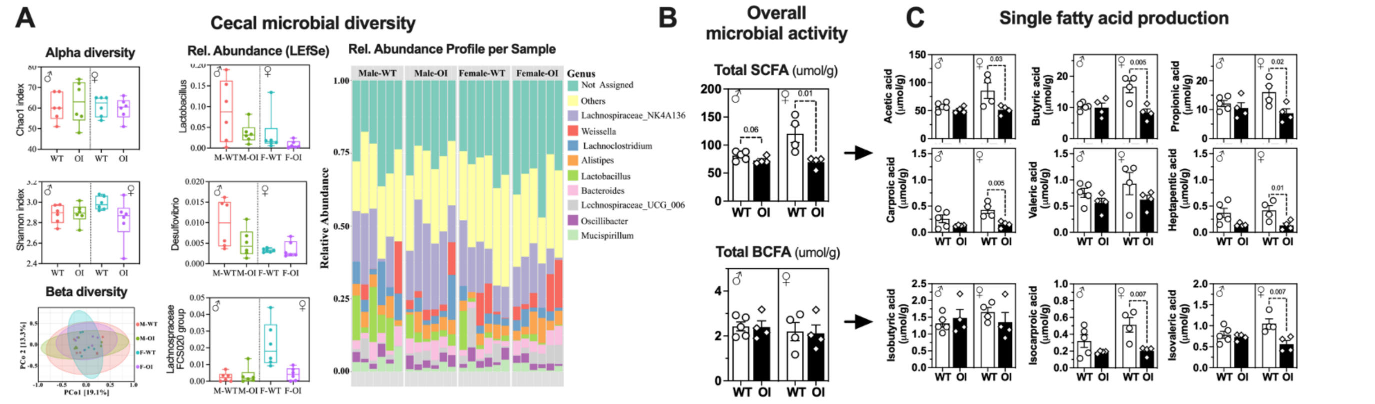

**Figure 3: Microbial diversity (A), overall microbial activity (B) and detailed fatty acid production (C) in cecal samples of 4-week-old WT (○, white bar) and Col1a1Jrt/+ (◇, black bar) mice.** Cecal samples derived from 4-week-old offspring mice born to wild-type females and OI males. Data represent mean±SEM. Statistics: (A) 16S data analysis was performed utilizing MicrobiomeAnalyst (Chong et al. 2020; Sun et al. 2023). Data were filtered by default, followed by good's coverage check (>99.98%) and rarefication to the minimum library size (n = 127,464). (B,C) Unpaired one-tailed T-Test to assess genotype effect per sex.

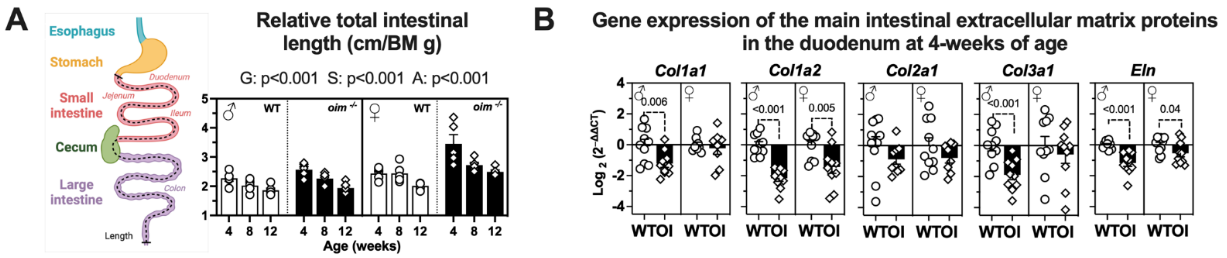

**Figure 4: Gastrointestinal phenotype in WT (o, white bar) and *oim* $^{-/-}$ (◇, black bar) mice.** Data represent mean±SEM. Statistics: (A) 3way-ANOVA with Bonferroni post-hoc-test, G, overall genotype-effect; overall S, sex-effect; A, overall age-effect. (B) Unpaired one-tailed T-Test to assess genotype effect per sex.

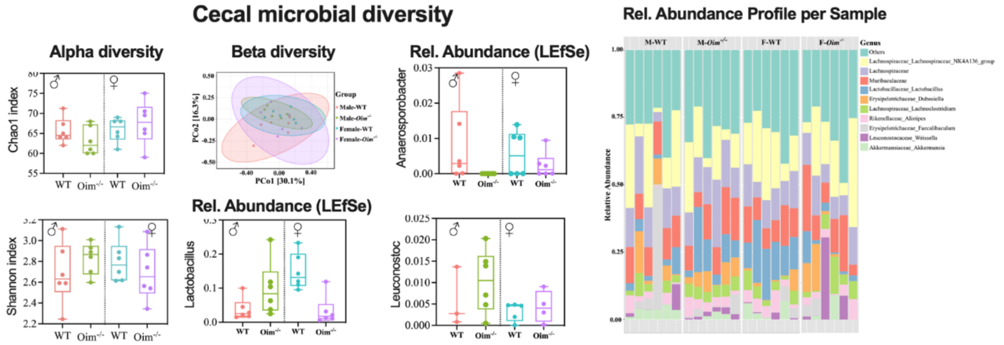

**Figure 5: Microbial diversity in cecal samples of 4-week-old WT (○, white bar) and *oim* $^{-/-}$ (◇, black bar) mice.** Cecal samples derived from 4-week-old offspring mice born to heterozygous females and males (*oim* $^{-/+}$ mice). Data represent mean±SEM. Statistics: (A) 16S data analysis was performed utilizing Microbiome-Analyst (Lu Y et al., Nucleic Acids Res. 2023;51(W1); Chong J et al., Nat Protoc. 2020;15(3)) Data were filtered by default, followed by good's coverage check (>99.98%) and rarefication to the minimum library size (n = 127,464).

**Conclusions:** This study reveals that collagen-I mutations in OI not only affect skeletal tissue but also lead to profound structural and functional changes in the GI system. While Col1a1Jrt/+ and oim-/- mice share some intestinal and microbial adaptations, distinct differences in gut permeability and extracellular matrix composition point to mutation-specific responses. Despite these challenges, evidence of intestinal structural adaptation suggests a compensatory response that helps maintain basic digestive function. However, altered microbial profiles and reduced microbial fermentation activity—particularly in females—may limit energy extraction from the diet and contribute to the metabolic phenotype observed in OI.

Importantly, these findings provide a potential biological explanation for the high prevalence of GI symptoms, nutritional challenges, and altered body composition seen in OI patients. Understanding the gut as a secondary but relevant site of disease opens new avenues for supportive therapies in OI, such as microbiota-targeted interventions, dietary modifications, or strategies to enhance intestinal barrier and absorptive function. This holistic view may ultimately improve quality of life and long-term health outcomes in individuals living with OI.

**References**

Anderesen CK, Al-Najami I, Liu W et al. (2025) Calcif Tissue Int 116**,** 15.
Boraschi-Diaz I, Tauer JT, El-Rifai O et al. (2017) J Endocrinol 234**,** 279-289.
Chagas CEA, Roque JP, Santarosa Emo Peters B et al. (2012) Nutrition 28**,** 138-142.
Chong J, Liu P, Zhou G et al. (2020) Nat Protoc 15**,** 799-821.
Cropp GJ, Myers DN (1972) Pediatrics 49**,** 375-391.
Folkestad L, Hald JD, Canudas-Romo V et al. (2016) J Bone Miner Res 31**,** 2159-2166.
Graham MF, Diegelmann RF, Elson CO et al. (1988) Gastroenterology 94**,** 257-265.
Gremminger VL, Harrelson EN, Crawford TK et al. (2021) Mol Genet Metab 132**,** 244-253.
Lee JH, Gamble JG, Moore RE et al. (1995) J Bone Joint Surg Am 77**,** 1352-1356.
Sun J, Lu F, Luo Y et al. (2023) Nucleic Acids Res 51**,** W397-W403.

<u>RESULTS:</u> *Ischemic calluses exhibit delayed chondrogenesis and increased fibroblastic stromal progenitor differentiation:* At 7 days post fracture (dpf), during early stromal expansion post-fracture, we observed a pronounced decrease in cartilage in the ischemic calluses compared to control callus (Fig 1A). However, consistent with Lu et al.[5], there was no difference in callus size, emphasizing the fibrotic tissue that comprises the ischemic fracture callus. To assess whether ischemia negatively altered stromal progenitor differentiation leading to increased fibrosis, we performed scRNAseq-based cellular trajectory analysis and found massive perturbations in the differentiation pathway from aSMA+ progenitors towards hypertrophic chondrocytes (Fig 1B). Analysis of gene expression as cells undergo differentiation revealed delayed and diminished pseudotime-dependent expression with Ischemia (Fig 1C). Targeted analysis of DEGs upregulated in Ischemic calluses at 7 dpf revealed overexpression of fibrosis-relevant genes including *Col3a1*[8], *Col5a1, and Mmp14*[9] (Fig 1D).

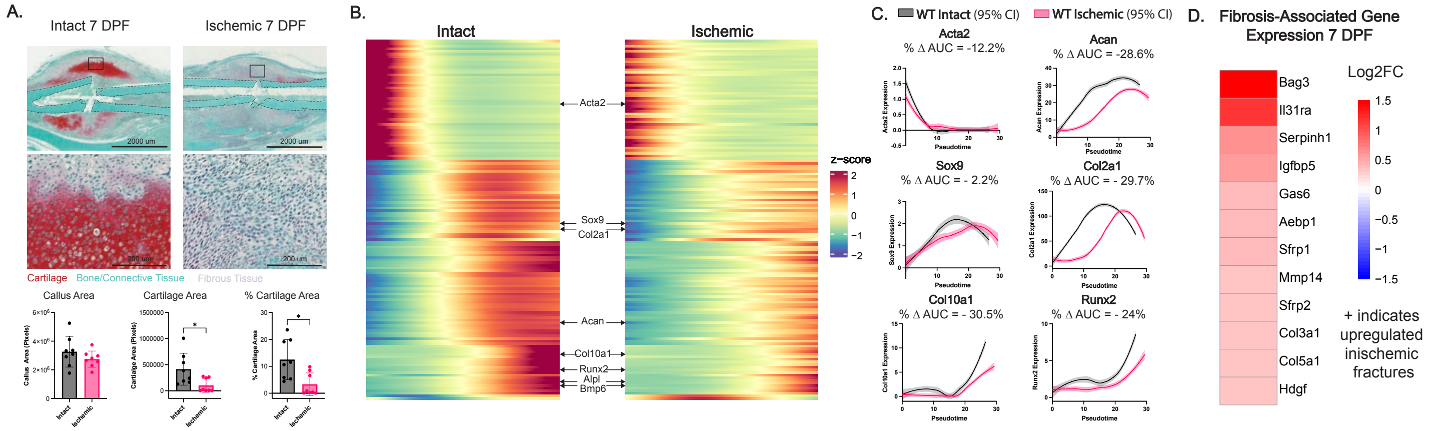

***Figure 1:*** *Ischemic calluses exhibit delayed chondrogenesis and increased fibroblastic stromal progenitor differentiation. (A) Representative Safranin-O histology of Intact and Ischemic fractured tibias at 7 days post fracture (dpf) (cartilage is red, bone/connective tissue is blue/green, fibrous tissue is purple/white). Top panels represent the entire callus (4X stitch, scale bar = 2000 $\mu$m), bottom panels represent zoomed in regions of cartilage and fibrous tissue (20X stitch, scale bar = 200 $\mu$m). Histologic analysis of callus morphology at 7 dpf; % cartilage area, cartilage area, callus area, (mean ±SD, n = 8 mice/condition). Mann-Whitney test; p*<0.05. (B) Heatmap of relative expression (depicted by z-score) of the top 138 genes with the greatest pseudotime-dependent expression changes in Intact (left). X-axis is increasing pseudotime, originating at αSMA+ progenitors, ending at hypertrophic chondrocytes. (C) Pseudotime regression plot of normalized expression by stromal cells in Intact (black) and Ischemic (pink) as a function of increasing pseudotime, along the respective trajectories with 95% confidence intervals and % change in Area Under the Curve (AUC). (D) Heatmap of relative gene expression of selected fibroblastic genes upregulated in Ischemic calluses at 7 dpf.*

*<u>Ischemic/hypoxic stress leads to elevated Cdk8 expression:</u>* Unbiased differential gene expression analysis revealed 563 DEGs at Padj<0.05, which were associated with biological pathways related to immune response, vascularization, osteogenesis, chondrogenesis, and stress response. Cyclin dependent kinase 8 (*Cdk8*) was amongst the highest upregulated genes under ischemic conditions (Fig 2A). CDK8 has been demonstrated to impede cell reprogramming and differentiation across multiple cell types, including fibroblast differentiation to hepatocytes and adipocytes[10]. Pseudotime analysis of *Cdk8* expression revealed highest elevation under ischemic conditions early on in differentiation (Fig 2B). Corroborating the potential role of ischemia in modulating *Cdk8* expression in stromal progenitors, hMSC cultured under hypoxic conditions exhibited upregulation of *CDK8* expression (Fig 2C).

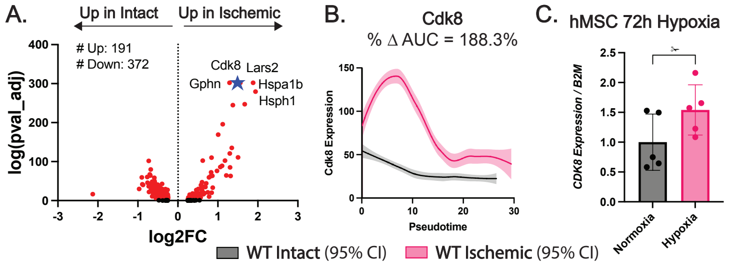

***Figure 2:*** *Ischemic/hypoxic stress leads to elevated Cdk8 expression. (A) Volcano plot of differentially expressed genes (Padj < 0.05 in red) between Intact and Ischemic at 7 dpf. Positive fold changes indicate upregulation in Ischemic. Blue star indicates Cdk8. Number of differentially upregulated and downregulated genes are listed. (B) Pseudotime regression plot of normalized Cdk8. (C) CDK8 gene expression by hMSCs cultured in normoxia (20% oxygen) or hypoxia (1% oxygen) for 72 hours via qPCR. N = 5 unique donors. Mean ± SD. *P<0.05, two-tailed paired t-test.*

*<u>CDK8 inhibition promotes chondrogenesis in vitro:</u>* Treatment of ATDC5 cells with Senexin B or SNX631-6 (data not shown) dramatically increased the expression of *Acan, Col2a1,* and *Col10a1* (Fig. 3A). 3D hMSC chondrogenesis in the presence of Senexin B for 14 days also resulted in increased *ACAN* and *COL2A1* (Fig 3B), and Senexin B-treated pellets exhibited ~25% greater sulfated glycosaminoglycan (sGAG) content (data not shown). Senexin B-treated pellets were larger with more robust and homogeneous sGAG content, with fewer sGAG-devoid regions compared to the control (Fig. 3C).

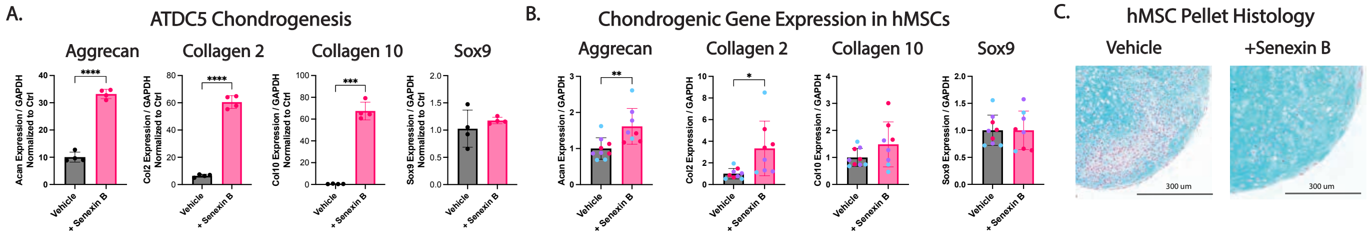

***Figure 3:*** *CDK8 inhibition promotes chondrogenesis in vitro. (A) Gene expression of ATDC5 chondrogenesis -/+ 400nM Senexin B after 14 days, n=4 replicates. *P<0.05, **P<0.01, ***P<0.001, ****P<0.0001, Welch-corrected 2 sided-t test (B) Gene expression of 3D hMSC chondrogenic pellets -/+ 400nM Senexin B after 14 days, n = 3 donors, 2-3 replicates/donor *P<0.05 Wilcoxon test. (C) Representative alcian blue stain of hMSC pellets.*

*CDK8 inhibition promotes fracture healing in vivo:* Ischemic fractures treated with the CDK8 inhibitor SNX631-6 during the early chondrogenic phase (5-10 dpf) (Fig 4A) caused a slight increase in callus size, but not bone volume by 15 dpf (Fig 4B). Histologic analysis revealed an increase in cartilage content, corroborating our *in vitro* findings that CDK8 inhibition promotes chondrogenesis. Interestingly, treatment during the chondrogenic to osteogenic transitionary phase (5-15 dpf) (Fig 4D) resulted in increased callus size and bone formation (Fig 4E), but not cartilage content (Fig 4F). This demonstrates the ability of CDK8 to promote cell differentiation.

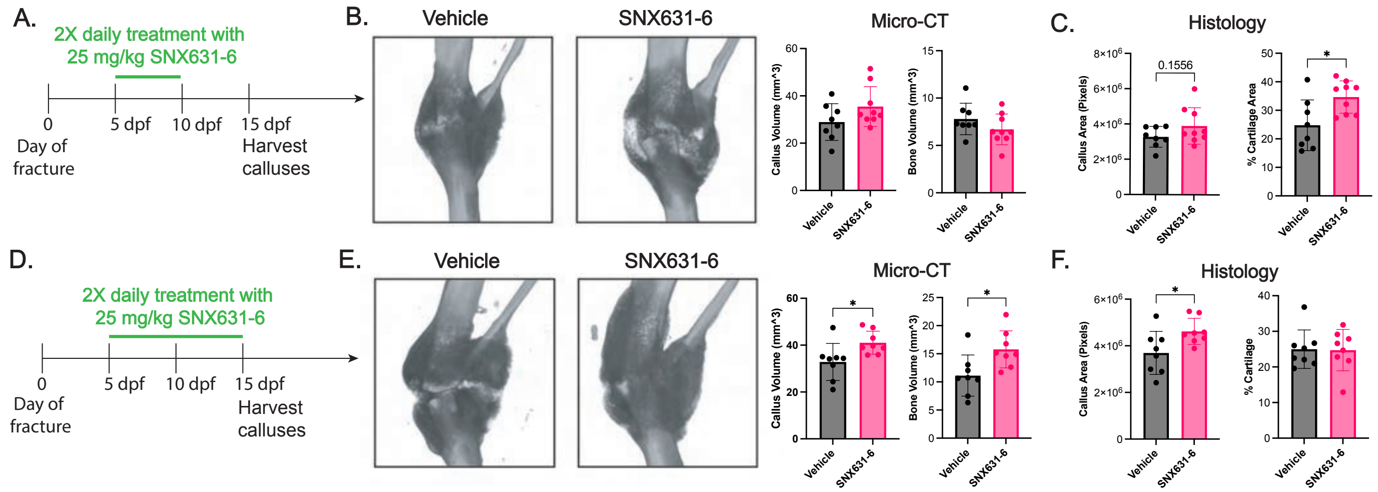

*Figure 4: CDK8 inhibition promotes fracture healing in vivo. (A) Timeline of oral treatment with SNX631 from 5-10 dpf, harvest 15 dpf. (B) µCT analysis of callus morphology in vehicle and SNX631-6 treated mice at 15 dpf. (C) Histologic analysis at 15 dpf of mice treated from 5-10 dpf using Safranin-O/Fast Green. (D) Timeline of oral treatment with SNX631 from 5-15 dpf, harvest 15 dpf. (B) µCT analysis of callus morphology at 15 dpf. (C) Histologic analysis at 15 dpf of mice treated from 5-15 dpf. *P<0.05 two-sided t-test.*

DISCUSSION: Here we revealed the impaired chondrogenic cell differentiation and increased fibrosis that underpins impaired ischemic fracture healing[5]. We found that impaired stromal progenitor differentiation during ischemic fracture healing is associated with upregulation of *Cdk8*. This corroborates the work by other groups that CDK8 inhibits fibroblast reprogramming and differentiation[6,10]. Using selective pharmacologic inhibitors, we demonstrated that CDK8 impedes chondrocyte differentiation, resulting in reduced chondrogenesis *in vitro* and *in vivo*. Most importantly, we confirmed that *in vivo* CDK8 inhibition using an orally-available drug improved callus formation, with enhanced chondrogenesis and callus bone formation, consistent with improved healing. These are clinically relevant results as they suggest that oral treatment with CDK8 inhibitors can modulate impaired fracture healing, addressing a major unmet clinical need.

REFERENCES:
[1] Einhorn & Gerstenfeld 2015 Nat Rev Rheumatol (PMID 25266456)
[2] Bahney et al. 2018 J Orthop Res (PMID 30370699)
[3] Einhorn et al. 1995 J Bon Miner Res (PMID 8585432)
[4] Miclau et al. 2017 Front Cell Dev Biol (PMID 28523266)
[5] Lu et al. 2007 J Orthop Res (PMID 17019699)
[6] Adler et al. 2012 Cancer Res (PMID 22345154)
[7] Fukasawa et al. 2021 Oncogene (PMID 33727660)
[8] Samokhin et al 2018 Sci Transl Med (PMID 29899023)
[9] Xiong et al. 2017 Oncotarget (PMID 29050340)
[10] Li et al. 2023 Cell Rep (PMID 37235474)

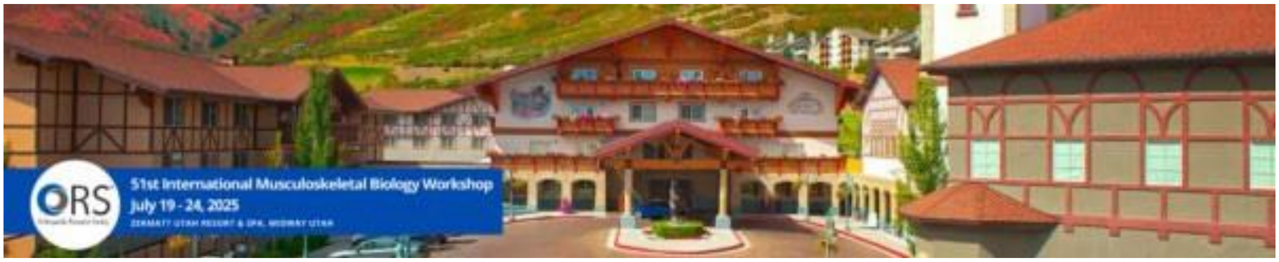

### Charles H. Turner

### Young Investigator Bone Research Award Recipient

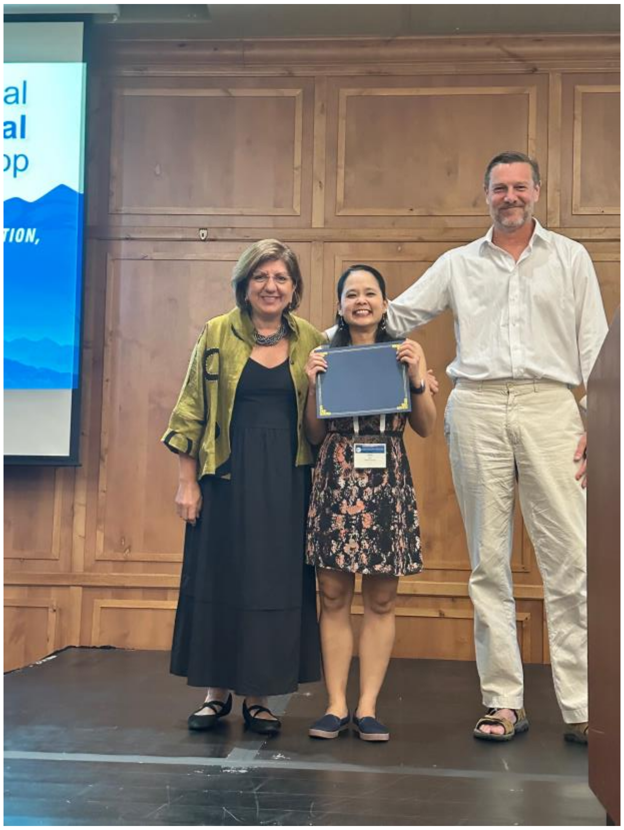

### Eccentric Muscle Contraction Increases Trabecular and Cortical Bone Volume in Aged Mice

*Hui Jean Kok, PhD[1], Daniel J. Horan, MA[1], Joshua R. Huot, PhD[1], Alexander G. Robling, PhD[1]*
*[1]Indiana University School of Medicine, Indianapolis, IN, USA*

Skeletal muscle is a major contributor of mechanical signals to the bone, where muscular contractions increase bone strains, resulting in mechanotransduction. Subsequently, adaptive responses triggering bone modeling and remodeling occurs leading to increased bone accumulation and mineralization. However, the type of muscle contraction required to produce optimal bone formation has yet to be explored. This is especially true for aged bones where bone formation rates are slower. The goal of this study is to determine if isometric, eccentric or a combination of both types of muscle contraction produce optimal bone gain. C57Bl/6 mice aged 6- and 20-month-old males were obtained from the National Institute on Aging rodent colony. Each cage houses 4 mice and were stratified to 4 groups: 1) Isometric (Iso), 2) Eccentric (ECC), and Isometric+Eccentric (Iso+ECC) contractions, and 4) Non-stimulated (NS) cage controls exposed to equal amounts of isoflurane inhalation. Mice (n=9/age/group) were unilaterally stimulated using needle electrodes targeting the sciatic nerve, while the foot is taped to the foot pedal of a 300C dual-mode servomotor transducer (Aurora Scientific), with a fixed knee joint. Each stimulation was induced for 800 ms pulse duration with a frequency of 150 Hz and were repeated 4 times with 12 seconds rest in between each stimulation (1 set). Iso contraction was performed with a 90°-foot angle (neutral position) throughout the stimulation and ECC with 200 ms at 90°, 300 ms at 70° and 300 ms at 110°-foot angle. Each training session consisted of 4 sets of 5 stimulations with 3 minutes rest in between each set. Iso+ECC animals performed 2 sets of Iso and 2 sets of ECC contractions. Animals were trained for 3 sessions a week for 4 weeks, along with muscle peak torque measurements before and after training sessions. Animals were allowed to recover for 1 week after the last training session and hindlimb muscles and bones were harvested 10 days after the last session. There were negligible changes in the body weight of young mice, however old mice showed progressive decreases in body weight throughout the study indicating the occurrence of osteosarcopenia and that the effects of muscle stimulation remained locally within the stimulated limb. There were significant decreases in dorsiflexion peak torque (-22%) in young mice and muscle mass (TA, EDL & soleus) in all mice that underwent ECC muscle contraction, suggesting ECC-induced muscle damage. While all stimulated tibias had increased cortical thickness vs. contralateral unstimulated tibias, a divergent trabecular bone phenotype between young and old mice were observed. Young mice that performed Iso muscle contraction displayed significant increases in tibial trabecular bone volume fraction (Tb. BV/TV: +32%) and thickness (Tb. Th: +12%) when compared to contralateral tibia. Intriguingly, old mice exhibited significant increases in tibial Tb. BV/TV (+53%) and Tb. Th (+22%) only from ECC muscle contraction vs. contralateral unstimulated tibia. Thus, eccentric muscle contraction produced a larger osteogenic effect in old mice, but isometric muscle contraction is more beneficial for young mice. We will further identify the microstructural changes within the muscle and bone.

*HJK is supported by the IU Charles H. Turner Young Investigator Bone Research Award & NIH-NIA K00AG068438.*

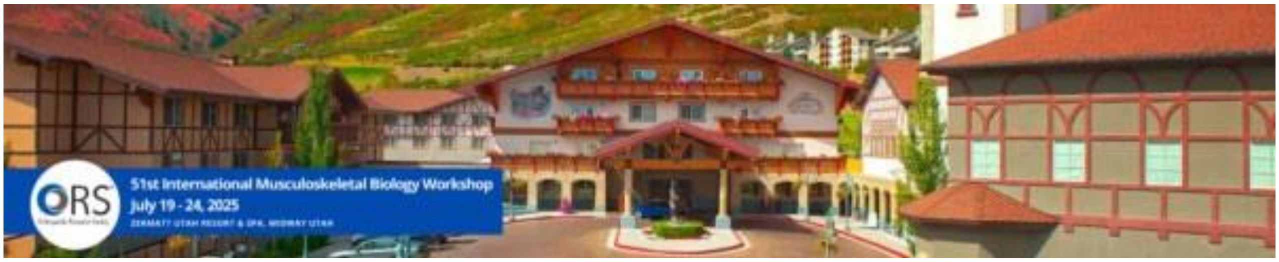

### Alice L. Jee

### Award Recipients

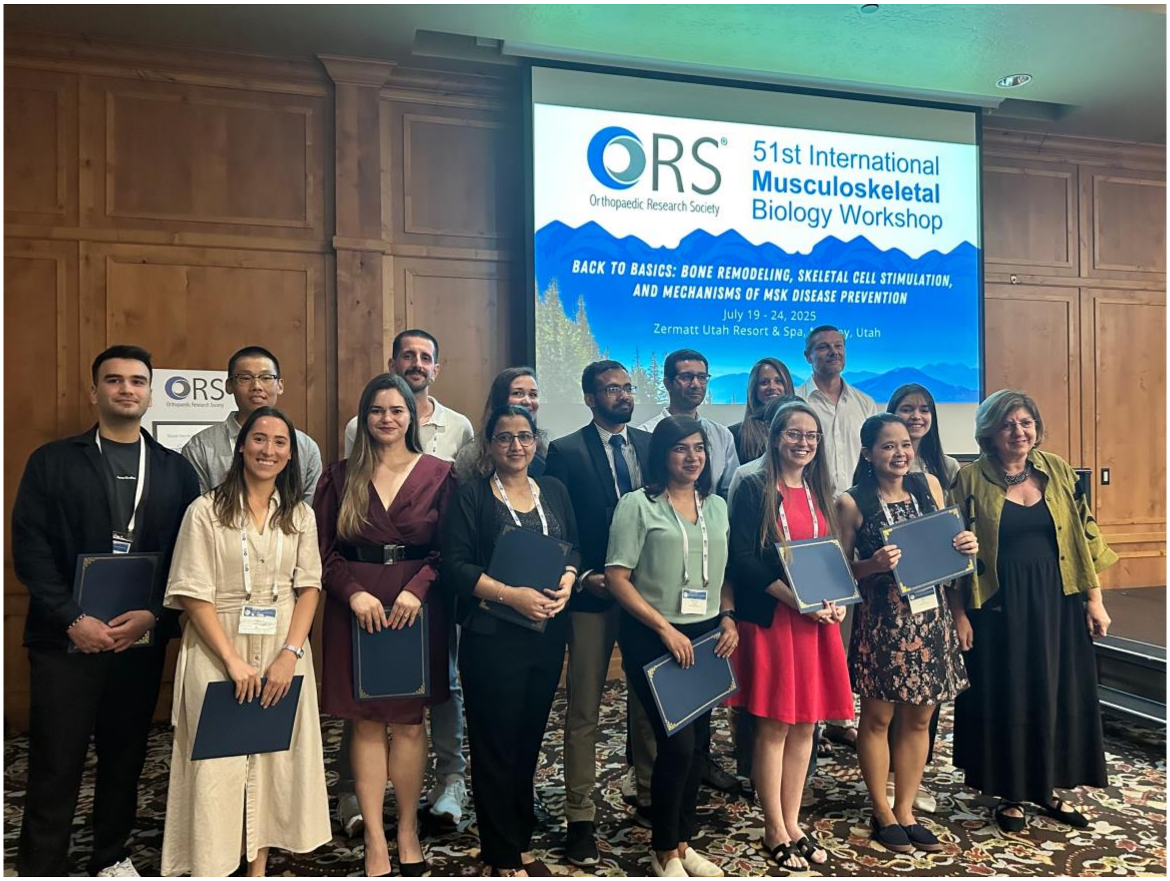

### Identification of subchondral bone endotypes in hip osteoarthritis

Assil-Ramin Alimy[1], Maximilian Möhlmann[1], Julius Rosenfeld[1], Eva Goedecke[1], Frank Timo Beil[1], Jeroen Geurts[2], Tim Rolvien[1]
[1]University Medical Center Hamburg-Eppendorf, Department of Trauma and Orthopaedic Surgery, Hamburg, Germany
[2]University Hospital Lausanne and University of Lausanne (CHUV-UNIL), Department of Musculoskeletal Medicine, Rheumatology, Lausanne, Switzerland.

Background
The hip is one of the anatomical sites most commonly affected by osteoarthritis (OA). While pathomechanistic aspects of hip OA are still largely unclear, current treatments focus on symptom relief without addressing patient-specific characteristics.

Purpose
This study aimed to identify distinct endotypes based on subchondral bone properties in hip OA to provide a reliable basis for future research and treatment strategies.

Methods
Femoral heads were collected during total hip arthroplasty (n=38). Standardized osteochondral specimens were prepared and underwent a detailed histological assessment of articular cartilage and subchondral bone. Additionally, quantitative backscattered electron imaging was performed to investigate mineralization and osteocyte lacunae properties. Principal component analysis (PCA) was conducted to reduce the dimensionality of the dataset, and hierarchical clustering was performed to group the specimens according to their similarity.

Results
Cluster analysis revealed four distinct groups based on their osteochondral characteristics. Clusters I and II were histologically characterized primarily by subchondral low bone mass, in contrast to the high bone mass phenotype of cluster IV. Histological assessment of the subchondral bone revealed that osteoblast and osteoid indices progressively increased across the clusters, with the highest amount in clusters III and IV. Remarkably, Cluster IV was notable for its low mineralization, accompanied by a higher number and larger osteocyte lacunae. Additionally, reduced bone marrow adipocyte size (a surrogate for lipolysis) was observed in both clusters III and IV and was also associated with increased subchondral bone formation in the overall cohort.

Conclusion
Our data identify four distinct subchondral bone endotypes with unique profiles of microstructure, mineralization, and cellular activity, reflecting varying disease activity, and possibly treatment needs, such as osteoanabolic treatment in clusters I and II and antiresorptive treatment in clusters III and IV. Furthermore, our results suggest a previously unrecognized role of lipolysis in modulating hip OA endotypes.

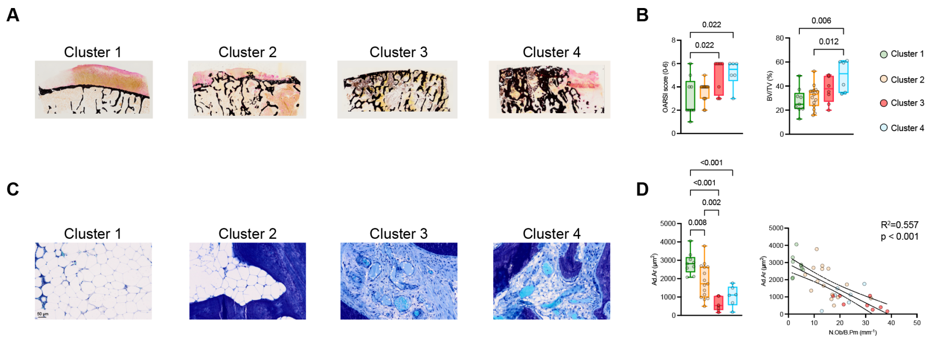

Figure legends
A) Representative von Kossa-stained sections of osteochondral specimens from patients with hip OA in each cluster. B) Histological evaluation of OARSI scores (left) and bone volume quantification (right). C) Representative toluidine blue-stained sections of bone marrow across clusters. D) Quantification of bone marrow adipocyte size (left) and its association with osteoblast density (right).

**Asfotase alfa treatment in a mouse model of later-onset HPP with comorbid chronic kidney disease**

Flavia Amadeu de Oliveira[1], Cintia Kazuko Tokuhara[1], Sonoko Narisawa[1], Elis J. Lira dos Santos[2], Marcel Rodrigues Ferreira[3], Brian L. Foster[2], José Luis Millán[1]

[1] Sanford Children's Health Research Center, Sanford Burnham Prebys Medical Discovery Institute, La Jolla, CA, USA

[2] Division of Biosciences, College of Dentistry, The Ohio State University, Columbus, OH, USA

[3] Molecular Genetics and Bioinformatics Laboratory, Experimental Research Unity, Botucatu Medical School, São Paulo State University, Botucatu, SP, Brazil

Hypophosphatasia (HPP) is a rare inherited metabolic bone disorder caused by loss-of-function mutations in the *ALPL* gene that result in deficient tissue-nonspecific alkaline phosphatase (TNAP) activity and impaired skeletal/dental mineralization. Asfotase alfa (AA) improves survival and skeletal/dental mineralization and is the only approved treatment for HPP. Because of its mineral-binding properties, AA also binds to minerals present at sites of ectopic calcification, such as the vasculature. The overexpression of TNAP in either the vascular media or intima has been shown to induce severe vascular calcification. Here we examined what might occur if a disease that features medial artery calcification, such as chronic kidney disease-mineral and bone disorder (CKD-MBD), were to develop in a subject with HPP undergoing AA treatment. Two-month-old $Alpl^{Prx1/-}$ (HPP) or $Alpl^{flox/-}$ (Control) mice were treated with subcutaneous AA injections (8.2 mg/kg, 3×/week) for four months. After three months of treatment, CKD was induced via a diet containing 0.2% adenine and 1.8% phosphorus. Mice were euthanized at six months, and skeletal, renal, cardiac, and vascular tissues were analyzed using biochemistry, micro-computed tomography (micro-CT), immunohistochemistry (IHC), proteomics, and gene expression profiling. Biochemical analysis revealed sex-specific responses to AA treatment, with females displaying ALP levels slightly increased with AA but significantly increased with CKD treatment in control and HPP mice. In males, ALP activity was significantly elevated in AA and AA+CKD induced treatment, with only a slight increase in the CKD only group. ALP levels were highest in HPP mice treated with both AA and CKD. Micro-CT analysis showed improved bone parameters (bone volume fraction, trabecular thickness/density, and cortical thickness) with AA. However, the improvements were dampened when CKD was introduced, compared to untreated $Alpl^{flox/-}$ controls. IHC in kidney tissues revealed increased TNAP immunolocalization in mice treated with AA+CKD, followed by AA alone, in both sexes. Notably, ectopic renal and vascular calcification was observed in AA+CKD-treated $Alpl^{Prx1/-}$ mice, with a greater incidence in females. These findings highlight the potential for AA to exacerbate ectopic calcification in the presence of CKD, underscoring the importance of evaluating TNAP-targeted therapy safety in HPP patients with comorbid conditions associated with ectopic calcification.

Keywords:

Preclinical model, mouse, loss-of-function, hypophosphatasia, chronic kidney disease-mineral and bone disorder

**Single cell profiling reveals transcriptional reprogramming of pre-osteoblasts, osteoblasts and osteocytes by diabetes and restoration by activation of the PTH1R/SIK axis.**

*Sudipta Baroi[1], Nisreen Akel[1,2], Daniel Garcia Sanchez[1,2], Joan Pizarro-Gomez[1,3], Ryan Allen[1], Hiroyuki Okada[4], Intawat Nookaew[5,6], Marc Wein[7], Teresita Bellido[1,2,6]*

[1]*Department of Physiology and Cell Biology, University of Arkansas for Medical Sciences, Little Rock, AR, USA;* [2]*Central Arkansas Veterans Healthcare System, John L. McClellan, Little Rock, AR, USA;* [3]*Institute of Advanced Molecular Medicine, Universidad CEU San Pablo, Madrid, Spain;* [4]*Center for Disease Biology and Integrative Medicine, The University of Tokyo, Tokyo, Japan;*
[5]*Department of Biomedical Informatics, University of Arkansas for Medical Sciences, Little Rock, AR, USA;* [6]*Winthrop P. Rockefeller Cancer Institute, University of Arkansas for Medical Sciences, Little Rock, AR, USA;*
[7]*Massachusetts General Hospital, Boston, MA*

Recent findings demonstrate that bone anabolic ligands of the parathyroid hormone receptor (PTH1R), PTH and abaloparatide (ABL), reverse the diabetic (DM) bone signature at the tissue, cellular, and molecular levels. To identify bone cell populations and responsible downstream signaling, we performed single-cell RNA sequencing of long bones from control (C) or DM mice, treated with PTH, ABL, or the salt inducible kinases inhibitor SK-124 (SK), which activates similar pathways as PTH/ABL bypassing the PTH1R. We transcriptionally profiled 23,759 mesenchymal lineage cells, with 25, 30, and 1.8% being preosteoblast/osteoCARs (preOb), osteoblasts (Ob), and osteocytes (Ot). DM increased preObs (150%) without changing Ob or Ot. PTH/ABL increased preObs (130–140%) whereas PTH/ABL/SK increased Ob (150–260%) and Ot (170–480%) in both C/DM, at the expense of adipoCARs (50%) and chondrocytes (60%).10 markers (*Limch1, Wif1, Angpt4, Kcnk2, Mmp13, Spp1, Slit2, Vdr, Serpine2, Tnc*) defined the preOb cluster, and were all decreased by DM, and 6 of them (*Limch1, Wif1, Kcnk2, Tnc, Vdr, Serpine2*) were increased by PTH/ABL/SK in both C/DM. 15 markers (*Col1a1/a2, Bglap1/2/3, Entpd3, Car3, Cgref1, Lman1, Ifitm5, Sp7, Bmp1, Rcn3,Nrp2, Fkbp11*) defined the Ob cluster, and DM decreased 7 (*Col1a1/a2, Car3, Cgref1, Lman1, Bmp1, Rcn3*) whereas PTH/ABL/SK increased 12 (*Col1a1/a2, Bglap, Sp7, Car3, Cregf1, Lman1, Ifitm5, Bmp1, Rcn3, Nrp2, Fkbp11*) in both C/DM. 9 markers (*Sost, Dmp1, Dkk1, Phex, Mepe, Pdpn, Ptprz1, Bambi, Enpp6*) defined the Ot cluster; and 6 (*Phex, Ptprz1, Dkk1, Pdpn, Enpp6, Bambi*) were decreased by DM and 7 (*Dmp1, Phex, Ptprz1, Dkk1, Pdpn, Enpp6, Bambi*) were increased by PTH/ABL/SK in C/DM. In contrast to other Ot markers, Sost was increased by DM (~1,000%), and PTH, ABL, and SK all effectively decreased it in both C/DM (to ~10-60% of veh). Further, DM decreased the expression of canonical and non-canonical Wnt target genes and ligands in all clusters and, conversely, PTH/ABL/SK increased these genes. For all clusters, ABL was ~2x more potent in C whereas PTH was ~2x more potent in DM; and SK mimicked ABL in C and PTH in DM, but also exhibited unique effects on cluster cell number, cluster markers and Wnt-related genes. We conclude that the decreased bone strength, damaged microarchitecture, and reduced bone formation induced by DM and corrected by the PTH1R/SIK axis, are due to profound transcriptional reprogramming of preOb, Ob and Ot impacting Wnt signaling in bone.

### Pharmacologic activation of Piezo1 restores load-induced bone formation in old mice

*Ana Resende-Coelho, MS[1], Adriana Marques-Carvalho, PhD[1], Jinhu Xiong, PhD[1], Maria Almeida, PhD[1]*
*[1]University of Arkansas for Medical Sciences, Little Rock, AR, USA*

Mechanical loading is a potent stimulator of bone formation [1]. Nonetheless, the osteogenic response to mechanical stimulation is reduced with aging [2], and the mechanisms responsible remain unknown. Piezo1, a mechanosensitive calcium channel, is essential for load-induced bone formation and plays a critical role in mitochondrial adaptation to mechanical signals [3]. Skeletal aging is associated with a decrease in Piezo1 expression, impaired mitochondrial respiration, and lower levels of nicotinamide adenine dinucleotide ($NAD^+$) – an essential cofactor for cellular energy metabolism [4,5]. Based on this evidence, we investigated whether stimulating mitochondrial activity can overcome the deleterious effects of aging on mechanoresponsiveness. To achieve this, we utilized the $NAD^+$ precursor nicotinamide riboside (NR) and/or activated Piezo1 with the chemical agonist Yoda2. We treated osteoblastic cells from 6 (young) and 22-month-old (old) C57BL/6 mice with either Yoda or NR for 24 hours and assessed their mitochondrial respiration by Seahorse (**Figure 1A**). Mitochondrial respiration was decreased in cultured osteoblastic cells from aged mice compared to young controls. Nonetheless, combined treatment with NR and Yoda significantly enhanced both basal and ATP-linked mitochondrial respiration in aged osteoblastic cells. Prompted by this *in vitro* evidence, we applied axial tibial compressive loading to the left tibiae of 22-month-old female C57BL/6 mice with a force of 8.5 newtons to achieve a strain magnitude of 1200με (**Figure 1B**). The right leg was used as an internal control. Mice were loaded for 2 weeks, during which they were simultaneously treated with vehicle, NR (1 g/kg/day) in drinking water, Yoda2 (2.5 mg/kg/day) via intraperitoneal injection 30 minutes before each loading session, or the combination of NR+Yoda2 (**Figure 1C**). Micro-CT measurements in vehicle- and NR-treated mice showed that loading did not alter cortical thickness. Nevertheless, loading increased cortical thickness in mice treated with Yoda2 and Yoda2+NR (**Figure 1D**). This cortical thickening was associated with an enhanced periosteal bone formation rate (BFR) and mineral apposition rate (MAR). Specifically, load-induced periosteal BFR and MAR increased by 40% in the vehicle and NR groups, while Yoda2 and Yoda2+NR treatments resulted in 60% and 70% increases, respectively (**Figure 1E**). In the endocortical surface, load-induced BFR increased by 20% in vehicle and NR-treated mice. Yoda2 and Yoda2+NR treatments led to a 50% and 70% increase, respectively (**Figure 1F**). Together, these findings demonstrate that pharmacological activation of Piezo1 restores load-induced bone formation in aged mice. NR improves the effects of Yoda2, most likely via actions on the mitochondria. This pharmacological approach is a promising strategy to counteract the deleterious effects of aging on bone mechanoresponsiveness.

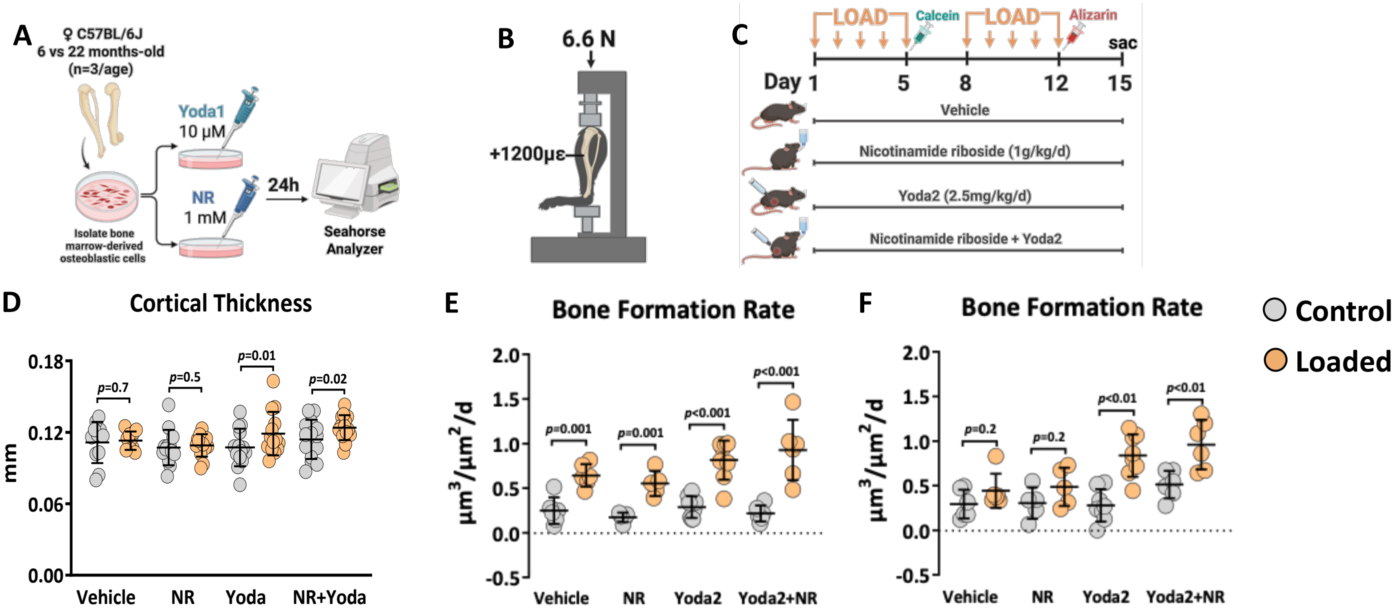

### References

[1] Ozcivici E, et al. *Nat Rev Rheumatol*. 2010;6(1):50-59. [2] Resende-Coelho A, et al. *Aging (Albany NY)*. 2025;17(3):610-629. [3] Li X, et al. *eLife*. 2019;8:e49631. [4] Li X, et al. *Aging Cell*. 2023;22(6):e13846. [5] Kim HN, et al. *NPJ Aging Mech Dis*. 2021;7(1):8.

### Short- and long-term adaptation in blood vessels within the murine hindlimb induced my mechanical loading

*Taylor deVet[1,2], Mahmoud Moussa [2,3], Svetlana Komarova [1,2,3,4,] Bettina M. Willie [1,2,3]*

*1Department of Biological and Biomedical Engineering, McGill University 2Research Centre, Shriners Hospitals for Children-Canada 3Faculty of Dental Medicine and Oral health sciences, McGill 4 Department of Biomedical Engineering, University of Alberta*

**Introduction:** Blood flow is increased with exercise and through induction of hemodynamic stimuli (shear and circumferential stress) on blood vessels remodeling occurs. In vivo compressive tibial loading in mice has been shown to increase overall limb perfusion acutely, evidenced by similar perfusion ratios after each episode of loading over a two-week period (Gohin 2020). However, the extent of short- and long-term vascular adaptation due to loading are unclear, both in terms of the overall limb vascularity, as well as the micro-vascularity in the bone cortex. We examined acute and chronic changes in femoral, saphenous, popliteal arteries, cortical bone vascular porosity, and cortical bone (re)modelling.

**Methods**: In vivo cyclic loading was applied to the left tibia of nine, 26-week-old female C57BL6/J mice (5x a week, 2 weeks, right tibia non-loaded). Ultrasonography was conducted before and after loading on days 1,3,7, and 9 as well as at baseline (before loading) and endpoint (after 15 days of loading). In vivo microCT and time-lapse morphometry was performed on days -2 and 15. At endpoint, microfil contrast agent was perfused into vasculature via the left ventricle and was analyzed via both microCT and synchrotron tomography. Synchrotron tomography was also used to analyze the vascular and lacunar pore properties within in the cortical bone.

**Results**: Each loading episode led to a significantly greater acute blood flow in the small vessels - popliteal and saphenous arteries - (femoral at day 7, 9), which was coincident with greater cortical bone thickness, and area, as well as volume of newly formed cortical bone at both the metaphysis and diaphysis (Fig. 1A, C). Chronic effects of loading were seen as changes in both popliteal and femoral artery blood flow velocity from day 0 to 15, with no significant change to the saphenous artery (Fig. 1C). A greater bone vascular volume and porosity could be seen via synchrotron tomography in the metaphyseal region as well as lacunar density and porosity in endocortical diaphyseal cortical bone due to loading (Fig. 1B).

**Conclusion:** We demonstrate that compressive limb loading results in adaptation to both the bone and local hindlimb vasculature. Blood flow to the hind limb vessels was increased acutely due to loading, which was likely in response to the increased induced bone and tissue strain. In the longer-term, loading led to more targeted vascular adaptations only seen in the femoral and popliteal arteries, but not in the saphenous. This shows that some vessels adapt to longer term loading, while others only change acutely after each loading episode. We also demonstrated that the microvasculature embedded within the bone adapts spatially, with more changes being seen in the cortical bone in the proximal metaphysis than in the mid-diaphysis. This demonstrates that vascular adaptation plays a significant role in loading induced bone (re)modeling.

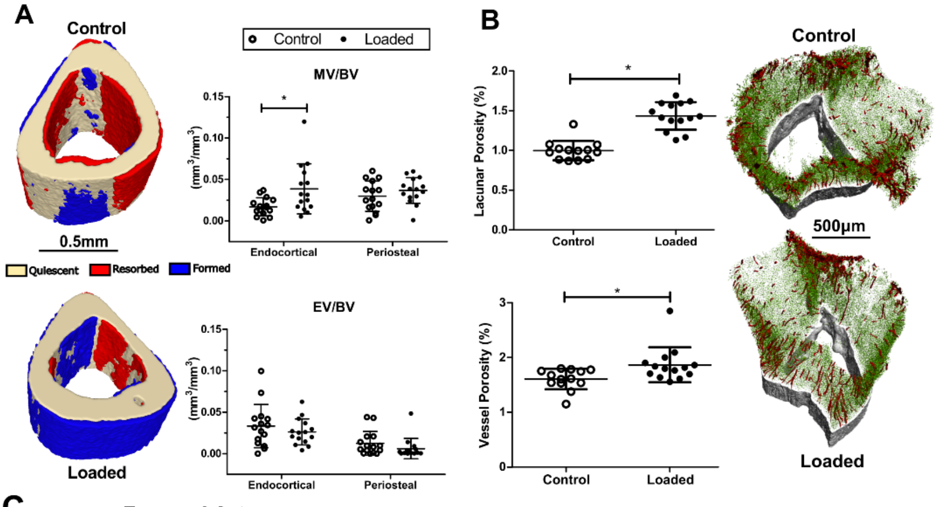

Figure 1: A) Left: Timelapse morphometry in control and loaded limbs Right: newly formed (MV/BV) and resorbed (EV/BV) volume fraction, on endocortical and periosteal surfaces of cortical bone B) Left: 3-D reconstruction of osteocyte lacunae (green) and vascular canals (red) Right: Lacunar porosity and vascular porosity between control (white) and loaded (black) limbs. C) Left: Change in blood velocity time integral (VTI) in femoral, saphenous, and popliteal arteries during loading episodes on days 1, 3, 7, and 9 Right: Chronic changes in VTI in all arteries measured as endpoint (day 15) – baseline ( day 0), Data presented as means ± SD; * p<0.05 indicates difference determined by paired t-test. ANOVA a) treatment effect b) day c) interaction

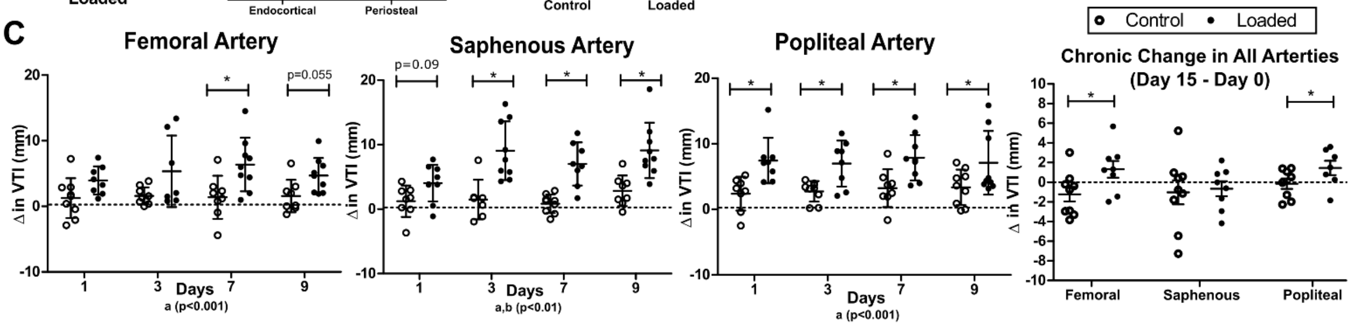

### Spatial-Temporal Specification of Progenitor Cells in the Tendon-to-Bone Interface: from Mouse to Human

*Julia Robbins[1], Angel Moonilall[1], Saswati Kar[1], Dylan Gordon[1], Fei Fang[1]*

*[1]Leni and Peter W. May Department of Orthopaedics, Icahn School of Medicine at Mount Sinai, New York, NY*

**Introduction:** The tendon-to-bone interface (enthesis), critical for daily rotator cuff function, is formed by a unique postnatal cell population with activated hedgehog (Hh) signaling, shown by expressing Gli1[1-2]. The Gli1-positive progenitors are shown to have stemness, build graded enthesis architecture, and have therapeutic potential[2-3]. The findings further raise an interesting question how the progenitor cells are orchestrated to differentiate and form a structural gradient. Additionally, whether human and mouse enthesis development are mediated by cell populations with similar profiles is unknown. Therefore, this study aims to decode enthesis spatial patterning regulated by Gli1-lineage (Gli1+) cells and examine convergence of enthesis development between mouse and human.

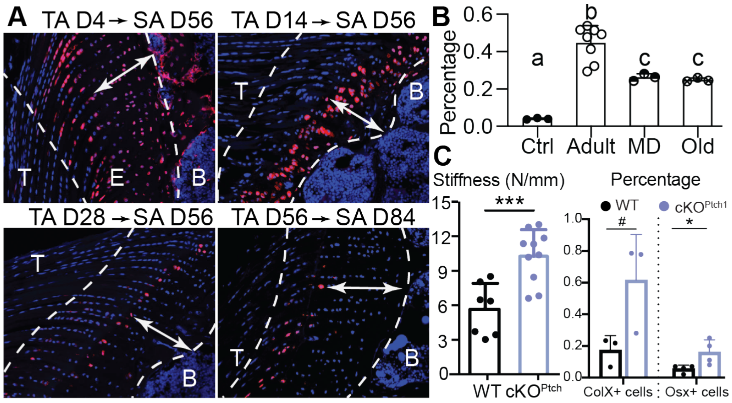

Figure 1. (A) Representative images showing spatial distribution of Gli1+ cells at entheses with tamoxifen (TA) injected and harvested (SA) at the indicated postnatal day (D). Scale bar, 50um. Entheses are highlighted between two dashed lines; T, tendon; E, enthesis; B, bone. (B) Percentages of Gli1+ cells in the running entheses of adult, middle-aged, and old mice and the control of different ages without running. (C) Stiffness of wildtype (WT, control) and cKO[Ptch1] entheses and percentage of cells expressing Col X or osterix (Osx). Mice were injected with tamoxifen at D4&D7 and sacrificed at D56.

**Methods:** <u>Animal models.</u> Gli1CreER[T2];Ai14 and Gli1CreER[T2]; Ptch1[fl/fl] mice ( Ptch1 as a Hh suppressor, Hh activation) were generated to track Gli1 activation and determine how Hh activation affects enthesis growth. Tamoxifen was injected at the indicated days. Gli1CreER[T2];Ai14 mice of adult, middle-aged and old groups were injected with tamoxifen and then subjected to treadmill running to examine cell mechanical responses to loading. <u>Human tissue collection.</u> The consent process and disposal of human fetal tissues were in full compliance. Fetal and adult enthesis cells were isolated for scRNA-seq analysis. <u>Tissue evaluation.</u> Both human and mouse supraspinatus enthesis were dissected, fixed, sectioned, and processed for histology and IHC[2-4]. Mechanical tests were conducted to evaluate mouse enthesis strength[4].

**Results:** Gli1-positive cells at D4 proliferated and differentiated into most of cell types for forming mature enthesis (Fig. 1A). Gli1-postitive cells at D14 only helped form the mineralized enthesis region. In contrast, Gli1-postitive cells at/after D28 maintained in the unmineralized region without expansion. More Gli1+ cells were observed in the running mice and younger mice after running had more Gli1+ cells (Fig. 1B). Hh activation postnatally results in higher enthesis stiffness and more expression of chondrogenic and osteogenic markers (Fig. 1C). Human enthesis began as a condensed primordium and matured into a graded fibrocartilage (Fig. 3A), expressing a low level of GLI1. scRNA-seq analysis of human fetal entheses found identical cell populations to mouse ones and revealed differentiation paths from progenitors to enthesoblasts and chondrocytes (Fig. 3B).

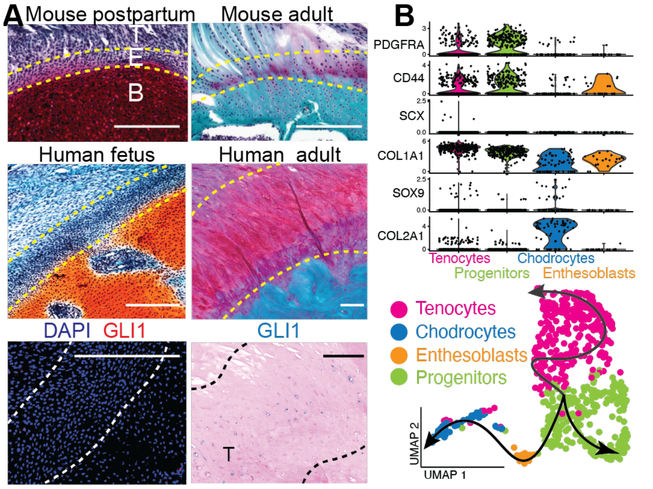

Figure 2. (A) safranin O staining of mouse (postpartum & adult) and human (fetus & adult) entheses; Immunostaining of GLI1 in human fetal and adult entheses. Scale bar: 200 µm. (B) Violin plots of markers for clustering fetal enthesis cells (top) and their differentiation trajectory (bottom).

<u>Discussion:</u> Our study showed that Hh activation is spatiotemporally regulated from development into maturity, potentially driving enthesis graded structure. Hh signaling were consistently activated by physiological loading[2], but mouse entheses at various life phases had different levels of mechano-sensitivity. Although Gli1 marks enthesis progenitor cells[2], continuous Hh activation in enthesis cells did not decrease cell differentiation to slow mineralization processes. However, more mineralized tissue were found in the Hh-activated enthesis. This observation is not surprising since Hh signaling in growth plate regulates both cell proliferation and osteogenic differentiation. Our comparison between mouse and human entheses demonstrated the conservation between species[5-6]. This will offer insights into translating discoveries from animal models into clinical application[6].

**References:** [1] S. Kult et al. Elife 2021 [2] F. Fang et al. Cell Stem Cell 2022 [3] A. Schwartz et al. Development 2015 [4] F. Fang et al. Sci Adv 2020 [5] H. Kronenberg et al. Nature 2003 [6] B. Zhang et al. Nature 2023

Immune-stromal dialogue during accelerated Bone healing at a single cell resolution

*Mohamed G. Hassan, BDS PhD[1,2], Anushka Gerald, BSc[1], Marguerite Mrad, PhD[3], Monika Bambouskova, PhD[3], Erica Scheller, DDS,PhD ,[1,2,4,5]*

*[1]Division of Bone and Mineral Diseases, Department of Medicine, Washington University School of Medicine, St. Louis, MO, USA. [2]Center of Regenerative Medicine, Washington University, St. Louis, MO, USA. [3]Department of Biomedical Engineering, Washington University, Saint Louis, MO, USA. [4]Division of Endocrinology, Metabolism & Lipid Research, Department of Medicine, Washington University School of Medicine, St. Louis, MO, USA. [5]Department of Cell Biology and Physiology, Washington University, Saint Louis, MO, USA.*

Inflammation and immune-stromal cell interactions are essential regulators of skeletal regeneration. Prior studies have shown that neural crest-derived bones exhibit accelerated healing compared to mesoderm-derived bones. However, the cellular and molecular mechanisms driving this enhanced regenerative response remain poorly understood. Here, by combining high-resolution whole-mount confocal imaging, high-dimensional spectral flow cytometry, targeted pharmaceutical approaches, and scRNA-seq, we demonstrate site-specific differences in blood vessel formation and the immune response that occur during enhanced healing of neural crest-derived bone. While immune cell populations appear similar in neural crest and mesoderm-derived bones before injury, significant differences in regenerating cell populations occur between sites post-injury. Specifically, neural crest-derived bone recruits larger, perivascular CD68+ macrophages as early as Day 3 post-injury. This response correlates with the infiltration of OSX+ cells and subsequent bone formation. Targeted depletion of macrophages using clodronate liposomes delays healing specifically in neural crest-derived bone, underscoring the functional importance of these cells. The analysis of publicly available scRNA-seq datasets further indicates that neural crest-derived osteoprogenitors and osteoblast clusters highly express *Ccl2*, *Tgfb1*, and *Csf1*, supporting a stromal-immune signaling axis that promotes superior bone regeneration compared to mesoderm-derived bone. Our data provides novel insights into the immunological differences underlying the site-specific mechanisms of bone healing and identifies potential stromal-derived immune regulators as potential therapeutic targets to enhance bone repair.

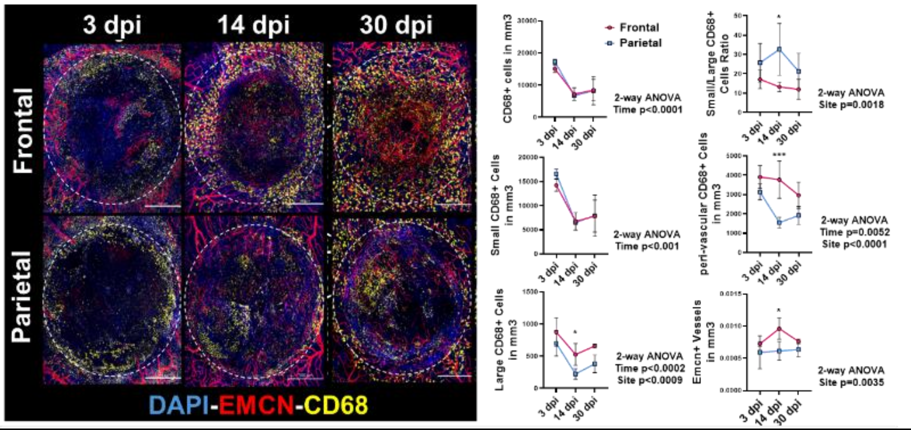

**Discovery of a Tendon/Ligament Stem Cell Causing Lumbar Spinal Stenosis**

Lingling Hu[1,2,4], Jun Sun[2], Kyle W Morse[1], Seoyeon Bok[2], Alisha R Yallowitz[2], Michelle Cung[2], Jason McCormick[3], Daniel Shinn[1], Anthony Pajak[1], Kasra Araghi[1], Eric Zhao, Sravisht Iyer[1*], Matthew B Greenblatt[2,4*]
[1]Department of Spine Surgery, Hospital for Special Surgery, New York, NY, USA
[2]Department of Pathology and Laboratory Medicine, Weill Cornell Medicine, New York, NY, USA
[3]Flow Cytometry Core Facility, Weill Cornell Medicine, New York, NY, USA
[4]Research Institute, Hospital for Special Surgery, New York, NY, USA
*co-corresponding author
iyers@hss.edu
mag3003@med.cornell.edu

Currently the identity of the stem cell giving rise to mature tenocytes in tendons and ligaments remains unknown. While several lineage markers of tenocytes have been identified, the identity of the tendon/ligament stem cell remains elusive. Lumbar spinal stenosis (LSS) is a common condition of narrowing of the spinal canal due in part to ligament hypertrophy resulting in nerve compression and lower extremity pain in 11% of older adults in the US. While this pathogenesis suggests that the LSS is caused by derangements in the self-renewal or differentiation capacity of a TLSC, identification of the TLSC is a prerequisite to understanding LSS pathogenesis.

Here we screen cell surface markers and fractionate tendon/ligamentum cells using a 7 color FACS panel. We identify a tendon/ligament stem cell (TLSC) defined by the markers (LIN-Thy-Sca1-CD73+CD140a-). TLSCs display formal evidence of stemness in the transplantation-based self-renewal and sit at the apex of their differentiation hierarchy in serial transplantation assays. The CRISPR-Cas9 lineage tracing system confirms the TLSC stemness. While undergoing self-renewal, they are capable of generating mature tenocytes during *in vivo* tendon organoid formation assays. The identity of the TLSC and its associated stemness properties were similar in all tendon/ligament tissues examined, suggesting that this represents a universal TLSC. Human counterpart of TLSCs were identified in ligamentum flavum specimens and display a conserved differentiation hierarchy and stemness features. In a LSS mouse, this TLSC is markedly expanded in the ligamentum flavum, and isolation and transplantation of the TLSC from the LSS mouse model to secondary hosts demonstrates that LSS enhances the tenocyte production capacity of this TLSC. This TLSC is expanded in the ligamentum flavum of LSS patients. Further, xenografting of equivalent numbers of human ligamentum flavum TLSCs from LSS and control patients demonstrates that the tenocyte production capacity of LSS patient derived TLSCs is enhanced on a per cell basis, demonstrating that the key feature of LSS pathology is intrinsic to the TLSC identified here. We also find myostatin, a secreted transforming growth factor, highly expressed in hypertrophic TLSC in both mice and human samples from our differential gene analysis, which is a potential therapeutic target for lumbar spinal stenosis.

Altogether, this work establishes new assays to formally assess stemness in tendon/ligament cells and identify a TLSC shared among tendon/ligament tissues in humans and mouse. Derangements in this cell and the secretion are central to LSS pathology which provides an insight to the cellular basis of LSS patients and therefore makes TLSCs and the secretion a potential therapeutic target for LSS, a disorder which currently lacks any disease modifying medical therapy.

Title: NLRP3 inflammasome is regulated in osteoclasts through a Tmem178-dependent mechanism that restricts calcium influx

*Khushpreet Kaur[1], Yael Alippe[1], Chun Wang[1], Nicholas Semenkovich[2,3], Saumya Bhagat[1], Kunjan Khanna[4], Yongjia Li[1], Nitin Pokhrel[1], Timothy Peterson[5], Deborah J. Veis[1], Yousef Abu-Amer[4,6], Roberta Faccio[4], and Gabriel Mbalaviele[1]*.*
*[1] Division of Bone and Mineral Diseases, Washington University School of Medicine; St. Louis, MO 63110, USA; [2] Data Science Institute, Medical College of Wisconsin; Milwaukee, WI 53226, USA; [3] Department of Medicine, Division of Endocrinology and Molecular Medicine; Milwaukee, WI 53226, USA; [4] Department of Orthopedic Surgery, Washington University School of Medicine; St. Louis, MO 63110, USA; [5] Health Span Technologies, Inc.; St. Louis, MO, USA; [6] Shriners Hospital for Children; St. Louis, MO, USA.*

Abstract: The NLRP3 inflammasome is a key driver of bone resorption in conditions ranging from low-grade to high-grade inflammation. This process is mediated by osteoclasts (OCs), which differentiate from macrophages in response to RANKL. While the non-autonomous effects of inflammasome activation on the OC lineage are well characterized, its cell-autonomous role remains unclear. To investigate this, we treated mouse bone marrow-derived macrophages (BMDMs) with RANKL and tracked NLRP3 expression. We observed a gradual decline in NLRP3 levels during osteoclastogenesis, which could be restored with LPS. When cultures were exposed to LPS and nigericin to assess inflammasome assembly, robust activation was seen in undifferentiated BMDMs, but not in OCs or their lineage-committed precursors. Mechanistic studies revealed that Tmem178, a protein that restrains $Ca^{2+}$ release from the endoplasmic reticulum (ER) and is highly expressed in OCs, suppresses NLRP3 inflammasome activation by restricting $Ca^{2+}$ release from the endoplasmic reticulum (ER). OCs deficient in Tmem178 exhibited increased inflammasome activity. Similarly, exogenous calcium or calcium released from culture substrata triggered inflammasome activation and enhanced resorptive function in OCs. In vivo, under conditions of high bone turnover—where OCs liberate $Ca^{2+}$ from bone—the assembly of the inflammasome was elevated. Importantly, deletion of Nlrp3 rescued the low bone mass phenotype in Tmem178-deficient mice. In conclusion, Tmem178 serves as a cell-autonomous regulator that limits ER-derived $Ca^{2+}$ release, thereby restraining NLRP3 inflammasome activation in osteoclasts under standard culture conditions. Loss of this regulation or increased intracellular $Ca^{2+}$ bypasses this brake, linking calcium signaling to inflammatory bone loss.

### Bi-directional control of YAP/TAZ Signaling Impacts Macrophage Function in Bone Healing

*Georgios Kotsaris[1], Madhura Nijsure[1], Sung Yeon Kim[1,2], Elizabeth Bernstein[1], Nathaniel Dyment[1], Robert Mauck[1,2], Joel Boerckel[1]*

*[1]University of Pennsylvania; [2]CMC VA Medical Center*

Georgios.kotsaris@pennmedicine.upenn.edu

**Introduction:** Bone can regenerate without scarring, but current treatments remain inefficient, costly, and limited in scope. Harnessing biological repair processes—especially inflammatory and mechanical cues—may enhance healing. Monocytes and pro-inflammatory (M1) macrophages (MΦs) infiltrate the fracture site within 24–48 hours, coinciding with peak mechanical stimuli from interfragmentary motion. As healing advances, MΦs transition to anti-inflammatory (M2) phenotypes and influence chondro/osteogenesis and angiogenesis [1]. Mechanical signals are transduced by YAP and TAZ, transcriptional regulators our group previously linked to skeletal development and regeneration [2, 3, 4]. While YAP/TAZ function in osteoblasts is established, their role in MΦs—particularly under mechanical strain—remains unclear [5, 6]. Here, we investigate how YAP/TAZ control MΦ mechanosensitivity and function in bone fracture healing.

**Results:** <u>MΦs in fracture repair:</u> Longitudinal analysis of bone fracture healing showed that MΦs localize to the periosteum and fracture hematoma during early stages (4 DPF). By 7 DPF, they accumulate in extramedullary regions and the soft callus, forming canopy-like structures over Col1⁺ osteoblasts and Col2⁺ chondrocytes. At later stages (10–14 DPF), MΦs infiltrate the hard callus, which contains Col10⁺ hypertrophic chondrocytes driving endochondral ossification (**Fig. 1A**). scRNA-seq of bone marrow cells from the fracture site at 3 DPF under stiff versus compliant fixation revealed eight transcriptionally distinct clusters (**Fig. 1B**), with monocytes/macrophages increased in the compliant group (**Fig. 1C**). <u>Effects of YAP/TAZ signaling on MΦ function:</u> To investigate YAP/TAZ function in MΦs, we performed TAT Cre-mediated recombination in BMDMs. Phalloidin staining revealed that WT MΦs exhibited small cell bodies with multiple protrusions, while both YAP/TAZ-knockout (KO) and gain-of-function (GOF) cells displayed increased cytoplasmic area and a rounded morphology (**Fig. 2A, B**). The expression of the pro-inflammatory marker IL-6 remained unchanged in YAP/TAZ-KO MΦs but was elevated in GOF MΦs (**Fig. 2C**). In contrast, the expression of the anti-inflammatory marker Arg1 was reduced by 60% in YAP/TAZ-KO MΦs, with no significant difference observed in GOF cells (**Fig. 2D**). YAP/TAZ-KO MΦs also exhibited impaired phagocytic capacity toward GFP-labeled E. coli particles (**Fig. 2E**).

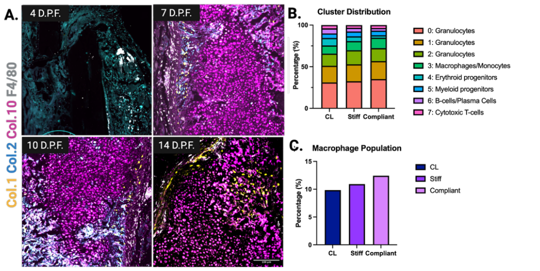

**Figure 1: Macrophage respond to mechanical stimuli *in vivo*.** **(A)** Immunofluorescence staining of the bone fracture callus. **(B)** scRNA-seq revealed eight subpopulations in the bone marrow. **(C)** Percentage of MΦs in contralateral unfractured (CL) limbs and in fractures with stiff or compliant fixation.

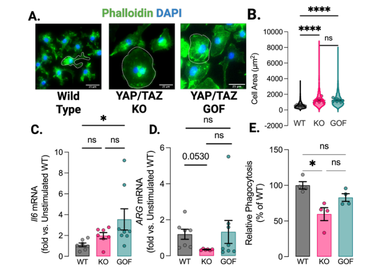

**Figure 2: Effects of YAP/TAZ signaling on macrophage function.** **(A)** Representative images of the actin cytoskeleton in unstimulated MΦs following TAT-Cre treatment. **(B)** Cell area quantification from phalloidin images. **(C, D)** qPCR analysis of IL-6 and Arg1 expression in unstimulated MΦs. **(E)** Phagocytosis of GFP-labeled E. coli, shown as percentage of internalized GFP.

**Discussion:** Our findings highlight a central homeostatic role for YAP/TAZ signaling in regulating MΦ morphology, inflammatory activation, and phagocytic function, with implications for tissue repair and immune regulation. Compliant fixation increased macrophage recruitment to the fracture site at 3 DPF, likely due to altered mechanical loading and enhanced angiogenesis. To dissect the role of YAP/TAZ, we developed an *in vitro* system using a cell-penetrating Cre recombinase to generate YAP/TAZ loss- and gain-of-function MΦs. Consistent with prior work showing that YAP/TAZ mediate responses to mechanical stimuli [7, 8], both loss and gain of function disrupted cytoskeletal organization and altered cell size and shape. Functionally, YAP/TAZ perturbation enhanced pro-inflammatory signaling and impaired phagocytosis. These results support the existence of a cytoskeletal feedback loop, wherein YAP/TAZ not only respond to but also regulate cytoskeletal dynamics, consistent with our previous findings in endothelial cells [9]. We have now generated MΦ-conditional YAP/TAZ and LATS1/2 knockout mice (using LysM-Cre), and our ongoing studies are investigating the role of MΦ YAP/TAZ signaling in mechanical load-regulated fracture repair.

**Significance:** Here we identify YAP/TAZ signaling as a key regulator of MΦ structure and function and identify mechanical cues as a regulator of MΦ mobilization *in vivo*. Advancing our understanding of how mechanical and molecular cues regulate MΦ behavior may offer novel avenues for immunomodulatory and regenerative therapies.

**References:** [1] Schlundt+ Acta Biomat. 2021, [2] Kegelman+ FASEB J. 2018, [3] Collins+ Dev. Cell. 2024, [4] Kegelman+ JBMR. 2020, [5] Hamza+ Front. Immunol. 2023, [6] Wong+ Immunity. 2021, [7] Hallström+ MBoC, 2023, [8] Jones+ PNAS. 2023. [9] Mason+ JCB 2019.

### Diurnal Glucocorticoid Rhythms play a Critical Role in the Development of Osteoarthritis During Chronic Disruption of Circadian Rhythms

*Eugenie Macfarlane, PhD[1], Lauryn Cavanagh, BMedSci[1], Colette Fong-Yee, MS[1], Joseph Tannous, B.Sci[1], Markus J. Seibel, MD PhD FRACP FAHMS[1], and Hong Zhou MD PhD[1].*
*[1]ANZAC Research Institute, The University of Sydney, Concord, NSW, Australia*

Chronic disruption of circadian rhythms from shift work increases the risk of osteoarthritis (OA). However, the underlying mechanisms that drive joint degeneration from shift work are unknown. Endogenous glucocorticoid secretion follows a diurnal rhythm and is known to be a potent regulator of circadian rhythms by synchronizing the cellular clocks throughout the body. We therefore asked whether glucocorticoid signaling in chondrocytes mediates the effects of environmental circadian rhythm disruption during OA development in wild-type (WT) and tamoxifen-inducible chondrocyte glucocorticoid receptor knockout mice.

Eight-week-old male Col2a1Cre$^{T2}$/GR$^{flox/flox}$ (GRKO) mice and their WT (GR$^{flox/flox}$) littermates were exposed to an established model of chronic circadian rhythm disruption for 22 weeks. Mice were maintained on either a normal 12:12hr light-dark cycle (non-shifted) or exposed to weekly 12hr phase-shifts, equivalent to spending alternate weeks in America and Australia (shifted; Fig.1A). Animal behaviour and activity was measured using the Promethion Metabolic Cage System (Sable Systems International). Shifted mice displayed pronounced disruption to sleep-wake cycles and feeding patterns, and were more active during normal periods of rest compared to non-shifted mice (Fig. 1B).

Chronic disruption of circadian rhythm abolished the diurnal rhythmicity of circulating glucocorticoids, characterized by a loss in the normal daily peak of serum corticosterone (upon awakening) in all shifted mice (Fig. 1C). Overall 24-hour concentrations of serum corticosterone were no different between groups. Consequently, rhythmic expression of the major clock gene *Bmal1* was abrogated in femoral cartilage tissue of WT shifted mice. However, in GRKO shifted mice cartilaginous *Bmal1* expression remained rhythmic, although in opposite phase to non-shifted animals. This indicates that blocking arrhythmic chondrocytic GC signaling allows a self-sustaining rhythm in *Bmal1* expression to persist in cartilage tissue (Fig.1D).

Histological analysis revealed that chronic disruption of circadian rhythm resulted in knee joint cartilage degradation in WT mice, but not in GRKO mice (Fig. 1E). To further investigate the effects of chronic circadian rhythm disruption on joint health we studied the progression of posttraumatic OA by destabilization of the medial meniscus (DMM) four weeks prior to harvest. In WT mice, chronic disruption of circadian rhythm accelerated cartilage degradation, subchondral bone sclerosis, and induced synovial mast cell infiltration during the early stages of posttraumatic OA. These features were significantly less pronounced in GRKO mice (Fig.1E), indicating that glucocorticoid signalling in chondrocytes drives cartilage damage, subchondral bone sclerosis and mast cell recruitment.

Our results, for the first time, provide compelling in vivo evidence that glucocorticoid signaling in chondrocytes is central to the development and progression of posttraumatic OA during chronic disruption of circadian rhythm. These novel findings provide valuable insight into how chronic disruption of circadian rhythm drives detrimental effects on skeletal health in shift workers.

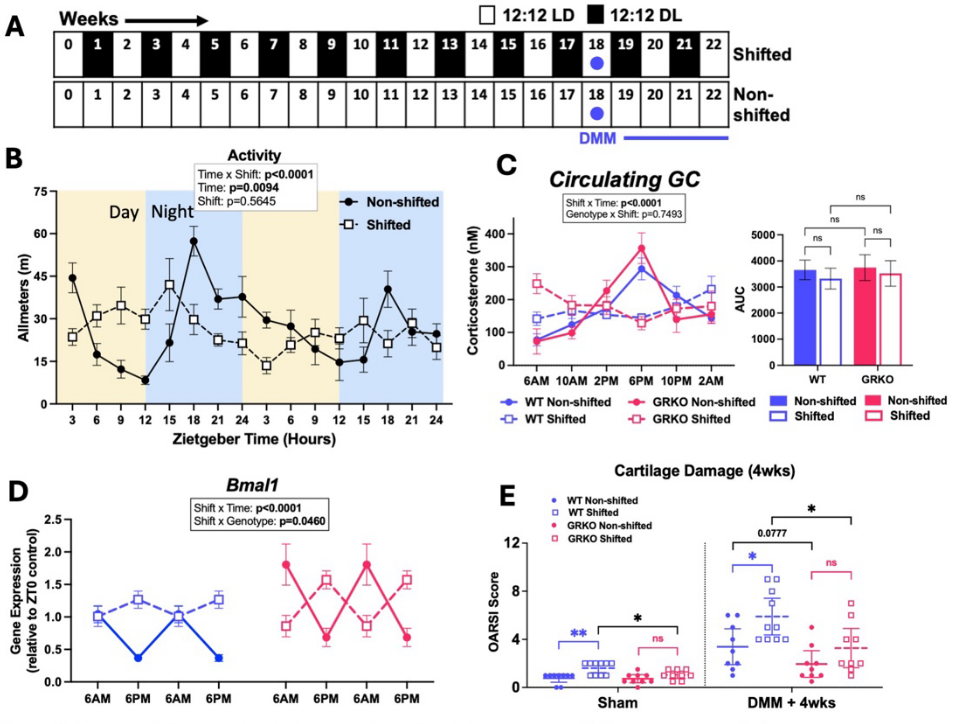

**Figure 1.** Phenotype induced by chronic disruption of circadian rhythm in wild-type (WT) and chondrocyte (ch) glucocorticoid receptor knockout (GRKO) mice. Destabilization of the medial meniscus (DMM) was performed at 4 weeks prior to harvest in shifted and non-shifted mice. GC, glucocorticoid; Zietgeber time: defined as 0 = 'Lights on', 12 = 'Lights off'; Bmal, brain and muscle ARNT-Like protein; LD, Light:Dark. OARSI, Osteoarthritis Research Society International; AUC, Area under curve (for overall GC concentration analysis). Analysed by 2- or 3-way ANOVA, n=4-10/group.

### Exercise Restores Bone Matrix Quality in Aged Mice via Molecular and Nanoscale Material Adaptations

*Nadja Michelle Maldonado Luna[1,2], Charles August Schurman, PhD[3], Eric Schiable[4], Serra Kaya, PhD[1], Turan Aghayev, PhD[5], Gregor Bieri, PhD[5], Birgit Schilling, PhD[3], David H. Kohn[6], Saul Villeda, PhD[5], Tamara N. Alliston, PhD[7]*

*[1]Department of Orthopaedic Surgery, University of California, San Francisco, CA, [2]Department of Bioengineering, UC Berkeley/UCSF, San Francisco, CA, [3]Buck Institute for Research on Aging, Novato CA, [4]Materials Sciences Division, Lawrence Berkeley National Laboratory, Berkeley, CA, [5]Department of Anatomy, University of California, San Francisco, CA, [6]Departments of Biologic and Materials Sciences and Biomedical Engineering, University of Michigan Ann Arbor, MI, [7]National Institute of Arthritis and Musculoskeletal and Skin Diseases, National Institutes of Health, Bethesda, Bethesda, MD*

Bone fragility presents major challenges to the aging population, limiting mobility and signaling a decline in systemic health. Current therapies for bone fragility primarily target low bone mineral density (BMD), yet nearly half of fractures occur in individuals with impaired bone quality (BQ) rather than low BMD[1,2]. BQ encompasses factors such as bone matrix composition, structure, and material properties that contribute to bone's mechanical behavior. Osteocytes play a central role in maintaining BQ, not only by regulating osteoblast and osteoclast activity, but also through local perilacunar/canalicular remodeling (PLR), where they resorb and replace extracellular matrix (ECM) to preserve tissue integrity[3–5]. With aging, PLR is suppressed, leading to degeneration of the lacunar/canalicular network, loss of collagen organization, increased local mineralization, and diminished ability of collagen to carry tissue strain, all of which contribute to increased bone fragility[6,7]. Despite advances in our understanding, it remains unclear whether BQ and PLR can be therapeutically targeted to mitigate age-related fragility. In particular, the potential for interventions such as exercise to restore these processes is not well established. To address this gap, we investigated whether short-term exercise can reverse age-related deterioration in bone matrix properties and studied its associated molecular signatures.

Young (3-month-old) and aged (19-month-old) male C57BL/6 mice (n=10/group) completed 21 days of voluntary wheel running or remained sedentary. Bone matrix properties were assessed using synchrotron-based small and wide-angle X-ray scattering during tensile testing, providing nanoscale insights into mineral and collagen organization. As expected, aged sedentary mice exhibited marked deficits in the ability of both collagen and mineral to carry tissue strain compared to young controls. Notably, exercise restored collagen and mineral strain capacity in aged mice by 1.9-fold and 1.7-fold ($p=0.0001$), respectively, achieving values comparable to young controls. To identify molecular drivers of this restoration, we performed transcriptomic and proteomic analyses, which revealed that exercise reverses age-related suppression of TGFβ signaling, a pathway critical for ECM composition and mineralization, and reactivated collagen cross-linking regulators (LOX, P4Ha1) and osteocyte function (DMP1, MEPE). In addition to these matrix-related pathways, exercise also upregulated oxidative stress markers Sirt2 and SOD1, suggesting that exercise may confer protection against oxidative damage, to preserve osteocyte PLR activity and bone quality in aged bone (figure 1).

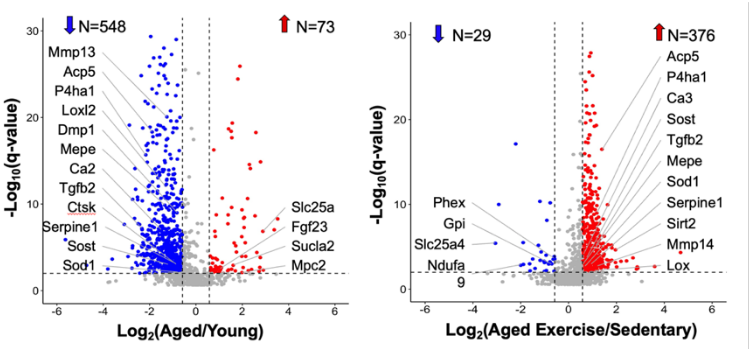

**Figure 1. Bone proteomics analyses of aging and exercise mouse studies.** Exercise regulates bone ECM composition and protein expression and suggests a rescue of bone formation lost with age after 21 days of exercise.

Our findings provide preliminary evidence that exercise may counteract age-related bone deterioration by restoring key molecular pathways and nanoscale material properties linked to bone quality and perilacunar remodeling as early as 21 days post-exercise and highlight important mechanistic targets for future investigations into bone rejuvenation.

References: [1]Nguyen, J Bone Miner Res, 2005, 1921; [2]Wainwright, J Clin Endocrinol Metab, 2005, 2787; [3]Dole, Cell Rep, 2017, 2585; [4]Bonewald, J Bone Miner Res, 2011, 229; [5]Heveran, Curr Osteoporos Rep, 2023, 11; [6]Schurman, Proc Natl Acad Sci USA, 2021, 118; [7]Schurman, Bone Res, 2024.

### Time-lapse HR-pQCT Reliably Assesses and Monitors Compartment-specific Bone Turnover in Patients with Chronic Kidney Disease

*Minhao Zhou, PhD[1], Saghi Sadoughi, PhD[1], Lauren Go[1], Gabriella Ramil[1], Isabel Yu[1], Isra Saeed, MD[1], Bo Fan, MD[1], Po-hung Wu, PhD[1], Isidro B. Salusky, MD[2], Thomas L. Nickolas, MD[3], Joachim H. Ix, MD[4], Galateia J. Kazakia, PhD[1]*
*[1]University of California-San Francisco, CA, USA, [2]University of California-Los Angeles, CA, USA, [3]Washington University in St. Louis, MO, USA, [4]University of California-San Diego, CA, USA*

***Introduction:*** Chronic kidney disease (CKD) is a global health concern affecting ~40% of the elderly population. CKD-associated osteoporosis is a common CKD complication. Patients with CKD-associated osteoporosis experience a 2-to-6 times higher risk of hip fractures, with a 4-to-6 times higher mortality, compared to non-CKD individuals.[1] CKD-associated osteoporosis largely results from high or low turnover abnormalities, which benefit from therapeutic strategies informed by patient-specific turnover assessment to increase or suppress turnover. However, due to limitations of current clinical turnover assessment (e.g., double-labeled iliac crest biopsy with quantitative histomorphometry), a reliable, accessible, and non-invasive turnover assessment remains an unmet clinical need. High-resolution peripheral quantitative computed tomography (HR-pQCT) provides the highest in vivo resolution for bone microarchitecture. By registering sequential scans, time-lapse (TL) analysis evaluates temporospatial bone gain/loss, which may reflect turnover status if the duration between scans does not exceed a full bone remodeling cycle (**Fig. A**). Thus, this work aimed to evaluate the feasibility of time-lapse HR-pQCT ('TL HR-pQCT') in CKD bone care. We hypothesized that a 2-month TL period could capture turnover in patients with CKD.

***Methods:*** A TL HR-pQCT pipeline was developed and optimized using ex vivo cadaveric phantom scan-rescans (*n*=26) and in vivo scan-rescans from healthy volunteers (*n*=20, **Fig. B**).[3] Least significant change in turnover (LSC) was assessed using in vivo scans acquired one year apart from the healthy volunteers (*n*=15). Eight patients with end-stage renal disease (ESRD) underwent three baseline and one 2-month follow-up scans. All distal tibia and radius scans were acquired on XtremeCT II. Scans with a motion grade of 1/2 were analyzed. Primary turnover metrics (volumetric bone formation and resorption rate, vBFR and vBRR) were evaluated with respect to baseline integral, cortical, and trabecular bone volumes. Derivative turnover metrics (total and net bone turnover, TBT and NBT) were calculated as vBFR +/- vBRR, respectively (**Fig. B**). Turnover metrics and LSC were annualized (%/year).

***Results:*** After excluding high-motion scans, 39 tibia and 24 radius healthy scan pairs, as well as 24 tibia and 18 radius ESRD scan pairs, were analyzed. At the tibia, LSC ranged from 0.9% to 4.7% per year. At the radius, LSC ranged from 1.7% to 8.2% per year. For the ESRD cohort, all primary bone formation and resorption metrics over the 2-month period were on average 3- and 6-fold higher than the respective LSC at the tibia and radius, respectively (**Fig. C**). Significant net cortical bone loss (-10.6%/year) was observed at the radius (**Fig. D**).

***Discussion:*** This study showed that TL HR-pQCT captured compartment-specific bone turnover within the ESRD cohort over a 2-month period, equal to or shorter than that required for clinical bone biopsy histomorphometry. The pipeline also accurately described the rapid cortical bone loss documented for ESRD.[4] Furthermore, the pipeline captured volumetric bone turnover consistent with gold standard bone biopsy histomorphometry data (**Fig. E**). While the pipeline requires further validation (e.g., patients with earlier-stage CKD), TL HR-pQCT has shown promise to non-invasively inform bone turnover status for CKD-associated osteoporosis management.

***REFERENCES:*** [1] Kim et al., 2016; [2] Christen & Müller, 2017; [3] Zhou et al., 2025; [4] Nicolas et al., 2013

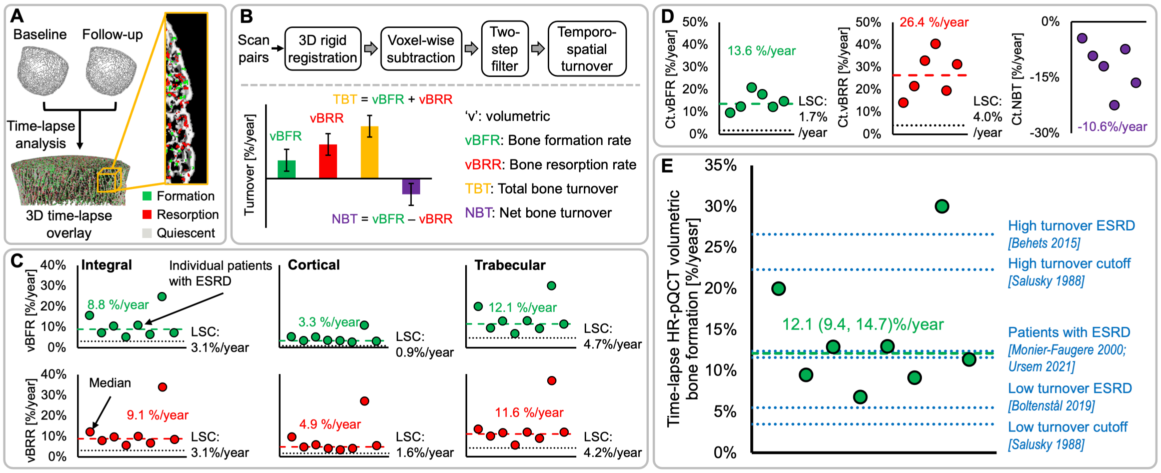

**Fig: (A)** Representative distal tibia scan pairs visualizing TL HR-pQCT bone formation and resorption assessment. **(B)** Representative TL HR-pQCT pipeline (top), with primary and derivative turnover metrics (bottom). **(C)** TL HR-pQCT turnover metrics assessed at the distal tibia, presented as median in patients with ESRD vs. least significant change (LSC). **(D)** TL HR-pQCT turnover metrics evaluated at the distal radius, highlighting the net cortical bone (Ct.) loss with ESRD. **(E)** TL HR-pQCT bone formation data agreeing with historic bone formation ranges via bone biopsy histomorphometry reported for ESRD.

### Bone Quality assessment via Impact Microindentation Predicts Fracture Susceptibility in Aging Females

*Rachana S. Vaidya, Ph.D.[1], Babak Jahani, Ph.D.[1], James M. Jin, M.S[1], Christian C. Gonzalez, M.S[2], Bhanu Pujari, B.S[3], Siva Krothapalli, B.S[3], Simon Y. Tang, Ph.D.[1,2]*

*[1]Department of Orthopaedic Surgery, Washington University School of Medicine in St. Louis, MO 63110, USA , [2]Department of Biomedical Engineering, Washington University School of Medicine in St. Louis, MO 63130, USA, [3]School of Medicine, St Louis University, MO 63104, United States.*

### Introduction:

Fragility fractures are a major cause of morbidity in aging female population and often occur in individuals with normal or mildly reduced bone mineral density (BMD)[1]. While dual-energy X-ray absorptiometry (DXA) has been the clinical standard for estimating BMD and fracture risk, its limitations are well recognized[2,3]. Fracture is fundamentally a mechanical event that occurs when applied loads exceed the structural and material strength of bone[4]. While tools like FRAX and trabecular bone score (TBS) have improved fracture risk assessment by incorporating clinical risk factors and estimating trabecular structure, they do not directly assess the biomechanical integrity of bone tissue[5]. Bone strength depends not only on bone mass but also on bone quality. Bone quality is a multifaceted concept that includes the material and structural features contributing to fracture resistance, which are influenced by underlying bone remodeling and metabolic processes[6]. With age and metabolic diseases, the regulation of these processes becomes impaired, leading to deterioration in bone quality that is not captured by BMD alone[7]. Despite advances in understanding the biological and mechanical basis of skeletal fragility, the lack of clinical tools that directly assess bone material quality remains a barrier to accurate fracture risk assessment.

To address the need for direct assessment of bone quality, Impact Microindentation (IMI) has emerged as a promising minimally invasive technique that quantifies cortical bone's resistance to localized deformation[8]. Clinically, IMI is performed at the tibial midshaft using the FDA-cleared OsteoProbe® device[8]. The Bone Material Strength index (BMSi) values, derived from IMI have shown associations with bone fragility across diverse metabolic conditions, with lower BMSi values linked with increased risk and severity of osteoporotic fractures, independent of BMD[9]. Yet, whether IMI can reliably distinguish individuals who are susceptible to fracture remains an open and clinically important question for translating IMI into clinical practice as a fracture risk assessment tool. Furthermore, the specific bone quality features detected by IMI remains unclear, making its interpretation less intuitive than that of BMD, which directly quantifies mineral content.

In this study, we aim to determine whether IMI captures tissue-level deficits in bone quality such as collagen integrity, microstructure, material properties and fracture toughness. We then assess how bone quality, assessed by IMI at the tibia, correlates with whole-bone strength across clinically relevant skeletal sites, both independently of, and in combination with, BMD measured by DXA. Finally, we explore the utility of IMI in subject-specific fall-force models to assess its potential for predicting individual fracture susceptibility, linking tissue-level deterioration to functional skeletal failure. We hypothesize that IMI measurement at the tibia reflects biomechanical deterioration at the tissue, whole-bone and individual level, providing insights that could improve early identification of high-risk individuals and inform targeted interventions to preserve bone quality. To investigate the biomechanical and age-related changes in bone quality that may contribute to skeletal fragility, we utilized human cadaveric bone specimens from aging female donors, a clinically relevant cohort for studying skeletal fragility in this high-risk population.

**Methods:** We conducted a cross-sectional study using human skeletal specimens from aging female donors (mean age, 71.5yo, 95%CI:68.3-74.7), including 67 femurs, 54 radii, 53 L4 vertebrae and 67 tibiae. Donors with orthopaedic hardware, prior fractures, or bone diseases were excluded. To evaluate whether impact IMI can detect meaningful changes in bone quality and estimate mechanical competence, we prepared cortical bone beams (2x2x30mm) from femur midshaft. IMI was performed on these beams (Beam IMI) to assess its relationship with bone material properties (3-point bending tests), cortical porosity and tissue mineral density (microCT), and organic matrix composition (denatured collagen and advanced glycation end-products (AGEs) accumulation assays). To assess whether IMI reflects fracture toughness, we performed 3-point bending on notched tibial beams to calculate crack initiation toughness($K_{IC}$), a direct measure of fracture toughness. We then compared $K_{IC}$ values to Beam IMI measurements using correlation and Bland–Altman analysis to assess both the strength of association and the level of agreement between these two mechanical metrics.

To assess if bone quality, assessed by IMI at the tibia midshaft reflects systemic skeletal strength, mechanical testing was conducted on femur, radii and vertebrae specimen to measure failure load(N), and IMI was performed on the tibiae specimens. We analyzed correlations between tibial IMI and whole-bone strength at other skeletal sites, both independently and in combination with BMD measured by DXA. To evaluate the utility of bone quality assessment using IMI in settings where BMD is insufficient, we stratified the dataset by BMD-defined T-score categories (osteoporotic, osteopenic, normal), and analyzed correlations between IMI measurements and femur strength within each subgroup.

Finally, to evaluate the utility of bone quality assessment with IMI in predicting fracture susceptibility in individuals, we used biomechanical models to estimate the forces experienced by specimen donors during a sideways fall onto the hip and a forward fall onto the wrist from standing height, using individual-specific anthropometric data (height, weight, BMI). The estimated fall forces were compared to laboratory-measured failure loads of corresponding bone specimens; Donors were classified as fracture-susceptible if the estimated fall force exceeded their measured bone strength. Using receiver operating characteristic (ROC) curve analysis, we compared how well IMI and Hip T-scores distinguished between donors classified as susceptible to fracture and those who were not.

**Results**: At the tissue-level, IMI is strongly correlated to bone material properties such as ultimate stress (r=0.61, 95%CI:0.42-0.74,p<0.001) and elastic modulus (r=0.44, 95%CI:0.22-0.62,p=0.002). IMI is also negatively correlated with organic matrix composition markers including the percentage of denatured collagen (r=-0.50, 95%CI:-0.66-0.29, p<0.001), advanced glycation end products (AGEs) (r=-0.25, 95%CI:-0.47-0.08, p=0.03) and microstructural property such as cortical porosity (r= -0.52, 95%CI:-0.68--0.31, p<0.001) and positively correlated to tissue mineral density (r= 0.28,95%CI:0.03-0.49, p=0.02) **(Fig1A).** IMI is strongly correlated with crack initiation toughness ($K_{IC}$) at the tibia (r=0.86, 95%CI:0.65-0.94, p<0.001). Bland-Altman analysis confirms close agreement between IMI and $K_{IC}$, with no systemic bias (Bias=0, SD bias=11%, 95%CI = +/-21%)(**Fig1B**).

At the whole bone level, total hip BMD (TH-BMD) was correlated with femur strength (r = 0.54, 95% CI: 0.34–0.69, p < 0.001, $R^2$ = 0.29) and radius strength (r = 0.54, 95% CI: 0.31–0.71, p < 0.001, $R^2$ = 0.29), but L4-BMD did not significantly correlate with vertebral strength (r = 0.26, 95% CI:-0.009–0.5, p = 0.06, $R^2$ = 0.07)**.** However, IMI, representing tissue-level bone quality, showed stronger and significant correlations with whole-bone strength at the femur (r = 0.66, 95% CI: 0.51–0.78, p < 0.001, $R^2$ = 0.44), radius (r = 0.55, 95% CI: 0.34–0.72, p < 0.001, $R^2$ = 0.31), and vertebrae (r = 0.31, 95% CI: 0.03–0.53, p = 0.03, $R^2$ = 0.09), compared to BMD. Including IMI with BMD in a regression model significantly enhanced the association with whole-bone strength compared to either measure alone. At the femur, combining both variables increased the explained variance in strength by 83%, compared to BMD alone (r = 0.73, 95% CI: 0.59–0.83, p < 0.001, $R^2$ = 0.53). Similarly, at the radius, the combined model improved the association by 41%, compared to BMD alone (r = 0.64, 95% CI: 0.45–0.77, p < 0.001, $R^2$ = 0.41). At the vertebrae, because BMD alone was not correlated to strength, IMI alone remained the significant predictor (r = 0.31, 95% CI: 0.03–0.53, p = 0.03, $R^2$ = 0.09) **(Fig1C)**. When femur specimens were stratified based on Hip T-scores into osteoporosis, osteopenic, and normal groups, IMI remained significantly correlated with bone strength, where BMD did not. In the osteoporotic group (T-score <-2.5), IMI was more strongly correlated with femur strength (r = 0.69, 95% CI: 0.42–0.84, p < 0.001) than BMD (r = 0.53, 95% CI: 0.18–0.75, p = 0.004). In the osteopenic group (-2.5 < T-score <-1.0), only IMI was significantly correlated with bone strength (r = 0.47, 95% CI: 0.29–0.73, p = 0.017), while BMD showed no significant correlation (r = 0.31, 95% CI:-0.09–0.62, p = 0.13). In the normal BMD group (T-score >-1), IMI remained significantly associated with femur strength (r = 0.66, 95% CI: 0.19–0.88, p = 0.01), whereas BMD showed no correlation (r =-0.16, 95% CI:-0.65–0.42, p = 0.59) **(Fig1D)**.

At the individual level, BMSi derived from IMI demonstrated superior discriminative ability compared to Hip T-scores in classifying donors as fracture susceptible or not, under simulated fall conditions. Based on biomechanical modeling, 33 donors were classified as hip fracture-susceptible and 33 as non-susceptible; For wrist fractures, 42 were susceptible and 14 non-susceptible. At the hip, IMI achieved higher discriminative accuracy (Area under the curve (AUC) = 0.75, 95% CI: 0.63–0.88) than Hip T-scores (AUC = 0.61, 95% CI: 0.47–0.75). At the wrist, both BMSi (IMI, AUC = 0.77, 95% CI: 0.64–0.91) and Hip T-scores (AUC = 0.78, 95% CI: 0.65–0.91) performed similarly. These findings suggest that bone quality assessment by IMI provides additional predictive value for fracture risk, particularly at the hip, beyond what is captured by DXA alone **(Fig1E)**.

**Conclusion:** This study demonstrates that IMI captures meaningful changes in bone tissue quality, including alterations in material properties, microstructure, and organic matrix integrity. Notably, the strong agreement between IMI and fracture toughness ($K_{IC}$) suggests that IMI may serve a surrogate measure of bone's resistance to fracture. IMI showed strong associations with whole-bone strength across skeletal sites of clinical importance, and remained informative across T-score categories, including specimens with normal or osteopenic T-scores, where DXA alone was less predictive. Moreover, IMI demonstrated higher accuracy in classification of individual's susceptibility to fractures under simulated fall conditions, particularly at the hip, outperforming T-scores.

**Clinical relevance:** These findings support the incorporation of tibial IMI into clinical assessments of fracture risk. IMI offers a minimally invasive, site-independent method for evaluating bone quality, and provides complementary information to DXA, particularly for individuals whose fracture risk is underestimated by BMD. These results lay the groundwork for our future work focused on integrating site-specific bone quality assessments into clinical workflows to guide surgical planning.

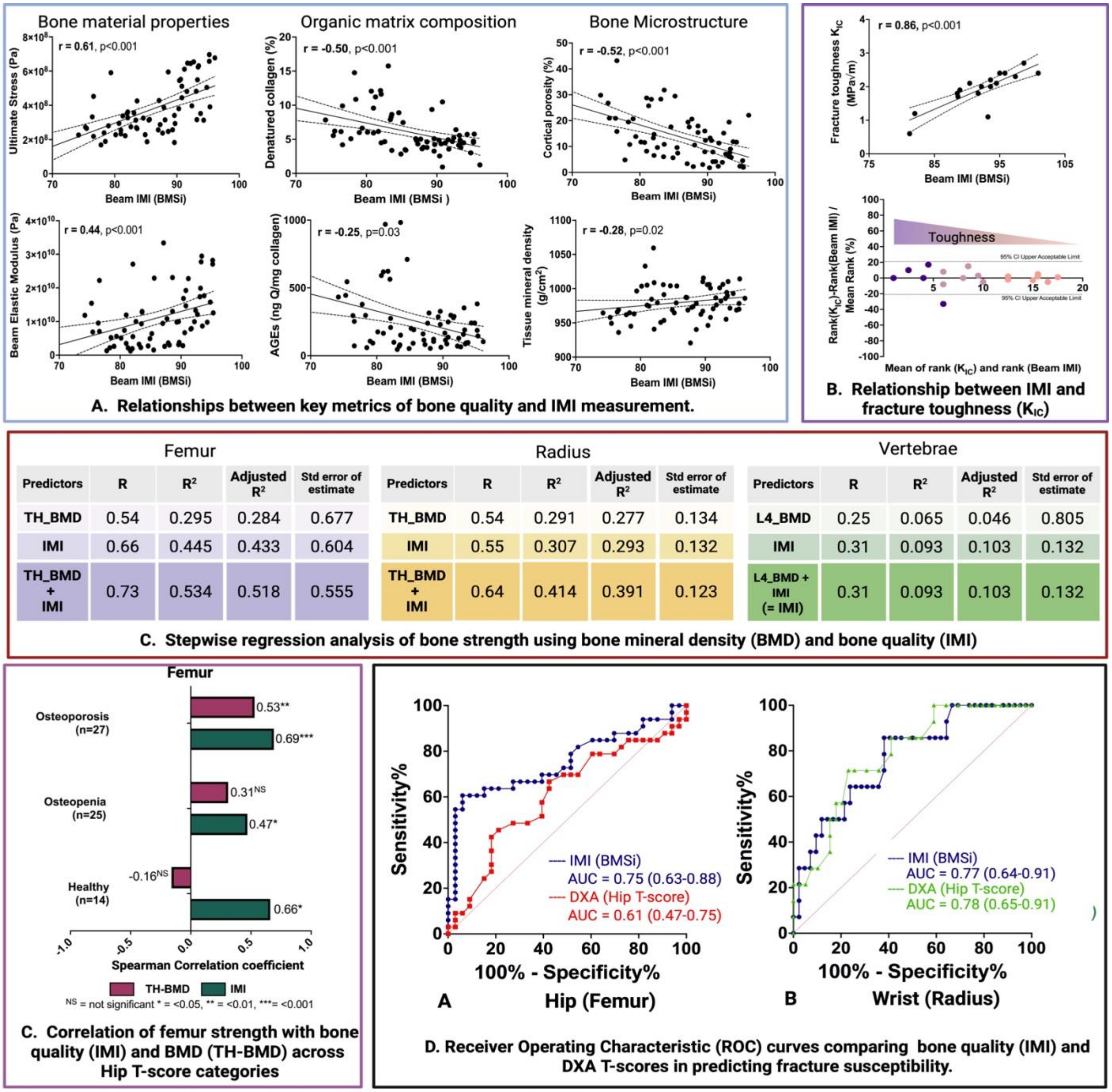

**Femur**

| Predictors | R | $R^2$ | Adjusted $R^2$ | Std error of estimate |
|---|---|---|---|---|
| TH_BMD | 0.54 | 0.295 | 0.284 | 0.677 |
| IMI | 0.66 | 0.445 | 0.433 | 0.604 |
| TH_BMD + IMI | 0.73 | 0.534 | 0.518 | 0.555 |

**Radius**

| Predictors | R | $R^2$ | Adjusted $R^2$ | Std error of estimate |
|---|---|---|---|---|
| TH_BMD | 0.54 | 0.291 | 0.277 | 0.134 |
| IMI | 0.55 | 0.307 | 0.293 | 0.132 |
| TH_BMD + IMI | 0.64 | 0.414 | 0.391 | 0.123 |

**Vertebrae**

| Predictors | R | $R^2$ | Adjusted $R^2$ | Std error of estimate |
|---|---|---|---|---|
| L4_BMD | 0.25 | 0.065 | 0.046 | 0.805 |
| IMI | 0.31 | 0.093 | 0.103 | 0.132 |
| L4_BMD + IMI (= IMI) | 0.31 | 0.093 | 0.103 | 0.132 |

**FIGURE 1**

**References:** [1]Leali PT, et al. Clin Cases Miner Bone Metab. 2011. [2]Hart NH, et al. J Musculoskelet Neuronal Interact. 2017.[3]Susan MO. Kidney Int. 2016.[4]Cole JH, van der Meulen MC. Clin Orthop Relat Res. 2011. [5]Palomo T. Arch Endocrinol Metab. 2022. [6]Donnelly E. Clin Orthop Relat Res. 2011. [7]Feng X, McDonald JM. Annu Rev Pathol. 2011. [8]Bridges D. Rev Sci Instrum. 2012.[9]Schoeb M, et al. Osteoporos Int. 2022.

### Complex Sphingolipids Accumulate in Skeletal Muscles Undergoing Wasting in Ageing and Cachexia – Evidence from Murine Models of Frailty-Coupled Sarcopenia and Cisplatin-Induced Cachexia

*Manja M. Zec, PhD[1], Justin M. Snider, PhD[2], Mladen Jergović, PhD[3], Laurel Johnstone, PhD[2], Nicholas Jamnick, PhD[4], Joshua R. Huot, PhD[5], Fabrizio Pin, PhD[5], Janko Ž. Nikolich, MD, PhD[3], Andrea Bonetto, PhD[4], Michael Zuscik, PhD[1]*

*[1]Colorado Program for Musculoskeletal Research, Department of Orthopaedics, University of Colorado Anschutz Medical Campus, Aurora, CO, USA [2]School of Nutritional Sciences and Wellness, University of Arizona, Tucson, AZ, USA [3]Department of Immunobiology, University of Arizona Center on Aging, University of Arizona College of Medicine-Tucson, Tucson, AZ, USA [4]Department of Pathology, University of Colorado Anschutz Medical Campus, Aurora, CO, USA; [5]Department of Anatomy, Cell Biology and Physiology, Indiana University School of Medicine, Indianapolis, IN, USA.*

**Introduction.** Skeletal muscle (SKM) dysfunction and weight loss occur in chronic and age-related diseases, dramatically impairing quality of life. The mechanistic and metabolic reprogramming, including lipid rearrangements, linked with the SKM functional decline, is unknown. **Methods.** Two studies were conducted in male C57BL/6 mice. A genetically inducible model of frailty (FRA), i.e., accelerated ageing, was validated by inducing high systemic Interleukin-6 levels in adult animals through the 5-day food-based administration of doxycycline [1]. FRA mice experienced 30% wasting in the gastrocnemius, but not the soleus [1]. Next, a model of chemotherapy-induced SKM dysfunction (i.e., cachexia; CAC) was carried over by administering cisplatin to young (2 months) and old (18 months) mice, either wild type or genetically modified to overexpress PGC1$\alpha$ in SKM, the latter counteracting cisplatin-induced SKM wasting and neuromuscular defects in the quadriceps of old mice [2]. Using LC-MS/MS, we applied untargeted and quantitative lipidomics in the SKM and other metabolic tissues. The experiments were approved by the IACUCs at the UA and IU. Computational analyses were done using R packages, MetaboAnalyst 5.0, and LipidSuite 1.0. **Results.** In FRA animals, we saw 1225 lipid events belonging to 25 lipid classes in the sarcopenic gastrocnemius. Only in the gastrocnemius (but not soleus), untargeted lipidomics indicated 2-fold changes in ceramides and di- and triglycerides, compared to the adult controls. Quantitative analyses confirmed the accumulation of complex sphingolipids, specifically hexosylceramides, in gastrocnemius, but not soleus (Upper Panel), driven by C16-, C18-, C24-, C24:1-glucosyl, and C16-, C18-, and C24:1-lactosyl features only in the FRA group. Lipid shifts in plasma and subcutaneous adipose tissue paralleled gastrocnemius and soleus, respectively. Quantitative enrichment analyses (QEA) in plasma confirmed sphingolipid metabolism as 3rd most enriched pathway in FRA. Next, in the CAC study, the discovery approach in cachectic quadriceps revealed 233 lipid features belonging to carnitines, sphingolipids, cardiolipin, and phosphatidic acid classes. Lipidomic fingerprints showed the upregulation of hexosylceramides in cisplatin-induced cachexia in old animals only; however, the trend was reversed in the presence of PGC1$\alpha$ (Lower Panel). The QEA showed $\beta$-oxidation as the 4th most enriched pathway, also in the presence of PGC1$\alpha$, indicating the role of mitochondrial biogenesis in intracellular protection in cachexia. All the findings were present in the quadriceps, but not plasma, pointing out SKM-specific regulation in cisplatin-induced cachexia. **Conclusion.** Intramyocellular hexosylceramides-driven lipid accumulation in the gastrocnemius in accelerated ageing, and quadriceps in cisplatin-induced wasting, underpins SKM wasting in frailty and cachexia. Reportedly, FRA animals experienced higher PGC1$\alpha$ levels in the sarcopenic gastrocnemius [1], the same mitochondrial regulator that acts protectively in CAC [2], indicative of mitochondrial adaptive reparative mechanisms in lipotoxicity-driven SKM wasting. **Clinical Application.** Accumulation of hexosylceramides might be a lipotoxic mechanism underlying SKM wasting in frailty-linked sarcopenia and cisplatin-induced cachexia, similar to what is already proposed in age-related sarcopenia [3]. Pharmacological inhibition of hexosylceramide synthase by eliglustat/miglustat will be tested for alleviating SKM wasting in chronic disease.

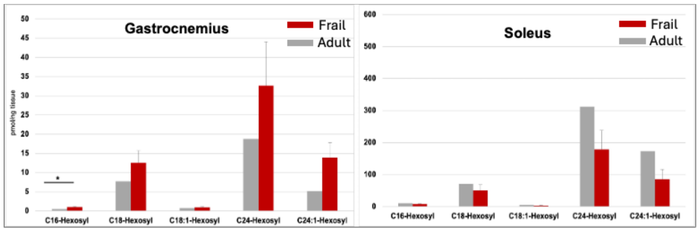

**FRA, Upper Panel**: Individual hexosylceramides accumulate in the inflammation-driven sarcopenic gastrocnemius (left), but not the intact soleus (right).

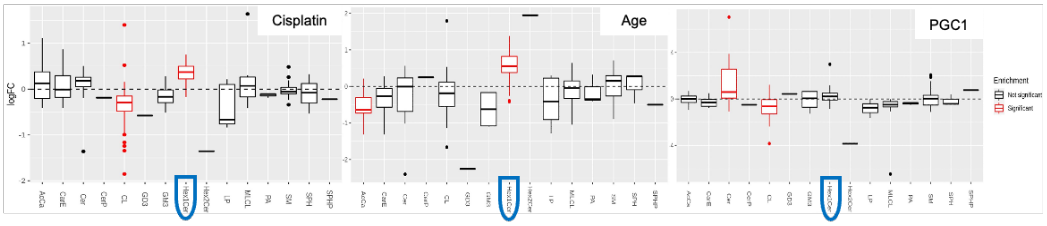

**CAC, Lower Panel**: Lipidomic fingerprints reveal accrual of hexosylceramides (circled in blue) in cisplatin-induced cachexic quadriceps in old mice (left), which is stronger in comparison with young mice (middle), and is reversed in old mice upon the induction of PGC1 (right). The negative and positive directions indicate the total number of down- and upregulated features within a lipid subclass.

**References.** [1] Jergović, M et al. (2021). IL-6 can single-handedly drive many features of frailty in mice. Gerosc, 43, 539-549. [2] Huot et al. (2022). PGC1α overexpression preserves muscle mass and function in cisplatin-induced cachexia. J Cachexia Sarcopenia Muscle, 13(5), 2480-2491. [3] Laurila PP, et al. (2022). Sphingolipids accumulate in aged muscle, and their reduction counteracts sarcopenia. Nat Aging, 2(12), 1159-1175.

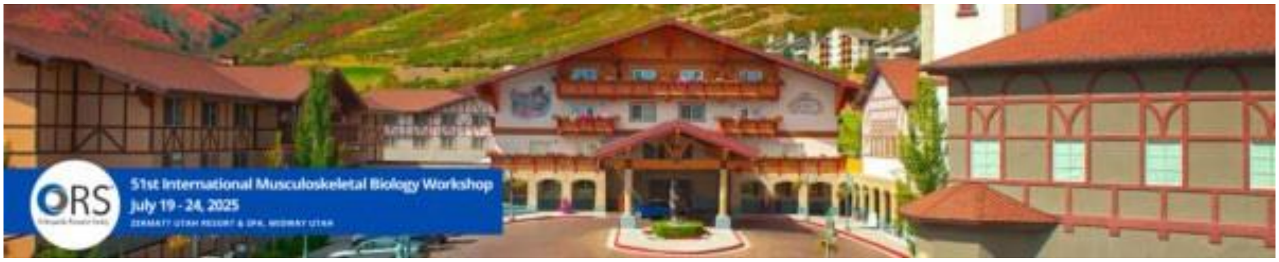

### WORKSHOP ABSTRACTS

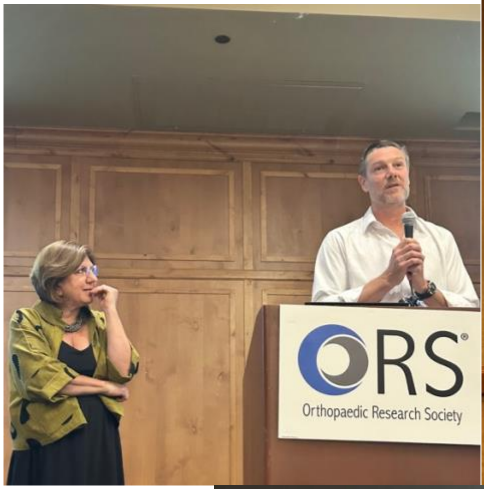

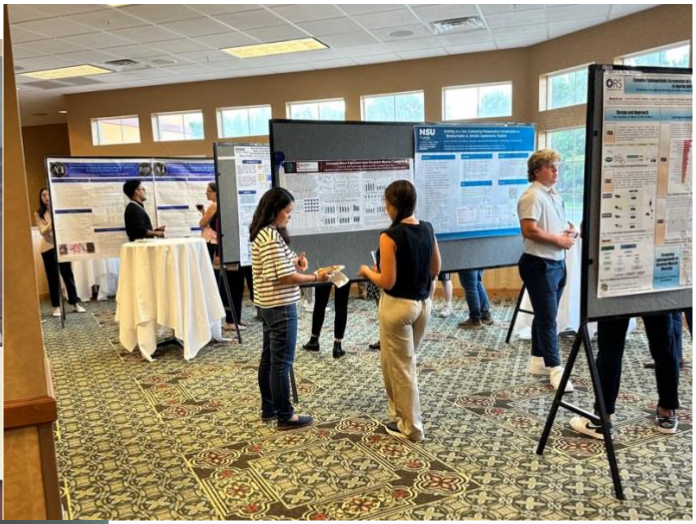

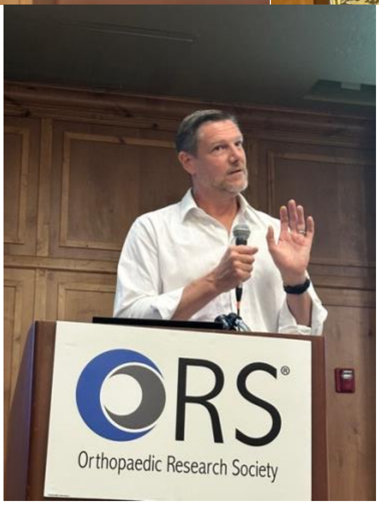

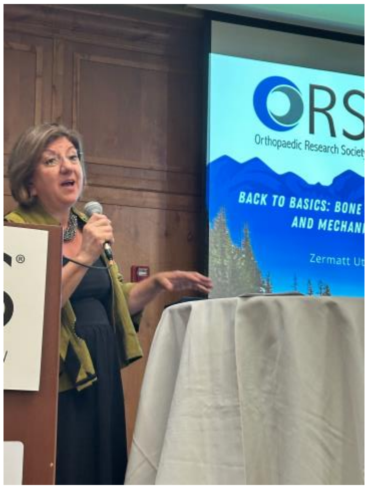

**Osteocytes drive bone gain, but not the increase in bone remodeling, induced by anabolic ligands of the PTH receptor in physiological and diabetic conditions.**

*Nisreen Akel[1,2], Sasidhar Uppuganti[3], Wyatt House[1], Gretel G Pellegrini[1,4], Joan Pizarro-Gómez[1,5], Shenyang Li[2], Meloney Cregor[1,2], Silvia Marino[1,2], Jeffry S. Nyman[3,6], Teresita Bellido[1,2,7]*

[1]*Department of Physiology and Cell Biology, University of Arkansas for Medical Sciences, Little Rock, AR, USA;*
[2]*Central Arkansas Veterans Healthcare System, John L. McClellan, Little Rock, AR, USA;*
[3]*Department of Orthopedic Surgery, Vanderbilt University Medical Center, Nashville, TN, USA;*
[4]*Institute of Immunology, Genetics & Metabolism, Laboratory of Osteopathies, University of Buenos Aires and the National Scientific and Technical Research Council, Buenos Aires, Argentina;*
[5]*Institute of Advanced Molecular Medicine, Universidad CEU San Pablo, Madrid, Spain;*
[6]*Department of Veterans Affairs, Tennessee Valley Healthcare System, Nashville, TN, USA;*
[7]*Winthrop P. Rockefeller Cancer Institute, University of Arkansas for Medical Sciences, Little Rock, AR, USA,*

Signaling downstream of the receptor of parathyroid hormone (PTH1R) exerts two major effects on the skeleton: increases bone remodeling and, when stimulated intermittently, induces bone anabolism. Osteocytes express the PTH1R ($PTH1R^{OCY}$) and are critical for the action of parathyroid hormone (PTH). However, it is unknown whether the $PTH1R^{OCY}$ also mediates the effects of the other receptor ligand abaloparatide (ABL) and whether it is required for the increase in remodeling and/or the bone gain induced by PTH/ABL in diabetes (DM). We addressed these questions by treating with anabolic regimens of PTH or ABL skeletally mature control (C) or DM mice lacking the $PTH1R^{OCY}$ (cKO) and fl/fl littermates. Both PTH and ABL increased bone mass and improved or corrected cortical and trabecular bone microarchitecture only in fl/fl but not in cKO, C or DM mice. Further, PTH/ABL increased bone strength and microindentation resistance only in fl/fl but not in cKO, C or DM mice. In contrast, PTH/ABL increased serum P1NP and bone formation on cancellous, periosteal and endocortical surfaces, in both fl/fl and cKO, C or DM mice. Moreover, serum CTX and osteoclast surface were elevated in DM vs C mice and further increased by PTH/ABL in C and DM mice of both genotypes. Thus, the $PTH1R^{OCY}$ is required for bone gain, microarchitecture restoration, bone strength and resistance to fracture induced by both PTH and ABL, whereas it is dispensable for their effects on bone remodeling under physiological and DM conditions. These findings demonstrate dissociation of bone gain from bone remodeling induced by PTH/ABL by deleting the PTH1R from osteocytes.

### *In Vitro* and *In Vivo* Evaluation of Fluorapatite Scaffolds seeded with Stromal Vascular Fraction for Bone Repair

Clark Nielson, MS[1,2], Jill Shea, PhD[1,2], Jay Agarwal, MD[1,2], Pooya Elahi, PhD[1,2], Sujee Jeyapalina, PhD[1,2], James P. Beck, MD[1,2]
[1]*University of Utah, Salt Lake City, UT,* [2]*Department of Veterans Affairs, Salt Lake City, UT*

**INTRODUCTION:** Due to the limitations of autografts, i.e. donor site morbidity and limited harvestable tissue volumes, a supply–demand gap exists in the availability of suitable bone substitutes for treating critical-sized bone defects (CSDs). Porous hydroxyapatite (HAp) scaffolds have been extensively studied for such applications, but have not achieved widespread clinical adoption, emphasizing the need to enhance their regenerative potential. Our previous research indicates that fluoridated HAp (fluorapatite (FAp)) promotes bone formation *in vivo*. Additionally, scaffold architecture and surface properties, which can be modified by sintering—influence bone regeneration. For example, gyroid-structured scaffolds outperform those with linear pores in both mechanical strength and bone deposition. Stromal vascular fraction (SVF), a heterogeneous cell population from adipose tissue, can be harvested intraoperatively and offers osteogenic potential without the regulatory burdens of culture-expanded therapies. This study aimed to fabricate and evaluate FAp scaffolds with gyroid architecture combined with SVF *in vitro* and in a translational sheep iliac CSD model.

**METHODS:** ***Scaffold Fabrication***: FAp scaffolds were fabricated by gel-casting within ash-free combustible 3D-printed gyroid templates and sintered at 1050 °C (T1), 1150 °C (T2), or 1250 °C (T3). Cylindrical scaffolds (10 mm × 13 mm) with 50%, 60%, or 70% porosity were sintered at T1, T2, and T3 and prepared for mechanical testing (9 groups, n = 9 per group). Separately, 2.5 mm-tall scaffolds with ~54% porosity were used for *in vitro* studies at all three temperatures. Trapezoidal scaffolds with 54% porosity and dimensions (2 cm (H) × 3 cm (top W) × 2 cm (base W) × 1 cm (D)) and ~54% porosity were fabricated for *in vivo* use and sintered at 1150 °C. ***Cell Culture Studies***: SVF was isolated from rat adipose tissue using a collagenase digestion process. Red blood cells were lysed, and cells with stem-like phenotype ($CD31^{-}$, $CD34^{+/-}$, $CD45^{-}$, $CD90^{+}$) were quantified using FACS. 150 rpm for 1 hour, transferred to fresh plates, and maintained in culture media for 1, 4, 7, 11, or 14 days. Cell viability and osteogenic differentiation were evaluated with immunohistochemical staining for osteopontin (OPN) and osteocalcin (OCN) markers. ***Animal Testing***: Bilateral trapezoidal iliac defects matching the scaffold dimensions were surgically introduced in twelve 18-month-old Rambouillet sheep. Animals were randomized (n = 6/group) to receive either autograft (positive control), no treatment (negative control), or FAp scaffold, with or without SVF. All implants were stabilized using Kirschner wires (K-wires). Post-operative care included analgesia and monitoring. At necropsy (t = 90 days), implants and surrounding tissues were harvested for histological and radiographic evaluation. **RESULTS and DISCUSSION:** Porous FAp scaffolds with gyroid architecture were successfully fabricated and tested, with pore and strut sizes ranging from 400–850 µm. Compressive strength data revealed that FAp scaffolds were comparable to human cancellous bone (5–30 MPa), except for the 60% and 70% porosity scaffolds sintered at T1. The scaffolds with ~50% porosity and 500–600 µm pore sizes were subjected to *in vitro* and *in vivo* testing. For cell culture, ~600,000 viable cells/g of adipose were isolated, with 24 ± 3.6% of SVF cells meeting stem cell criteria by FACS. At 4 days post-seeding, more cells adhered to scaffolds sintered at T3 (~30,000 cells) compared to those sintered at T1 or T2 (each <15,000 cells). By day 11, cell number increased with sintering temperature: approximately 40,000 cells on T1 scaffolds, 330,000 on T2, and 480,000 on T3 Immunofluorescence showed osteopontin expression on all scaffolds by day 4. Osteocalcin, a late osteogenic marker, was observed on disks sintered at T2 and T3 by day 7 and on all scaffolds by day 11. In summary, SVF cells adhered and proliferated more robustly on scaffolds sintered at T2 and T3, and differentiated earlier, as indicated by earlier osteocalcin protein expression. Preliminary micro-CT at 12 weeks post-surgery in the sheep iliac crest model showed incomplete regeneration in untreated defects. Autografts integrated with surrounding tissue, though voids lacking mineralized bone remained centrally, indicating bone resorption. FAp scaffolds showed peripheral bone deposition, including regions distant from the scaffold interface. Bone volume was 28±1.4% in autograft-treated defects and 21±4.7% in FAp-only scaffolds, compared to 43±3.1% in native iliac crest. Analysis of the FAp + SVF group is ongoing.

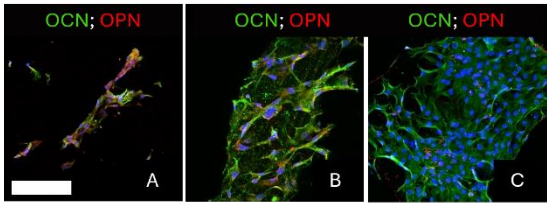

*Figure 1:* Fluorescence microscopy of SVF cells on FA scaffolds at days 7 (A), 11 (B), and 17 (C). Nuclei (blue), OPN (red, early osteogenesis), and OCN (green, late osteogenesis) are shown. OCN expression was detectable by day 7. Scale bar = 100µm.

**Conclusion:** SVF cells expressed late osteogenic markers on FAp scaffolds in vitro, and sintering temperature modulated their differentiation behavior and scaffold compressive strength. *In vivo,* early data indicate that FAp scaffolds with SVF promote bone formation in critical-sized defects, supporting their potential as a clinically translatable autograft alternative.

**Apatite-Based Coating for Improving Epidermal Integration with Percutaneous Osseointegrated Implants**

Samantha Steyl[1,2], Jill Shea[1,2], James Peter Beck[1,2], Kent Bachus[1,2], Jay Agarwal[1,2], Sujee Jeyapalina[1,2]
[1]Veteran Affairs Medical Center, Salt Lake City, UT, [2]University of Utah, Salt Lake City, UT

samantha.steyl@utah.edu

***Introduction***: Percutaneous osseointegrated (OI) prosthetic devices have emerged as promising alternatives to traditional socket-type prostheses. These systems involve the surgical placement of a titanium endoprosthesis into the intramedullary canal, with a portion of the implant extending through the skin to allow for external prosthetic attachment. Compared to socket suspension systems—which are often associated with skin irritation, poor fit, and limited wear time—, percutaneous OI devices offer improved comfort, stability, and ease of use, including faster donning and doffing and unrestricted wear duration. Despite these advantages, a significant challenge remains: the percutaneous nature of these implants creates a permanent transcutaneous breach—termed the "stoma"—which is prone to chronic wound healing complications and infection. As titanium is biologically inert, the body mounts a foreign body response (FBR) at the implant-tissue interface. This response results in the formation of a fibrous capsule and epithelial downgrowth at the stoma, compromising the impermeable epithelial protection at the interface. The absence of a robust epithelial seal leads to the formation of sinus tracts that serve as potential entry points for pathogens, ultimately increasing the risk of infection-related device failure. Currently, no clinically effective strategy exists to prevent or reverse skin downgrowth at the stoma. To address this challenge, we draw inspiration from the dental literature, where hemidesmosomes play a key role in anchoring gingival epithelial cells to the enamel surface. We hypothesize that replicating this biological adhesion mechanism at the percutaneous interface may enhance the impermeable epithelial seal at the implant exit site. We hypothesized that the use of a fluorapatite (FA) coating, which resembles the dental enamel surface, on titanium implants would promote hemidesmosome-mediated epithelial attachment and improve stomal healing.
***Methods:*** To test our hypothesis, we first examined the ability of FA surfaces to promote keratinocyte differentiation and hemidesmosome expression *in vitro.* For this, keratinocytes were seeded at a density of 6,000 cells/cm$^2$ onto cell culture plates, Titanium discs, and FA discs sintered at 1150C, and cultured in both DMEM + 10%FBS, and calcium-deficient DMEM + 10%FBS for 3, 7, and 12 days, after which they were subjected to evaluation using immunocytochemistry (ICC) and quantitative reverse transcription polymerase chain reaction (RT-qPCR) for protein and gene expression respectively. Upon confirming the bioactivity of FA surfaces, low-temperature coating techniques were developed and applied to titanium implants for *in vivo* evaluation. Coated and uncoated percutaneous implants were implanted in a non-load-bearing rat dorsal skin model for four weeks. Finally, to validate these findings under physiological loading conditions, a previously established load-bearing sheep amputation model was employed. Five sheep were implanted with optimized FA-coated implants and monitored for 12 weeks. Tissue surrounding the implant was collected at necropsy and compared to historical samples. For both the rat and sheep models, histological analyses were performed to measure epithelial downgrowth and the area of granulation tissue, while protein expression was assessed using immunohistochemistry (IHC).
***Results and Discussion:*** Overall, the data support our hypothesis that FA coatings enhanced epithelial sealing and reduced downgrowth. The *in vitro* ICC studies qualitatively showed surface-induced differentiation and hemidesmosome expression on FA compared to Ti surfaces. The RT-qPCR revealed quantitative increases in gene expression markers for differentiation (Keratin 10 and involucrin) and hemidesmosome (BP180) markers. Using the rat-back model, we confirmed that epidermal downgrowth and granulation tissue were significantly reduced around FA-coated implants when compared to uncoated implants. Additionally, wound-healing markers were significantly reduced in the FA-coated samples compared to the uncoated samples. The load-bearing sheep model also showed a decrease in both granulation tissue area and downgrowth in the FA-coated implant compared to the uncoated titanium implant, as well as a reduction of the expression of proteins necessary in wound healing.
***Conclusion:*** Our *in vitro* assays effectively predicted surface-induced keratinocyte differentiation and hemidesmosome expression on FA surfaces. These findings were further validated *in vivo*, demonstrating that FA coatings support epithelial integration with the implant interface. The results confirm the potential to improve the clinical success of percutaneous devices.

### 3D, functional, single-cell osteocyte networks in custom architectures within collagen on a chip.

Arun Poudel, Puskal Kunwar, Ujjwal Aryal, Anna-Blessing Merife, and Pranav Soman[1#]

[1]Department of Chemical and Biomedical Engineering, L.C. Smith College of Engineering Syracuse University, Syracuse, NY 13244 USA

**Disclosures**: Pranav Soman (3DMicrofluidics LLC)

INTRODUCTION: Osteocytes are the primary mechanosensory cells in bone. However, due to the difficulties in obtaining and culturing primary human osteocytes, the field has largely relied on immortalized murine osteocyte cell lines such as MLO-Y4, primarily studied in 2D culture systems. A lack of enabling tools hinders our understanding of how organized 3D osteocyte networks integrate information from individual cells and sense, respond, and adapt to various stimuli. Despite advances in cell-patterning, bioprinting, and microfluidics, current methods are unable to generate 3D osteocytes circuits at single-cells within natural extracellular matrices. Here, we report a novel technology, referred to as the 'CellNet' that can generate 3D, single-cell osteocyte circuits embedded within collagen matrix. We show that osteocyte CellNets with defined connectivity architectures can be built, defined stimuli (fluid flow, stimulants) or lethal injury to individual cells can be applied, and associated changes in real-time calcium signaling can be precisely monitored.

METHODS: Digital light projection stereolithography was used to design and fabricate a master mold using PEGDA resin and replica casting was used to fabricate PDMS three-chambered microfluidic chips. Type I collagen solutions at varying concentrations were perfused in the central chamber of the chips, and post-gelation, femtosecond laser (Ti:Sapphire, Coherent, Chameleon, USA) was used to ablate microchannel network using user-defined architectures. Then, murine osteocyte-like cell line MLO-Y4 (Kerafast, Inc. Boston, MA) were seeded and cultured in the side chambers of the chip. Cell viability was characterized using an Invitrogen Live/Dead assay kit, while morphology was assessed using staining (f-actin, nucleus). Osteocyte cell number and their connectivity, as the percentage of cells connected to the number of adjacent cells in the Cellnet, was quantified using Image J. Calcium Signaling Fluo-4 Calcium Imaging Kit (Invitrogen#F10489) dye was used to conduct calcium signaling experiments. To apply mechanical stimulation to the cells, 150µl of fresh media was pipetted in the inlet of Ch#1 which generates shear stress at the interface of Ch#1 and Ch#2. For biochemical induced cell responses, 5µl of ionomycin (45µmol.L-1) (Invitrogen#I24222) was introduced into the side chambers of the chip. To model the effects of network discontinuity, femtosecond laser irradiation (dosage = 0.64J/cm2) was used to lethally injure individual osteocytes within MLO-Y4 Cellnets and resulting changes to signal propagation and time-delays were analyzed.

RESULTS SECTION: Upon seeding, MLO-Y4s migrate into ablated networks (lumen size ~7-8µm) within hours, forming interconnected, 3D networks in a user-defined configuration. Results show that this template-based strategy is highly reproducible and provides control over cell-network layout and density. Functionality of MLO-Y4 Cellnets was tested using real-time Ca2+ signaling of individual cells and signal propagation throughout the network when subjected to only fluid flow stimuli, or a sequence of biophysical and biochemical stimuli, and within femtosecond laser assisted user-disrupted networks. Furthermore, these 'organized' osteocyte cellnets simplify image processing algorithms, enabling accurate mapping of how stimuli are converted and propagated across in 3D networks.

DISCUSSION: As compared to the current self-assembly based methods that exhibit high variability and poor reproducibility, Cellnets can generate organized 3D single-cell osteocyte networks and their real-time signaling responses to a range of stimuli can be accurately captured using simple cell seeding and easy-to-handle microfluidic chips. Cellnet could also emerge as an ideal tool to study signaling heterogeneity, a potentially important yet unstudied phenomenon in osteocyte biology. The modular nature of Cellnet allows 'swapping in' relevant cell types, ECM composition, network designs, optical tracers for other signals (NO, ROS, ATP) and/or fluorescent reporters or inhibitor drugs and acquire new knowledge using standard imaging and culture methods. Cellnets will enable broad utility and adoption in the field to test new hypothesis across cell, tissue, or stimulus types and by linking individual osteocytes and tissue networks' function could elucidate yet unknown higher-level emergent properties related to osteocyte biology.

SIGNIFICANCE/CLINICAL RELEVANCE: Cellnet technology, agnostic of cell types, ECM formulations, 3D cell-connectivity designs, or location and timing of network disruptions, could pave the way to address a range of fundamental questions related to osteocyte mechano biology.

ACKNOWLEDGEMENTS: NIH R01 AR083466-01 and R21 DK136083-0; Poudel, A., Kunwar, P., Aryal, U., Merife, A.B. and Soman, P., 2025. Cellnet technology to generate 3D, functional, single-cell networks in custom architectures within collagen. Biofabrication, 17(2), p.025036..

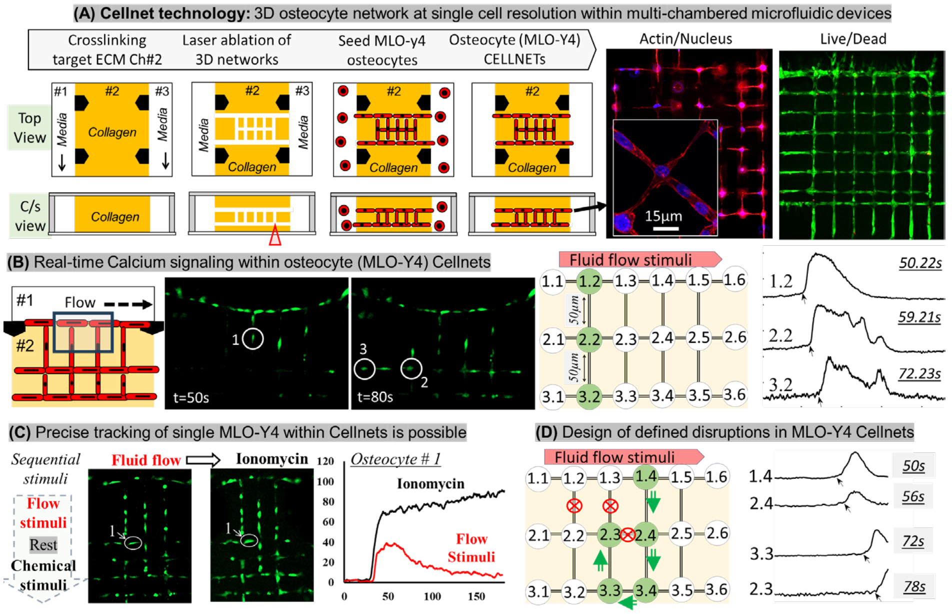

**Figure Caption:** (A) Schematic of process flow to generate organized 3D osteocyte circuits within a 3-chambered microfluidic device. (#1, #2, #3) (B) Ability to study real-time calcium signaling within MLO-Y4 cellnets, (C) Schematic showing application of a biophysical fluid flow stimuli in #1. Time-lapse fluorescence images showing calcium signaling at specific time-points. Simplified schematic: 1.2 represents a single osteocyte in row 1 and column 2, located at the interface of Ch#1-2. Upon stimulation, 1.2 initiates a Ca signal that propagates to adjacent connected osteocytes embedded deeper within collagen (2.2 and 3.2). (C) Signaling response of individual osteocytes and the entire network can be accurately analyzed in the presence of sequential biophysical and biochemical stimuli. (D) Real-time signaling within user-disrupted osteocyte Cellnets. Schematic showing application of a biophysical fluid flow stimuli in chamber #1 after femtosecond laser irradiation was used to lethally injury three cells (shown by cross mark). Simplified representation of how signal propagation is diverted due to targeted disruptions. Intensity plots showing delayed propagation of signal to cell 2.2 as signal travels from cell 1.4 to 2.4 to 3.4 to 2.2.

### Bone Multicellular Unit (BMU) chip under Pulsatile Unidirectional Fluid Flow Stimuli (PUFFS)

Anna-Blessing Merife[1], Arun Poudel[1], Angelika Polshikova[1], Michael P. Seitz[1], Zachary J. Geffert[1], Jason A. Horton[2], Mohammad Mehedi Hasan Akash[3], Anupum Pandey[4], and Saikat Basu[3], Daniel Fougnier[1], Paola Divieti Pajevic[5] and Pranav Soman[1#]
[1]Department of Chemical and Biomedical Engineering, L.C. Smith College of Engineering Syracuse University, Syracuse, NY 13244 USA
[2]Department of Neuroscience and Physiology, Alan and Marlene Norton College of Medicine, SUNY Upstate Medical University, Syracuse NY 13210 USA
[3]Department of Mechanical Engineering, South Dakota State University, Brookings, SD 57007, United States.
[4] Department of Mechanical and Aerospace Engineering, L.C. Smith College of Engineering Syracuse University, Syracuse, NY 13244 USA
[5] Translational Dental Medicine, Goldman School of Dental Medicine, Boston University, Boston, MA 02118 USA

**Disclosures**: Pranav Soman (3DMicrofluidics LLC)

INTRODUCTION: Mechanistic understanding of how mechanotransduction drives bone remodeling remains incomplete due to the constraints of models that require tradeoffs between experimental flexibility of 2D in vitro models and the physiological relevance of animal models. Further progress in this field is confounded by experimental complexity, high costs, slow throughput, poor reproducibility and low statistical power of these models. Recently, we designed and developed a microfluidic based in vitro model to study 3D osteocyte networks cultured under daily application of Pulsatile Unidirectional Fluid Flow Stimuli (PUFFS) for up to 21 days.(1) In this work, we have extended this model to develop a bone multicellular unit device (BMU-Chip) to include a 3D network of interconnected osteocytes (OCY454) housed in a collagen matrix, that are co-cultured with pre-osteoblasts (MC3T3-E1.4) and/or pre-osteoclastic cells (Raw-264.7). This model has the potential to significantly advance our understanding of how osteocytes respond to mechanical stimuli, propagate signals across their networks, and coordinate mechanoadaptive remodeling by osteoblasts and osteoclasts.

METHODS: Digital light projection stereolithography was used to design and fabricate a three-chambered PDMS microfluidic chip. Murine osteocytic cells (OCY454) were encapsulated in collagen matrix (2.5 or 4mg/ml) within the central chamber of the chip to form self-assembled three-dimensional (3D) cell networks and subjected to daily PUFFS (0.33Hz) for 21 days using chamber 3 of the chip. At specific time-points, pre-osteoblastic cells (MC3T3-E1.4) were introduced in chamber 1 and cultured under osteogenic media for 21 days. For some chips, MC3T3-E1.4 were removed, and model pre-osteoclastic cells (Raw-264.7) were added in chamber 1, while continuing to apply PUFFS in chamber 3. This experimental design was chosen to mimic in vivo like conditions and study differentiation of osteoblasts, or osteoclasts within the BMU-chip. At specific time-points, cell morphology, viability, protein and gene expression, and calcium signaling were characterized using established assays.

RESULTS: Representative brightfield image shows GFP+ OCY454 cells encapsulated within collagen (4mg/ml) in the central channel of the chip (A), while MC3T3s are seeded in #1, and PUFFS is applied in #3, from Day 10 to 31. For tri-culture chips, Raw264.7 cells are added on Day 31 and assessed on Day 34. At specific time-points, cell viability, morphology, gene expression, and intracellular calcium signaling, are characterized in co-culture and tri-culture systems, and results are compared to mono-cultures chips. Results show a combination of PUFFS and osteocytes within a co-culture system up-regulated osteoblastic and osteoclastic genes associated with maturity and matrix modeling. Calcium signaling within the central chamber with 3D osteocyte network is more sensitive to PUFFS as compared to MC3T3s seeded in chamber 1. Overall, such BMU-chips enable complex study of multiple cells types relevant to skeletal tissue while applying cyclic mechanical stimuli.

DISCUSSION: BMU-on-chip approach that overcomes limitations of current model systems by integrating several novel features including (a) applying defined directional mechanical stimuli using PUFFS simulating physiologically relevant forces and interstitial fluid flow; (b) detection of real-time signaling and signal propagation across 3D osteocyte networks; (c) co-/tri-culturing cell types relevant to the BMU in longer-term cultures (up to 34 days) while remaining compatible with standard imaging and analysis techniques.

SIGNIFICANCE/CLINICAL RELEVANCE: This work will significantly advance our understanding of osteocyte mechanotransduction and its role in bone remodeling. By elucidating how osteocytes coordinate osteoblast and osteoclast activities in response to mechanical loading, this work could be used to identify new mechanisms of bone diseases involving aberrant mechanotransduction. Integrating novel microfluidic technologies with cell biology will open new avenues for in vitro modeling of patho-physiological processes, reduce reliance on animal models, and accelerate discovery of new treatments.

ACKNOWLEDGEMENTS: NIH, R01 AR083466-01, COBRE Award 2P20 GM109024.

REFERENCE: (1) doi: https://doi.org/10.1101/2025.04.30.651549.

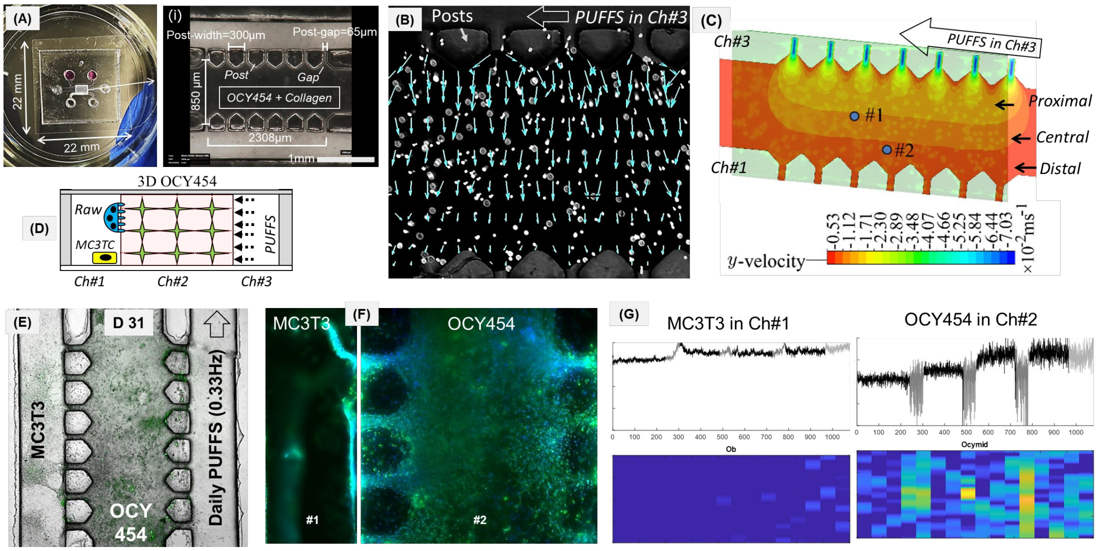

**Figure Caption:** (A) Picture of a three-chambered microfluidic PDMS chip with 3D network of OCY454s in collagen in the central chamber. (B) Vector map superimposed on brightfield image showing the magnitude of bead displacement. (C) Velocity distribution within collagen during PUFFS. (D) Schematic showing chip cross-section with seeding of model pre-osteoblasts or pre-osteoclasts in chamber 1, while applying PUFFS to chamber 3. (E) Brightfield image showing the top-view of the chip with GFP+ OCY454 cells in Ch#2 on Day 31 with MC3T3s seeded in #1 on Day 10 and culture using osteogenic media for 2 weeks. (D) Cell morphology of cells in different chambers of the chip. (G) Calcium signals of individual OCY454s recorded from chamber 1 and 2 when subjected to PUFFS 3 times.

### APOE4 exerts sexually dimorphic effects on bone quality in a mouse model of neurodegeneration

*Gurcharan Kaur, PhD[1]*, Charles A. Schurman, PhD[2]*, Serra Kaya, PhD[1], Joanna Bons[2], PhD, Carlos Galicia Aguirre, PhD[2], Kathy Liu[2], Christina King, PhD[2], Kenneth A. Wilson, PhD[2], Harrison L. Baker[2], Lisa M. Ellerby, PhD[2+], Birgit Schilling, PhD[2+], Tamara Alliston, PhD[1+] (*equal authorship, +equal shared correspondence) 1. University of California, San Francisco, CA 2. Buck Institute for Research on Aging, Novato, CA, USA*

Osteocytes engage in perilacunar canalicular remodeling (PLR) to maintain lacunocanalicular networks (LCN), bone quality, and skeletal and systemic homeostasis. In three independent models of osteocytic PLR suppression, we observed upregulation of neurodegenerative transcriptional pathways in bone. Moreover, individuals with bone fragility and osteoarthritis experience an increased risk of Alzheimer's disease (AD) and vice versa. However, the mechanisms driving the coincident decline in skeletal and neurological health remain unclear. This study aims to determine the effect of AD on osteocytes and bone quality using humanized APOE knock-in mouse model of late-onset AD (APOE2 protective, APOE3 neutral, and APOE4 AD risk factor). We *hypothesize that AD risk allele APOE4 is sufficient to compromise skeletal function with bone quality deficits due to suppression of osteocytic PLR*.

Long bones and hippocampus were isolated from 15-month-old male and female (N=8-11/group) APOE mice to analyze the transcriptome, proteome, bone structure (microCT), bone mechanical properties (3-pt bending), LCN and PLR marker expression (histology). We found a striking sex-specific effect of the APOE4 allele on bone. Female bone was more susceptible to APOE allele-mediated transcriptome changes compared to males with a higher number of differentially expressed genes (**Fig. A** Females:146, Males:22, FDR-0.1), and downregulated neuronal and synaptic pathways in APOE4 vs. APOE3 bone. Relative to the hippocampus, the female bone proteome was more severely affected by APOE allele status (**Fig. B** APOE4 vs APOE3 DE proteins - Hippocampus: 40, Bone: 393, FDR-0.05), with enriched TGF-β signaling and ECM remodeling pathways. Though we observed no significant difference in trabecular and cortical bone structure in either sex, mechanical testing revealed bone fragility in APOE4 females, with reduced work to fracture (2-fold decrease, p=0.0021) and post-yield displacement (1.7 fold decrease, p=0.015) compared to APOE3 (**Fig. C**). This APOE4 bone quality deficit in females is accompanied by evidence of PLR suppression, with disorganized LCN, truncated canaliculi, and reduced osteocytic expression of Cathepsin K, Sclerostin and MMP13, in APOE4 relative to APOE3 bone (**Fig. D**).

We demonstrate that AD driven by the APOE4 risk allele is a bone disease that selectively impacts bone quality and osteocyte function in female mice, mirroring the disproportionate impact of AD, osteoporosis, and osteoarthritis in women.

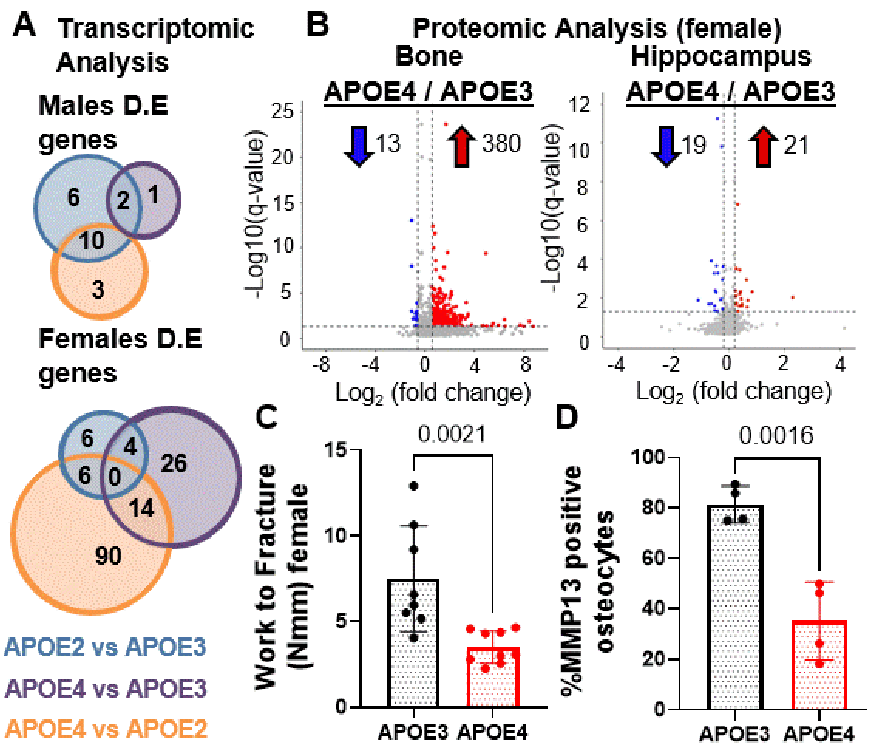

**APOE4 selectively impacts bone quality and osteocyte function in female mice. (A)** Bulk RNAseq analysis of male and female humanized APOE knock-in mouse bones reveals higher number of differentially expressed (D.E) genes in female bone compared to males. **(B)** Proteomic analysis of female bone and hippocampus reveals that APOE alleles disproportionately affect female bone proteome compared to the hippocampus. **(C)** 3-point bending test shows significantly lower work to fracture in female APOE4 bone compared to APOE3 bone indicating APOE4 allele-induced bone fragility in female mice. **(D)** Immunofluorescence quantification for MMP13 expression shows decline in percent MMP13 positive osteocytes in female APOE4 bones compared to APOE3 indicating PLR suppression.

### Mitochondrial mechanisms of age-related decline in bone quality and osteocyte function

*Isabel Amado, PhD[1], Gen Matsumae, PhD[1], Serra Kaya, PhD[1], Clarissa Aguirre Luna[1], Jean Luke Campos[1], Gabriella Baylon[1], Simon Melov, PhD[2], Akos Gerencser, PhD[2], Rebecca L. Robker, PhD[3] and Tamara Alliston, PhD[1,4]*
*[1]University of California San Franscisco, San Francisco, CA, USA, [2]Buck Institute, Novato, CA, USA, [3]The University of Adelaide, Adelaide SA, Australia, [4]National Institute of Health, Maryland, USA*

Aged-related loss of bone quality arises in part from TGF-β dependent suppression of osteocyte perilacunocanalicular remodeling (PLR). Mitochondrial function declines with age and TGF-β signaling is modulator of mitochondrial function in many tissues [1,2, 3]. In tibia and fibulae from mice with poor bone quality and PLR suppression, due to aging or osteocyte-intrinsic defects in TGF-β signaling ($T\beta RII^{OCY-/-}$), dynamic imaging revealed reduced mitochondrial membrane potential ($\Delta\Psi M$) in osteocytes using ex vivo photon microscopy. In fibulae from $T\beta RII^{OCY-/-}$ mice (15 weeks) relative to wild type (WT) controls $\Delta\Psi M$ was lower by 30mV (n=6, $p<0.05$). Gene set enrichment analysis of RNAseq using osteocyte-enriched cortical bone from the same conditions confirmed the repression of pathways associated with mitochondrial function (n=4, $p<0.05$). We demonstrated that $T\beta RII^{OCY-/-}$ mice have compromised mitochondrial function (Figure 1A-B). To test the hypothesis that restoration of mitochondrial function would be sufficient to rescue age related declines in osteocyte function and bone quality, we treated male and female mice with BGP-15 (100 mg/kg), a pharmacologic agent that mitigates mitochondria dysfunction in other tissues relative to PBS for 18 days (n=6-8 mice/group). BGP-15 was insufficient to normalize the impaired osteocyte $\Delta\Psi M$ in 12-month old WT female tibia relative to 4-month old controls. However, even without detectable changes in bone structure or mineral density, BGP-15 significantly improved mechanical properties and PLR analysis (Figure 1C-D), suggesting that BGP-15 improves bone quality. RNAseq of BGP-15 treated bone showed downregulation in lipid and fatty acid metabolism, Plin4 and Plin5 (n=4, FDR<0.1) (Figure 1E). In MLO-Y4 cells challenged with oxidative stress (10 μM $H_2O_2$ for 3 or 6h), BGP-15 reduced lipid droplet accumulation (Figure 1F). Moreover, BGP-15 reduced Plin4 mRNA and induce MMP13 mRNA levels (Figure 1G), suggesting that BGP-15 exerts cell-intrinsic effect on osteocyte metabolism. Therefore, age-related declines in bone fragility accompanied by declines in osteocyte mitochondrial function can be mitigated by systemic agents that regulate osteocyte cellular metabolism and PLR.

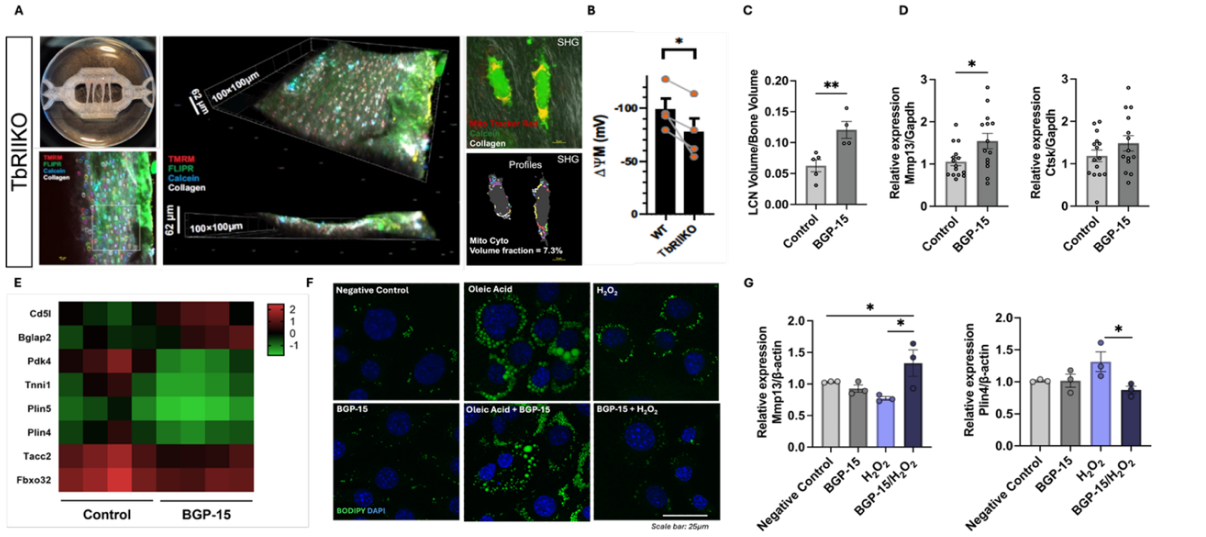

**Figure1: A-B** Visualization of mitochondria and mitochondrial membrane potential ($\Delta\Psi M$) measurement was performed on mouse fibula. **C** Female WT mice Lacuna/canalicular network (LCN) analysis were performed in samples treated and untreated with BGP-15. **D** Relative gene expression analysis by qPCR was performed, MMP13 and Ctsk. **E** RNAseq analysis was performed to mouse humerus **F** Fluorescence microscopy analysis of MLOY4 cells stained with BODIPY 493/503 and DAPI **G** Relative gene expression of MMP13 and lipid droplet maker Plin4.

**References**:
[1]Sambrook et al, (2006) Lancet; [2]Kobayashi, et al, (2015) Scientific Reports; [3]Agota et al, (2020) Molecules

### Deletion of Osteocytic Von Hippel Lindau Prevents Age-Associated Bone Loss

*Sarah V. Mendoza[1], Deepa K. Murugesh[2], Gabriela G. Loots[1,2], Damian C. Genetos[1], Clare E. Yellowley[1]*
*[1]University of California, Davis, CA, [2] Lawrence Livermore National Laboratories, Livermore, CA*

Nearly 200 million people worldwide suffer from osteoporosis, causing >8.9 million fractures every year predominantly in the aging population. Impermanence and adverse side effects of current osteoporotic therapies necessitate the identification of novel therapeutic targets that provide lifelong improvements in bone mass and quality. Hypoxia inducible factor (HIF) signaling drives skeletal health and maintenance. HIFs are transcription factors composed of oxygen-dependent HIF-α subunits (transcriptionally active paralogs: HIF-1α, HIF-2α) and a constitutively stable nuclear HIF-ß subunit. Under normoxia, HIF-α subunits are hydroxylated, recognized by an E3 ligase complex Von Hippel-Lindau (VHL), and targeted for degradation. With hypoxia, HIF-α subunits escape VHL-mediated degradation, complex with HIF-ß, and induce osteoanabolic transcriptional changes (1). In male and female mice, we find that deleting osteocytic (OCY) *Vhl* via *Dmp1*-Cre generates striking increases in cortical and trabecular bone mass, while expressing a degradation-resistant (cDR) OCY HIF-2α, not HIF-1α, generated a high trabecular bone mass phenotype. The objective of this study was to evaluate the effects of OCY VHL/HIF manipulation on the aging skeleton. **We hypothesize that the osteoanabolic skeletal effects of VHL/HIF manipulation will abrogate age-induced bone loss in male and female mice**. Using micro-computed tomography (uCT) to evaluate changes in cortical and trabecular microarchitecture, we utilized 4-month-old (adult) and 18-month-old (aged) mice with loss-of-function *Vhl* (*Vhl* cKO), gain-of-function HIF-1α (HIF-1α cDR), or gain-of-function HIF-2α (HIF-2α cDR) mutations in OCYs.

MicroCT revealed robust osteoanabolic potential in *Vhl*-deficient femora that persisted with age in both sexes (**Fig.1A**). *Vhl*-deficient male mice showed increased cortical and trabecular bone mass that persisted with age, whereas *Vhl*-deficient female mice exhibited progressive osteoanabolism in both skeletal compartments (**Fig.1B-C**). HIF-1α cDR mice demonstrated age-induced cortical or trabecular bone loss in both sexes, indistinguishable from that observed in Cre-negative aged controls (**Fig.1B-C**). HIF-2α cDR mice exhibited age-associated cortical bone loss in both sexes (**Fig.1B**). Male HIF-2α cDR maintained trabecular bone mass with age, whereas female HIF-2α cDR mice showed age-associated trabecular bone loss (**Fig.1C**).

VHL/HIF signaling performs distinct compartment- and age-related functions in the aging skeleton. OCY *Vhl* deletion harbors robust osteoanabolic potential that is maintained in male mice but is progressive in female mice- filling available trabecular space and extensively invading the cortical medullary cavity. HIF-2α accumulation generated a sex- and compartment-specific bone loss, reducing cortical bone loss in both sexes yet abrogating trabecular bone loss in male mice. HIF-1α accumulation did not prevent age-induced bone loss. Our finding that *Vhl* deletion not only prevented age-associated bone loss, but promoted bone accrual in older animals is striking. Further, our data show that this may occur independent of HIF-α signaling. Understanding the molecular mechanisms by which *Vhl* deletion drives continued bone formation in aged mice could provide novel treatment strategies for age-related bone loss.

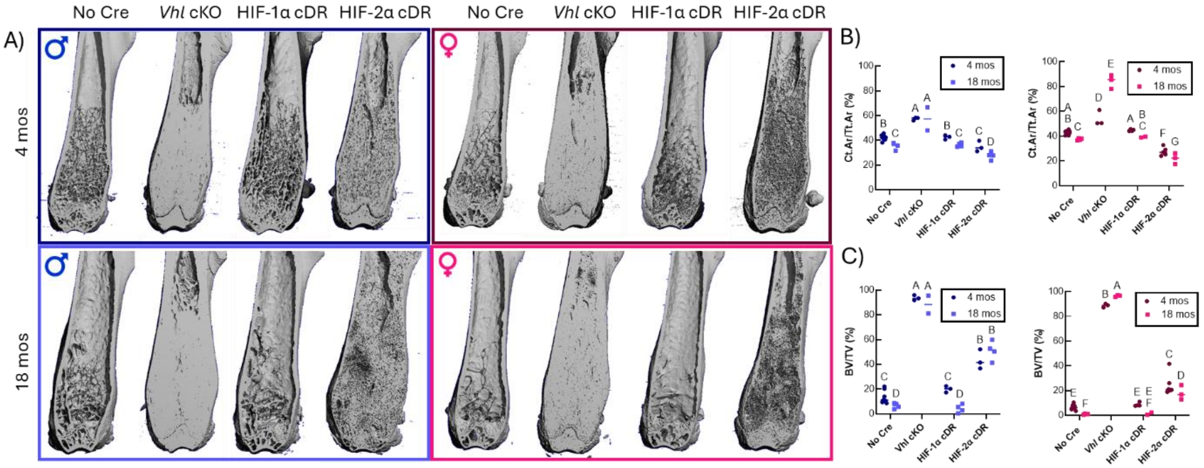

**VHL/HIF manipulation imparts sexually dimorphic effects in the aging skeleton**. Representative longitudinal 3D reconstruction images from **(A)** adult and aged male (left) and female (right) Cre-negative, *Vhl* cKO, HIF-1α cDR, and HIF-2α cDR mice. Quantification of **(B)** cortical bone area fraction and **(C)** bone volume fraction of the trabecular compartment from male (*left)* and female (*right*) Cre-negative, *Vhl* cKO, HIF-1α cDR, and HIF-2α cDR mice. Bars represent mean ± SD; groups with different letters are statistically different from each other. n = 2–12 for adult and aged mice/sex/genotype.

References:
(1) Semenza GL. J Bone Miner Res. 2014;29(4):820-829.
(2) Loots GG et al. Bone.2018 Nov; 116:307-314.
(3) Mendoza SV et al. JBMR Plus. 2023 Feb 17;7(4).
(4) Borah B et al. Bone. 2006 Aug; 39(2):345-52.

### Single-Cell Transcriptomic Analysis of Stromal Vascular Fraction Differentiation on Fluorapatite Surfaces

James Firpo[1,2], James Peter Beck[1,2], Jay Agarwal[1,2], Sujee Jeyapalina[1,2], Jill Shea[1,2,]
[1]Veterans Affairs Medical Center, Salt Lake City, UT, [2]University of Utah, Salt Lake City, UT

**Background**: Allograft and autograft shortages have driven the development of engineered alternatives for bone grafting. Among synthetic materials, hydroxyapatite (HA) is a promising candidate due to its osteoconductive properties; however, it often lacks sufficient structural integrity. To enhance the mechanical strength of HA grafts, fluoridation and high-temperature sintering are viable options. To further optimize these apatite-based grafts, incorporating autologous cells is considered necessary. Our previous data demonstrated that adipose-derived stem cells (ADSCs)—the stem cell subset of stromal vascular fractions (SVFs)—differentiate into osteoblasts more rapidly on fluorapatite (FA) surfaces compared to HA. Consequently, SVF is considered a suitable source of autologous cells. As ADSCs are capable of trilineage differentiation into adipocytes, chondrocytes, and osteoblasts, it is considered essential to confirm the efficiency of ADSCs extracted from the SVFs to undergo osteogenic differentiation on FA surfaces. Given the wide range of possible modifications to fluoridation and sintering processes, testing each variation in animal models is impractical. Therefore, we propose using single-cell RNA sequencing (scRNA-seq) technique to assess surface bioactivity prior to *in vivo* studies. In this study, we hypothesized that SVF cells seeded on FA surfaces would undergo osteogenic differentiation more rapidly than those seeded on HA, as indicated by the expression of osteogenic transcriptional markers.

**Materials and Methods**: Human fat tissue was obtained with an IRB-approved protocol (#89989). Using an optimized collagenase-based procedure, SVFs were extracted and viable cell types quantified using fluorescence-activated cell sorting. Cells at a density of $1.6x10^5/cm^2$ were seeded on FA, HA, and cell culture (CC) plates for various time points (12 hours, and at 4, 7, and 14 days). At the pre-defined time points, cells were harvested and subjected to scRNA-seq. After processing the results with FastQC and CellRanger, the data were analyzed using Seurat following the removal of low-quality cells and doublets. Cell type prediction was performed through enrichment analysis using Enrichr. Also, differential gene expression analysis was used to infer cell lineages at each time point. Several external data sets were compared to the baseline time point to assess their comparability to other data sets utilizing Harmony integration. Additionally, commercial cell lines were used to determine if the differentiating cells were comparable to established ADSC lineage cells.

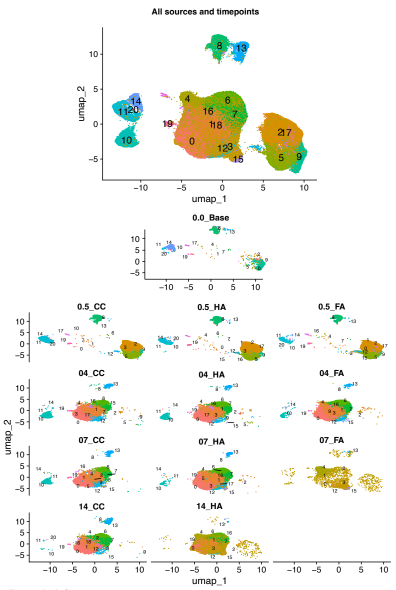

Figure 1 (top): Cluster map of entire data set.
Figure 1 (middle): Cluster map of baseline data (before plating).
Figure 1 (bottom): Cluster maps of data split at timepoints and by sources. The number represents the day the cells were measured, with .5 being 12 hours after plating.

**Results and Discussion**: At baseline, approximately 46% of the cells were immune cells, consisting of T-cells (Cluster 14), macrophages (Cluster 8), and natural killer cells (Cluster 11). Another 44% were stem cells/fibroblasts (Cluster 9), and 10% were endothelial cells (Cluster 19) (**Figure 1**). The percentage of each cell type was similar to that obtained by analyzing our baseline cells with FACS, as well as when compared to other published datasets using data integration. There were no significant differences in the types of cells adhering to the different surfaces at 12 hours. However, the number of immune cells decreased at each subsequent time point, most disappearing by Day 7 from the FA group and Day 14 from the HA group. In contrast, the CC group retained about 10% of immune cells, primarily macrophages, at Day 14. It is known that adipose-derived stem cells can differentiate into three main lineages: adipocyte, chondrocyte, and osteocyte. There were pre-adipocyte cells present in both the baseline and 12-hour groups; however, their numbers decreased at each subsequent time point. Based on enrichment analysis and specific gene expression, the stem cell clusters 0 and 1 were undergoing differentiation along the osteocyte pathway, with limited evidence of progression along the chondrocyte pathway. By Day 7, 99% of the cells in the FA group were in Clusters 3, 4, and 15, with indications that they were continuing along the osteocyte pathway. Unfortunately, the Day 14 FA sample was lost during sequencing. By Day 14, the HA clusters began to resemble the Day 7 FA clusters, although there was still greater cell diversity in the HA sample compared to the FA group.

**CONCLUSION:** Almost half of the baseline cells were immune, but their proportion of total cells decreased over time. On the FA surfaces, SVF differentiated along the osteoblast lineage without any growth factors at an earlier time point than on HA surfaces. Future analysis of the dataset will focus on gaining a better understanding of the mechanistic pathways of SVF differentiation on apatite surfaces.

### Mechanical Instability Drives Fibrotic Remodeling via Strain-Driven Immune Reprogramming in Fracture Healing

*[1]Matthew Patrick, [2]Jaimo Ahn, [2]Kurt Hankenson, and [1]Ramkumar T. Annamalai**
*[1]University at Buffalo, Buffalo, NY, [2]University of Michigan, Ann Arbor, MI*

**Introduction:** Musculoskeletal injuries are a leading cause of long-term disability, with 5–15% of the 7 million annual fractures resulting in delayed healing or nonunion. Hypertrophic nonunions, the most common subtype, arise from excessive interfragmentary movement and high mechanical strain. While an early inflammatory response is essential for bone repair, persistent instability can skew immune behavior, promoting fibrosis over regeneration. Mechanical strain may reprogram macrophages toward profibrotic phenotypes, yet the spatial and cellular mechanisms underlying this process remain poorly defined. To address this gap, we developed a murine model of delayed fracture healing mimicking the mechanical etiology of hypertrophic nonunion. Using single-cell RNA sequencing and spatial transcriptomics, we identified early macrophage reprogramming and sustained fibroblast activation as key drivers of fibrotic remodeling, uncovering predictive biomarkers and mechanosensitive signaling pathways as potential therapeutic targets.

**Methods:** Transverse femoral fractures were created in C57BL/6 mice and stabilized with custom intramedullary NiTi nails of either low (2.7 N/mm) or high (16.5 N/mm) stiffness to produce low (<5%) or high (>15%) interfragmentary strain. Healing tissues were collected over time for analysis using 10X Genomics single-cell RNA sequencing and Visium spatial transcriptomics. Cell type annotation, gene expression mapping, and intercellular communication analyses were performed using Seurat and CellChat. To model strain effects in vitro, primary murine macrophages and fibroblasts were co-cultured in fibrin gels and subjected to 0%, 5%, or 15% uniaxial strain for 2 hours using the Cytostretcher platform, followed by gene expression analysis.

**Results & Discussion:** At day 1, single-cell RNA-seq showed that high-strain fractures induced a pro-fibrotic macrophage phenotype with upregulation of *Smad3* and inflammatory mediators (*S100a9, Spp1, Cxcr2*), activating TGF-β signaling and ECM remodeling. In contrast, low-strain fractures triggered a restrained macrophage response focused on ribosomal and anti-inflammatory genes (*Rpl35, Ccr2*). These divergent immune profiles persisted through day 7, defining distinct healing trajectories. High-strain macrophages maintained elevated fibrotic and inflammatory gene signatures (*Mmp12, Thy1, Il18r1*), while low-strain macrophages exhibited a regulatory IL-18-centered profile. By day 14, infiltrating monocytes in high-strain fractures differentiated into a hybrid phenotype expressing both inflammatory (*Ptgs2, Igf1*) and matrix-remodeling markers (*Col3a1, Tgfbr3*), whereas low-strain monocytes favored immune homeostasis (*Ccl2, Plac8).* These transcriptional paths contributed to the divergent tissue environments seen by day 21. On day 21, spatial transcriptomics revealed fibroblast-rich zones in high-strain calluses, co-localized with *Lgals3⁺* macrophages and enriched for fibrosis-related genes (*Pdgfrb, Col3a1, Spp1).* High-strain macrophages sustained the expression of remodeling mediators (*Ngp, Cxcl3, Socs3*), while low-strain macrophages displayed a more homeostatic profile (*Slfn4, Ace, Ceacam1*). Cell signaling analysis identified the THBS–SDC4 axis as a key mediator of macrophage–fibroblast crosstalk, with macrophage-derived *Thbs1/2* engaging fibroblast *Sdc4* to sustain fibrotic signaling—absent under low strain. Enhanced SPP1 and TGF-β signaling also emerged as dominant pro-fibrotic pathways in high-strain calluses. Supporting this, *in vitro* co-cultures under 15% strain showed significant upregulation of fibrotic markers (*Col3a1, Thbs1, Acta2*) in fibroblasts, and *Mrc1* in macrophages, confirming strain-induced fibrotic activation. Together, these findings demonstrate that mechanical instability drives early macrophage reprogramming and persistent macrophage–fibroblast communication through THBS–SDC4 and SPP1–TGF-β pathways, promoting fibrosis and impaired healing. These mechanosensitive circuits represent promising therapeutic targets to redirect healing toward regeneration.

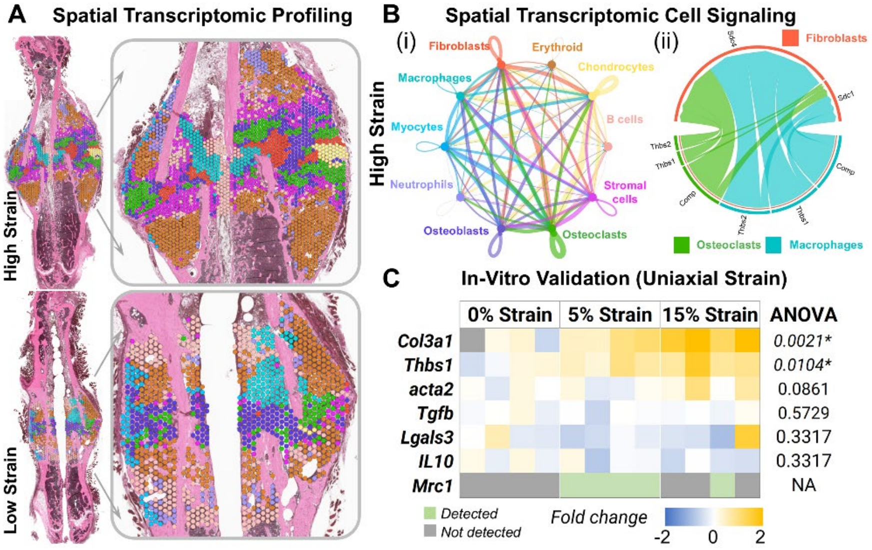

*Fig. 1. Transcriptomics of the fracture callus. (A) Spatial transcriptomics of day 21 calluses under high (top) and low (bottom) strain, with cell types annotated by gene markers. (B) (i) Cell-cell communication networks in high-strain callus highlighting macrophage–fibroblast interactions. (ii) THBS–SDC4 signaling identified as a key pathway. (C) Heatmap of gene expression in macrophages and fibroblasts exposed to static, 5%, or 15% uniaxial strain.*

### Osteocyte-conditional knockout of *Bmal1* results in decreased bone formation response to in vivo tibial loading in mice

*Leanne van der Merwe, MBBS[1,2], Micheal Bruccoleri, PhD[1], Meet Shah,BDS, MSc[1,2] Catherine Julien[1], MSc, Nicolas Cermakian, PhD[3,4], Suzanne N. Morin, MD, MSc, Bettina M. Willie, PhD[1,2]*

*[1]Research Centre, Shriners Hospitals for Children-Canada, Montreal, Qc, Canada, [2]Faculty of Dental Medicine and Oral Health Science, McGill University, Montreal, Qc, Canada, [3]Douglas Research Centre, Montreal, Qc, Canada [4]Department of Psychiatry, McGill University, Montreal, Qc, Canada, [5]Faculty of Medicine and Health Sciences, McGill University, Montreal, Qc, Canada, [6]Research Institute of the McGill University Health Center*

The existence of clock genes in bone cells (1), identification of rhythmic oscillation of osteocyte-specific genes *Sost* and *Dkk1*, and time-dependent tissue level response to in vivo mouse tibial loading supports the notion of circadian control on bone (2). Studies have shown that deletion of essential clock gene *Bmal1* in osteoblast (3) and osteoclasts (4) result in low and high bone mineral density, respectively. We aimed to investigate how disruption of *Bmal1* in osteocytes (OcKO) alters bone mass, microstructure, strength and mechano-adaptive response.

OcKO mice were obtained through the Cre-loxP system. 10-week-old male and female OcKO mice and their $Bmal1^{fl/fl}$ and Dmp1Cre littermate controls (n=7-10/genotype/sex) were assessed in two separate cohorts. Tibiae (baseline & day 21) and vertebrae (day 21) of the first cohort were assessed by µCT (8µm) and histomorphometry. Strain-matched in vivo tibial loading (216 cycles, at 4Hz, 1200µε, 5d/wk, 2wks) was conducted on the left limb of the second cohort, and in vivo µCT imaging of the left and right tibia (nonloaded control) were analyzed at baseline and day 15. ANOVA, Tukeys posthocs tests t-tests, and paired t-tests were performed ($p<0.05$).

Osteocyte-specific deletion of *Bmal1* was confirmed by PCR. A cortical bone phenotype was observed in the tibia of female OcKO mice, but not in the vertebrae. OcKO exhibited greater cortical thickness (Fig. 1A), area and total area compared to $Bmal1^{fl/fl}$ mice. No difference was observed in mineral apposition rate between genotypes. Male mice did not exhibit a bone phenotype. After in vivo tibial loading in female mice, all genotypes had a greater change from baseline cortical area and thickness (Fig. 1B) in loaded versus non-loaded limbs. Interestingly, a lower response to loading (eg. cortical thickness, area, total area, cortical area/total area, Ec.MV/BV, Ps.MV/BV) was observed in OcKO mice compared to $Bmal1^{fl/fl}$ mice ($p<0.05$) For example, in $Bmal1^{fl/fl}$ mice Ec.MV/BV in the loaded limb was 449% greater than the control limb, whereas in OcKO mice, the increase was only 136%. Trabecular bone mass changes from baseline did not differ across genotypes.

Loss of Bmal1—and consequently, the circadian clock—in osteocytes led to increased cortical bone mass at the tibial mid-diaphysis and a diminished mechano-adaptive response in female mice. Ongoing research is focused on identifying the molecular mechanisms underlying this increased bone mass and reduced bone formation response to loading in female OcKO mice, as well as exploring mechano-adaptation in male OcKO mice. Gaining insight into the role of the circadian clock in bone maintenance and adaptation could uncover new therapeutic targets for enhancing bone health.

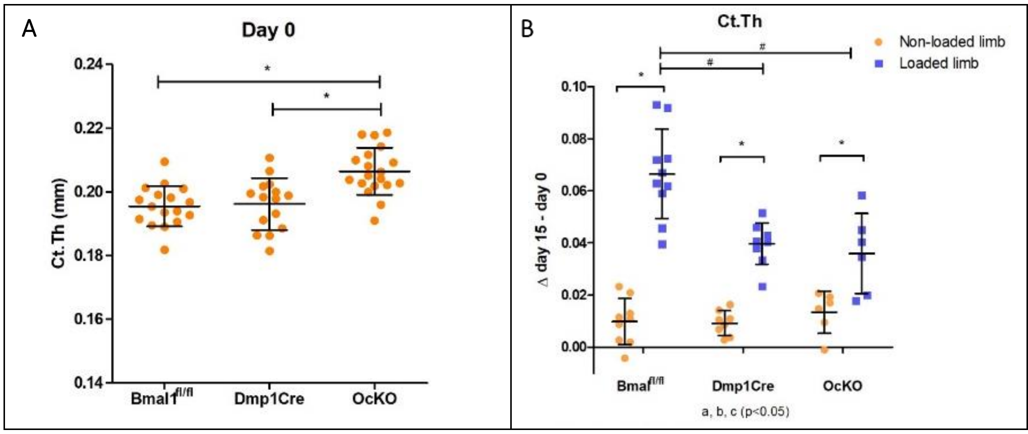

Fig 1. (A) Baseline µCT analysis of female mice at tibial midshaft; Means ± SD; * indicates differences between genotypes determined by t-test ($p<0.05$).

(B) Change from baseline (day15-day0) in cortical bone thickness after 2-wks of in vivo loading. Means ± SD; ANOVA (a=genotype, b=loading, c=interaction), # indicates Tukeys posthoc test and * paired t-test ($p<0.05$).

1. Schilperoort et al. , FASEB Journal., 2020
2. Bouchard et al., Bone, 2021
3. Takarada et al., JBMR, 2017
4. Xu et al., JBMR, 2016

### Cigarette Smoke Extract Induces Redox Imbalance and Dysregulated Life Fate in the Cartilage Endplate Chondrocytes

*Hui Li[1], Yiqing Ye[2], Joshua Kelley[1], Hai Yao, PhD[1,2], Danyelle M. Townsend, PhD[2], Yongren Wu, PhD[1,2]*
*[1]Clemson University, Clemson, SC, USA, [2]Medical University of South Carolina, Charleston, SC, USA*

**Introduction**: Cigarette smoking is a major public health concern. Among musculoskeletal disorders, it is frequently associated with intervertebral disc (IVD) degeneration [1]. Previous in vivo animal models have showed that cigarette smoke exposure disrupts collagen architecture in the annulus fibrosus (AF) and induces fibrotic changes in the nucleus pulposus (NP) [2-3]. Our recent studies using Sprague Dawley (SD) rats further revealed that two-month cigarette smoke exposure induces aberrant cartilage endplate (CEP) calcification, along with progressive IVD degeneration, suggesting its potential as a critical marker of degeneration [4-5]. However, the mechanisms underlying cigarette smoke-induced aberrant CEP calcification remain unclear. Recognizing the pivotal role of redox homeostasis in maintaining chondrocyte fate [6], we hypothesize that cigarette smoke induces oxidative stress by increasing oxidant levels and impairing antioxidant defenses in CEP chondrocytes. This imbalance further perturbs the hypertrophy–apoptosis axis of chondrocytes, ultimately leading to aberrant CEP calcification. To validate this hypothesis, we established an in vitro cigarette smoke extract (CSE) collection – entire IVD tissue culture model to characterize the levels of oxidant, antioxidant, and osteogenic marker expressions in CEP chondrocytes. Understanding the role of redox imbalance in the dysregulation of CEP chondrocyte life fate could provide valuable insights into the mechanism of endplate calcification and inform potential therapeutic development.

**Method**: Cigarettes were smoked using a custom syringe-based apparatus following ISO 3308 guidelines [7]. To ensure consistency across CSE batches, concentrations were measured by optical density at 320 nm, and nitrite levels were quantified using Griess reagent kit. Lumbar motion segments (L1–L6) were harvested post-euthanasia from three institutionally approved SD rats, pre-cultured for two days, and then treated for three days in control (5 mM glucose, 5% $O_2$), 10% CSE, or 10% CSE + 5 mM N-acetylcysteine (NAC). After treatment, IVD tissues (CEP, AF, NP) were isolated, flash-frozen in liquid nitrogen, and stored at −80 °C. Protein solutions were extracted and quantified using the Pierce™ BCA Protein Assay Kit. Reactive oxygen or nitrogen species (ROS/RNS) and Glutathione (GSH) levels were assessed via the OxiSelect™ and thiol fluorescent probe IV assays, respectively. Alkaline phosphatase (ALP), collagen X, and Caspase-3(Cas-3) expressions were measured using colorimetric and fluorescent assays. Two-tailed t-tests were used for statistical analysis.

**Result**: The prepared 100% CSE exhibited consistent quality and a stable nitrite concentration (Fig. 1). For redox and osteogenic markers, total and regional values were normalized to the control and AF control groups, respectively. CSE treatment increased overall ROS/RNS levels by 32%, which were reduced by 31% with NAC (Fig. 2A). Regionally, the CEP showed the greatest ROS/RNS elevation (51%), compared to 26% in NP and 7% in AF; NAC treatment reduced ROS/RNS in the CEP by 43% (vs. 28% in NP and 10% in AF) (Fig. 2B). Total GSH levels decreased by 29% with CSE and increased by 36% with NAC; regionally, CEP followed a similar trend without significant differences (Figs. 2C–D). ALP levels increased overall by 95% with CSE and decreased by 94% with NAC (Fig. 3A). Again, the CEP exhibited the most pronounced ALP response, rising by 419% and falling by 410%, compared to AF ( ↑ 64%, ↓ 68%) and NP ( ↑ 15%, ↓ 17%) (Fig. 3B). Cas-3 levels rose by 109% with CSE and dropped by 86% with NAC (Fig. 3C). Regionally, the CEP showed a 359% increase and 310% decrease, while NP and AF exhibited an increase of 157% and 64%, and decrease of 116% and 33%, respectively (Fig. 3D).

**Discussion**: Our in vitro findings demonstrate that CSE disrupts redox homeostasis in CEP by elevating oxidant levels and diminishing antioxidant defenses. This oxidative imbalance promotes CEP chondrocyte hypertrophy and apoptosis, as evidenced by elevating the levels of osteogenic markers. NAC co-treatment restored redox homeostasis by reducing ROS/RNS levels, restoring GSH content, and mitigating the osteogenic response. Notably higher ROS/RNS and ALP expression levels in CEP compared to AF and NP suggest distinct cellular responses, possibly driven by regional differences in IVD extracellular environment and intrinsic variations of cell phenotype. These results underscore the need for CEP-targeted interventions and highlight NAC's potential as a clinically accessible antioxidant to reduce CEP calcification.

**Reference**: [1] Malham GM, et al. Int J Spine Surg. 2021;15(6):1054-1059. [2] Nemoto Y, et al. J Orthop Sci. 2006;11(2):191-197. [3] Wang D, et al. Osteoarthr Cartil. 2012;20(8):896–905. [4] Kelley J, et al. JOR Spine. 2024;7(4): e70015. [5] Kelley J, et al. JBMR Plus. 2025;9(5): ziaf016. [6] Morita K, et al. J Exp Med. 2007;204(7):1613–23. [7] DiNicola ES, et al. J Orthop Res. 2024;42(8):1682–1695.

**Acknowledgement**: This work was supported by NIH/NIGMS COBRE grants: South Carolina Translational Research Improving Musculoskeletal Health (P20GM121342) and South Carolina Oxidants, Redox Balance and Stress Signaling (P20GM103542).

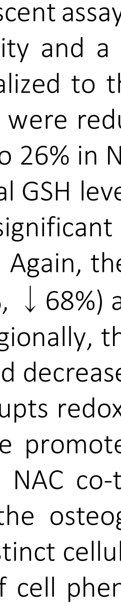

Figure 1. Quality control of cigarette smoke extract.
A. Optical density at 320 nm.
B. Nitrite concentration.

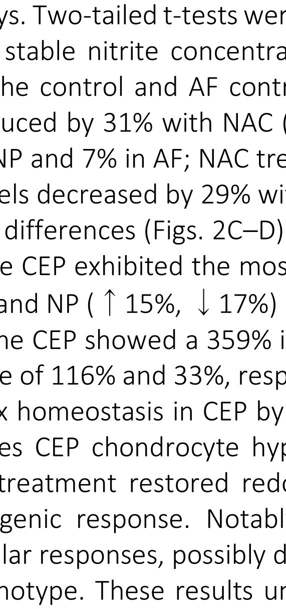

Figure 2. Levels of redox markers in IVD under different treatments.
A. Total ROS/RNS levels. B. Regional ROS/RNS levels.
C. Total GSH levels. D. Regional GSH levels.

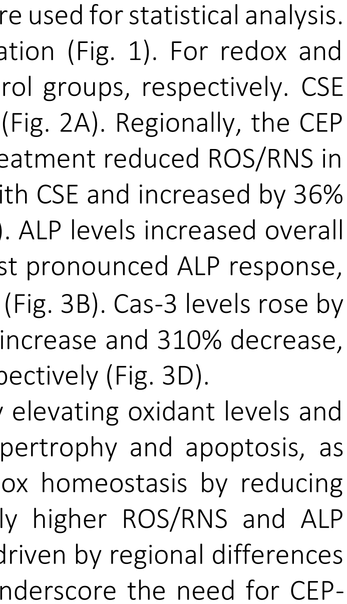

Figure 3. Levels of osteogenic markers in IVD under different treatments.
A. Total ALP levels. B. Regional ALP levels.
C. Total Cas-3 levels. D. Regional Cas-3 levels.

Notes (Figure 2 & 3): Data represent six IVDs per treatment group. * indicates a significant difference between control and CSE groups, while # indicates a significant difference between CSE and CSE+NAC groups.

**Long-Term Survival of Bioabsorbable vs. Metal Implants in Fracture Fixation**

### Aim

The aim of this paper is to compare the long-term survival, clinical outcomes, and complications associated with bioabsorbable and metal implants used in fracture fixation, with a focus on understanding the advantages and limitations of each type of implant in terms of durability, patient outcomes, and overall effectiveness.

### Objectives

1. To evaluate the long-term survival rates of bioabsorbable implants versus metal implants in fracture fixation.
2. To analyze the clinical outcomes, including fracture healing, complications, and the need for secondary surgeries, for both implant types.
3. To compare the biomechanical properties, such as strength, load-bearing capacity, and integration with bone tissue, of bioabsorbable and metal implants.
4. To review the incidence of complications like infection, implant failure, and irritation associated with bioabsorbable and metal implants.
5. To provide a comprehensive analysis of patient-specific factors that may influence the success or failure of each implant type.
6. To explore advancements in bioabsorbable and metal implant technologies and their implications for future clinical practices.

### Inclusion Criteria

1. **Study Types**: Randomized controlled trials (RCTs), cohort studies, observational studies, and meta-analyses comparing bioabsorbable and metal implants in fracture fixation.
2. **Population**: Studies involving adult patients (18 years and older) with fractures requiring surgical fixation, who have been treated with either bioabsorbable or metal implants.
3. **Intervention**: Studies focusing on fracture fixation with bioabsorbable or metal implants, including clinical outcomes and complications.
4. **Outcome Measures**: Studies reporting on long-term survival, healing rates, complication rates, implant failure, the need for removal surgery, and other relevant clinical outcomes.
5. **Follow-up Duration**: Studies with a follow-up period of at least 12 months to assess long-term survival and outcomes.

### Exclusion Criteria

1. **Study Types**: Case reports, small case series, studies without comparative data between bioabsorbable and metal implants, and non-peer-reviewed publications.
2. **Population**: Studies involving pediatric patients or individuals with comorbidities that significantly affect bone healing or implant survival
3. **Intervention**: Studies that do not specifically compare bioabsorbable and metal implants or those with insufficient details about the implant materials and design.

4. **Outcome Measures**: Studies that do not report on relevant long-term clinical outcomes, such as survival rates, fracture healing, or complications associated with the implants.

**PICO**

- **P** (Patient/Population):
  - Adult patients (18 years and older) with fractures requiring surgical fixation.
- **I** (Intervention):
  - Bioabsorbable implants (e.g., PLA, PGA, etc.) used for fracture fixation.
- **C** (Comparison):
  - Metal implants (e.g., titanium, stainless steel) used for fracture fixation.
- **O** (Outcome):
  - Long-term survival of the implants, fracture healing rates, complications (e.g., infection, failure, irritation), need for secondary surgeries (e.g., removal), and overall clinical outcomes.

### Key Words

- Bioabsorbable Implants- 504 sciendirect
- Metal Implants- 8 pubmed
- Fracture Fixation
- Implant Survival
- Long-Term Outcomes
- Complications in Fracture Fixation-
- Fracture Healing

1 paper clincial trial from pubmed

258- Cpmplication pubmed

Sciedirect- 2

### Introduction

- **Background of Fracture Fixation**: Importance of implants in bone healing; overview of fracture fixation methods.
- **Types of Implants**: Introduction to bioabsorbable and metal implants, including their properties, materials, and usage.
- **Objective**: To compare the long-term survival, complications, and clinical outcomes of bioabsorbable vs. metal implants in fracture fixation.

### 2. Fracture Fixation Implants

- **Bioabsorbable Implants**:
  - Material composition (e.g., PLA, PGA).

- Mechanism of absorption and its implications for fracture healing.
    - Clinical applications and advantages (e.g., no need for removal surgery).
- **Metal Implants**:
    - Common materials (e.g., titanium, stainless steel).
    - Characteristics and mechanical properties.
    - Long-term performance and need for removal surgery.

### 3. Comparison of Long-Term Survival

- **Healing and Integration**:
    - Effect of implant type on bone healing and remodeling.
    - Rate of fracture union with bioabsorbable vs. metal implants.
- **Longevity and Complications**:
    - Rate of complications (e.g., infections, implant failure, irritation).
    - Comparison of the risk of mechanical failure over time.
    - Need for secondary surgeries (e.g., removal or revision surgeries).
- **Biomechanical Properties**:
    - Strength and stability of implants during the healing phase and beyond.
    - Load-bearing capacity and biomechanical performance over time.

### 4. Clinical Studies and Evidence

- **Review of Published Studies**:
    - Overview of clinical trials, cohort studies, and meta-analyses comparing bioabsorbable and metal implants.
    - Focus on long-term outcomes, complication rates, and healing metrics.
- **Meta-Analysis**
    - Statistical analysis of survival rates, complication incidence, and functional outcomes.

### 5. Factors Affecting Long-Term Survival

- **Patient-Specific Factors**:
    - Age, bone density, health conditions, and comorbidities.
    - Influence of lifestyle and adherence to post-operative care.
- **Implant-Specific Factors**:
    - Material properties, design features, and the manufacturing process.
- **Surgical Factors**:
    - Surgical technique, implant placement accuracy, and post-operative management.

### 6. Advantages and Disadvantages

- **Bioabsorbable Implants**:
    - Advantages: No need for second surgery for implant removal, reduced risk of metal-related complications.
    - Disadvantages: Risk of premature absorption, lower mechanical strength during healing, possible inflammatory responses.
- **Metal Implants**:

- Advantages: High strength, established clinical track record, minimal risk of early failure.
    - Disadvantages: Need for second surgery to remove implants, potential for long-term irritation or adverse reactions, MRI compatibility issues.

### 7. Future Directions

- **Advances in Bioabsorbable Implants**:
    - New materials and improved bioabsorption rates.
    - Solutions to address mechanical strength and absorption timeline.
- **Innovations in Metal Implants**:
    - Development of biodegradable alloys or coatings to enhance bioabsorbability and reduce the need for removal.
- **Personalized Approaches in Implant Selection**:
    - Tailoring implant choices to patient characteristics

### 8. Conclusion

- **Summary of Key Findings**: Recap the comparison of bioabsorbable and metal implants in terms of long-term survival and clinical outcomes.
- **Clinical Implications**: Recommendations based on implant performance, complication rates, and patient outcomes.
- **Future Research**: Areas where further research is needed to refine implant materials, design, and clinical decision-making.

### Person 1: Introduction & Background (≈650 words)- sharvesh

- **Introduction**
    - Background of Fracture Fixation
    - Types of Implants
    - Objective
- This sets the stage and introduces key concepts, materials, and the aim of the paper.

---

### Person 2: Fracture Fixation Implants (≈650 words)- Jem

- **Bioabsorbable Implants**
    - Material composition
    - Mechanism of absorption
    - Clinical applications and advantages
- **Metal Implants**
    - Common materials
    - Characteristics and mechanical properties
    - Long-term performance and removal needs

---

### Person 3: Comparison of Long-Term Survival (≈700 words)- table sukha

- **Healing and Integration**
- **Longevity and Complications**
- **Biomechanical Properties**

This section is data-rich and may require deeper explanations, so it's slightly longer.

---

### Person 4: Clinical Studies and Evidence (≈650 words)- ayaan and arian

- **Review of Published Studies**
- **Meta-Analysis**

Should include specific study examples, statistics, and outcomes.

---

### Person 5: Factors Affecting Long-Term Survival & Advantages/Disadvantages (≈700 words)- tafheem

- **Patient-Specific Factors**
- **Implant-Specific Factors**
- **Surgical Factors**
- **Advantages and Disadvantages**
    - Of both implant types

This merges two logical sections that both focus on pros, cons, and influencing variables.

---

### Person 6: Future Directions & Conclusion (≈650 words)- shiona and aditi

- **Future Directions**
    - Advances in bioabsorbable & metal implants
    - Personalized approaches
- **Conclusion**
    - Summary of findings
    - Clinical implications
    - Future research

This wraps up the document with insights and vision.

### Spatial Proteomics via MALDI Mass Spectrometry Imaging Revealed Delayed Extracellular Matrix Remodeling in Aged Fracture Healing

Charles A. Schurman[1], William Chandler[1], Diane Hu[2], Nannan Tao[3], Ralph Marcucio[2], Peggi Angel[4], Birgit Schilling[1]

[1] Buck Institute for Research on Aging, Novato, CA, USA
[2] University of California San Francisco, San Francisco, CA, USA
[3] Bruker, San Jose, CA, USA
[4] Medical University of South Carolina, Charleston, SC, USA

Delayed fracture healing in the elderly is a common challenge that may lead to bone infection, nerve damage, and amputation. In fracture healing, bone formation is marked by the transition of extracellular matrix (ECM) proteins, including a progression from Collagens Type 2 and Type 3 that are eventually replaced by Collagen Type 1 in newly healed bone. It is unknown whether dysregulation of ECM proteins is associated with age-related delays in healing. Here, we investigate differential bone proteomics associated with delayed fracture healing. Changes in collagen and other ECM peptides in fractures of aged mice compared to young controls were evaluated using ECM-targeted Matrix-Assisted Laser Desorption/Ionization (MALDI) mass spectrometry imaging (MSI). Fracture calluses were collected from un-stabilized tibial fractures from 3 & 18 month-old c57B6 mice (N=4, ea). Formalin-fixed, paraffin-embedded tissue sections were treated sequentially with PNGase F and collagenase III using established MSI workflows that produce spatially localized ECM proteomes. Image data were acquired on a timsTOF fleX (Bruker) mass spectrometer with a laser step size of 20 µm. MS1 mass spectra were processed using the Bruker SCiLS Lab software. Retrieved tissue sections were stained with hematoxylin/eosin to identify callus regions. In young animals, a clear boundary was observed at the transition zone (TZ) (**Fig.1A**) between the newest cartilage-like callus and the matured mineralized bone that was defined by specific peptides for COL1A1, GPAGEEGKRGARGEP$^{(ox)}$ at m/z 1483.72, and COL2A1, GNRGFP$^{(ox)}$GQDGLA at m/z 1204.56 (**Fig. 1B**). The peptide for COL2A1 was most abundant in the most cartilage-like callus regions and the COL1A1 peptide most abundant in the matured mineralized bone along the outer edges of the callus. Overlay of signal from these two peptides shows the spatial gradient of ECM proteins in the callus matched known features from histologic staining such as the boundary of the TZ (**Fig.1C**) In fracture calluses from aged mice the cartilage marker peptide from COL2A1 was significantly higher than in the fracture calluses from young animals. Conversely, the more mature bone marker peptide of COL1A1 was consistently lower in aged fracture calluses than in young, an apparent delay in fracture healing in aged animals (**Fig.1D-E**). The regulation of these peptides were not unique in that Area Under the Receiving Operator Curve (AUROC) analysis further identified 41 unique m/z features that discriminated fracture calluses from young and old animals with AUROC > 0.75. Of these, 27 were significantly upregulated in young animals and 7 were upregulated in aged tissues ($p<0.05$). Of these peptides, many upregulated peptides in the young callus were identified as belonging to COL1A1 but also other proteins involved in fracture healing and callus remodeling such as the calcium chelator protein Calreticulin which can control a switch between osteogenesis and chondrogenesis. The aged callus had many peptides consistent with cartilage ECM including COL2A1 as well as Elastin and Lumican. The specific regulation of several ECM proteins between fracture calluses from young and aged animals putatively identifies a point during fracture healing where aged tissues fail to progress forward in osteochondral remodeling. Knowledge of ECM regulation during callus remodeling may aid in understanding disruptions in age-related fracture healing. Here we demonstrate the first use of MALDI-MSI on fracture calluses targeting ECM proteins that identifies a transition where aged bone exhibited reductions in healing capacity.

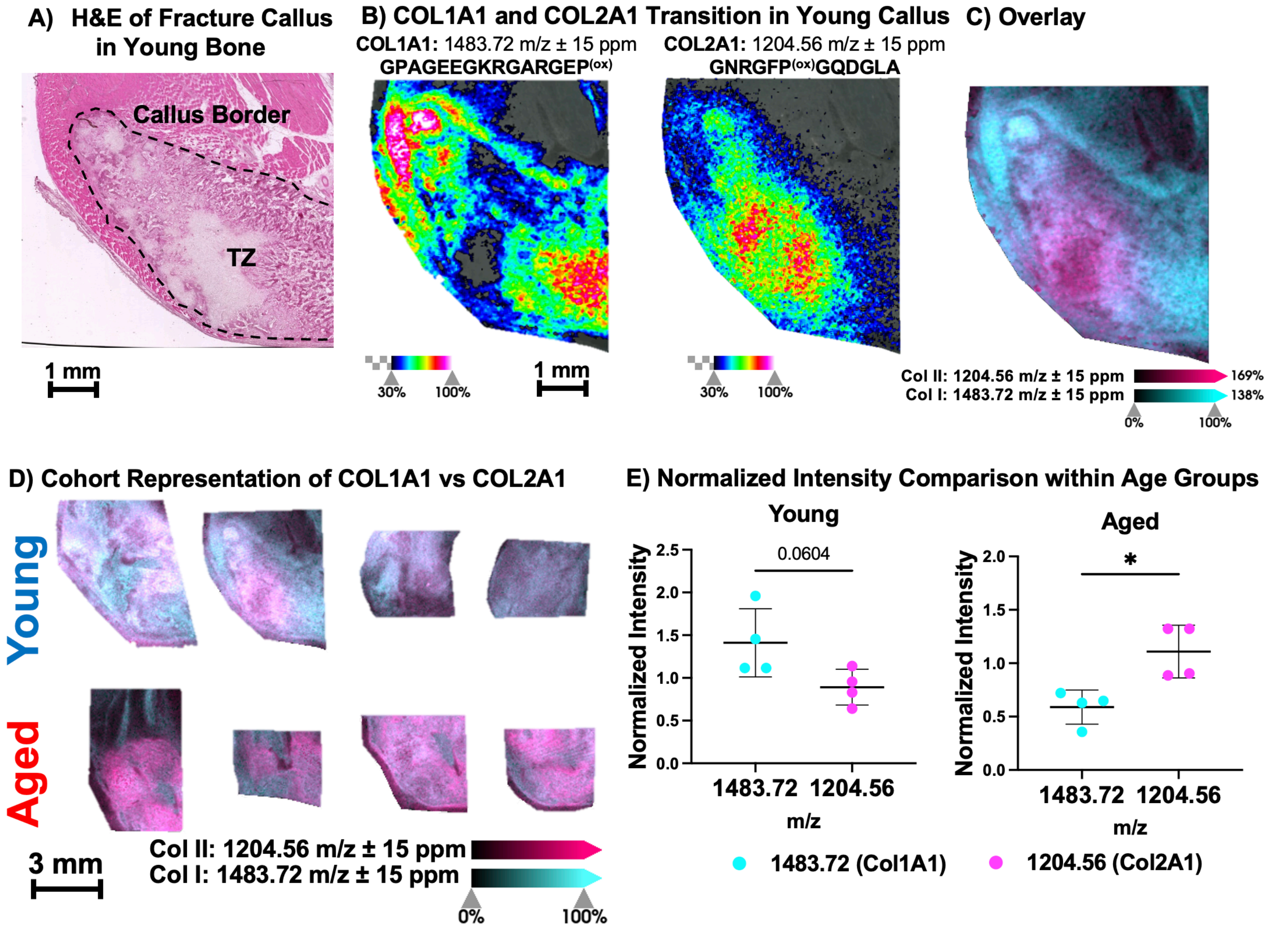

**Figure 1: Spatial Proteomic Mapping of Fracutre Callus: (A)** Hematoxylin/eosin (H&E) staining of a representative section of fracture callus from young mice (3 months) four weeks after un-stabilized tibial fracture depicting the boundary between healed bone at the callus border and the un-remodeled callus at the transition zone (TZ). **(B)** Mapping of bone and cartilage peptide markers from COL1A1 (m/z 1483.72) and COL2A1 (m/z 1204.56) respectively in fracture callus from young animals via Matrix-Assisted Laser Desorption/Ionization (MALDI) Mass Spectrometry Imaging (MSI) revealed the spatial distribution of matrix remodeling. **(C)** Overlay of signal from both COL1A1 and COL2A1 matched histologic features seen in H&E staining including the TZ. **(D)** Visualization of the peptides from COL1A1 and COL2A1 across 4 replicates for young and aged animals revealed consistently higher signal for cartilage markers in aged animals. **(E)** Quantification of the normalized feature intensity from MALDI-MSI showed a repression of COL2A1 signal in young animals compared to COL1A1 signal, where aged animals showed the opposite with a significant upregulation of the cartilage marker peptide of COL2A1, indicating a delay in callus remodeling.

### Pip5k1c deficiency causes aging-like musculoskeletal phenotypes in mice

*Xiaohao Wu, PhD[1], Mingjue Chen, MS[2], Minghao Qu, MS[2], Qinnan Yan, MS[2], Orr Sharpe, MS[1], William Robinson, MD PhD[1], Guozhi Xiao, PhD[2]*
*[1]Stanford University, Palo Alto, CA,[2]Southern University of Science and Technology, Shenzhen, China*

Phosphatidylinositol-4-phosphate 5-kinase type-1 gamma (PIP5K1C) is a pivotal lipid kinase responsible for generating phosphatidylinositol 4,5-bisphosphate (PIP2). PIP5K1C plays crucial roles in various cellular functions including focal adhesion formation, cell migration, and signal transduction.

Here we show that genetic deletion of Pip5k1c in musculoskeletal tissues/cells leads to multiple aging-like phenotypes in mice. Specifically, Pip5k1c deficiency in mesenchymal stem cells results in low-turnover osteopenia (Figure 1); in articular cartilage, it accelerates the development of osteoarthritis-like conditions (Figure 2); and in intervertebral discs, it fosters disc degeneration (Figure 3). Mechanistically, our findings suggest that Pip5k1c is essential for activating the AMP-activated protein kinase (AMPK) signaling pathway and preserving the extracellular matrix anabolism during aging (Figure 4). Of clinical importance, we observed that the absence of Pip5k1c diminishes the therapeutic efficacy of metformin, a known AMPK activator, in treating lumbar spine instability-induced disc lesions in adult mice (Figure 5).

Collectively, these results underline the critical role of Pip5k1c in maintaining cellular homeostasis across various tissues during aging and highlight the potential of targeting Pip5k1c in the treatment of aging-related diseases. Further investigations into the therapeutic potential of Pip5k1c are warranted.

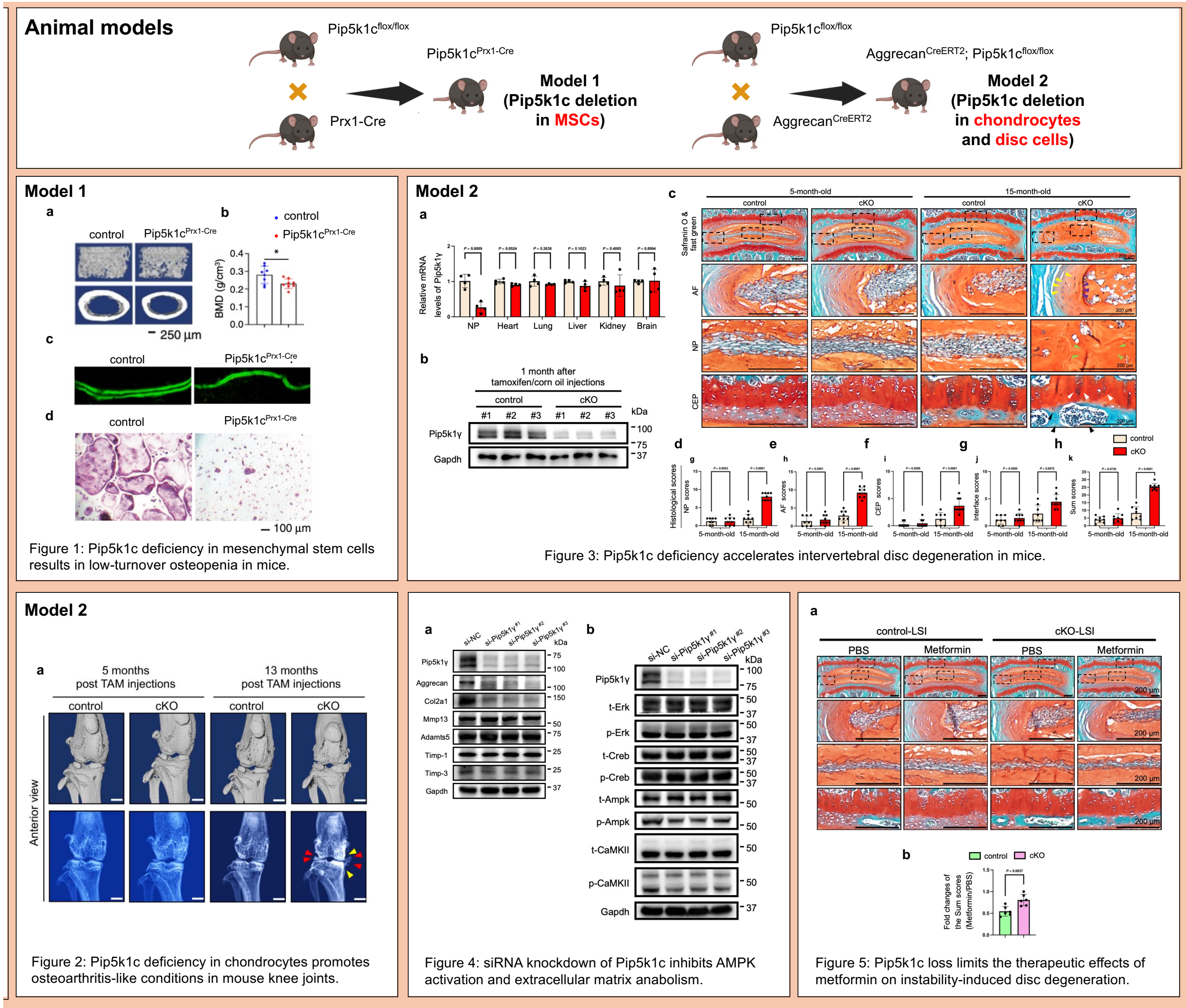

Figure 1: Pip5k1c deficiency in mesenchymal stem cells results in low-turnover osteopenia in mice.

Figure 3: Pip5k1c deficiency accelerates intervertebral disc degeneration in mice.

Figure 2: Pip5k1c deficiency in chondrocytes promotes osteoarthritis-like conditions in mouse knee joints.

Figure 4: siRNA knockdown of Pip5k1c inhibits AMPK activation and extracellular matrix anabolism.

Figure 5: Pip5k1c loss limits the therapeutic effects of metformin on instability-induced disc degeneration.

### Rejuvenation of synovial macrophage senescence via a senotherapeutic nanoparticle for the treatment of trauma- and aging-induced osteoarthritis

*Yuhang Liu, PhD[1], Xin Ma, PhD[1*]*
*[1]Department of Orthopaedics, Shanghai Sixth People's Hospital Affiliated to Shanghai Jiao Tong University School of Medicine, 600 Yishan Rd, Shanghai, 200233, China*
**Corresponding author*

Abstract:
Osteoarthritis (OA) is one of the most concerned aging-related diseases in the worldwide, yet the investigation of immune senescence in joint and related therapies are still poorly identified. Single-cell sequencing analysis and immunofluorescence of OA synovium reveal increased senescent macrophages in trauma-induced OA compared to controls, along with functional decline which might exacerbate OA. Hence, a novel senotherapeutic nanoparticle is developed using chloroquine (CQ)-bearing polymers (pCQ) for targeted delivery of superoxide dismutase (SOD) to synovial macrophages, termed as pCQ/SOD. The nanoparticle achieves efficient intracellular delivery of SOD to synovial macrophages. RNA-seq results reveal that pCQ/SOD nanoparticle inhibits macrophage senescence via p53 and cellular senescence signaling pathway, further reprograms M1-to-M2 repolarization. Furthermore, the delivered SOD inhibits BAX-dependent mitochondrial outer membrane permeabilization (MOMP) which further reduces mitochondrial DNA (mtDNA) release and secretion of senescence-associated secretory phenotype (SASP), while pCQ promotes macrophage efferocytosis against apoptotic cells via STAT3/ADAM17/MerTK signaling. As a result, intraarticular injection of pCQ/SOD nanoparticles in mice successfully alleviates not only trauma-induced OA, but aging-induced OA as well. The developed senotherapeutic nanoparticle in this study offers an effective approach for rejuvenation of synovial macrophage senescence and a promising therapeutic strategy for OA treatment.

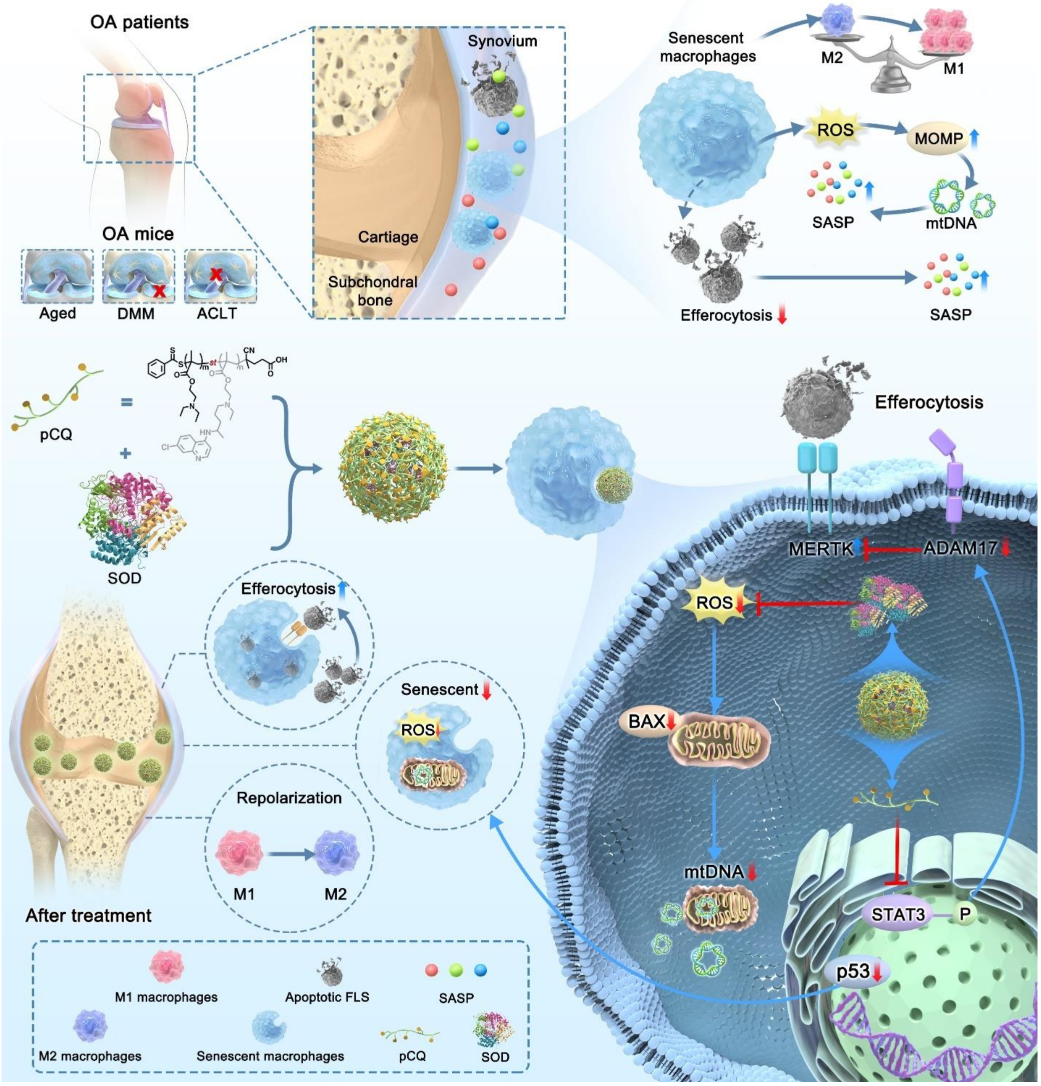

Fig1. Schematic illustration of the mechanisms of senotherapeutic nanoparticle in the treatment of OA

### Title- Preclinical Evaluation of Synthetic Bone Graft Materials in an Instrumented Sheep Posterolateral Lumbar Spinal Fusion Model

*Nathan William Kucko, PhD[1], Florence Barrère de Groot, PhD[1], Joost D. de Bruijn, PhD[1], Daniel Wills, BVSc[2] Tian Wang, PhD[2], Matthew Pelletier, PhD[2], William R. Wals, PhD[2]*

*Kuros Biosciences, Bilthoven, Netherlands[1], Surgical and Orthopaedic Research Laboratories, Prince of Wales Clinical School, University of New South Wales, Sydney[2]*

### Objective;
The objective was to evaluate the efficacy of commercially available synthetic bone grafts when used as standalone in a state-of-the-art instrumented sheep PLF model. The performance was measured using a comprehensive range of assessment tools.

### Summary of Background Data:
The amount of (pre)clinical data on the quality of spinal fusions using synthetic bone grafts are limited. Recently, a biphasic CaP with a needle-shaped submicron surface topography has demonstrated enhanced bone-forming ability driven by its surface structure triggering a favorable biological response from the innate immune system.

### Methods:
Six sheep underwent instrumented PLF surgery using the following bone grafts as standalone: (1) biphasic CaP with a needle-shaped submicron surface topography embedded in a collagen matrix (BCP<µm), (2) inorganic bone mineral with a synthetic peptide sequence in a hydrogel carrier (ABM/P15), (3) silicate-substituted CaP in a hydrogel carrier (nSiCaP), and (4) 45S5 Bio glass embedded in a collagen matrix (BG). After 12 weeks, spinal fusion was determined by manual palpation, radiograph and µCT imaging, range-of-motion mechanical testing, and histological and histomorphological evaluation.

### Results:
Radiography and µCT demonstrated superior fusion rates for BCP<µm compared to all other groups. Histology revealed significant graft resorption complemented by abundant bone tissue and continuous bony bridging between TPs in 3/3 implants for BCP<µm compared to only 1/3 implants for nSiCaP and 0/3 implants for ABM/P15 and BG. Histomorphometrically analysis, showed BCP<µm having the greatest new bone formation at 53 ± 2.2%.

### Conclusions:
These results show distinct differences in the performance between commercially available bone graft substitutes, highlighting the importance of utilizing clinically relevant animal models with robust, multi-endpoint analyses to evaluate their efficacy. Specifically, BCP<µm achieved superior fusion rates compared to other synthetic bone grafts when used standalone, further demonstrating its enhanced bone-forming ability in vivo.

### Spontaneous Calcium Signaling and Cell-Cell Communication in Human Articular Cartilage

*Ying Peng, MS, Annie Porter, MS, Michael Axe, MD, X. Lucas Lu, PhD*
*University of Delaware, Newark, DE, USA*

<u>INTRODUCTION</u> Intracellular calcium ($[Ca^{2+}]_i$) signaling plays an essential role in chondrocyte mechanotransduction. This study aimed to (1) characterize the spontaneous $[Ca^{2+}]_i$ signaling in healthy human cartilage and compare it with the late-stage osteoarthritis (OA) cartilage, (2) correlate the $[Ca^{2+}]_i$ signaling with the matrix synthesis rate of chondrocytes, and (3) test the presence of cell-cell communication among *in situ* chondrocytes through $[Ca^{2+}]_i$ signaling.

<u>METHODS</u> <u>*Tissue harvest and $[Ca^{2+}]_i$ imaging*</u>: Full-thickness cartilage samples ($\phi$ = 3 mm) were harvested from knee joints of healthy donors (4 male, avg. age = 38 yrs old) or total knee replacement (TKR) patients (12 female, 7 male, avg. age = 60 yrs old). After 2-day *in vitro* culture, samples were dyed with 5 µM Calbryte-520 (AAT Bioquest) to label the $[Ca^{2+}]_i$ and then imaged on a confocal microscope (Zeiss LSM 510) for 30 min at a frequency of 2 s/frame (Fig. 1). Calcium videos were processed with a custom-designed program to extract each cell's $[Ca^{2+}]_i$ transient and subsequently the spatio-temporal parameters of the $[Ca^{2+}]_i$ peaks. <u>*Matrix synthesis*</u>: Newly synthesized glycosaminoglycans (GAG) and collagen in cartilage samples were labelled during a 96-hour culture and quantified using a click chemistry technique we recently developed, with DNA content of each sample measured for normalization. <u>*$[Ca^{2+}]_i$ wave propagation between neighboring cells*</u>: The analysis focused on $[Ca^{2+}]_i$ active cells (n = 368 cells from 3 healthy donors, n = 672 cells from 3 TKR patients). For each active cell, the percentage of the six neighboring cells that displayed a $[Ca^{2+}]_i$ peak within 200 seconds following a peak of the active cell was calculated (Fig. 6a). Meanwhile, the same probability was calculated for six randomly selected faraway cells. Here we hypothesized that if $[Ca^{2+}]_i$ signaling in the active cell can propagate or induce $[Ca^{2+}]_i$ signaling in its neighboring cells, more coupled $[Ca^{2+}]_i$ signaling should be observed in the neighboring cells.

<u>RESULTS AND DISCUSSION</u> Spontaneous $[Ca^{2+}]_i$ signaling was observed in both healthy and TKR cartilage (Fig. 3a). Chondrocytes in OA tissues exhibit more vigorous $[Ca^{2+}]_i$ signaling, with more active cells, sharper and more frequent $[Ca^{2+}]_i$ peaks (Fig. 2, 3b). Both GAG and collagen synthesis rates in OA chondrocytes were higher than healthy cells (Fig. 4). Additionally, fingerprint-like features of $[Ca^{2+}]_i$ peaks were observed in chondrocytes (Fig. 5), *i.e.*, $[Ca^{2+}]_i$ peaks from a cell feature a unique and consistent shape. In healthy cartilage, within 200 seconds of a cell releasing a $[Ca^{2+}]_i$ peak, the neighboring cells had a significantly higher chance of having $[Ca^{2+}]_i$ peaks. This trend is also evident in the OA cartilage (Fig. 6b), indicating the presence of calcium wave propagation between neighboring chondrocytes in cartilage.

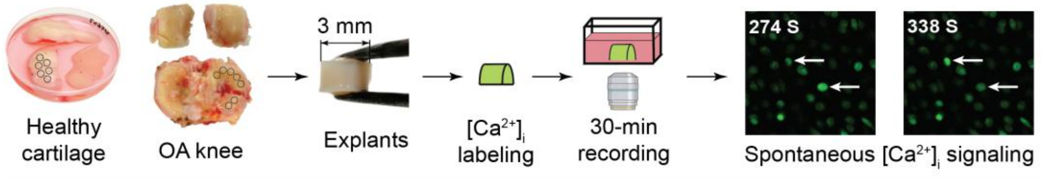

**Fig. 1: Workflow for cartilage sample harvesting and imaging of spontaneous $[Ca^{2+}]_i$ signaling in *in-situ* chondrocytes.**

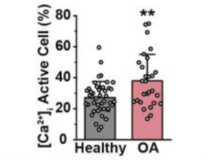

**Fig. 2: $[Ca^{2+}]_i$ active cell percentage.**

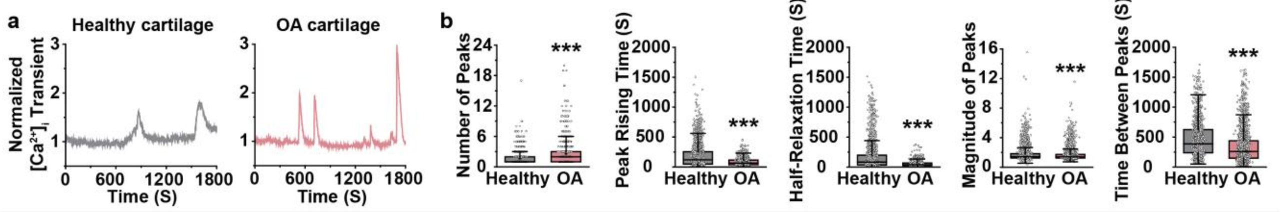

**Fig. 3: $[Ca^{2+}]_i$ signaling of healthy and OA cartilage. (a) Typical $[Ca^{2+}]_i$ transients from chondrocytes. (b) Spatial-temporal parameters of peaks.**

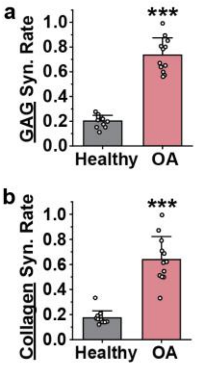

**Fig. 4: Cartilage matrix synthesis.**

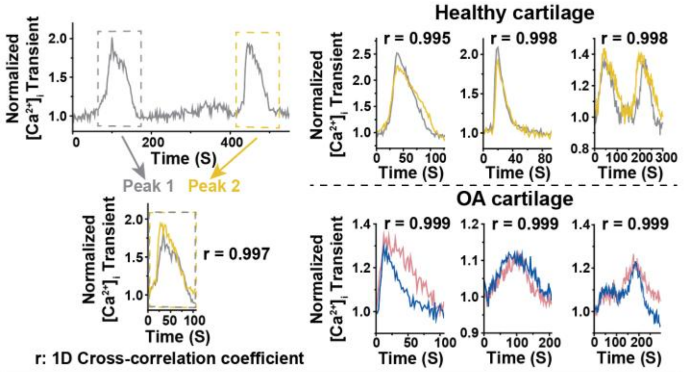

**Fig. 5: Fingerprint-like features of $[Ca^{2+}]_i$ peaks for each individual chondrocyte from healthy and OA cartilage.**

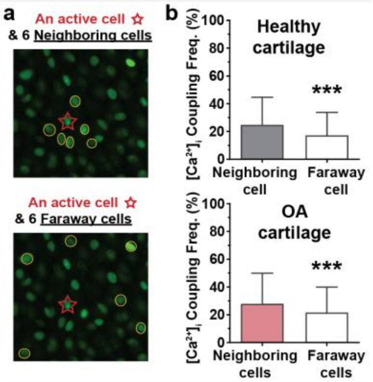

**Fig. 6: Intercellular communication among chondrocytes through $[Ca^{2+}]_i$.**

**Title: Mechanosensing activates Piezo1 channel and regulates the function of bone marrow peri-arteriolar Osteolectin$^{+}$ cells and osteogenesis**

**Author: Jiaming Guo, Bo shen**

Bone's ability to adapt to mechanical stress is a fundamental principle in orthopedics, stated by Wolff's Law, which posits that bone formation and strength are directly related to the degree of mechanical loading they experience. We have discovered that the mechanosensitive ion channel Piezo1 plays a crucial role in the osteogenic differentiation of bone marrow-derived LepR$^{+}$Osteolectin$^{+}$ cells (Osteolectin$^{+}$ cells), a process integral to bone formation. Specifically, we have shown that exercise-induced mechanical stimulation activates Piezo1, thereby promoting bone formation and reinforcing the relevance of Wolff's Law in the context of peri-arteriolar stromal cells. Despite Piezo1's broad expression across LepR$^{+}$ cells, including peri-sinusoidal LepR$^{+}$Oln$^{-}$ cells, the precise reason for the dependency of peri-arteriolar Osteolectin$^{+}$ cells on Piezo1 for their function is not fully understood. To address this, we developed *Piezo1-GCaMP* mice, which feature a genetically encoded fluorescent probe allowing for non-invasive optical monitoring of Piezo1 activity. Our studies revealed a significant increase in Piezo1 activation in peri-arteriolar LepR$^{+}$Osteolectin$^{+}$ cells, compared to peri-sinusoidal cells, highlighting the selective role of mechanosensing in these cells. Importantly, we observed that weight-bearing bones, which are more responsive to mechanical loading, have a higher frequency of Osteolectin$^{+}$ cells and are more affected by the conditional deletion of Piezo1 in LepR$^{+}$ cells, leading to a loss of bone mineral density and cortical bone thickness. Our ongoing work is focused on uncovering the molecular mechanisms downstream of Piezo1 that contribute to these observations, with a hypothesis that peri-arteriolar Osteolectin$^{+}$ cells exhibit increased mechanosensitivity due to enhanced matrix protein deposition and local stiffness near arterioles. This research underscores the critical importance of Piezo1-mediated mechanosensing in peri-arteriolar Osteolectin$^{+}$ cells for bone formation, particularly in weight-bearing bones, which have a higher mechanosensitivity and a greater abundance of Osteolectin$^{+}$ cells.

**Is Surgical Repair of the Deltoid Ligament Necessary in Ankle Fractures? A Systematic Review and Meta-Analysis**

Andy Suarez[1] , Taylor Checkley[1] , Alejandra Rinaldi[2], Leon Liu[1] , Pradith Santapur[1] , Iman Fakhoury[1] , James Ro[1] , Christian Palacios[1] , Mariafe Reyes[1] , Gary B. Schwartz[1] MD

[1] Nova Southeastern University, Dr. Kiran C Patel College of Allopathic Medicine, Fort Lauderdale, FL, USA.
[2] Nova Southeastern University, Dr. Kiran C Patel College of Osteopathic Medicine, Fort Lauderdale, FL, USA.

**INTRODUCTION:**
The deltoid ligament stabilizes the ankle by anchoring the medial malleolus to the tibia and adjacent tarsal bones, preventing excessive rotation. In ankle fractures, this ligament is frequently ruptured, and the optimal treatment, surgical repair versus conservative management, remains debated. This study systematically reviews current literature comparing functional and postoperative outcomes between the two approaches.

**METHODS:**
A systematic search was conducted in PubMed, Embase, Web of Science, and the Cochrane Library for prospective, randomized controlled trials involving adult patients. Screening and selection were performed using Covidence software, with two independent reviewers and a third to resolve conflicts. Five studies met inclusion criteria and assessed outcomes using the American Orthopedic Foot and Ankle Hindfoot Score (AOFAS), complication rates, and return-to-work timelines.

**RESULTS:**
Meta-analysis of five studies revealed no statistically significant difference in AOFAS scores between repair and non-repair groups (MD 4.57; 95% CI, -3.03 to 12.17; $p = 0.24$). However, pooled complication rates were significantly lower in the repair group (RR 0.43; 95% CI, 0.24–0.77; $p = 0.004$). No significant difference in return-to-work time was observed (RR 1.02; 95% CI, 0.65–1.60; $p = 0.93$).

**DISCUSSION & CONCLUSION:**
While functional outcomes and return-to-work times were similar between groups, surgical repair was associated with significantly fewer postoperative complications. These findings suggest that deltoid ligament repair may reduce the risk of adverse events in the management of ankle fractures. Further large-scale randomized trials are needed to strengthen the evidence base and guide clinical practice.

### Scaffold-Free Cartilage Regeneration Using Electrosprayed Adipose-Derived Stem Cells for Growth Factor-Free Chondrogenesis in a Preclinical Model

*Gary Coffman, Madison Higgins, Xavier Lewis, Nasim Nosoudi*

*Department of Biomedical Engineering, College of Engineering and Computer Sciences, Marshall University, Huntington, WV, USA*

**Abstract:**

**Background:**
Cartilage injuries present a significant clinical challenge due to the limited regenerative capacity of articular cartilage and the shortcomings of current repair strategies, which often rely on exogenous growth factors or scaffold materials. Adipose-derived stem cells (ADSCs) represent a promising autologous source for cartilage repair, yet scalable, growth factor-independent differentiation strategies remain underdeveloped.

**Objective:**
This study introduces a novel, scaffold-free approach for cartilage regeneration by utilizing electrosprayed ADSCs that undergo in vitro chondrogenic differentiation without exogenous biochemical inducers.

**Methods:**
Human ADSCs were electrosprayed to promote biophysical stimulation and cultured under defined, serum-free conditions for 14–21 days to induce chondrogenesis. Differentiated cells were characterized by morphology, viability, and expression of chondrogenic markers (Sox9, COL2A1, and Aggrecan). The therapeutic potential of these cells was evaluated in a rabbit model of full-thickness cartilage defects. Cells were delivered intra-defect using a fibrin-based carrier to ensure retention.

**Results:**
Electrospray-induced ADSCs exhibited robust chondrogenic differentiation, with significantly reduced phenotypic heterogeneity compared to chemically induced counterparts. In vivo, treated defects demonstrated improved cartilage matrix deposition, superior integration with host tissue, and enhanced mechanical properties relative to controls.

**Conclusion:**
This study demonstrates that electrospraying can drive functional, growth factor-free chondrogenesis of ADSCs and support effective cartilage repair in vivo. The findings highlight a scalable, cost-effective, and translationally relevant platform for autologous cartilage regeneration.

**Stability at a Cost: Evaluating Postoperative Complications in Bioabsorbable vs. Metallic Syndesmotic Fixation**

Leon Liu[1] , Andy Suarez[1], Taylor Checkley[1], Samir Sakaria[1], Alejandra Rinaldi[2], Christian Palacios[1] , Pradith Santapur[1] , Gary B. Schwartz MD[1]

[1] Nova Southeastern University, Dr. Kiran C Patel College of Allopathic Medicine, Fort Lauderdale, FL, USA.
[2] Nova Southeastern University, Dr. Kiran C Patel College of Osteopathic Medicine, Fort Lauderdale, FL, USA.

**Introduction:**

Bioabsorbable screws for syndesmotic fixation in complex ankle fractures have become increasingly popular as they may eliminate the need for secondary surgeries for implant removal. However, their efficacy when compared against metallic screws is unclear with some concerns persisting regarding higher complication rates compared to the traditional metallic option.

**Methods:**

A systematic search was conducted using the PubMed and Google Scholar databases, yielding 315 studies. After title and abstract screening followed by full-text review, eligible randomized controlled trials and prospective studies comparing bioabsorbable versus metallic screw fixation in ankle syndesmotic injuries were included. The primary outcome assessed was the total rate of postoperative complications. Risk ratios (RR) and 95% confidence intervals (CI) were calculated.

**Results:**

The pooled analysis revealed a significantly higher rate of complications in the bioabsorbable screw group compared to the metallic group (RR = 3.35; $p = 0.0003$).

**Conclusion:**

Bioabsorbable screws were associated with a significantly increased complication rate, raising concerns about their routine use in syndesmotic fixation. Although previous studies have suggested comparable functional outcomes and fewer reoperations due to implant removal (1–4), the current data underscore the importance of prioritizing safety and reliability. Given the increased risk of inflammatory reactions, hardware failure, and delayed local symptoms seen in bioabsorbable groups, metallic screws remain the more dependable option. Future research should focus on improving the biomechanical and biocompatibility profiles of absorbable materials before they are widely adopted in clinical use.

**Generation of endogenous structure of articular cartilage using chondrocyte activating peptide drug CK2.1 for the treatment of Osteoarthritis**

*Venu Pandit, Anja Nohe*
*University of Delaware, Newark, DE*

Peptide drugs are promising candidates for targeted treatment without the danger of immunogenicity and toxicity associated with monoclonal antibodies and small-molecule drugs, respectively. Osteoarthritis is a debilitating condition with an ever-increasing incidence rate in the aging population. A synthetic peptide drug, CK2.1, has reversed the symptoms of surgically induced Osteoarthritis in a mouse model. The drug was delivered by local systemic injections of CK2.1 conjugated to hydrogel particles (CK2.1-HGP) for sustained release. We investigated the mechanism of uptake of CK2.1 in the knee articular cartilage extracellular matrix and the primary chondrocytes. We used the paraffin-embedded tissue section of the knee cavity where the OA was induced, and the drug was injected intraarticularly. With the immunofluorescence technique, we show that the peptide CK2.1 accumulates in the superficial and middle zone of the hyaline cartilage. There is an increase in the expression of cartilage-specific markers SOX9 and Type IX collagen—localization of the peptide and these two markers highly overlap. Intracellular localization of the peptide is seen to be within the cytoplasm. Finally, with the blockage of endocytosis by Clathrin-coated pits or caveolae or both, there is a reduction in the uptake of CK2.1 in C3H10T1/2 cells. Hence, the drug has a highly targeted activation of articular chondrocytes and can activate chondrogenesis. We intend to create a robust 3-dimensional system to recreate the endogenous structure of articular cartilage ex vivo with the regenerative ability of the underlying drug. We use a hyaluronic acid-based culture system to achieve that.

### Understanding Bone Biomechanics and Pathology Through Osseosurface Electronics

*Gerardo Figueroa Romero[1,2], Kevin A. Kasper[2], Dania L. Perez[2], Avery M. Miller[2], David A. Gonzales[1], Jesus A. Siqueiros[2], Philipp Gutruf, PhD[2], David S. Margolis, MD, PhD[1,2]*

*[1]University of Arizona Department of Orthopedic Surgery, Tucson, AZ, [2]University of Arizona Department of Biomedical Engineering, Tucson, AZ*

**Introduction:** Bone mass and remodeling is heavily regulated by mechanical strain. Strain gauges are the gold standard for evaluating *in vivo* bone strain. Despite successful attempts in achieving long-term bonding between bone and strain gauges, long-term evaluation of bone strain is limited due to bulky and expensive telemetry units. We have recently reported the development of a wireless, battery-free, multimodal platform we call osseosurface electronics (OE)[1]. OE devices are made of a thin flexible substrate offering strain and temperature sensing capabilities. Furthermore, OE devices can be coupled with 3D printed wearable technology for continuous communication[2]. In this study, we demonstrate the utility of OE devices for long-term strain measurements and its use in fracture healing monitoring.

**Methods:** All animal experiments were performed under approved IACUC protocols. OE devices were manufactured and prepared as previously reported[1]. Six sheep were used for long-term *in vivo* evaluation of bone strain parameters. Up to two OE devices were placed on each bone: two on the proximal and distal femoral diaphysis and two on the medial and lateral tibia (**Figure 1A**). Animals were walked weekly on a custom treadmill. A custom-made wearable was placed on the limb to interface with the OE device on the lateral size of the proximal tibia (**Figure 1B**). The animal was left to walk voluntarily for continuous data acquisition without human intervention.

In our fracture healing sheep model, two OE devices were secured proximal and distal to the fracture site and the bone was fixed with a modified intramedullary nail (**Figure 1D**). Weekly radiographs were taken to observe fracture healing progression. The animal was walked on a treadmill to observe strain changes during healing. The peak-to-peak strain was calculated as the delta change between the highest and lowest points of compressive strain in one gait cycle.

**Results:** Implantation of OE devices showed no negative effect on the animal's natural gait. Wearable technology demonstrated reliable power and communication with OE devices for long-term data collection. Representative bone strain data during treadmill walking is presented in **Figure 1C**, demonstrating the increase and decrease in compressive strain during locomotion with an average peak to peak strain of 125+/-8 με. *Ex vivo* analysis demonstrated excellent device biocompatibility with no effects on bone mechanical and structure properties (Data Not Shown).

Radiographic images demonstrated evident changes in the bony callus through the duration of the study (**Figure 1E**). The peak-to-peak strain decreased during healing, plateauing between week 9 and 10, and increasing until week 12 (**Figure 1F**).

**Discussion:** OE devices combined with wearable technology allow for long-term reliable bone strain data in a large animal model up to 11 months. In addition, OE devices offer precise measurements with a high sampling rate for reliable representation of bone strain during locomotion. In a fracture healing study, OE devices demonstrate significant changes in *in vivo* bone strain, demonstrating a quantitative method of monitoring fracture healing.

**References: [1]**. Cai L, Burton A, Gonzales DA, et al. Osseosurface electronics—thin, wireless, battery-free and multimodal musculoskeletal biointerfaces. *Nat Commun*. 2021;12(1):1-12. doi:10.1038/s41467-021-27003-2, **[2]** Kasper KA, Romero GF, Perez DL, et al. Continuous operation of battery-free implants enables advanced fracture recovery monitoring. *Science Advances*. 2025;11(19):eadt7488. doi:10.1126/sciadv.adt7488

**Acknowledgements:** We acknowledge the support of the University of Arizona Technology and Research Initiative Fund (TRIF), DOD W81XWH-22-PRORP-ARA, and NIH 1R01AR082394-01A1.

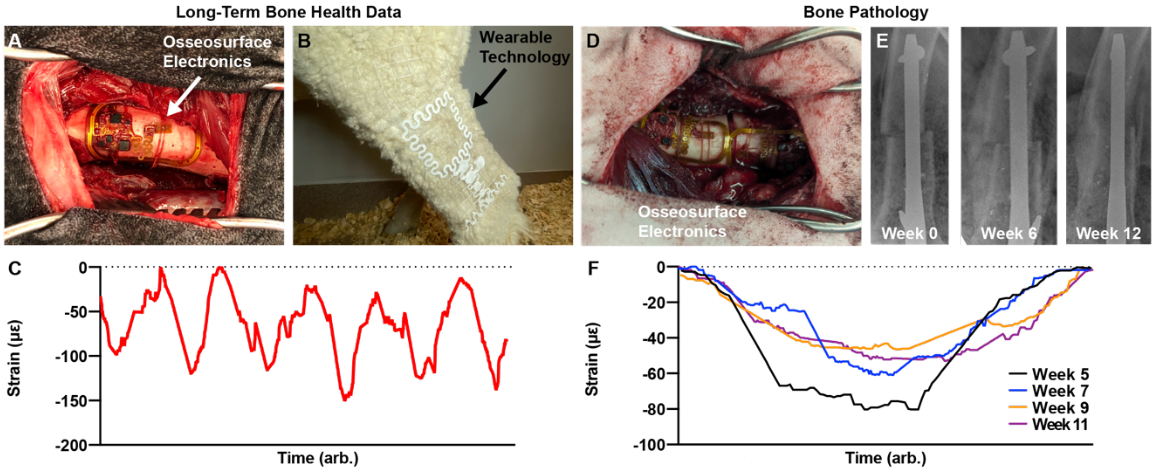

### Diffusion Tensor Imaging of the Degenerating Human Meniscus

*Nataliya Tod(BS)[1], Luisa de Ro(BS)y[2,] Sarah Salem(BS)[3], Jie Chen(PhD)[1], Rui Hu(MS)[1], Saira Tabassam(MS)[1], Zhuoheng Liu(MS)[1], Fang Liu(PhD)[4], Yang Xia(PhD)[3], Andreas Martin Seitz(PhD)[2], Nian Wan (PhD)[1,]*
*[1]UTSW, Dallas, TX, USA, [2]Ulm Univ, Ulm, Germany, [3]Oakland Univ, Rochester, MI, USA, [4] MGH, Charlestown, MA, USA*

**Introduction**: The meniscus' role in shock absorption, joint stabilization and cartilage protection is made possible by highly organized collagen fiber networks. Damage to this leads to osteoarthritis (OA). While OA is rising due to obesity and an aging population, exact prevalence is unknown due to discrepancies between clinical and radiographic findings[1]. Moreover, no current therapy exists to regenerate lost tissue, highlighting the necessity for better understanding of OA progression. There is a need for a noninvasive, sensitive modality to characterize structural changes during this progression. Diffusion MRI emerged as an advanced tool for revealing tissue microstructure in soft tissues. Recently, diffusion tensor imaging (DTI) has enabled even more detailed information about structural connections, like 3D fiber network through whole collagenous tissue[2, 3]. Our goal was to investigate microstructural details of human menisci at various stages of degeneration with DTI.
**Methods:** 16 ex-vivo human menisci were fixed and scanned on a 9.4T small animal MRI. 3D diffusion-weighted spin-echo pulse sequence was performed (TR = 100 ms, Matrix size = 106 × 50 × 34, FOV= 42.4 ×20 × 13.6 mm3, TE = 13.9 ms, 400 µm isotropic spatial resolution, b value = 500, 750, and 1000 s/mm2) with 41 diffusion gradient encoding directions and 4 b0 measurements. Gradient separation time was 7.6 ms; diffusion gradient duration was 2.6 ms. Scan time was 6 hours for each specimen during which temperature fluctuation was < 1 ºC. The DTI model was employed to calculate fiber direction and scalar indices: fractional anisotropy (FA), mean diffusivity (MD), axial diffusivity (AD), radial diffusivity (RD). Gross pathology, histology, and polarized light microscopy (PLM) was performed to compare with MRI findings. Finally, quantitative analysis was performed by dividing menisci into three regions and using the Spearman correlation method on Pauli degeneration scores, DTI metrics, and biomechanical tests (thickness, instantaneous modulus (IM), and stiffness (K)).
**Results:** Figure 1 shows representative results of moderately and severely degenerated menisci. Compared to the moderate sample, the severe sample had a more inhomogeneous appearance on gross pathology (a) and more disruptions in collagen, as evidenced by PSR staining (b). It also had a more disorganized fiber orientation (white arrows), as represented by 3D collagen fiber architecture (tractography, c), confirmed by structure tensor analysis (d) and PLM (e). The three-region correlation analysis revealed that degree of degeneration (Pauli score) was negatively correlated with FA ($p < 0.001$) and positively correlated with MD, AD, and RD ($p < 0.001$). Additionally, FA had a mild negative correlation with thickness and mild positive correlation with stiffness while MD, AD, and RD showed the inverse result (Figure 2).
**Discussion:** Previously, our team successfully used diffusion imaging to trace collagen architecture in porcine menisci[4]. Here, we extend that success to *human* menisci and show that DTI can distinguish different stages of degeneration. The structural breakdown detected by DTI was bolstered by PLM and structural tensor analysis that visually confirmed disruptions in fiber orientation. Given that compromised tissue would result in changes to water diffusion, it is coherent that more ordered (healthy) menisci have higher FA and lower MD, AD, and RD than these same tissues after chronic wear and tear. When even minor structural breakdown of collagen tracts occurs, this eliminates the highly restricted paths possible for water molecules. DTI offers a powerful, non-invasive, and non-destructive approach to study subtle meniscal damage. This technique emerges as a promising tool for more accurate pathological assessment and potentially guiding future treatment strategies in patients with degenerative joint disease.
**References:** (1) Litwic A, et al. *Br Med Bull*. 2013; 105:185-199. (2) Raya J, et al. *J Magn Reson Imag*. 2024; 59: 376-396. (3) Shen et al., *Front Endocrinol*. 2022; 13: 876784. (4) Zhao Q, et al. *Quant Imag Med Surg*. 2024; 14(4): 2738-2746.

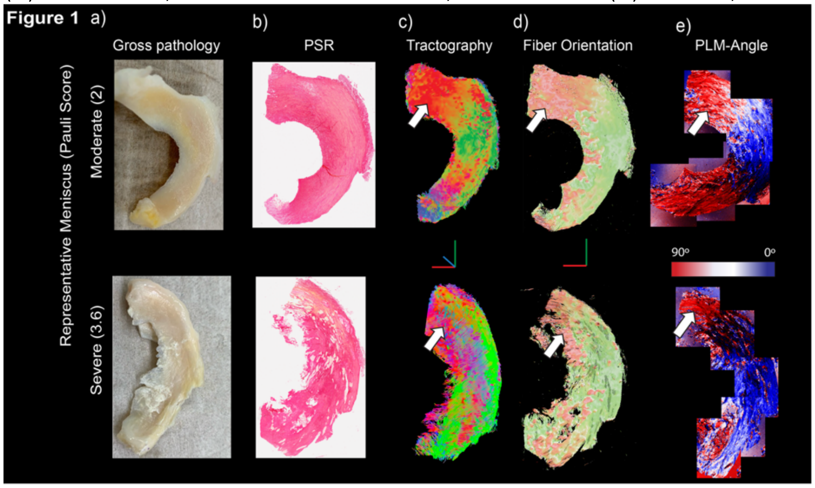

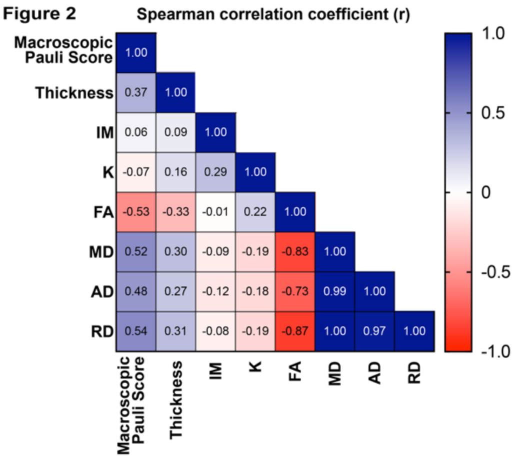

**Correlation of Patient-Reported and Performance-Based Outcomes Following Total Knee Arthroplasty: A Retrospective Study with a Narrative Review of Adipokine-Mediated Extracellular Matrix Dysregulation in Knee Osteoarthritis**

### Authors:

*Indel T. Niles, MD MSc[1], Narjis Aji, MD[2], Samantha L. Mathurin, MD , PgDip[3] Shawn Robbins, PhD[4]*

[1]Department of Surgical & Interventional Sciences, McGill University, Montreal Quebec Canada

[2]Department of Experimental Medicine, McGill University, Montreal, Quebec, Canada,

[3]Owen King European Union Hospital-Millenium Heights Medical Complex, Castries, St. Lucia, WI

[4] School of Physical therapy & Occupational Therapy, McGill University, Montreal, Quebec

### Abstract

Background: Obesity is a well-established risk factor for knee osteoarthritis (OA), traditionally attributed to excessive mechanical loading. However, recent evidence suggests that adipokines—cytokines secreted by adipose tissue—play a critical biochemical role in OA pathogenesis by promoting extracellular matrix (ECM) degradation and joint inflammation. Leptin has been shown to upregulate matrix metalloproteinases (MMPs), accelerating cartilage breakdown. This study examines the relationship between body mass index (BMI) and postoperative functional outcomes following total knee arthroplasty (TKA), supported by a narrative review that establishes the theoretical framework and biological plausibility of adipokine-mediated influences on surgical recovery.

Methods: A retrospective cohort analysis was performed using data from 27 patients enrolled in a TKA implant design trial. Functional outcomes were assessed at 1 and 2 years postoperatively using the Knee Injury and Osteoarthritis Outcome Score (KOOS) and three performance-based measures: the 6-Minute Walk Test (6MWT), 11-Second Stair Climb Test (11SCT), and 30-Second Chair Stand Test (30CST). Correlation and regression analyses were conducted to assess associations between BMI and outcome measures. To contextualize the findings, a narrative literature review was conducted using PubMed/MEDLINE, EMBASE, and Scopus databases. A manual search strategy identified studies published between 2000 and 2024 that examined the roles of leptin, adiponectin, resistin, interleukin-6 (IL-6), and C-reactive protein (CRP) in OA-related ECM remodeling and systemic inflammation.

Results: Higher BMI was significantly associated with poorer performance on the 6MWT ($r = -0.44$, $P < .05$) and longer completion time for the 11SCT ($r = 0.42$, $P < .05$), independent of age. Strong correlations were observed between KOOS Pain and KOOS Activities of Daily Living (ADL) ($r = 0.71$, $P < .01$). Moderate correlations were identified between KOOS subscales and performance measures, including KOOS ADL and 30CST ($r = 0.56$, $P < .05$), KOOS Pain and 30CST ($r = 0.59$, $P < .05$), and KOOS Pain and 11SCT ($r = -0.44$, $P < .05$).

Discussion: The narrative review highlighted the emerging role of adipokines—particularly leptin, adiponectin, and resistin—in osteoarthritis pathogenesis. These cytokines, secreted by adipose tissue, contribute to extracellular matrix (ECM) degradation and synovial inflammation by promoting matrix metalloproteinase activity and increasing pro-inflammatory mediators such as interleukin-6 (IL-6) and C-reactive protein (CRP). Mechanistically it provides a strong theoretical basis for the hypothesis that elevated BMI impairs joint health not only through mechanical loading, but also via metabolic inflammation.

The retrospective study findings align with this framework, demonstrating that higher BMI was significantly associated with worse performance-based and patient-reported functional outcomes following total knee arthroplasty (TKA). The integration of these data suggests that adipokine-mediated pathways may underlie the observed impairments in recovery, reinforcing the need to consider obesity as a systemic, inflammatory condition in orthopedic outcomes. These insights support future research into biomarker profiling to inform risk stratification and tailored rehabilitation strategies.

Conclusions: The findings highlight the multifactorial impact of obesity on TKA outcomes, implicating both biomechanical and biochemical mechanisms. The accompanying literature review underscores the role of adipokines in ECM dysregulation, supporting the role of Integrating metabolic and inflammatory profiling into preoperative evaluations to improve risk stratification and inform personalized rehabilitation strategies. Future research should incorporate prospective measurement of adipokines and systemic inflammatory markers such as IL-6 and CRP to elucidate their roles in TKA recovery and support the development of biomarker-informed therapeutic interventions.

### References

1. Roos EM, Lohmander LS. The Knee injury and Osteoarthritis Outcome Score (KOOS): from joint injury to osteoarthritis. Health Qual Life Outcomes. 2003;1:64. doi:10.1186/1477-7525-1-64
2. Kennedy DM, Stratford PW, Riddle DL, Hanna SE, Gollish JD. Assessing recovery and establishing prognosis following total knee arthroplasty. Phys Ther. 2008;88(1):22-32. doi:10.2522/ptj.20070040
3. Fortin PR, Clarke AE, Joseph L, et al. Outcomes of total hip and knee replacement: preoperative functional status predicts outcomes at six months after surgery. Arthritis Rheum. 1999;42(8):1722-1728. doi:10.1002/1529-0131(199908)42:8<1722::AID-ANR11>3.0.CO;2-R
4. Pua YH, Clark RA, Ong PH. Evaluation of the Wii Balance Board for walking aids prediction: proof-of-concept study in total knee arthroplasty. PLoS One. 2015;10(1):e0117124. doi:10.1371/journal.pone.0117124
5. Lingard EA, Katz JN, Wright EA, Sledge CB; Kinemax Outcomes Group. Predicting the outcome of total knee arthroplasty. J Bone Joint Surg Am. 2004;86(10):2179-2186. doi:10.2106/00004623-200410000-00015
6. Scher JU, Pillinger MH. The anti-inflammatory effects of prostaglandins. Curr Opin Pharmacol. 2005;5(4):405-411. doi:10.1016/j.coph.2005.03.010
7. Gualillo O, Lago F, Gómez-Reino J, Dieguez C. Adipokines as emerging mediators of immune response and inflammation. Nat Clin Pract Rheumatol. 2007;3(12):716-724. doi:10.1038/ncprheum0674
8. de Boer TN, van Spil WE, Huisman AM, et al. Serum adipokines in osteoarthritis: comparison with controls and relationship with local parameters of synovial inflammation and cartilage damage. Osteoarthritis Cartilage. 2012;20(8):846-853. doi:10.1016/j.joca.2012.04.001
9. Collins KH, Herzog W, MacDonald GZ, et al. Obesity, metabolic syndrome, and musculoskeletal disease: common inflammatory pathways suggest a central role for loss of muscle integrity. Front Physiol. 2018;9:112. doi:10.3389/fphys.2018.00112
10. Griffin TM, Guilak F. The role of mechanical loading and obesity in the onset and progression of osteoarthritis. Exerc Sport Sci Rev. 2005;33(4):195-200. doi:10.1097/00003677-200510000-00008
11. Zhao X, Shah D, Gandhi K, et al. Clinical, humanistic, and economic burden of osteoarthritis among noninstitutionalized adults in the United States. Osteoarthritis Cartilage. 2019;27(11):1618-1626. doi:10.1016/j.joca.2019.07.002

### Preliminary Outcomes of a Novel Biologic Proximal Interphalangeal Joint Arthroplasty Technique Using Meniscus Allograft

*Naomi Gadinsky, MD[1], Theodore Brown, BA[1], Seena Sebt, BA[2], Stuart Kuschner, MD[1], Ryan DellaMaggiora, MD[1], Eugene Tsai, MD[1], Justin Koh, MD[1], Meghan McCullough, MD[1], David Kulber, MD[1]*
*[1]Cedars-Sinai Medical Center, Los Angeles, CA, USA, [2]UC Riverside School of Medicine, Riverside, CA, USA*

**Intro:** Proximal interphalangeal (PIP) joint arthritis of the hand can cause significant pain, disability, and negative impacts on quality of life. Patients who fail nonoperative treatment historically have turned to arthrodesis or silicone arthroplasty for management of their disease. We have devised a novel biologic joint resurfacing technique using meniscus allograft to alleviate pain, restore motion, and avoid synthetic implant use in patients with arthritis of the PIP joint. The purpose of this study is to report the preliminary outcomes of this technique.

**Methods:** A retrospective review was performed of all patients who underwent PIP joint resurfacing arthroplasty with meniscus allograft at our institution between 2014 and 2024. Charts were reviewed to collect demographic and operative data. Outcomes, complications, and satisfaction with the procedure were reviewed at latest follow up.

**Results:** 35 procedures were performed in 31 patients within our timeframe. This included 24 females and 11 males with a mean age of 65.1 years old (range: 19-87). Procedures were performed on 8 index, 12 middle, 7 ring, and 8 small digits. Average follow-up was 11 months (range: 1-60). At latest follow up, 28 patients (80%) were overall satisfied with the procedure. There were 3 complications (8.5%) including 1 patient requiring steroid injection for painful scarring, 1 sagittal band rupture, and 1 revision surgery for persistent stiffness. 4 patients (11%) experienced significant postoperative stiffness. No infections were observed.

**Conclusion:** Hand PIP joint resurfacing arthroplasty with meniscus allograft is a novel motion-preserving procedure utilizing biologic tissue and avoiding the inherent complications of synthetic implants. Our early outcomes are favorable, with a majority of patients feeling satisfied with their surgery, and a low complication rate. Further studies will be needed to obtain longer-term data on this procedure.

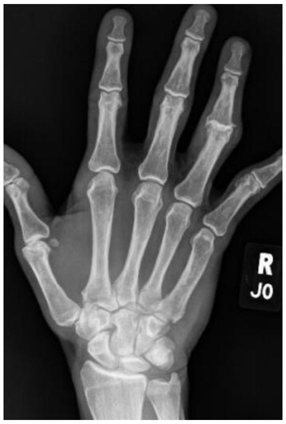

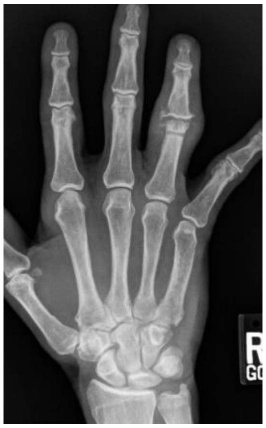

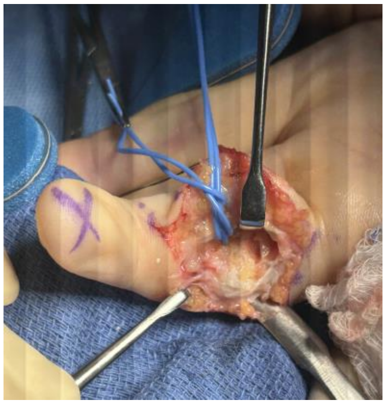

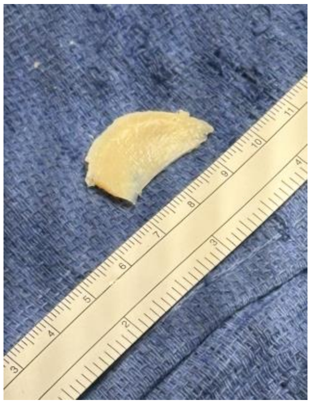

**Abstract Title (Calibri Light 11, Bold)**

Title: A bispecific antibody neutralizing both dickkopf-1 (DKK1) and sclerostin favorably impacts bone density and other characteristics in an OI mouse model.

Authors: *Baozhi Yuan[1], Xiaofeng Liu[1], Qingqiang Zeng[1], Baohong Shi[2], Xiangbo Meng[3], Jinghe Wei[1], Bingjie Wang[1], Ling Li[3], Ren Xu[2], Xinluan Wang[3], Hua Zhu Ke [1], Ann Zovein[1]*

[1.] Angitia Biopharmaceuticals (Angitia Incorporated Limited), Guangzhou, China, and Westlake Village, CA, USA
[2.] State Key Laboratory of Cellular Stress Biology, Xiamen University, Fujian, China.
[3.] Shenzhen Institute of Advanced Technology, Chinese Academy of Sciences, Shenzhen, Guangdong, China

Background: Osteogenesis imperfecta (OI) is characterized by skeletal fragility, frequent fractures, and complex bone deformities. Elevated serum concentrations of the Wnt antagonist dickkopf-1 (DKK1) have been reported in OI and appear correlated with OI illness severity. We hypothesize that neutralizing DKK1, a potent pro-resorptive Wnt inhibitor, in addition to sclerostin may result in larger bone density gains as well as in improvement of other material properties. Therefore, we evaluated the effects of a rodent bispecific antibody (rbsAb) against DKK1 and sclerostin in the oim/oim mouse of severe OI.

Methods: In two separate studies, 4-week-old oim/oim mice were treated subcutaneously (SC) with phosphate-buffered saline (PBS) or rbsAb weekly for 6 weeks. Serum biomarkers of bone metabolism, microCT, three-point bending, and fractures were evaluated.

Results: Serum levels of P1NP were significantly elevated suggesting increased bone formation. MicroCT demonstrated increased trabecular and cortical bone mineral density (BMD) with rbsAb treatment, while three-point bending mechanical tests showed rbsAb improved maximum load and stiffness. Preliminary analysis showed rbsAb improved bone quality in the oim/oim mouse model. A significant reduction in fracture numbers was also observed in the oim/oim mouse model in response to rbsAb.

Conclusions: These findings provide compelling evidence that neutralizing both DKK1 and sclerostin provides favorable improvements in bone mass, strength, and architecture, while reducing fractures in the severe oim/oim spontaneous fracture model. The data supports further clinical development of a bispecific antibody for the treatment of osteogenesis imperfecta.

### 30Kc19α: A Versatile Cell-Penetrating and Stabilizing Protein Platform for Targeted Intracellular Delivery

*Jiyeon Hong, MS[1], Nathaniel Suk-Yeon Hwang, PhD[123]**

*[1]Seoul National University, School of Chemical and Biological Engineering, Seoul, Republic of Korea, [2]Seoul National University, Interdisciplinary Program for Biochemical Engineering and Biotechnology, Seoul, Republic of Korea, [3]Seoul National University, Bio-MAX/N-Bio Institute, Institute of Bio-Engineering, Seoul, Republic of Korea*

Cell-penetrating peptides (CPPs) have revolutionized the field of intracellular drug delivery by enabling the direct translocation of macromolecular cargos across the cell membrane. These short peptides, typically rich in positively charged residues such as arginine and lysine, bypass traditional endocytosis-dependent pathways and offer enhanced cellular uptake without compromising membrane integrity. Among various CPPs, 30Kc19α, a truncated N-terminal α-helical domain of the *Bombyx mori*-derived 30Kc19 protein, has emerged as a uniquely multifunctional platform. In addition to its robust cell-penetrating ability, 30Kc19α confers enhanced solubility and stability to fused proteins, addressing two major limitations of recombinant protein therapeutics. Unlike many synthetic or virus-derived CPPs, 30Kc19α is inherently biocompatible and shows minimal immunogenicity, making it a promising candidate for therapeutic applications.

To explore its utility in bone-targeted regenerative therapy, we developed a recombinant protein by fusing 30Kc19α to RUNX2, a master transcription factor driving osteoblast differentiation. A hydroxyapatite-binding (HAB) tag was added to facilitate bone-specific accumulation. The resulting HAB-30Kc19α-RUNX2 protein was solubly expressed in *E. coli*, efficiently translocated into the nucleus of human mesenchymal stem cells (hMSCs), and successfully activated osteogenic gene expression. In vivo, systemic administration of this construct in an ovariectomy-induced osteoporosis mouse model led to selective bone accumulation, enhanced bone regeneration, and reduced off-target effects compared to non-targeted controls. These results highlight the dual functionality of 30Kc19α in enabling both intracellular delivery and bioactivity preservation of its fusion partner.

While this study demonstrates the efficacy of 30Kc19α in a bone-regenerative context, its potential applications extend far beyond. 30Kc19α can be broadly adapted to deliver diverse cargos—including therapeutic proteins, peptides, or nucleic acids—across various cell types and tissues. Moreover, by incorporating targeting ligands or molecular sensors, the platform can be engineered for cell- or disease-specific delivery, offering high precision and reduced systemic toxicity. Ongoing efforts are focused on expanding its use in other therapeutic domains such as oncology, and immune modulation. With its favorable biophysical properties and demonstrated in vivo performance, 30Kc19α represents a next-generation platform technology for targeted intracellular therapy.

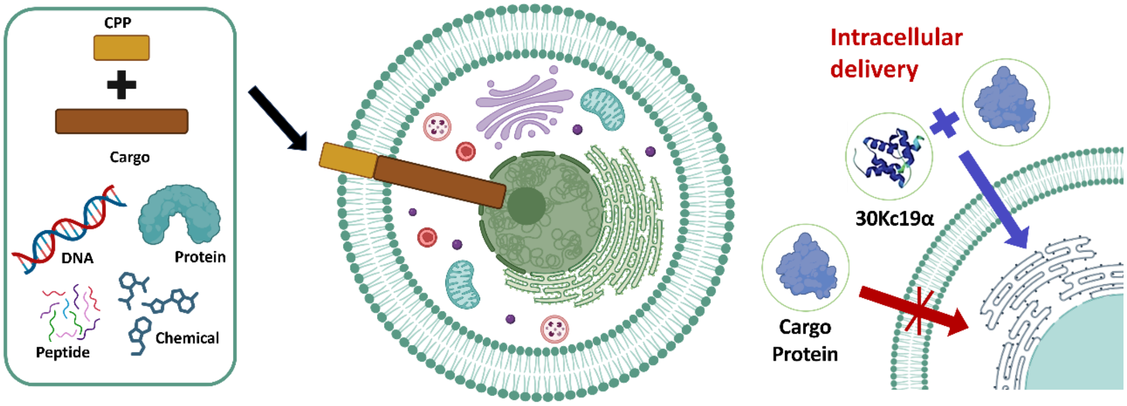

Figure 1. Schematic illustration of intracellular delivery mediated by 30Kc19α. Cell-penetrating peptides (CPPs) assist in transporting therapeutic macromolecules into the cytosol by facilitating their passage through cell membranes, without compromising the integrity of the membrane. 30Kc19α has a high cell-penetrating efficiency due to its small size and can deliver cargoes into the cell cytosol.

[1] Kim, S., Lee, H., Hong, J., Kim, S. H. L., Kwon, E., Park, T. H., & Hwang, N. S. (2023). Bone-Targeted Delivery of Cell-Penetrating-RUNX2 Fusion Protein in Osteoporosis Model. Advanced Science, 2301570.

### Comparative Efficacy of Biologic Augmentations in Gluteus Medius Tendon Repair is lacking: A Systematic Review

Yuan Liu[1,2], Matthew Allen[1], Rahul Kumar[1], Stefano Bini, MD[1]

[1] Department of Orthopaedic Surgery, University of California San Francisco, San Francisco, CA, USA
[2] Touro University California College of Osteopathic Medicine, Vallejo, CA, USA

Objective: Gluteus medius tendon tears are a widely recognized source of lateral hip pain and weakness. While surgical repair often leads to symptom relief, outcomes may be less favorable in cases involving chronic or high-grade tears. To improve healing, a variety of biologic augmentation strategies have been explored, including platelet-rich plasma (PRP), platelet-rich fibrin matrix (PRFM), collagen patches, dermal allografts, synthetic scaffolds, and bone marrow aspirate concentrate (BMAC). However, the effectiveness of these biologics in promoting gluteus medius tendon healing remains uncertain. This study aimed to systematically review published clinical outcomes associated with these techniques.

Methods: A systematic literature search was conducted through PubMed, Cochrane Library, and Embase for English language studies published between 2000 and 2025 reporting on the surgical repair of gluteus medius tendon tears with biologic augmentation. Data extracted included study design, type of biologic intervention used, and postoperative clinical and imaging outcomes.

Results: A total of six studies comprising 221 hips were included, evaluating the use of platelet-rich fibrin matrix (PRFM), collagen patch scaffolds, acellular dermal allografts, and synthetic mesh augmentation in gluteus medius tendon repair. All studies reported improvements in patient-reported outcomes following surgical repair, regardless of augmentation. However, the two available comparative studies found no significant differences in pain or function between augmented and unaugmented repairs. Case series of collagen patch augmentation demonstrated favorable structural healing in early clinical outcomes, while synthetic mesh combined with muscle transfer showed improvement in patients with irreparable tears.

Conclusion: While biologic augmentation in gluteus medius tendon repair appears safe and technically feasible, more data needs to be published relative to the results obtained with commonly used orthobiologic augmentation to determine if and under what circumstances such treatment provides consistent clinical advantage over standard repair. Given the limited number of high-quality comparative studies, no specific augmentation technique can be routinely recommended as superior to other techniques at this time.

### Joint Preservation Two Years after Arthroscopy-Guided Decompression and Biomaterial Augmentation for Subchondral Knee Lesions

*Austin M. Looney, M.D.*[1]*, Raj Pandya, M.D.*[1]*, Ruturaj S. Patil, B.S.*[1]*, Zahraw Shah, B.S.*[1]
[1]*Atlanta Orthopaedic Institute, Atlanta, GA*

Magnetic-resonance imaging identified subchondral bone-marrow lesions in forty-two consecutive knees (mean age 59.5 ± 12.9 years; 31 percent male) treated between August 2018 and June 2023 by arthroscopy-guided decompression with biomaterial augmentation (Fig. 1). Patients were observed for a minimum of twenty-four months. Kaplan–Meier analysis showed 92.5% arthroplasty-free survival at two years (95% CI, 78.6%–97.5%) (Fig. 2) with an incidence of 4.9 conversions per 100 person-years (95% CI, 1.8–10.7). Survival curves were similar across Kellgren–Lawrence grades (log-rank $\chi^2$ = 2.03, P = 0.73) (Fig. 3); multivariable Cox proportional-hazards modelling confirmed no significant effect of KL grade, age, or body-mass index on conversion risk (all P > 0.40). Median International Knee Documentation Committee scores rose from 25 (IQR 17–34) to 56 (IQR 46–78) (P < .001), and Knee injury and Osteoarthritis Outcome Scores increased from 42.9 (34.2–50.0) to 65.3 (58.8–77.2)(P < .001); overall Visual Analogue Scale pain ratings declined markedly from 8 [IQR, 7–9] to 3 [IQR, 2–5](P < 0.001). Targeted intervention therefore affords durable pain relief, functional improvement, and a joint-preservation probability exceeding ninety-two percent, benefits that appear independent of global radiographic degeneration.

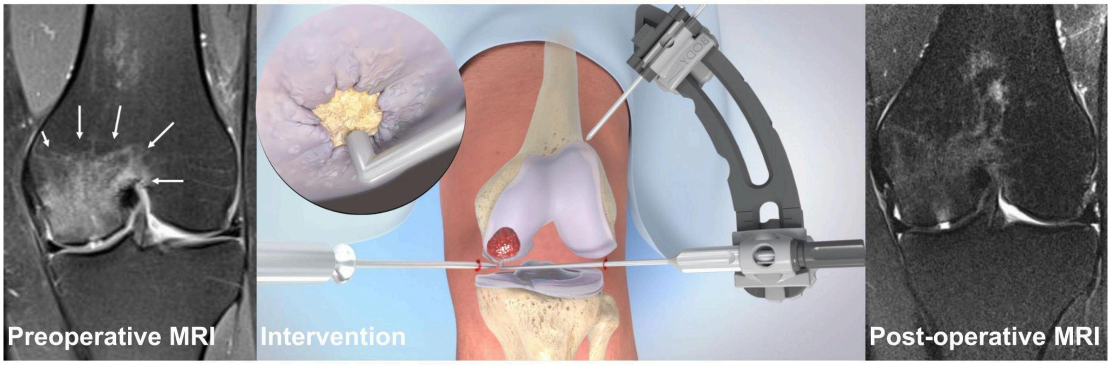

Figure 1

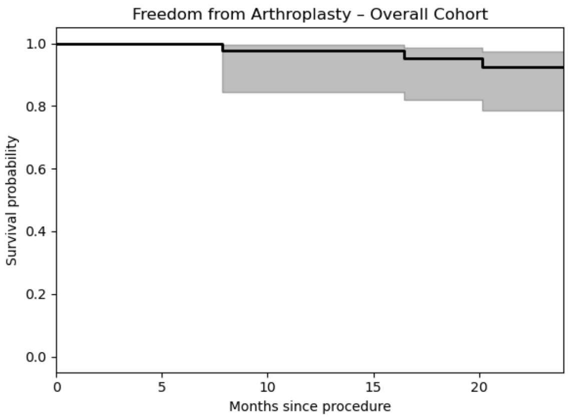

Figure 2

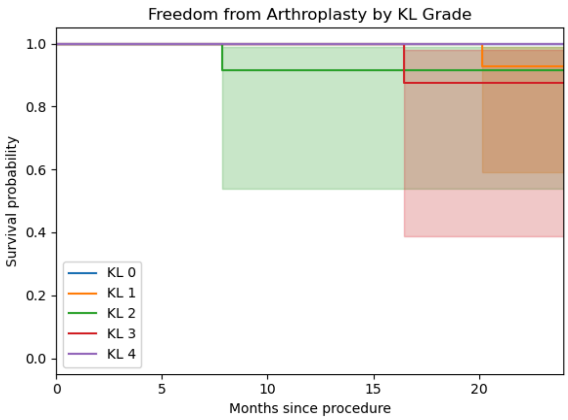

Figure 3

**Outcomes of Arthroscopy-Guided Knee Subchondral Bone Marrow Lesion Do Not Significantly Differ by Kellgren-Lawrence Grade**

*Austin M. Looney, M.D.[1], Raj Pandya, M.D.[1], Ruturaj S. Patil, B.S.[1], Zahraw Shah, B.S.[1]*
*[1]Atlanta Orthopaedic Institute, Atlanta, GA*

Magnetic-resonance imaging identified subchondral bone-marrow lesions in forty-two consecutive knees (mean age 59.5 ± 12.9 years; 31 percent male) treated between August 2018 and June 2023 by arthroscopy-guided decompression with calcium-phosphate augmentation (Fig. 1). Patients were observed for a minimum of twenty-four months. Median International Knee Documentation Committee scores rose from 25 (IQR 17–34) to 56 (IQR 46–78) (P < .001), and Knee injury and Osteoarthritis Outcome Scores increased from 42.9 (34.2–50.0) to 65.3 (58.8–77.2)(P < .001); overall Visual Analogue Scale pain ratings declined markedly from 8 [IQR, 7–9] to 3 [IQR, 2–5](P < 0.001). Improvement did not vary with Kellgren-Lawrence grade in either tibiofemoral compartment (Table 1). These findings show that targeted treatment of bone-marrow lesions provides durable pain relief and functional gains irrespective of global radiographic degeneration, supporting its use as a distinct joint-preserving strategy within knee osteoarthritis care.

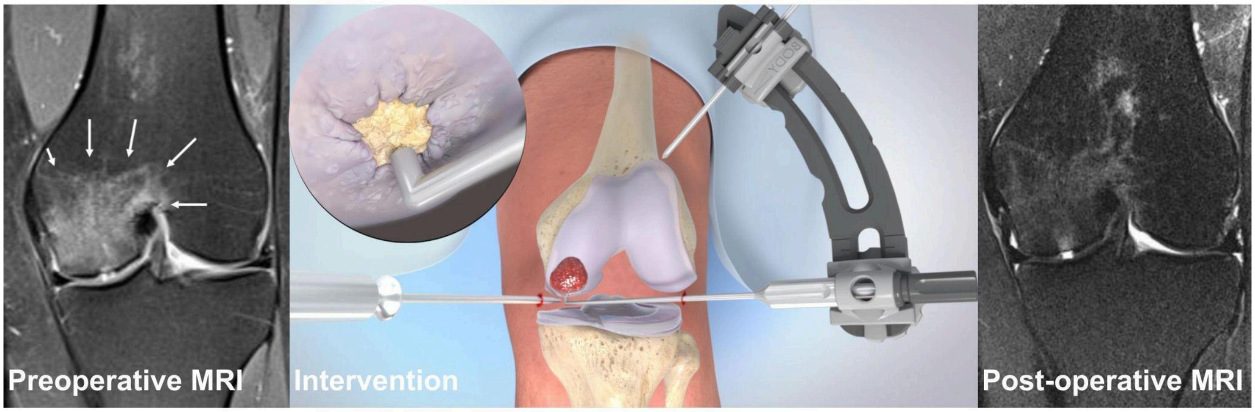

Figure 1

| Location | KL grade | Change in IKDC score No. (%) | Median | IQR | *P* value | Change in KOOS score No. (%) | Median | IQR | *P* value |
|---|---|---|---|---|---|---|---|---|---|
| Medial lesions | | | | | .286 | | | | .227 |
| | 0 | 2 (8.0) | 37.5 | 34.25–40.75 | | 1 (4.3) | 27.5 | - | |
| | 1 | 10 (40.0) | 47.0 | 19.0–53.0 | | 10 (43.5) | 32.7 | 12.8–34.5 | |
| | 2 | 6 (24.0) | 22.5 | 10.5–30.75 | | 5 (21.7) | 19.8 | 4.4–20.7 | |
| | 3 | 7 (28.0) | 20.0 | 11.5–27.0 | | 7 (30.4) | 7.9 | 2.3–17.8 | |
| | 4 | 0 (0.0) | - | - | | 0 (0.0) | - | - | |
| Lateral lesions | | | | | .492 | | | | .591 |
| | 0 | 3 (37.5) | 41.0 | 32.5–53.5 | | 3 (37.5) | 25.2 | 19.7–42.1 | |
| | 1 | 1 (12.5) | 34.0 | - | | 1 (12.5) | 34.5 | - | |
| | 2 | 2 (25.0) | 48.0 | 44.5–51.5 | | 2 (25.0) | 39.3 | 32.2–46.3 | |
| | 3 | 1 (12.5) | 19 | - | | 1 (12.5) | 8.9 | - | |
| | 4 | 1 (12.5) | 46 | - | | 1 (12.5) | 25.9 | - | |

Table 1

**Age and Serum Vitamin D Levels in Relation to Fall Risk**

*Isaac Edwards, B.S.[1], Cameron Miller, B.S.[1], Lucas Edwards[2]*
*Texas Tech University Health Sciences Center School of Medicine, Lubbock, TX, USA[1], Brigham Young University, Provo, UT, USA[2]*

**Background:** Falls are a major contributor to musculoskeletal injury and morbidity, particularly among older adults. Vitamin D has been widely studied for its role in bone remodeling, skeletal integrity, and fall prevention, but the relationship between serum levels and actual fall risk remains complex[1]. Understanding this relationship in a nationally representative population may clarify potential targets for musculoskeletal disease prevention. **Methods:** Data were obtained from the National Health and Nutrition Examination Survey (NHANES) 2021–2023[2], combining datasets on vitamin D biomarkers, balance and fall history, and demographic characteristics. Fall history was assessed via self-report, sorted into fallers and non-fallers. Serum 25-hydroxyvitamin D levels were measured using liquid chromatography-tandem mass spectrometry (LC-MS/MS). Participants with complete data for fall history, vitamin D levels, and age were included (n = 3,270). Descriptive and stratified analyses examined the relationship between fall status, age, and vitamin D levels. **Results:** Among the included participants, 212 (6.5%) reported at least one fall in the prior year. Fallers were significantly ($p < 0.001$) older than non-fallers (mean age: 54.2 vs. 47.1 years). Unexpectedly, fallers also had higher mean serum 25-hydroxyvitamin D levels (88.2 ng/mL) compared to non-fallers (76.2 ng/mL). A Mann–Whitney U test confirmed this difference was statistically significant ($p < 0.001$). These findings suggest a potential confounding influence of age, vitamin D supplementation, or other health-related behaviors. Despite the established importance of vitamin D in musculoskeletal function, elevated serum levels were not inversely associated with fall history in this population-based analysis. **Conclusion:** These findings underscore the complex interplay between age, vitamin D status, and fall risk in musculoskeletal health. Higher vitamin D levels among fallers may reflect increased medical attention, supplementation, or underlying comorbidities common in older populations. Notably, the elevated vitamin D status observed in fallers may suggest that these individuals are being proactively treated for musculoskeletal conditions, which in itself signals an elevated baseline risk for falls. This presents a potential confounding effect, where those at greater musculoskeletal risk are both more likely to fall and more likely to be supplemented. These data highlight the importance of contextualizing biomarkers within clinical risk profiles and advancing mechanistic research in skeletal disease prevention.

**References**

[1]Wei FL, Li T, Gao QY, Huang Y, Zhou CP, Wang W, Qian JX. Association Between Vitamin D Supplementation and Fall Prevention. Front Endocrinol (Lausanne). 2022 Aug 10;13:919839. doi: 10.3389/fendo.2022.919839. PMID: 36034418; PMCID: PMC9399608.

[2]Centers for Disease Control and Prevention (CDC). (2024). *National Health and Nutrition Examination Survey: August 2021–August 2023 Examination Data*. U.S. Department of Health and Human Services. https://wwwn.cdc.gov/nchs/nhanes/search/datapage.aspx?Component=Examination&Cycle=2021-2023

### Surfactant-Stripped Micellar Nanocarriers Enable Targeted Senescence Modulation in Osteoarthritis

*Zachary Varrenti[1], Yiting Song[1], Jacqueline Shine[2], Mingqian He[2], Jayishnu Roy[1], Brian O. Diekman[2], Jonathan F. Lovell[1], Ramkumar T. Annamalai[1*]*

*[1]University at Buffalo, Buffalo, NY, [2]University of North Carolina, Chapel Hill, NC*

**Introduction**: Osteoarthritis (OA) remains the predominant cause of pain and disability among older adults, yet no disease-modifying therapies are currently available; existing interventions are limited to symptomatic pain management. Addressing this critical therapeutic gap, we have developed surfactant-stripped, kinetically frozen (SKiF) nanocarriers engineered for the high-capacity encapsulation of hydrophobic agents in aqueous media. This platform is particularly suited for targeted intra-articular delivery, where injection volume is constrained. We encapsulated MDL-800, a potent Sirtuin-6 activator, within poloxamer-based SKiF nanocarriers to selectively target senescent chondrocytes, a key pathological driver in OA (**Fig. 1A**). These nanocarriers demonstrate robust MDL-800 loading, exceptional stability, efficient cellular uptake and demonstrate therapeutic potential, collectively positioning SKiF as a promising modality for OA intervention.

**Methods:** SKiF nanocarriers were synthesized via a surfactant-stripping protocol, in which MDL-800 (10 mg/mL, organic solvent) was added to 10 wt% poloxamer F127, followed by solvent removal, washing, and filtration (100 kDa, 0.22 µm). Nanocarrier size and morphology were characterized by dynamic light scattering (DLS) and transmission electron microscopy (TEM), respectively. Drug loading was quantified by spectrophotometry. ATDC5 chondrocytes were rendered senescent with doxorubicin (100 nM, 4 days) or 10 Gy irradiation, then treated with SKiF (40 or 200 µM) for 4 days; controls included poloxamer and DMSO. Senescence was assessed by SA-β-gal assay and qPCR. In vivo efficacy was evaluated in a murine anterior cruciate ligament rupture (ACL-R) model of OA.

**Results:** SKiF nanocarriers exhibited a mean diameter of 26.2 ± 2.6 nm (DLS) and achieved a MDL-800-to-poloxamer molar ratio of 19.2:1 (mass ratio 1:1) (**Fig. 1A, B**). The formulation remained stable for at least one week at room temperature and several weeks at –20°C and –80°C (**Fig. 1C**). *In vitro*, SKiFs were rapidly internalized by ATDC5 chondrocytes, localizing to endosomes within hours (**Fig. 1D**), and reduced cellular senescence by 12.5% compared to controls (SA-β-gal assay). *In vivo*, SKiF-MDL-800 restored pain tolerance in ACL-R mice to levels comparable to uninjured controls by week 8 (n = 12, $p < 0.05$), whereas PBS-treated mice showed no recovery (**Fig. 1E**). MicroCT revealed greater osteophyte formation and tibial subchondral bone loss in PBS-treated mice versus SKiF-treated animals (**Fig. 1F**).

**Discussion:** These findings establish SKiF nanocarriers as a promising disease-modifying platform for OA, targeting senescence-associated pathways central to disease progression. The surfactant-stripping approach enables high drug loading with minimal carrier, potentially reducing off-target effects. Efficient cellular uptake and significant reduction in senescence markers in vitro, together with functional and structural improvements in vivo, underscore the therapeutic potential of MDL-800–loaded SKiF. Future studies will focus on long-term efficacy, safety, and translation to clinical models.

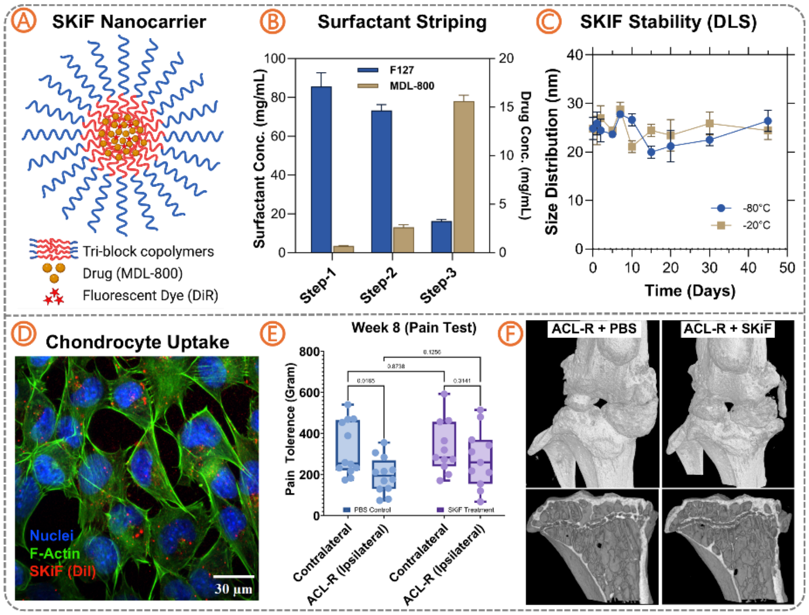

***Figure 1: SKiF nanocarrier synthesis and characterization.*** *(**A**) Schematic of SKiF nanocarrier structure and composition. (**B**) High-concentration MDL-800 loading via surfactant-stripping. (**C**) DLS analysis showing SKiF stability at –20°C and –80°C over several weeks. (**D**) Rapid uptake of MDL-800–loaded SKiFs by chondrocytes in vitro. (**E**) In ACL-R mice, SKiF-MDL-800 restored pain tolerance by week 8 to levels comparable to uninjured knees (n = 12, p < 0.05), unlike PBS controls. (**F**) MicroCT images showing greater osteophyte formation and tibial subchondral bone loss in PBS group versus SKiF-treated mice.*
